# Dispersion-generated concentration gradients and cyclic flow for reaction optimisation and kinetic analysis

**DOI:** 10.1039/d6re00378h

**Published:** 2026-10-14

**Authors:** Dawid Drelinkiewicz, Stephen T. Alston, Thomas Durand, Richard J. Whitby

**Affiliations:** a School of Chemistry and Chemical Engineering, Faculty of Engineering and Physical Sciences, University of Southampton Southampton UK rjw1@soton.ac.uk; b Jealott's Hill International Research Centre, Syngenta Ltd. Bracknell UK

## Abstract

The use of dynamic flow experiments where variables such as reaction time, light exposure, temperature or component concentration vary during a continuous run rapidly generates reaction data. The variation of concentration of one or more components is a powerful technique but requires dynamic control of flow rates of several pumps, something generally not available in commercial systems. Herein we present a method for varying component concentrations without needing dynamic flow rate control, readily implemented in simple flow systems. Taylor dispersion of a plug of a reaction component as it passes through a tube generates a concentration gradient which, when combined with the other components and passed through a reactor with continuous monitoring of output, allows the effect of all concentrations up to the maximum of the dispersed component to be examined in a single flow experiment. The dynamic flow methodology was successfully applied to kinetic studies of first-order and second-order processes, and to investigate the influence of the concentration of palladium, ligands, and additives on the Heck and Sonogashira coupling reactions. Cyclic reciprocating and unidirectional flow were used alongside dispersion-generated concentration gradients to allow simultaneous investigation of the effect of species concentration and residence time on an S_N_Ar process. Further methodology refinements include the introduction of concentration gradient clipping, the use of glass bead-filled reactors to reduce dispersion, and the use of multiple concentration gradients of different reactants in a single unidirectional cyclic flow experiment.

## Abstract 2

Dispersion-generated concentration gradients allow the effect of a range of concentrations of one component on a reaction outcome to be determined in a single flow experiment, *e.g.* the effect of ligand/metal ratios on the Sonogashira reaction.

## Graphical abstract

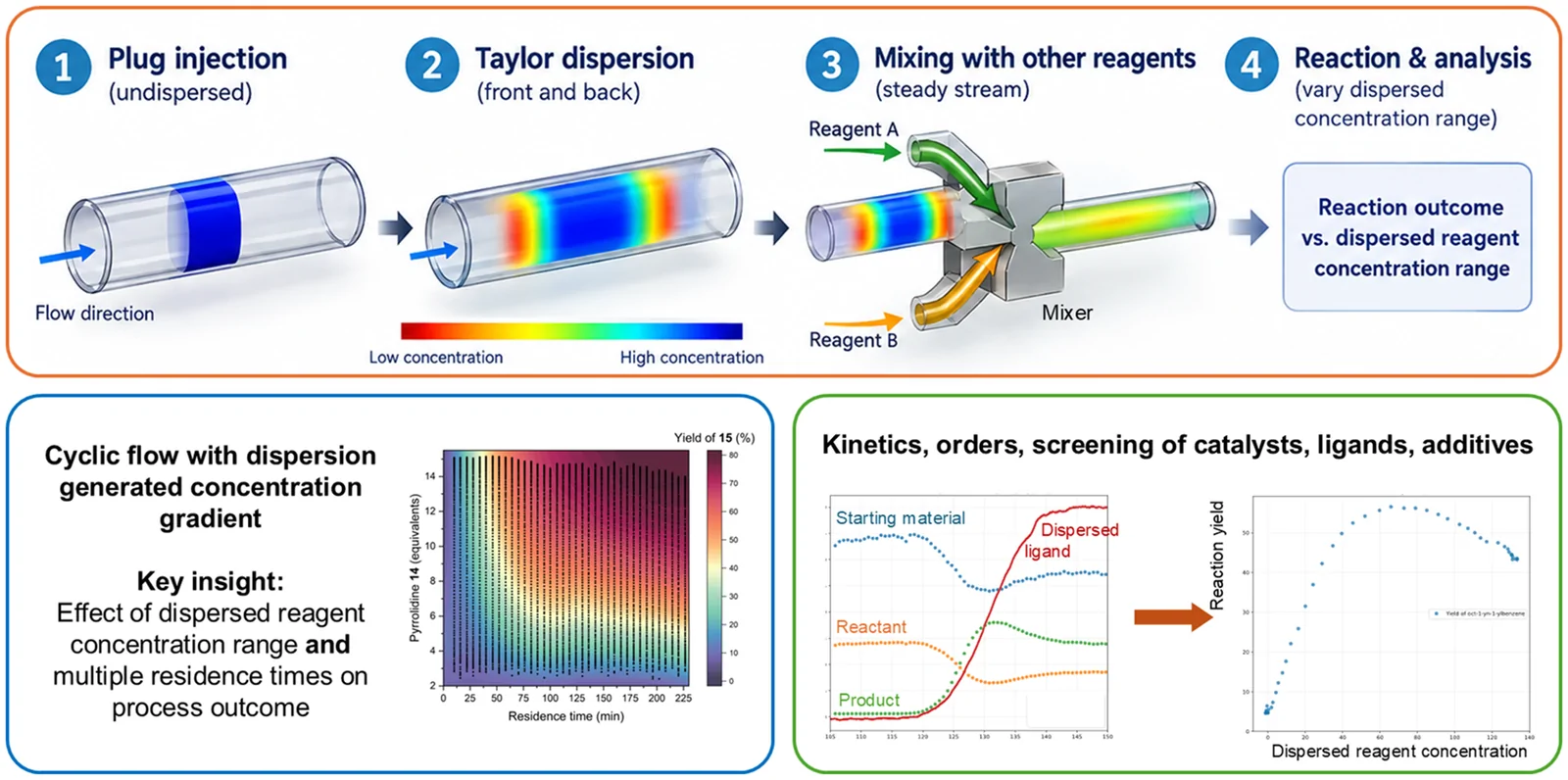

## Introduction

The presence of continuous flow processing techniques in chemistry laboratories has vastly expanded over the past two decades, and they are having an increased influence in both academia and fine chemical research, development, and manufacturing.^1–3^ To achieve sustainable chemistry, the use of continuous processing technology as a tool for data-rich experimentation to provide efficient and straightforward approaches for reaction development, optimisation, and kinetic analysis is important.^4–9^

Normally, process optimisation or mechanistic study in flow is conducted utilising steady-state conditions allowing the determination of reaction outcome for a single set of reaction conditions. Many experiments with different sets of reaction conditions are needed to give satisfactory amounts of data. The use of transient/gradient/dynamic flow experiments has emerged recently as an attractive alternative, in which the values of one or more parameters (such as temperature, residence time, irradiation time, or process stoichiometry) are screened in a single continuous flow experiment.^3,10–12^ Such methodologies allow for a drastic reduction in the amount of material used, waste generated, and time spent on the experimentation. Additional benefits include high-density data generation by coupling flow platforms with in-line analytical techniques, such as UV-vis, Raman, near IR, or IR spectroscopy, and off-line methods such as GC, HPLC, MS, or NMR. Fully automated operation through dedicated software or custom-written scripts further increases the attractiveness of flow chemistry.^13–17^

Various methods utilising the potential of flow chemistry have been developed to study processes and gain kinetic information in flow mode.^18–27^ Methods such as push-out or switch-off restore the ‘batch’ advantage of easy sampling over various residence or irradiance times, respectively, without the need for multiple experiments.^28–33^ Investigations into the effect of the concentration of reaction components on the process outcomes such as yield or impurity profile are crucial in kinetic analysis, reaction development, and optimisation; however, in batch mode only one set of stoichiometries can be screened in each experiment, requiring multiple runs to effectively screen the available chemical space. A variable-flow-rate (VFR) methodology, in which the flow rate of several pumps is dynamically controlled to vary the concentration of a reaction component, has been successfully used in various kinetic investigations,^25,26,28,34,35^ exploration of the chemical space,^27,36^ determination of reaction models,^37,38^ and process optimisation.^39,40^ However, in order to use the VFR method, one has to be equipped with a binary pump setup for creating concentration gradients as well as dedicated software to control the flow rates.

Herein we present a novel and straightforward methodology which allows the determination of the effect of continuously varying the concentration of a studied component(s) on a process by utilising the often-undesired Taylor dispersion to generate a concentration gradient.^41,42^ This method is simple to execute and requires only additional flow tubing to be added to the standard flow setup, making it usable in virtually all meso-scale flow chemistry systems. Moreover, the developed methodology has been used within a circular flow setup, allowing for simultaneous screening of multiple component concentrations and residence times.

## Results and discussion

### Dispersion phenomena

When carrying out flow chemistry experiments using meso-scale flow systems (*e.g.* 0.5–1.0 mm ID tubes and 1.0–10.0 mL min^−1^ flow rates), laminar flow occurs without turbulent mixing between fluid layers.^43^ When injecting plugs of materials in such systems, the extent to which these spread out (mix with solvent adjacent to the plug edges) is important. One mechanism is Taylor dispersion which occurs *via* the combined actions of the variation of the cross-sectional velocity of the fluid and radial diffusion of the molecules. Under laminar flow, the fluid velocity decreases radially from a maximum at the centre of the channel to a minimum at the channel wall. Such a parabolic velocity distribution (Fig. 1, bottom left) spreads the injected sample plug down the tube (Fig. 1, top). Radial diffusion, and when the tube is curved Dean circulation (Fig. 1, bottom right), reduces the along-tube spreading as the solute molecules move between the layers of different linear velocity.

**Fig. 1:**
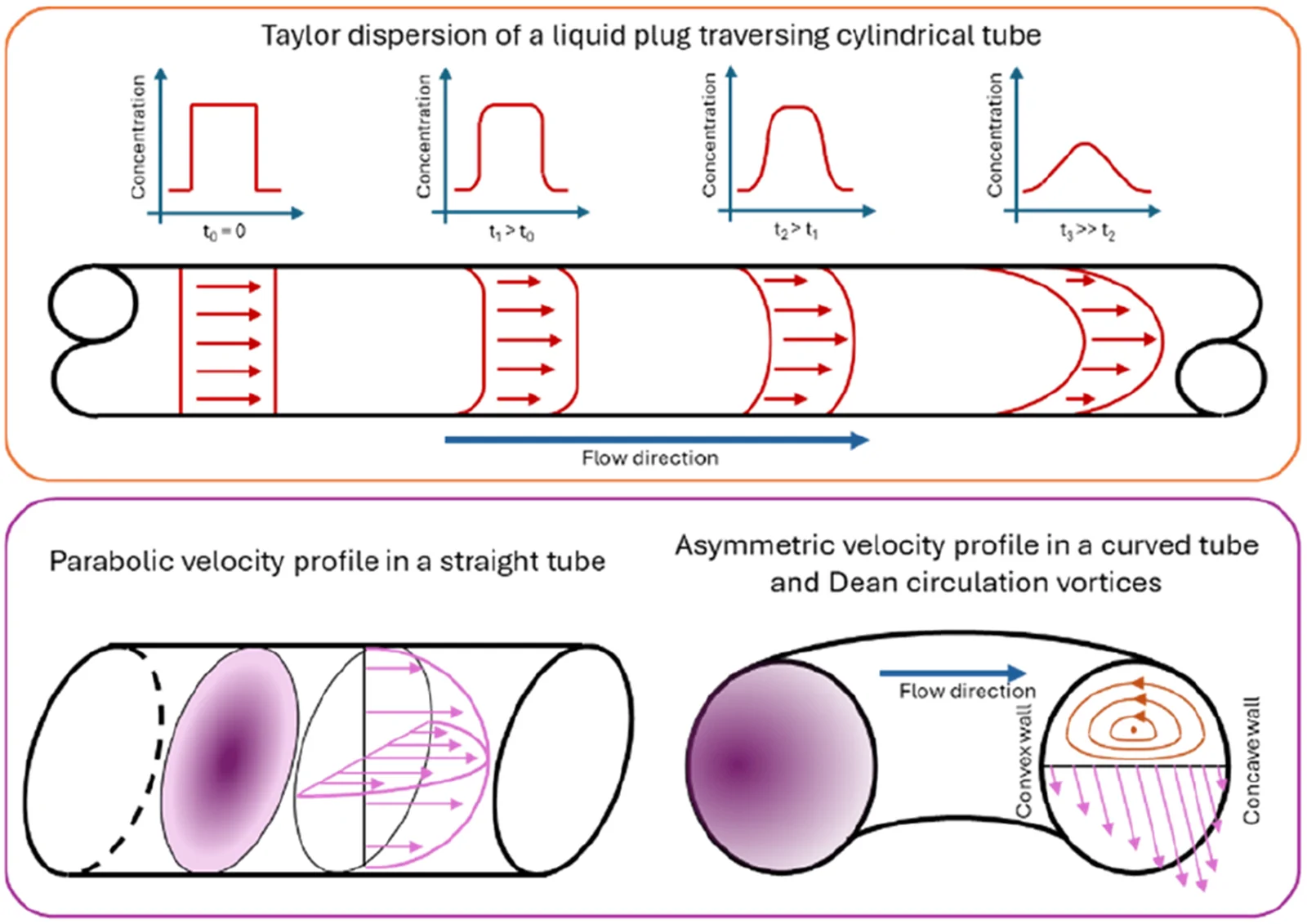
Top: Graphical representation of a plug being dispersed upon flowing through a cylindrical tube. Bottom: Velocity profiles in straight (left) and curved (right) tubes.

Jensen *et al.*^43^ described the importance of mixing and dispersion in various flow systems. In meso-scale flow systems the effect of dispersion is that an injected plug of material that passes through the flow system will spread out. For example, when a 10.0 mL plug passes through a 1.0 mm ID 10.0 mL stainless steel reactor at a 1.0 mL min^−1^ flow rate, the collected plug will be over 12.0 mL. Moreover, in comparison with the theoretical ‘plug flow’ concentration profile, a gradient of concentrations between 0 and *C*_0_ is observed at the beginning and the end of the injected plug (Fig. 2).

**Fig. 2:**
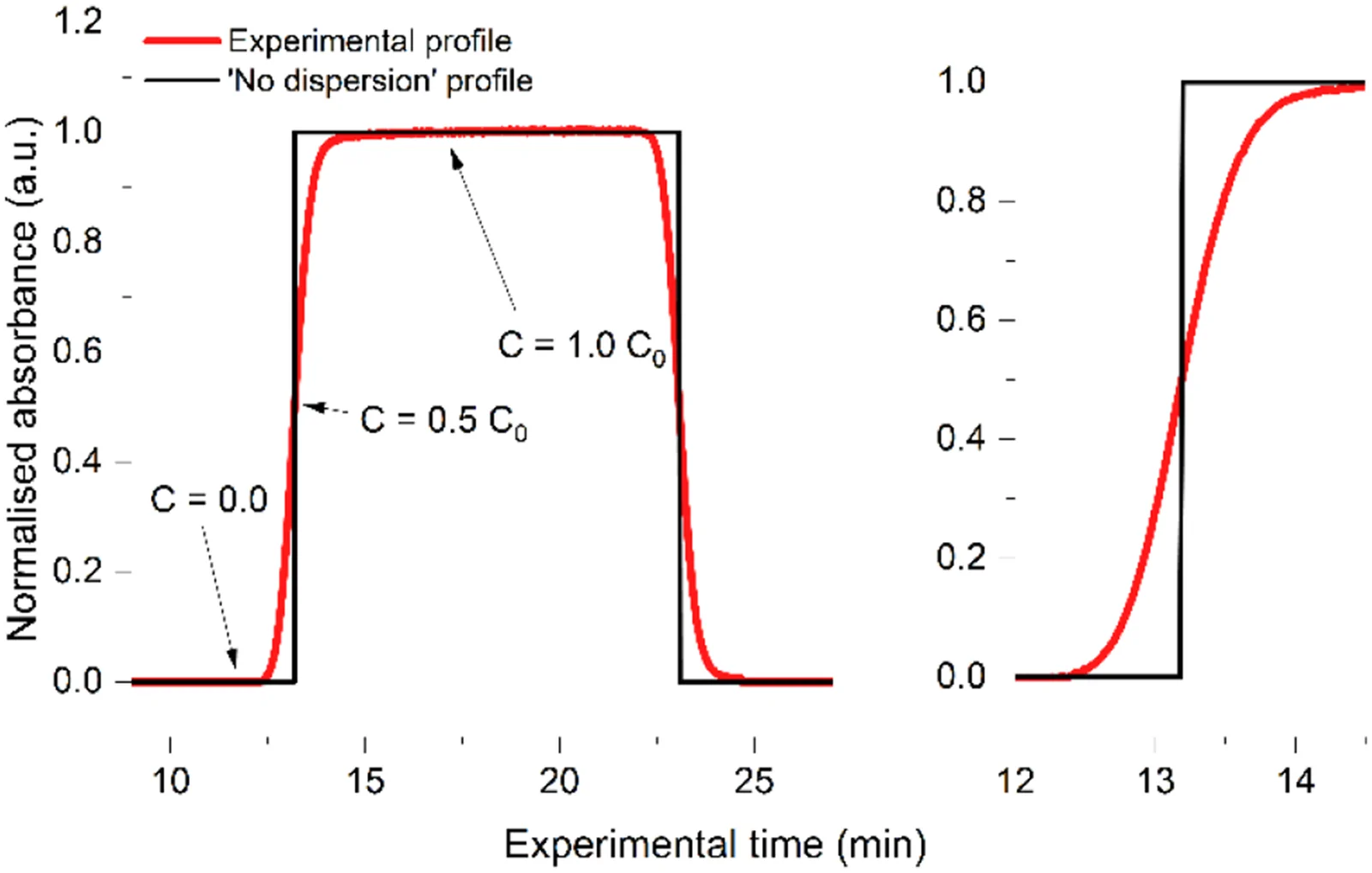
Experimental (red line) and theoretical (plug flow, black line) dispersion profiles of a 10.0 mL plug passed through a 1.0 mm ID 10.0 mL reactor at a 1.0 mL min^−1^ flow rate.

Herein, we describe how the concentration gradients generated at both the leading and trailing edges of plugs of a component can be used to evaluate the effect of all concentrations of a component on a chemical reaction in a single flow experiment with very simple apparatus and no need to control the flow rates of multiple pumps.

### Methodology

The methodology for the generation of a concentration gradient using dispersion is straightforward and involves passing a plug of the investigated reactant(s) through a tube, described here as the dispersion coil. The dispersed reagent line is then connected to the remaining reaction component(s) line(s) *via* a mixer, which ensures radial homogenization of the concentration gradient as well as thorough mixing of all reagent streams. The combined stream is then directed to the flow reactor, and the process outcome is measured using either in-line or off-line analysis. The amount of dispersion (or length of the concentration gradient) can be easily controlled by the length and internal diameter of the dispersion coil.

### Quantification of the dispersion profile

To successfully analyse data obtained using a concentration gradient, the dispersion profile must be quantified. One method is to pass a plug of a tracer, for example a UV-active compound, through the dispersion coil using the same flow setup, solvent, and flow rate as in the planned experiment while using in-line monitoring such as UV spectroscopy to observe the dispersion. Such an approach allows the determination of the dispersion profile expected in subsequent experiments and is relatively fast and straightforward to perform; however, it must be repeated if there is any significant change in the flow rate or solvent. A more reliable method is to use an inert internal standard which would undergo dispersion with the dispersed component and could be monitored with the chosen process analytical tool. In this approach the initial stock concentration of the dispersed reagent (further diluted down the flow stream) can be normalised with the dispersed internal standard signal allowing for quick determination of the concentrations. The use of an internal standard does not require prior calibration and provides a means to account for any variations in the system that might affect the dispersion profile, which enhances the reproducibility and reliability of the method.

### Determination of reaction orders *via* dispersion-generated concentration gradients

#### Thermal formation and trapping of a reactive ketene *via* retro-Diels–Alder reaction

The developed methodology for creating *in situ* concentration gradients in flow systems was initially tested in two simple model reactions. The first was the thermal fragmentation of 2,2,6-trimethyl-4*H*-1,3-dioxin-4-one 1 to a reactive acylketene 2, which was trapped with ethanol, forming a β-ketoester 3 as the final reaction product (Scheme 1).^44,45^

**Scheme 1:**
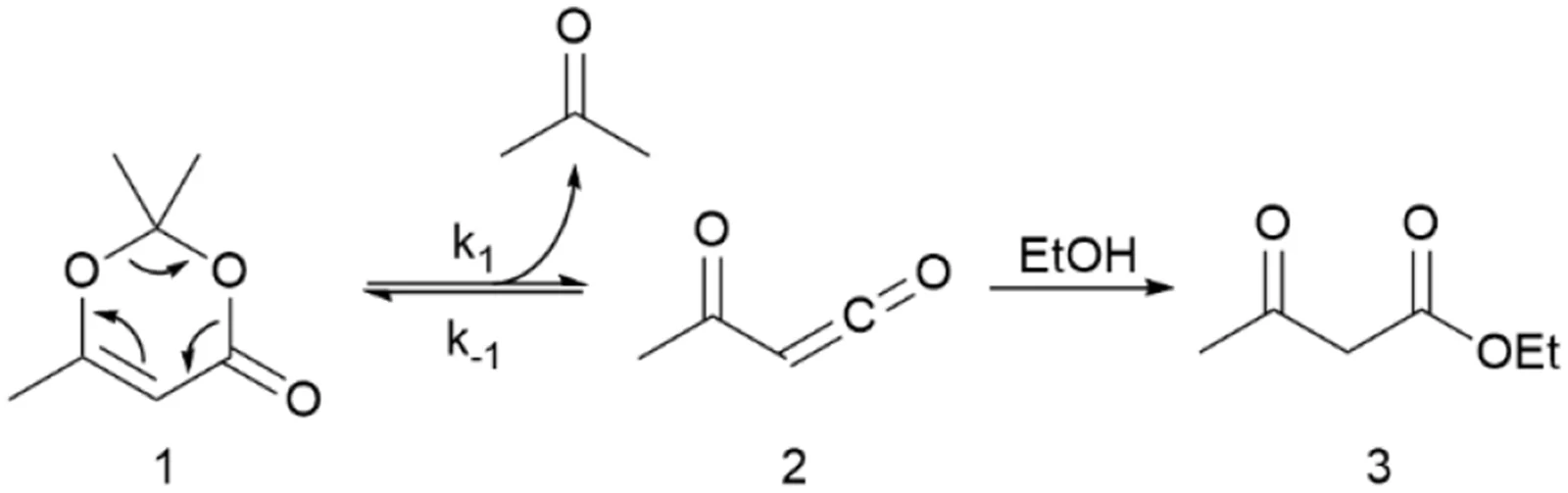
Retro Diels–Alder reaction of 2,2,6-trimethyl-4*H*-1,3-dioxin-4-one with subsequent trapping of ketene with ethanol.

This thermolysis conducted with an excess of ethanol is a first-order reaction, as the rate of the Diels–Alder reaction of acetone with the ketene intermediate 2 is not significant when compared to the rate of trapping of the ketene with ethanol (*k*_2_[EtOH] ≫ *k*_−1_[acetone]). Performing steady-state experiments for various residence times at 120 °C with off-line ^1^H NMR analysis of the reaction mixture gave a consistent first-order reaction rate constant between 1.0 and 4.0 equivalents of EtOH (*k*_1_ = 1.85 × 10^−3^ s^−1^ ± 1%).^42^
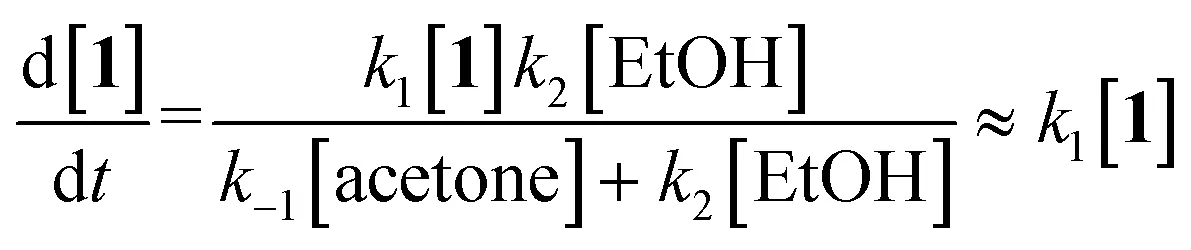
ln[1]_*t*_ = −*k*_1_*t*_R_ + ln[1]_0_A dispersion-generated concentration gradient was then applied in this transformation to obtain information on the reaction conversion dependency on the starting material concentration. The concentration gradient was generated by passing the reaction mixture through the dispersion coil, then a preheated flow reactor, cooling coil, in-line IR monitoring cell, and autosampler (Fig. 3).

**Fig. 3:**
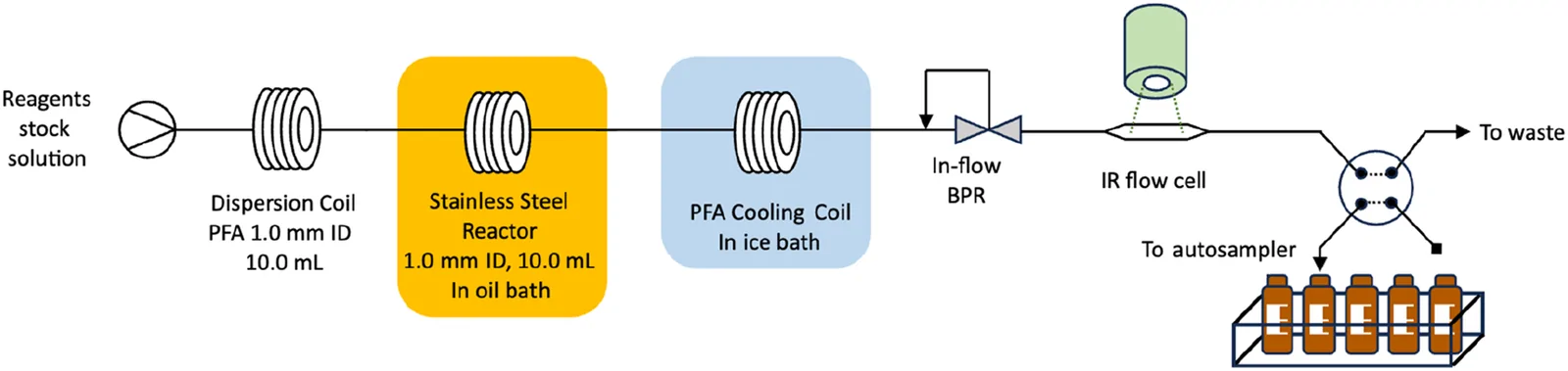
Flow setup used in investigation of reaction orders *via* dispersion-generated concentration generation gradient methodology.

Calculation of the reaction conversion shows that it is independent of the initial starting material concentration (*τ*_1/2_ = ln(2)/*k* = constant). This is well depicted by in-line IR monitoring of gradient regions (Fig. 4, dashed burgundy rectangle), where the conversion (Fig. 4, black points) remains constant while the concentration of the dioxinone 1 (Fig. 4, blue line) changes. The data from the gradient regions were extracted and by using the integrated first-order rate equation and plotting the ln[*C*_0_] *vs.* ln[*C*] a straight line of slope 1 and intercept equal to *k*·*t*_r_ was obtained (Fig. 5). From this intercept, *k* (100 °C) = 0.18 × 10^−3^ s^−1^ (with *t*_R_ = 2000 s) was obtained which is almost identical to that calculated with the “push-out” method (*k*(100 °C) = 0.23 × 10^−3^ s^−1^).^29^

**Fig. 4:**
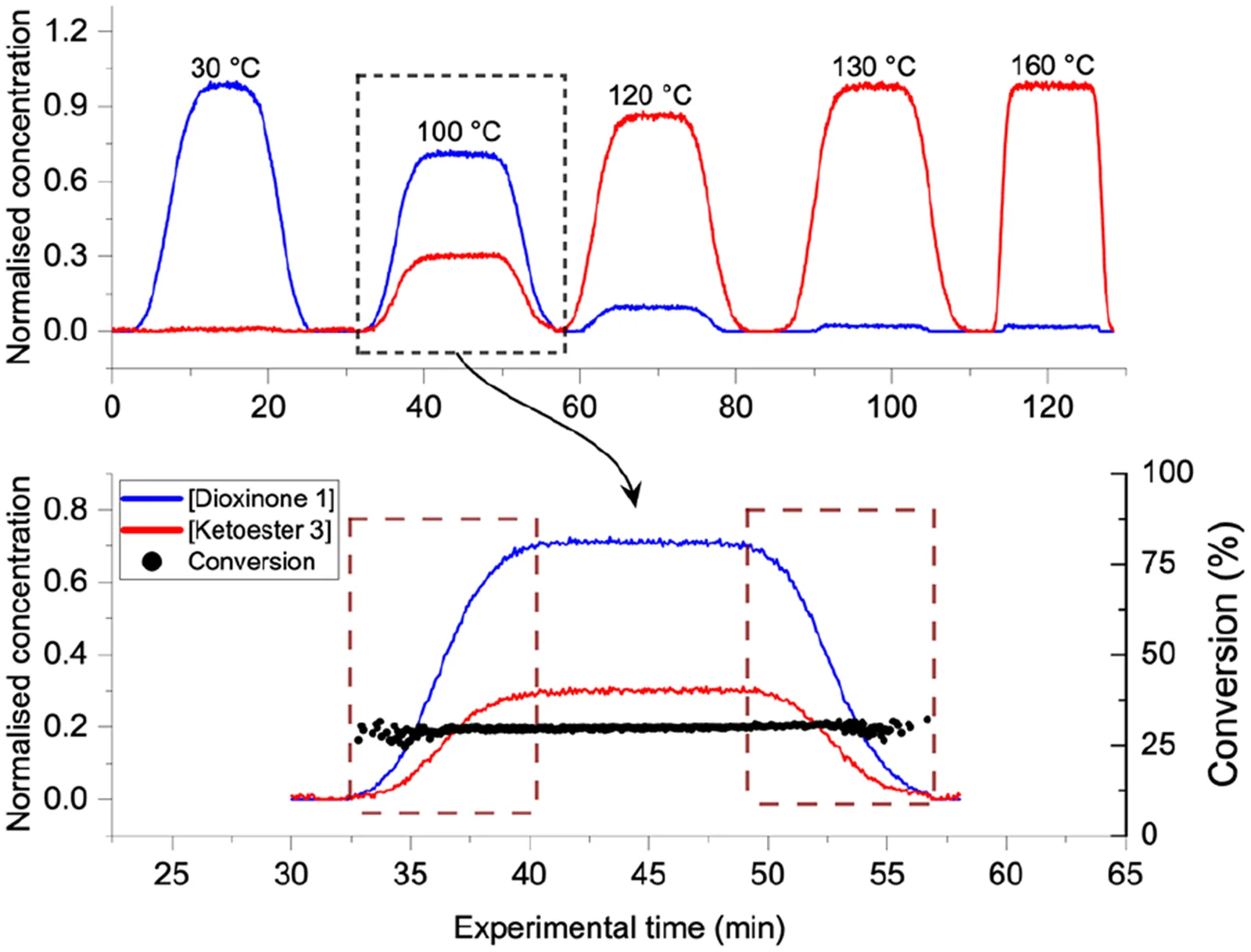
IR traces of diketone 1 (blue) and ketoester 3 (red) from in-line IR monitoring and calculated conversion of 1 (black). Dashed-line burgundy rectangle shows the gradient area where concentration of dioxinone 1 is variable.

**Fig. 5:**
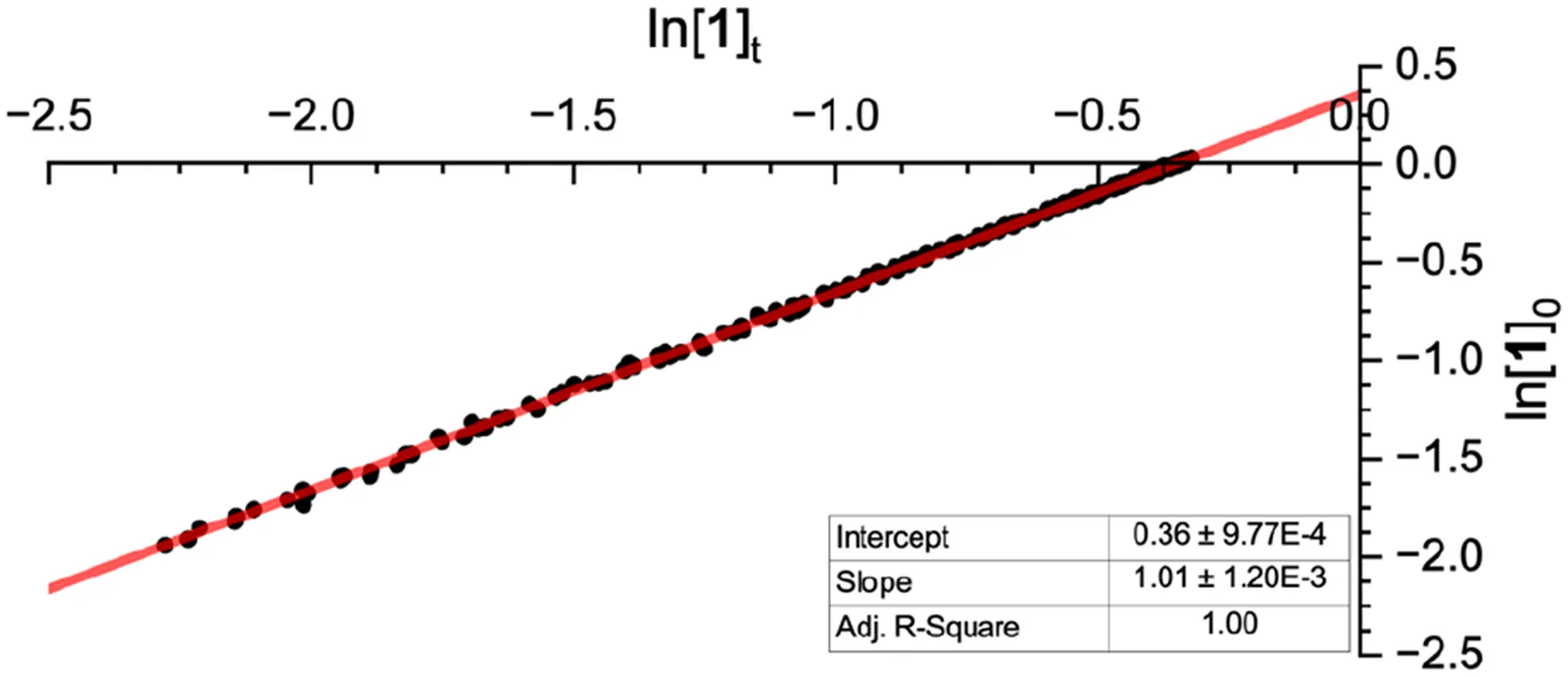
Plot of ln[1]_0_*vs.* ln[1]_*t*_ and fitted linear trend.

#### [4 + 2] Diels–Alder cycloaddition of isoprene and methyl acrylate

The second model reaction was the Diels–Alder reaction of isoprene 4 and methyl acrylate 5 to afford 4-methyl 6a (major product) and 3-methyl 6b (minor product) substituted cyclohexenes (Scheme 2), which has been utilised previously in flow investigations.^46–48^ Only one dispersion experiment was performed using the same flow setup as before (Fig. 5), where a plug containing the starting materials dissolved in toluene was injected into the flow system with the reactor heated to 200 °C. The reaction was monitored utilising in-line IR spectroscopy and calibrated off-line gas chromatography. Concentration profiles of methyl acrylate and both reaction products obtained from IR and GC monitoring were in excellent agreement (Fig. 6).

**Scheme 2:**
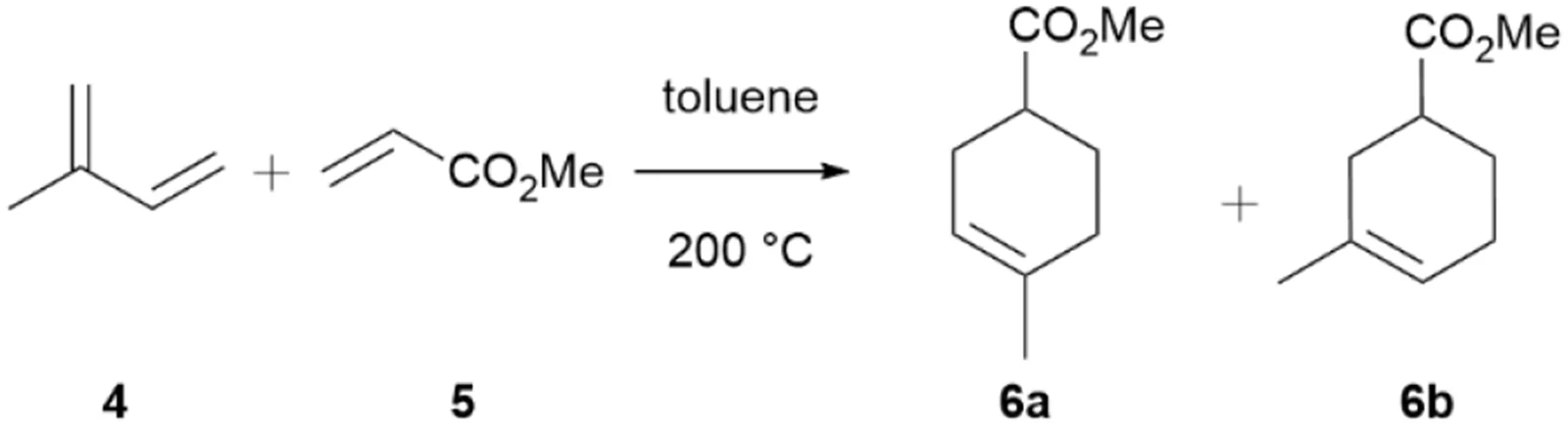
Diels–Alder reaction between isoprene and methyl acrylate.

**Fig. 6:**
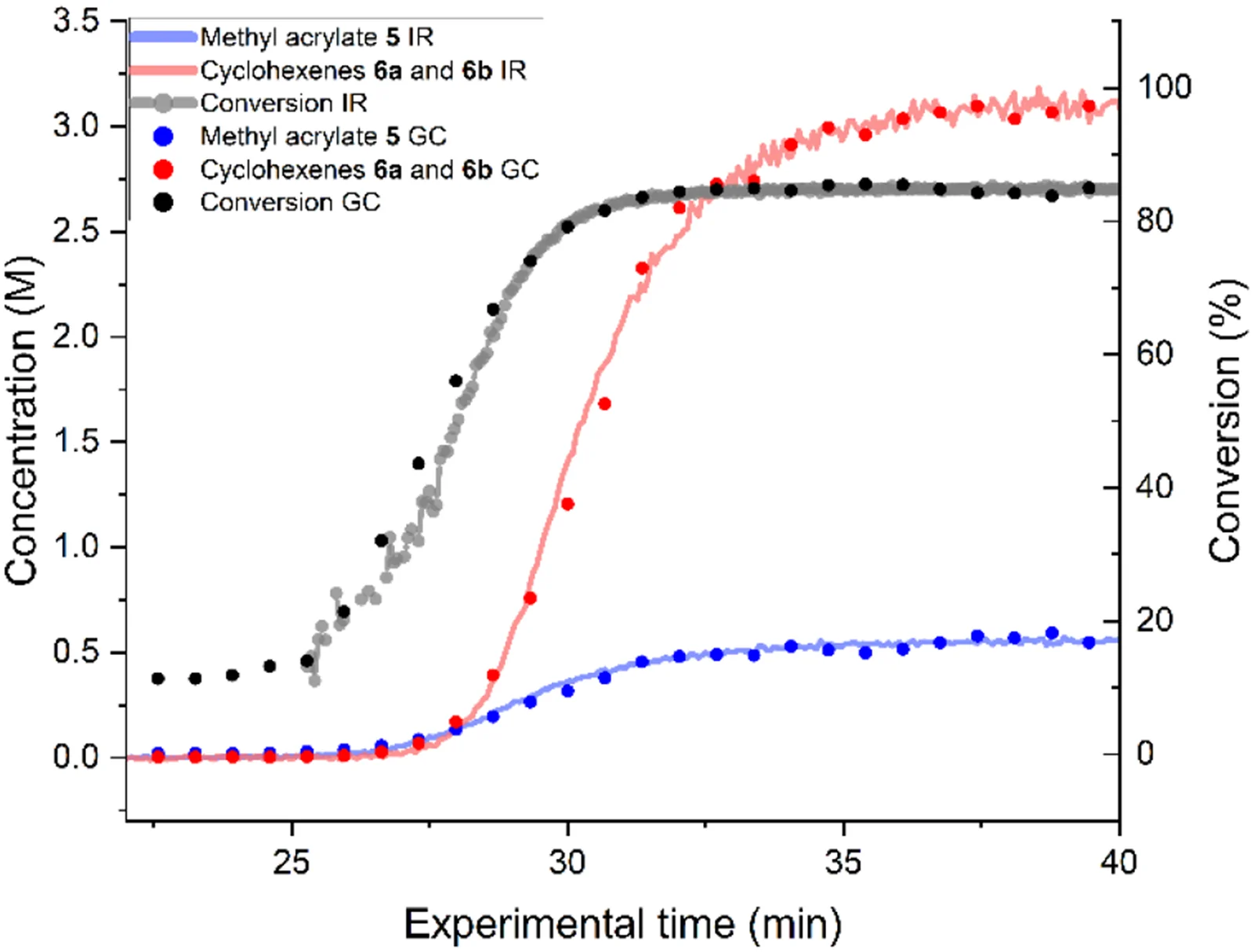
In-line IR and off-line GC traces of the investigated Diels–Alder reaction.

As opposed to the first-order reaction examined above, the conversion of the second-order reaction is dependent on the initial concentration of methyl acrylate (*τ*_1/2_ = 1/*C*_0_·*k*). Indeed, the conversion is smaller with the low initial starting material concentration and grows up to a maximum of 85% conversion for the highest concentration studied (Fig. 6). Moreover, a second-order reaction where the initial concentration of the two reagents is equal should give a linear plot of 1/*C*_*t*_*vs.* 1/*C*_0_.
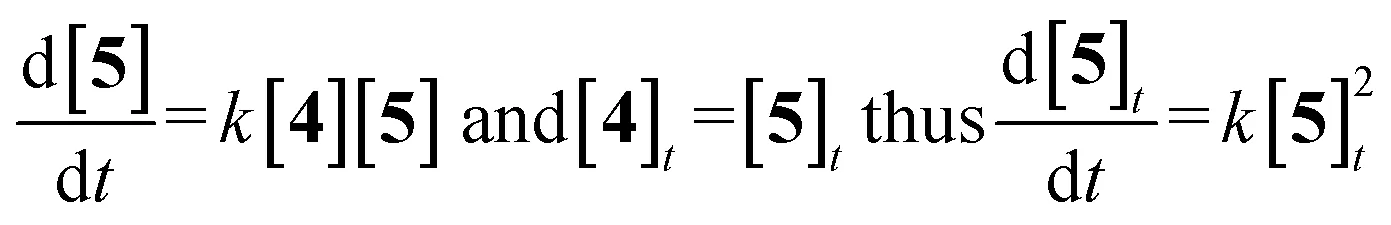

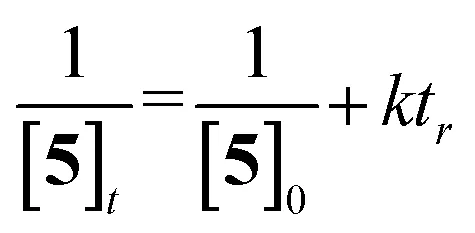
Kinetic information for the Diels–Alder reaction was extracted from the dispersion experiment, and by plotting 1/[5]_*t*_*vs.* 1/[5]_0_ (Fig. 7), a straight line with a slope of approximately 1 and intercept equal to *k*·*t*_R_ was obtained. From this intercept, the rate constant was determined to be *k*^200°C^ = 1.33 × 10^−3^ [L mol^−1^ s^−1^] (with *t*_R_ = 2000 s).

**Fig. 7:**
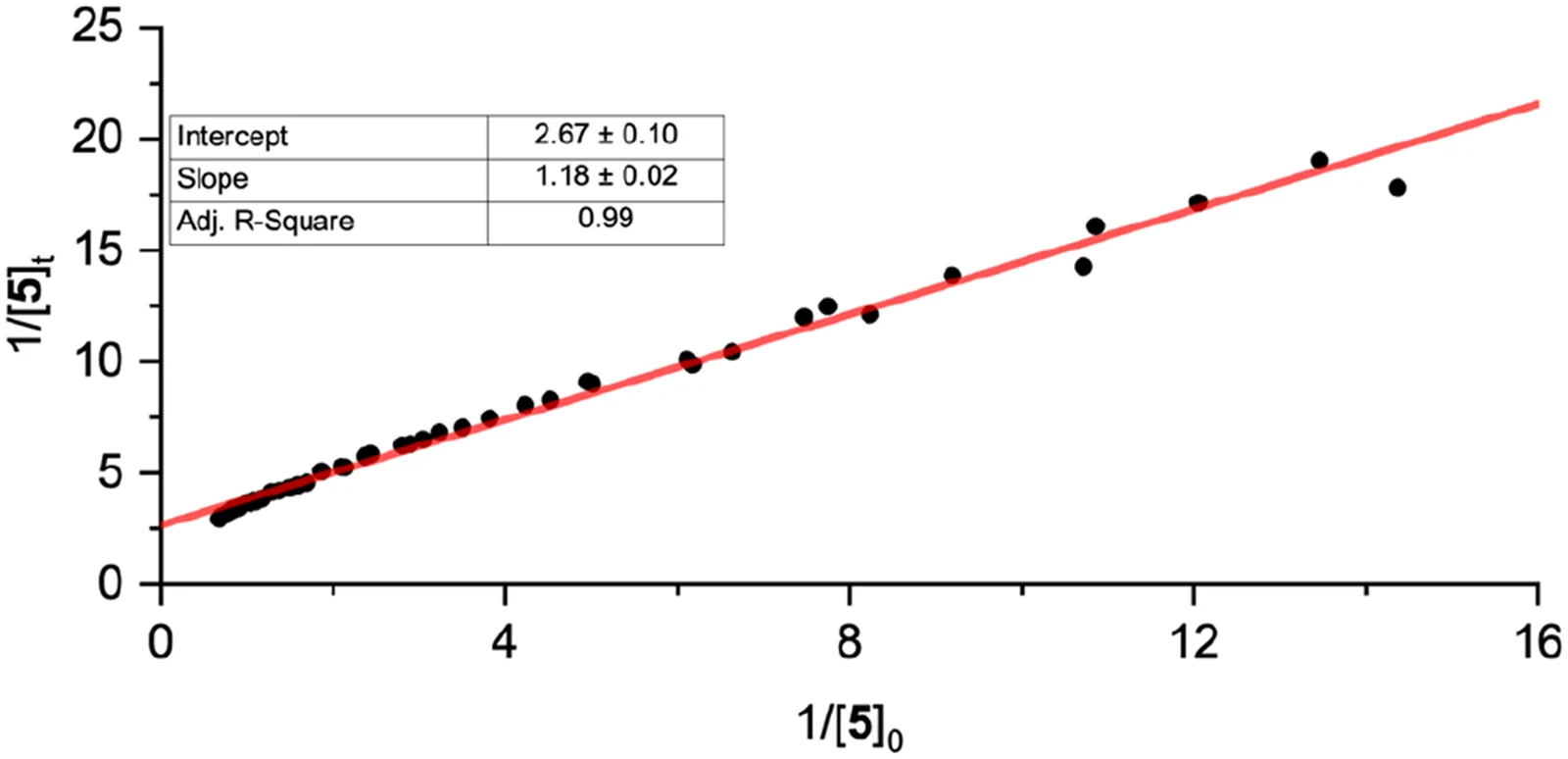
Plot of 1/[5]_0_*vs.* 1/[5]_*t*_ and fitted linear trend.

The above kinetic analysis illustrates and validates the dispersion method of generating concentration gradients. With such simple systems, the kinetics are of course readily obtained from single batch reactions by monitoring concentrations over reaction time. Sophisticated methods of using concentration *vs.* time data from batch reactions to elucidate reaction kinetics *via* visual or AI comparison with models have been developed recently.^49–51^ The method we have developed, having both the advantages and disadvantages of being in flow, generates complementary data. A particular strength is the determination of initial rates of reactions as the starting concentration of a component is varied. It should also be particularly useful for probing the roles of components which are not consumed in the studied reaction (for example catalysts, ligands, other additives) for which time domain data is less useful.

### Study on the effects of the concentrations of palladium, ligands and additives on the rate of the Heck cross-coupling

The Heck coupling reaction is a well-established process allowing alkene and (pseudo)halide moieties to be joined, resulting in the formation of a new C–C bond and was chosen to demonstrate how dispersion-generated concentration gradients could be used to probe the effect of palladium, ligands, and additive concentrations. Various protocols for performing the Heck coupling have been developed; however, a homogeneous Pd catalysis was desired to avoid the issues arising from the formation of Pd black nanoparticles.

Investigations of the effects of varying concentrations of reaction components on the product formation rate were conducted in a flow system (Fig. 8) where the component to be studied was passed through the dispersion coil and then joined with the remaining reaction components, directed to the reactor, followed by in-line IR spectroscopy. Samples were collected and analysed using calibrated GC to determine experimental outcomes, whereas in-line monitoring was used for determination of the dispersion profiles in the used flow setups, when a plug of dye was passed through the flow system.

**Fig. 8:**
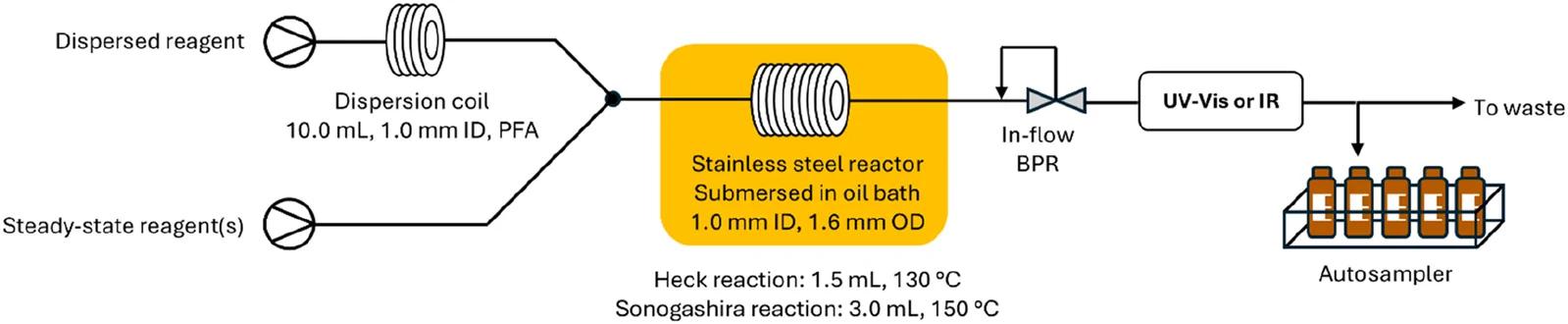
Flow setup used in the Heck (in-line IR) and Sonogashira (in-line UV-vis) coupling reactions with concentration gradient *via* dispersion methodology.

#### Palladium source screening

Initial studies on the Heck reaction involved screening of various palladium sources. Heck coupling between iodobenzene 7 and *n*-butyl acrylate 8 forming *n*-butyl cinnamate 9 (Scheme 3) was investigated, with conditions similar to those reported by Cyr *et al.*^52^ Dispersion-generated concentration gradients of Pd(OAc)_2_, [Pd(allyl)Cl]_2_, Pd(PPh_3_)_2_Cl_2_, and Pd(dppf)Cl_2_ were combined with a flow stream containing the remaining reactants to give concentrations between 0.0 and 0.45 mol% palladium.

**Scheme 3:**
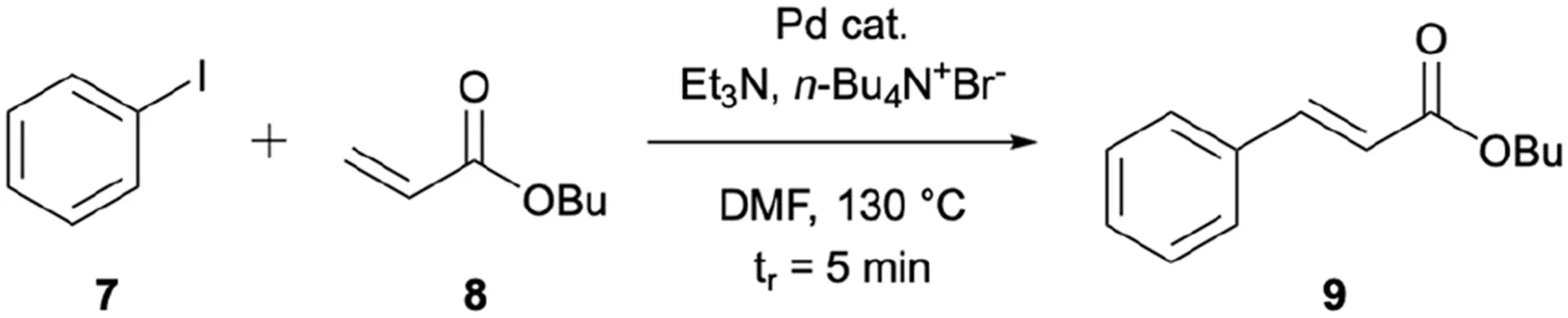
Heck cross-coupling between iodobenzene and *n*-butyl acrylate.

Palladium(ii) diacetate was found to be the optimal catalyst, with 100% yield of 9 at 0.225 mol% and 5 min residence time. Pd(dppf)Cl_2_ reached nearly 90% yield at 0.45 mol%, however, both the allylpalladium chloride dimer and Pd(PPh_3_)_2_Cl_2_ gave approx. 40% yields at the maximum investigated concentration of 0.45 mol% (Fig. 9).

**Fig. 9:**
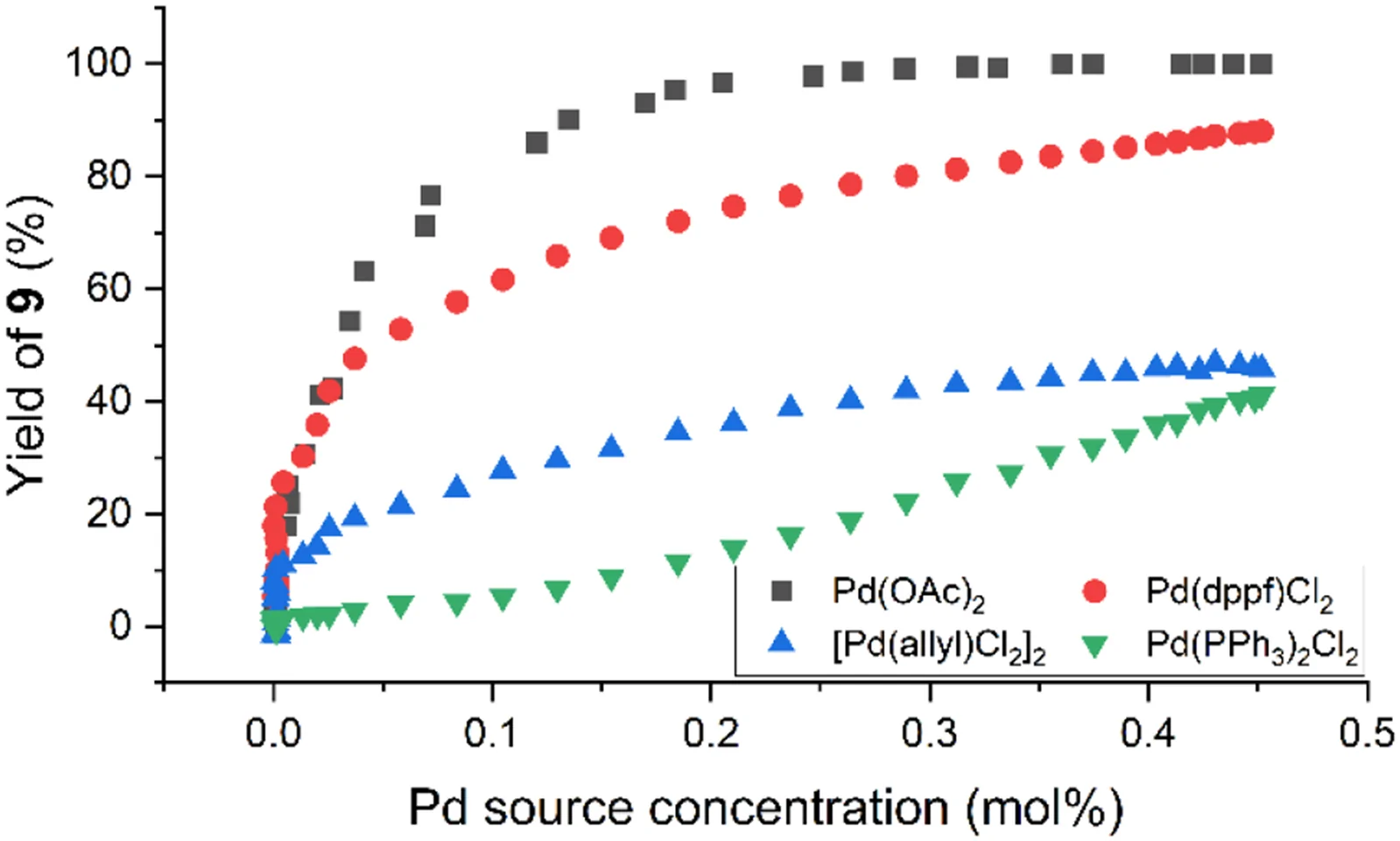
Yield of 9 at 5 min residence time for different concentrations of various Pd sources used. Yields were determined using calibrated GC.

#### Comparison of dispersion-generated and variable-flow-rate generated concentration gradients

The results were validated by comparing concentration gradients obtained using the dispersion-generated methodology described above (Fig. 10, red circles) with those obtained using variable-flow-rate methodology (Fig. 10, blue triangles). The flow setup was modified to simultaneously include a dispersion coil as well as two binary pumps (Fig. 11) to allow for execution of both methods without any changes in the flow setup. Results obtained from dispersion-generated and VFR-generated concentration gradients show excellent agreement (Fig. 10).

**Fig. 10:**
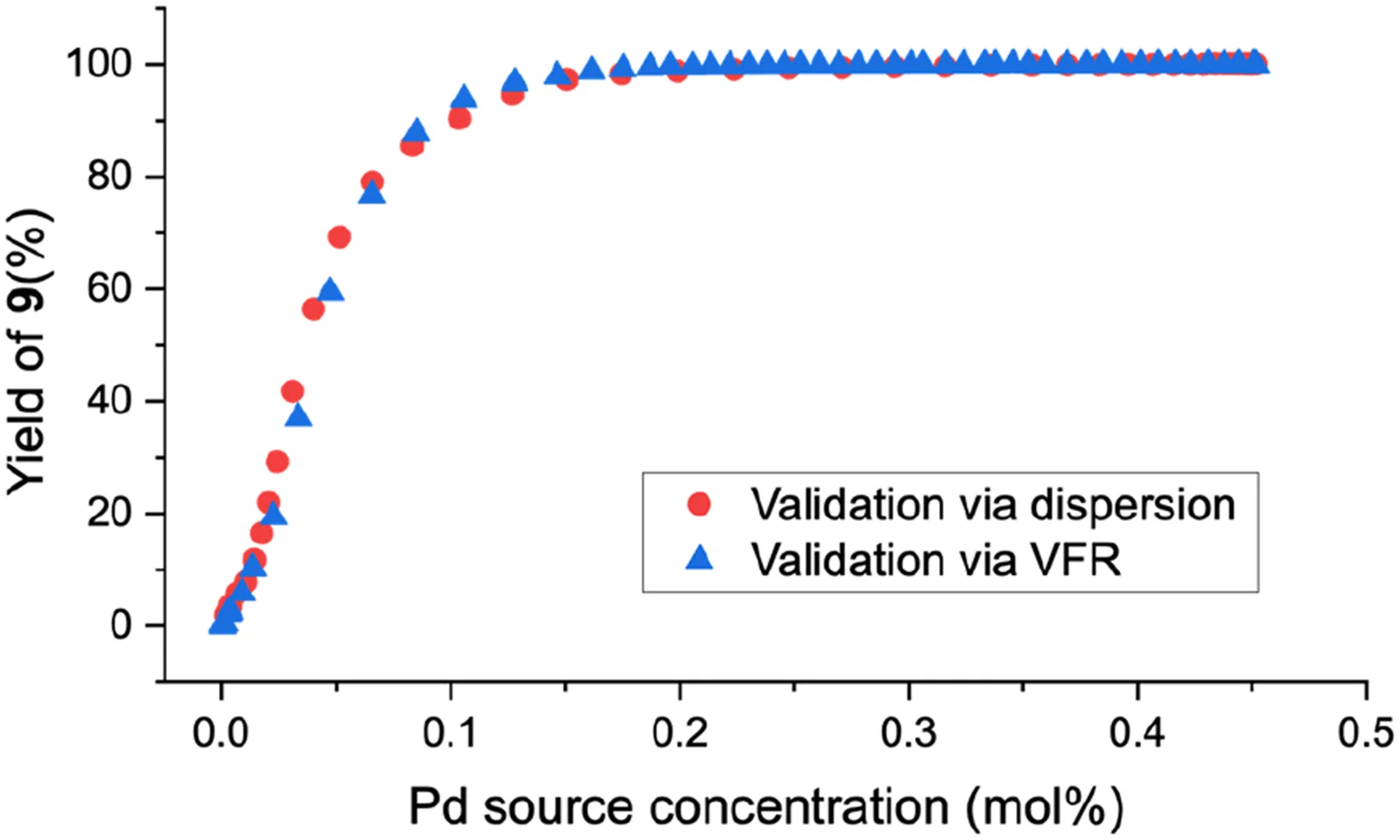
Overlaid yield of 9 (*t*_r_ = 5 min) obtained from dispersion-generated concentration gradient (red) and variable flow rates (blue) used for method validation. Yields were determined using calibrated GC.

**Fig. 11:**
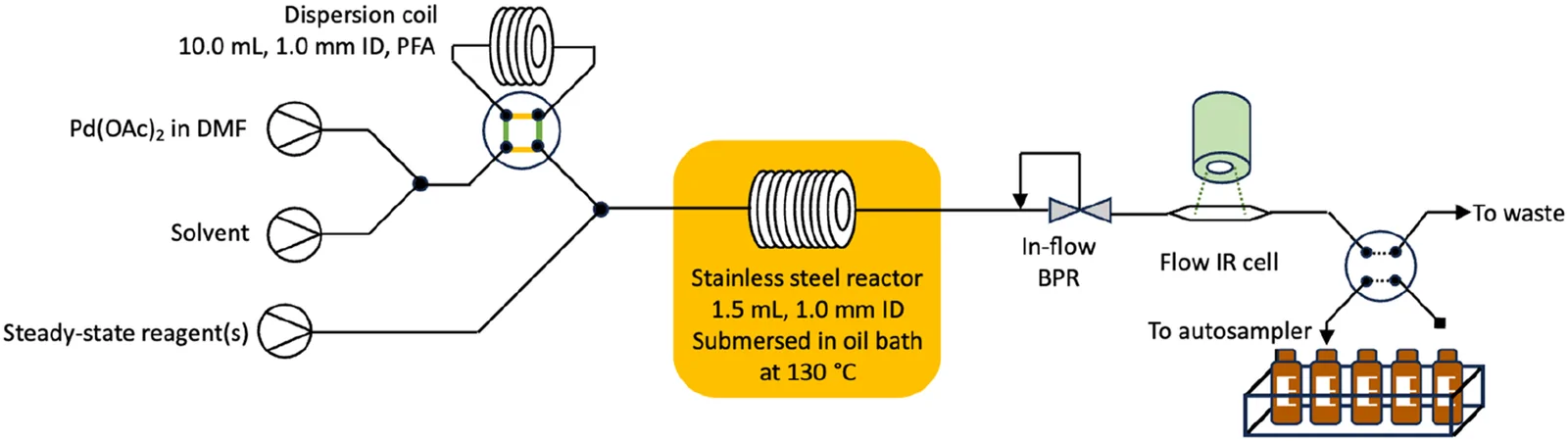
Flow setup used for validation of Pd(OAc)_2_ catalysed Heck coupling using dispersion of binary pump setup, without the need for flow path change.

#### Screening of concentration gradients of phosphine- and nitrogen-containing ligands in the Heck coupling reaction

Phosphine and amine ligands are commonly used to modify the performance of palladium catalysts, although their influence is strongly dependent on the studied process, catalyst precursor, reaction conditions, and substrate combination. Accordingly, the ligandless palladium(ii) diacetate system served as a reference for examining the effect of ligand addition over a range of concentrations from zero up. Concentration gradients of various phosphines commonly employed in Pd-catalysed coupling reactions were generated using dispersion and the effects on the studied Heck coupling reaction were investigated. The same flow setup as used for screening of Pd sources was used (Fig. 8); however, the dispersion line was connected to a stock solution of ligand and was later joined with the lines delivering the remaining reaction components.

A number of phosphines were investigated (Fig. 12) with their maximum concentration reaching 18.0 eq. wrt 0.225 mol% of Pd, with the exception of dppf for which equivalence was limited to 6.2 eq. due to solubility limits. Under the reaction conditions investigated, none of the ligands examined provided an improvement in reaction rate over the ligand-free catalyst. Addition of P(*o*-tolyl)_3_ resulted in a slight linear decrease in the rate of formation of 9 down to 77% at 18.0 eq., probably due to formation of dimeric [P(*o*-tolyl)_3_PdOAc]_2_ species which can undergo reductive elimination to form a catalytically active Pd^0^ species.^53^ Addition of the remaining phosphines resulted in a rapid decrease of the yield of 9 at 5 min residence time, with TTBP·HBF_4_, dppf, PPh_3_, dtbpf, and dppe equivalencies of 10.0, 4.5, 4.2, and 3.0, and 2.0, respectively, to reach <10% yield. These results show that the active palladium species generated from Pd(OAc)_2_ is faster in the absence of added coordinating ligands, but does not provide information on the effect of ligands on catalyst stability or the yields obtainable with longer reaction times.

**Fig. 12:**
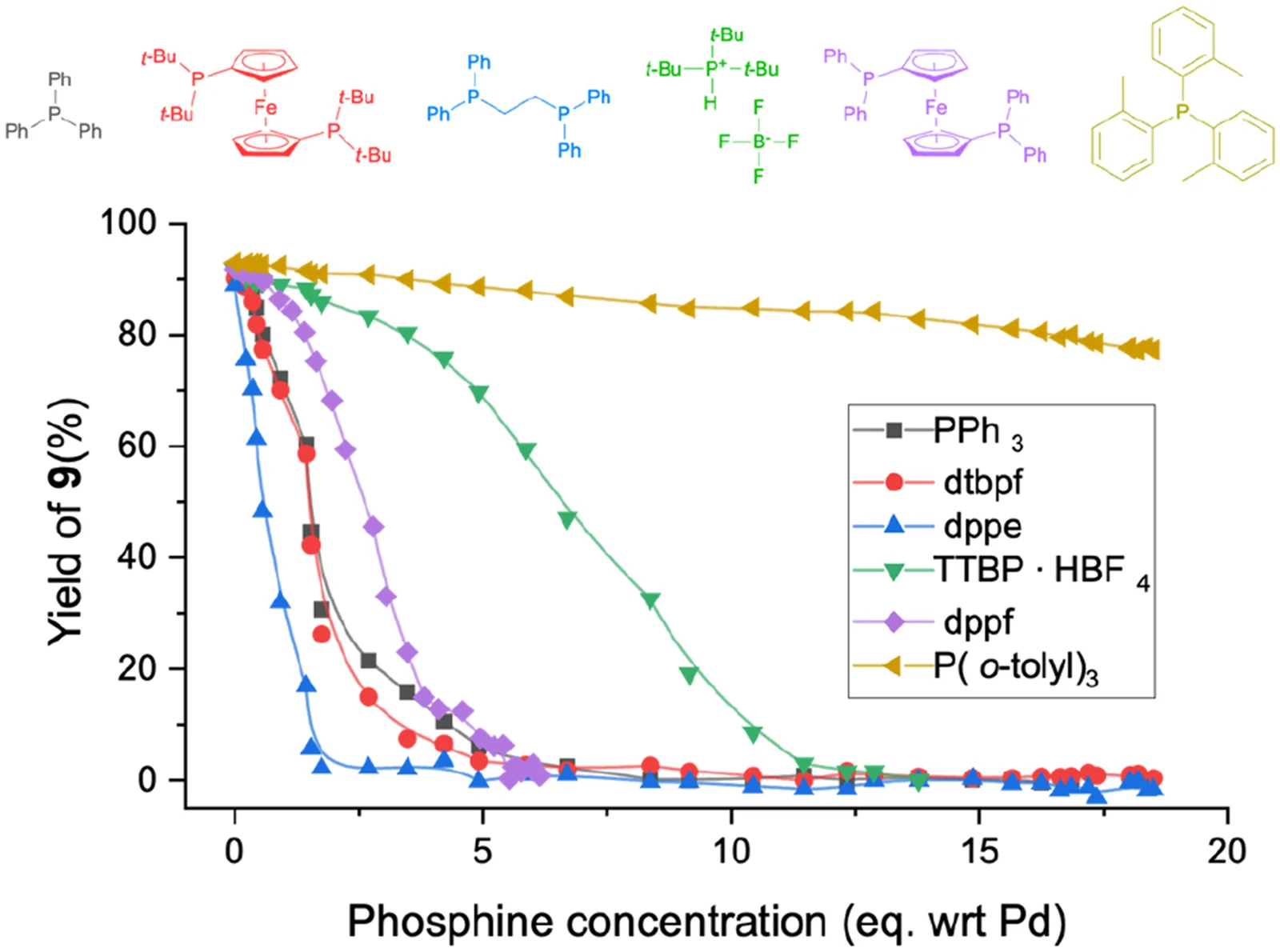
Phosphines screened in the Heck reaction and their effect on the yield of 9 at 5 min residence time and 0.225 mol% Pd catalyst. Yields were determined using calibrated GC.

Multi-dentate nitrogen-based ligands are less common than phosphine ligands; however, examples of palladacycles and free ligands used in the Heck coupling have been reported,^54–56^ thus bipyridine, phenanthroline, and terpyridine were investigated (Fig. 13). The results show inhibition curves for all three ligands, with bipyridine and phenanthroline completely inhibiting the catalytic activity at approx. 8.0 eq. Terpyridine inhibited the process as well, however, to a much lower degree, reaching approx. 20% yield of 9 at 12.5 eq. wrt Pd and 5 min residence time.

**Fig. 13:**
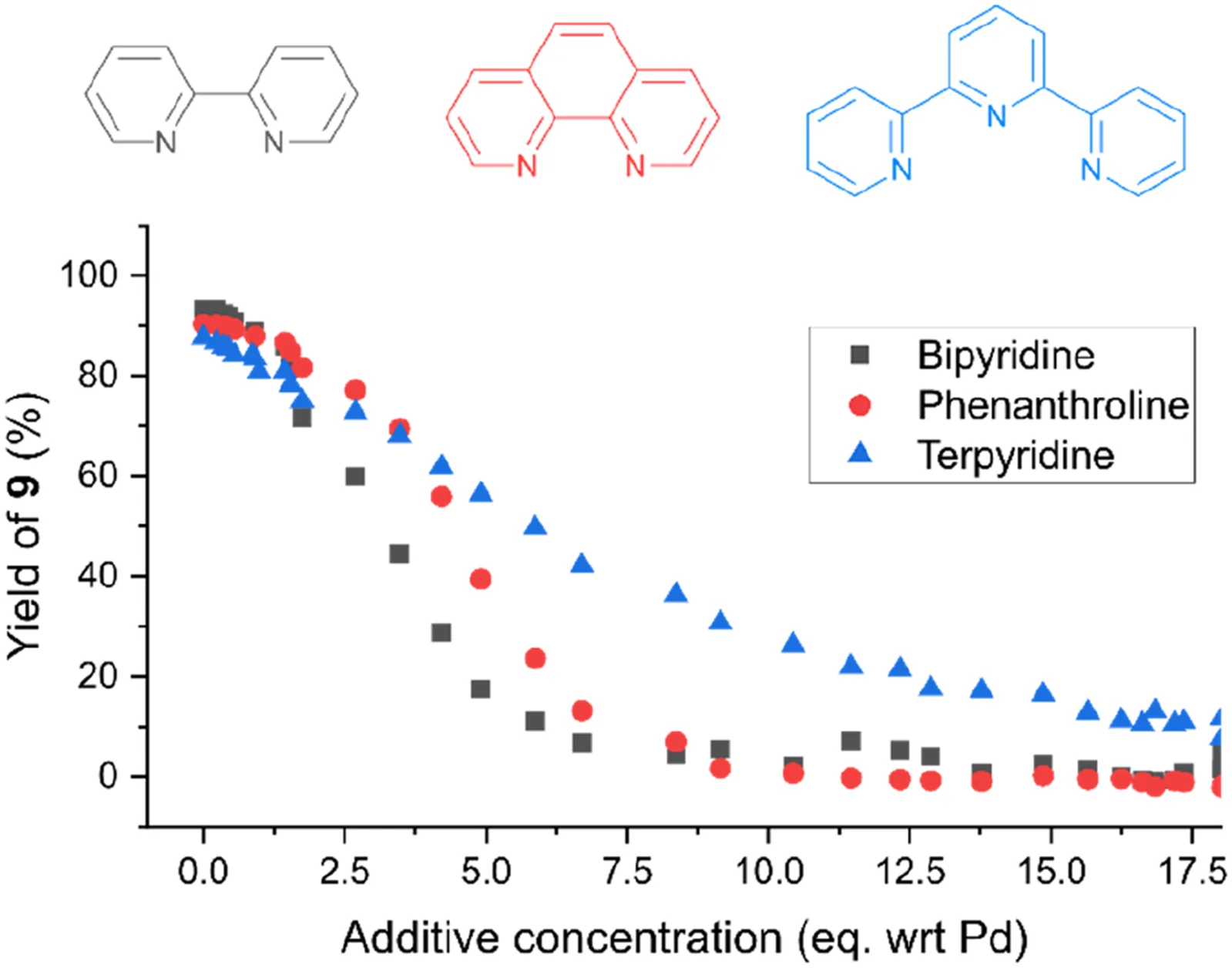
Yield of 9 (*t*_r_ = 5 min) in Heck coupling reaction with 0.225 mol% Pd catalyst utilising a concentration gradient of nitrogen-containing ligands. Yields were determined using calibrated GC.

#### Investigation into the effects of various additives and their concentrations on the Heck coupling reaction

One approach to predicting the functional group compatibility of a reaction is to carry it out in the presence of additives containing the groups to be queried.^57^ The same flow setup (Fig. 8) was used with the only change being that a solution of the additive was injected into the dispersion coil and then joined with the remaining reactants. Initially tested additives involved sulphur-containing organic molecules (Fig. 14), as these are known to act as poisons in metal-catalysed reactions^58–60^ and metal scavengers^61^ but are also used as ligands^62^ or chiral additives^63^ in various protocols. DMSO, thiophene, diphenyldisulfide, thioanisole, and 2-naphthalenethiol were investigated using the dispersion-generated concentration gradient method in the range between 0.0 and 1.0 eq. with respect to *n*-butyl acrylate 8 (thus 0–20 equiv. relative to the 5 mol% Pd catalyst). The results show a rough dependence on sulphur nucleophilicity with an almost immediate inhibition of the catalyst activity when thiol was added to the reaction mixture. Less nucleophilic diphenyldisulfide also inhibited the process, however, at a much slower rate, reaching full inhibition at 1.0 eq. of the disulfide. Addition of thioanisole did not affect the formation of 9 up to 0.6 eq., but the catalyst then started to rapidly lose its activity. DMSO and thiophene did not significantly affect the reaction even after reaching 1.0 eq. of the additive.

**Fig. 14:**
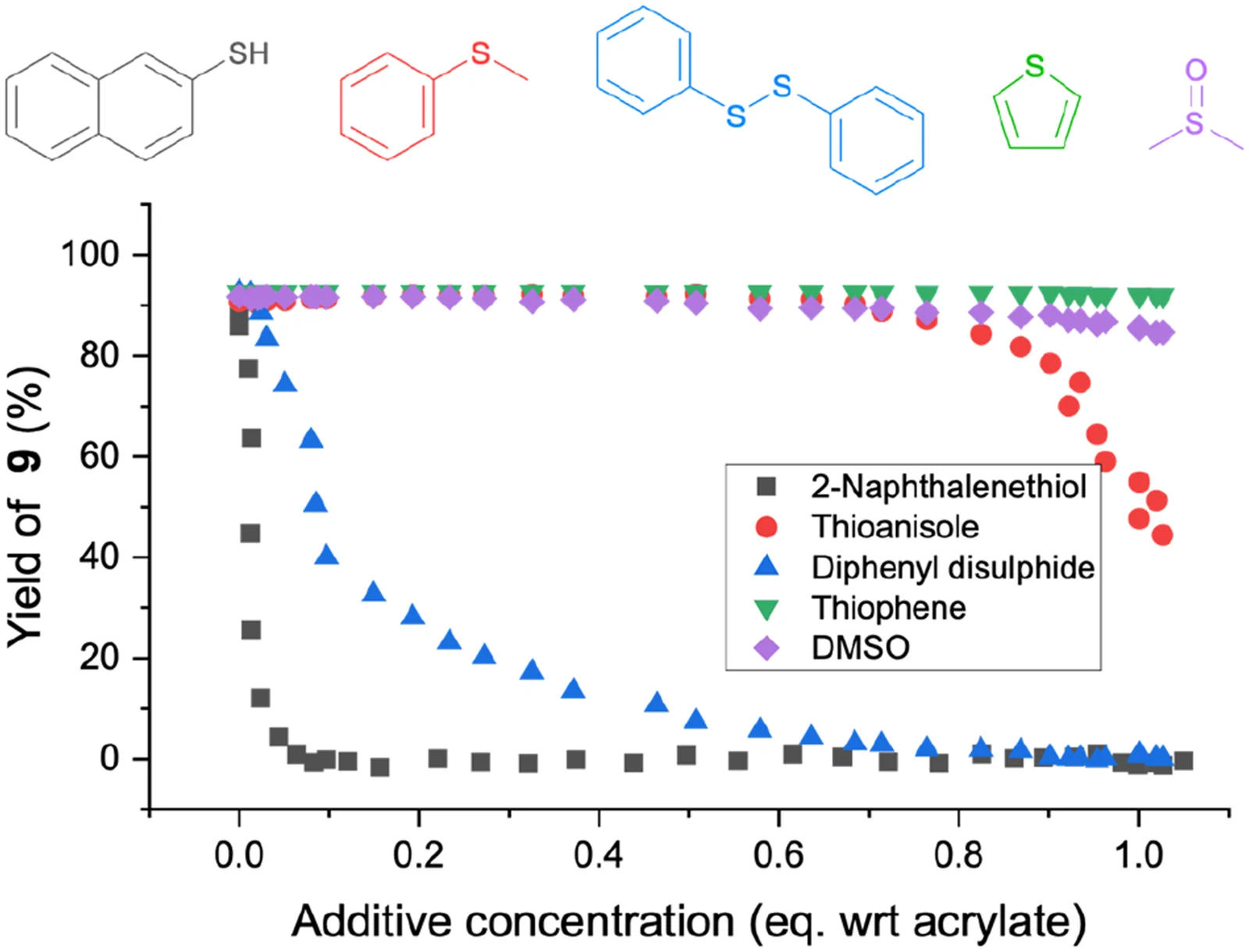
Sulphur-containing molecules screened in Heck coupling with 0.225 mol% Pd(OAc)_2_, and their effect on the yield of 9 obtained after 5 min residence time. Yields were determined using calibrated GC.

Heterocycles are present in the vast majority of active ingredients; thus, various heterocycles and the effects of their concentrations were investigated (Fig. 15). Furan, pyrrole, and MOXA (3-methyl-2-oxazolidinone) showed no process inhibition across the entire tested concentration range of up to 1.0 eq. wrt acrylate. Pyridine caused a linear decrease of the rate of formation of 9 down to 15% at 1.0 eq., whereas imidazole reached full inhibition at approx. 0.5 eq.; however, the curve produced was more sigmoidal. Addition of 2-amino-5-bromo-1,3,4-thiadiazole resulted in instant inhibition of the process, presumably due to the free amine group.

**Fig. 15:**
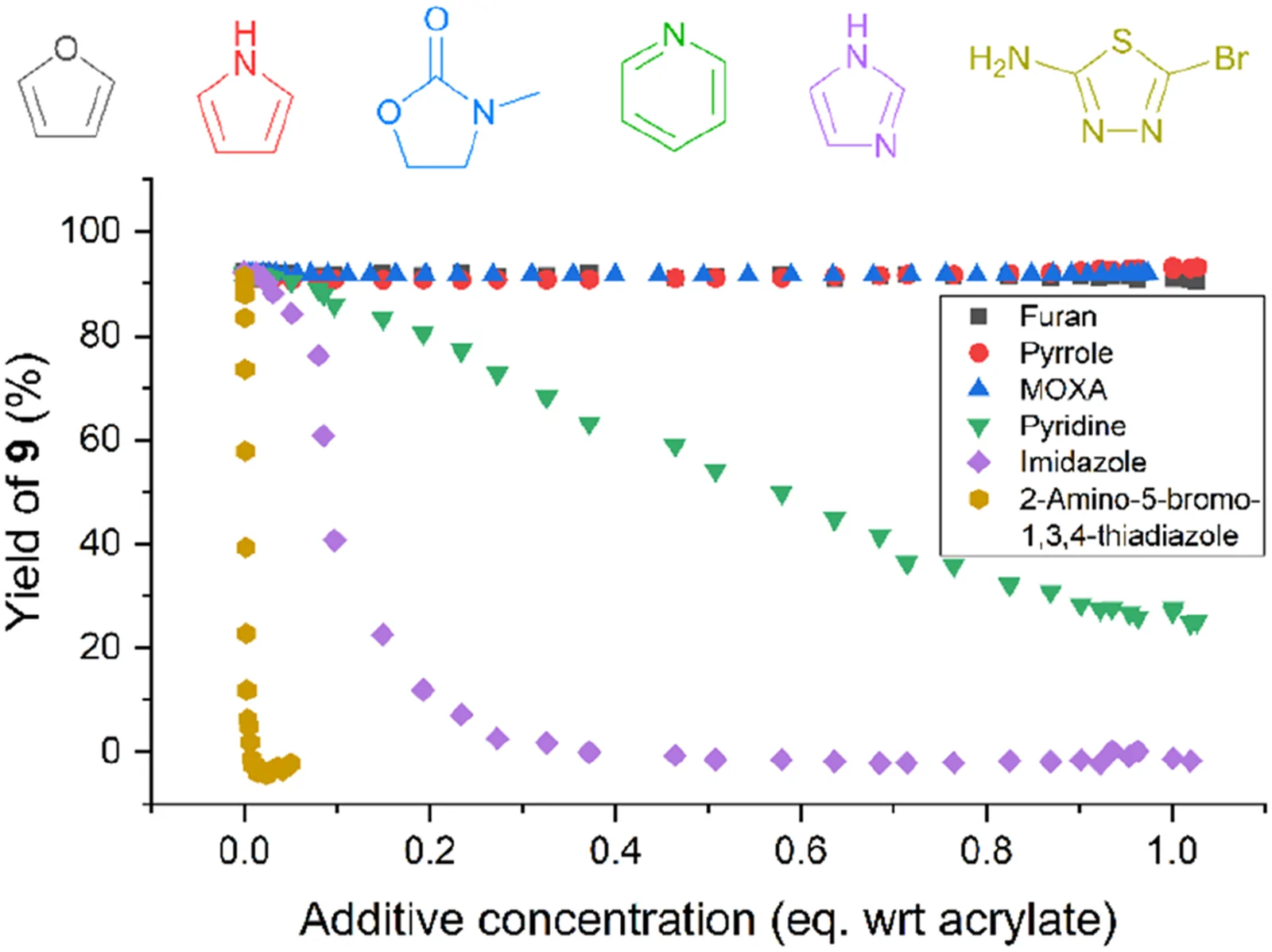
Heterocycles screened in Heck coupling and their effect on the yield of 9 at 5 min residence time. Yields were determined using calibrated GC.

Alkenes and alkynes are commonly encountered functional groups both in small organic molecule synthesis and in various active ingredients. Screening (Fig. 16) showed that 1-octyne and 1,5-cyclooctadiene both inhibit the Heck coupling of acrylate 8 with exponential decay-type curve. Norbornene on the other hand inhibits the coupling process with a sigmoidal-shaped curve reaching 14% yield of 9 at 0.75 eq. and 5 min residence time. 1-Hexene shows no effect on the process until 0.7 eq., after which a slow inhibition is observed, probably due to competition with the substrate. Additional experiments with the concentration gradient of 1-hexene reaching up to 3.0 and 10.0 eq. were performed showing that the Heck coupling reaction is further inhibited by excess amount of 1-hexene, reaching approx. 25% yield of 9 at 10.0 eq. of the additive and showing good overlay of all three experiments.

**Fig. 16:**
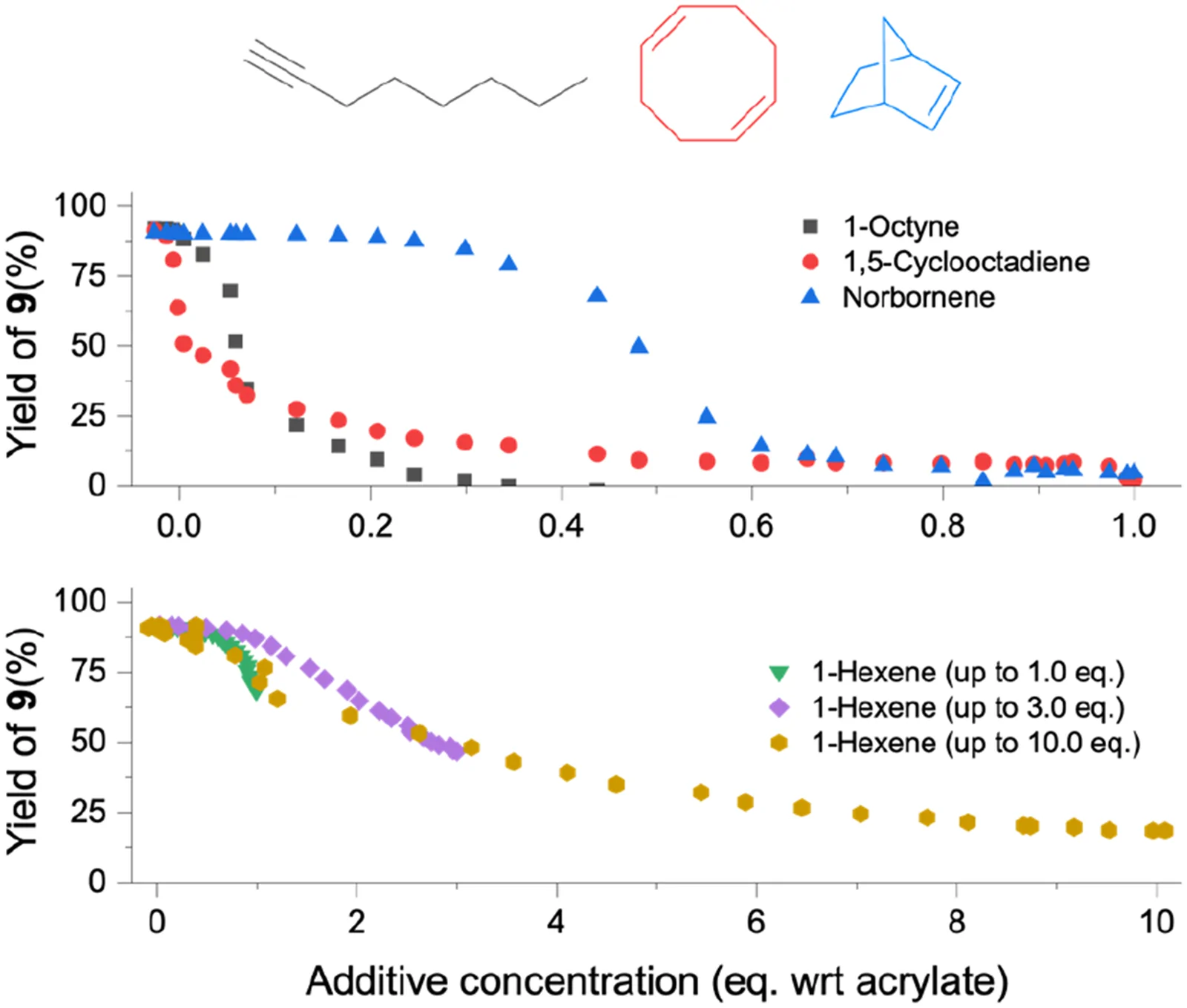
Alkenes and alkyne and their effect on the yield of 9 in Heck reaction. Yields were determined using calibrated GC.

### Optimisation of reactant stoichiometry and effects of additive concentrations on the Sonogashira coupling reaction

The Sonogashira reaction has become an important tool for synthetic chemists, allowing coupling of terminal alkynes with aryl or vinyl (pseudo)halides, resulting in the formation of a new C–C bond.^64–66^ A typical procedure for Sonogashira coupling involves both a Pd source and a copper cocatalyst, and by far the most used is the original procedure which requires 5–10% palladium catalyst.^67^ The inclusion of a Cu cocatalyst often causes undesired Glaser homocoupling of the alkyne. Several heterogeneous Cu-free Sonogashira reactions (also known as Heck alkynylations) have been reported;^68–71^ however, due to the possible Pd clustering and nanoparticle formation a homogeneously catalysed process was desired, just as in the case of Heck coupling.

We utilised the same flow setup as in herein reported Heck coupling studies (Fig. 8), with the only change being the use of larger 3.0 mL reactor. In-line IR was used to obtain dispersion profiles and in-line data, whereas sampling for off-line GC was used for determination of reaction outcome. We found that a Sonogashira procedure (Scheme 4) employing 0.5 mol% Pd(OAc)_2_ as a palladium source, 4 eq. of triphenylphosphine, 2 eq. of DBU as a base, and DMF using bromobenzene 10 and 1-octyne 11 as coupling partners with a flow reactor heated to 150 °C gave suitable conversion of 46% after 10 min residence time and no formation of palladium black, allowing for further studies to probe the effect of ligand and ligand concentration on the rate of the Sonogashira reaction.

**Scheme 4:**
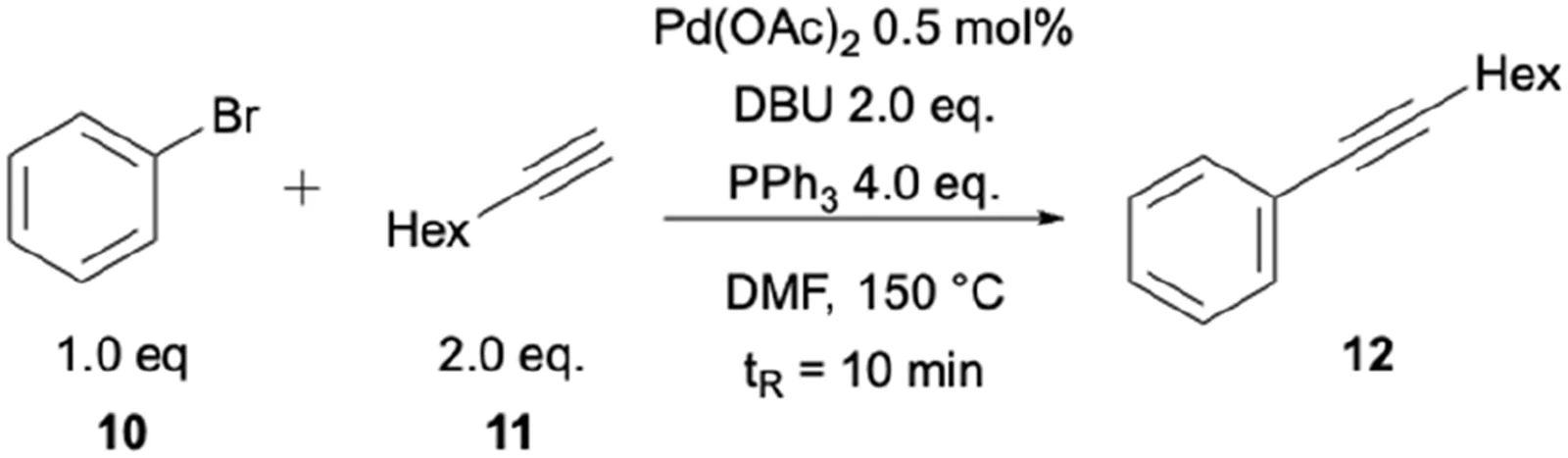
Sonogashira coupling between bromobenzene 10 and 1-octyne 11.

#### Screening of phosphine- and nitrogen-containing ligands and their concentration effects in Sonogashira coupling reactions

Initial optimisation of phosphine concentrations in the Sonogashira coupling reaction was performed with triphenylphosphine concentrations of up to 16.0 eq., as the usual loading is <4.0 eq. with respect to the palladium. The result showed monotonic increase in yield of 12 at 10 min residence time; thus, a second experiment with concentrations of up to 160.0 eq. (Fig. 17, red dots) was performed. Surprisingly, optimal loading of PPh_3_ was found to be at approx. 47.0 eq. with respect to the palladium, which is far in excess of the traditional amounts. The dramatic difference from the Heck reaction where phosphine ligands strongly inhibit the rate of reaction is notable. Further screening of phosphines showed that P(*p*-tolyl)_3_ gives slightly faster conversion to 12 at lower equivalency when compared to PPh_3_, whereas addition of P(*o*-tolyl)_3_ resulted in only a slight increase of yield of 12 to approx. 10% at 10 min reaction time. Methyl- and ethyl diphenylphosphines both gave lower yields at 10 min than PPh_3_ with the latter generating the higher yield of the two, suggesting the positive influence of bulk and increased electron density.

**Fig. 17:**
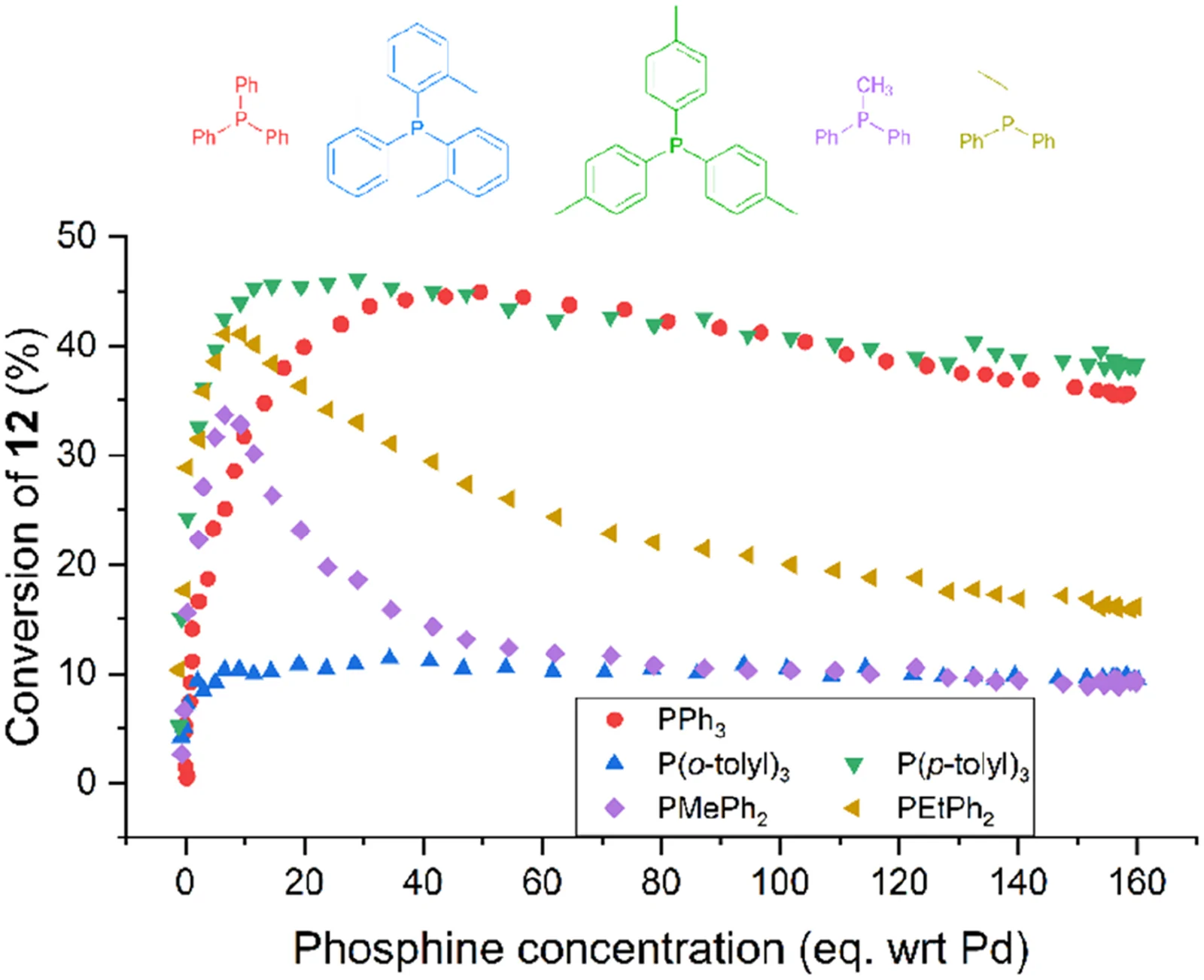
Screening of phosphine effects on the yield of 12 at 10 min residence time in Sonogashira coupling. Yields were determined using calibrated GC.

Heterocycle-containing phosphines were also screened (Fig. 18), as it is possible to dramatically change the nature of the ligand, *e.g.* exchanging phenyl groups for 2-furyl groups decreases σ-bond donating capability^72^ and increases the π-accepting ability of the phosphine. Investigation of bis(2-furyl)phenylphosphine and tri(2-furyl)phosphine showed slightly slower conversion when compared to PPh_3_; however, the optimal amount of phosphine was much higher. Exchanging a phenyl group for a 2-pyridyl group affects the sterics and allows for the ligand to act as both a mono- and a bidentate ligand; thus diphenyl-2-pyridyl phosphine was investigated and showed overall higher yield of 12 equal to 44% at approx. 72.5 eq. and 10 min residence time.

**Fig. 18:**
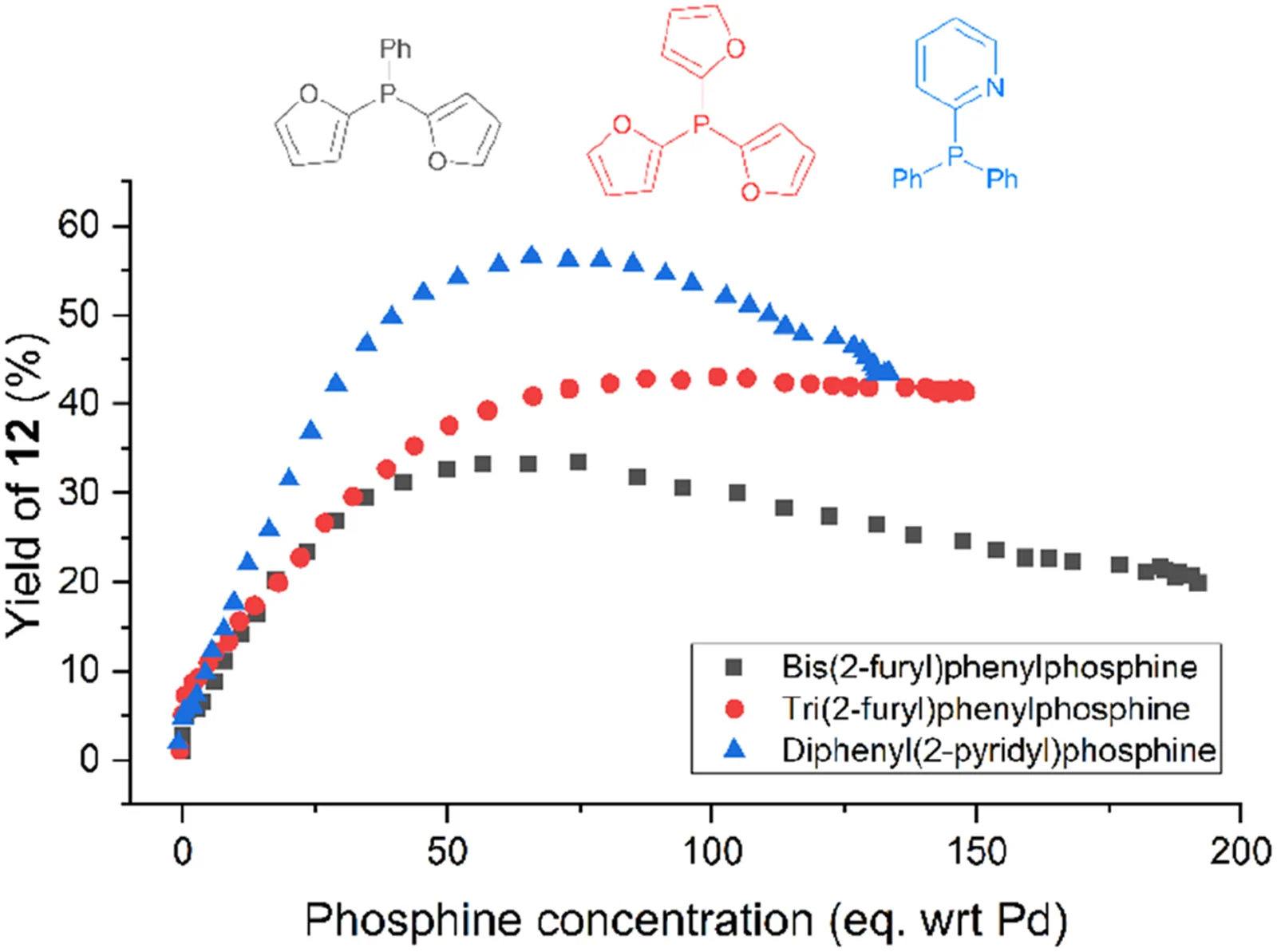
Effects of heterocycle containing phosphine ligand on the yield of 12 at 10 min residence time in Sonogashira coupling reaction. Yields were determined using calibrated GC.

Several bidentate phosphines were investigated (Fig. 19), and as expected, the optimum equivalency for these ligands is much lower than that of monodentate phosphines, reaching optimum equivalencies of dppm, dppe, and dppb at approx. 2.7 eq., and dppp at 4.0 eq. Interestingly, xantphos reached an optimal rate of formation of 12 at 14.0 eq. with respect to Pd, suggesting that it may act more like a monodentate ligand.

**Fig. 19:**
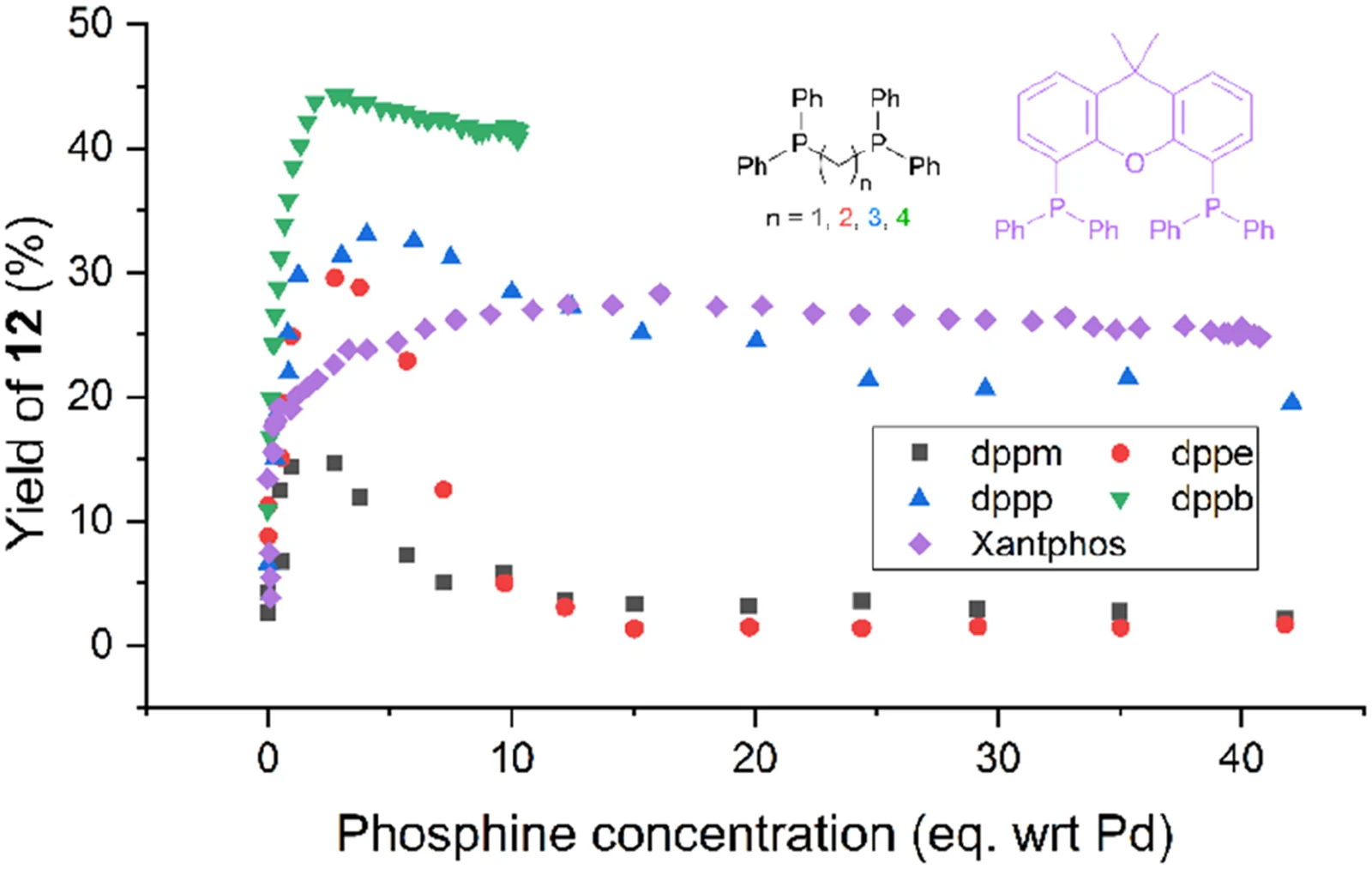
Effect of bidentate phosphines on the yield of 12 (*t*_r_ = 10 min). Yields were determined using calibrated GC.

Although intended only as an illustration of the concentration-gradient method the studies indicate that by using copper-free conditions with DBU as the base and high equivalents of mondentate or modest equivalents of bidentate phosphine ligands, the Sonogashira coupling can be carried out with low amounts of palladium catalyst, a method which deserves further study.

### Multiple time points concentration gradients

As powerful as the methodology for the creation of concentration gradients *via* dispersion phenomena is, it still has a limitation of being applicable to only a single reaction time point, due to the nature of flow systems which operate in the spatial domain rather than the time domain of batch chemistry. To overcome this, the flow setup was modified and equipped with a dedicated switching valve to allow the reaction mixture plug to be flowed through the flow reactor and detector in a closed loop, *aka* cyclic flow. Two modes of such motion are possible, namely reciprocating and unidirectional (or non-reciprocating) flow. Such methodology has been previously reported for use in small-scale flow investigations of reaction kinetics under steady-state conditions,^73^ affording time series data from a single flow experiment; however, to the best of our knowledge no previous report has described the use of transient flow methods with cyclic flow.

#### Reciprocating cyclic flow

Initially the investigation of multi-time-point methodology was performed using a reciprocating flow setup (Fig. 20) and a model S_N_Ar reaction between 1-fluoro-4-nitrobenzene 13 and pyrrolidine 14, affording 1-(4-nitrophenyl)pyrrolidine 15 (Scheme 5) which could be easily monitored using in-line UV-vis spectroscopy.

**Fig. 20:**
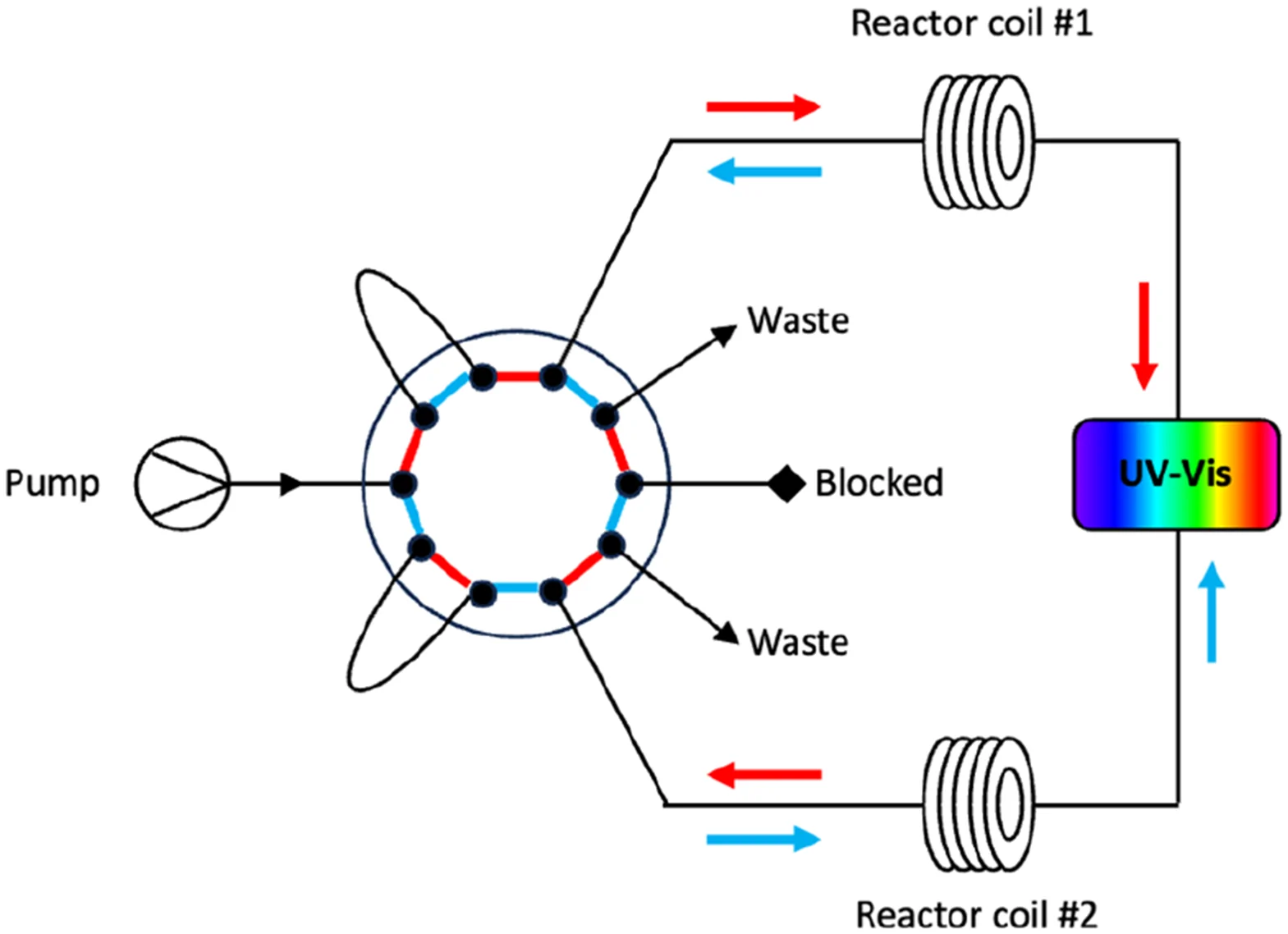
Flow setup used for reciprocating flow.

**Scheme 5:**
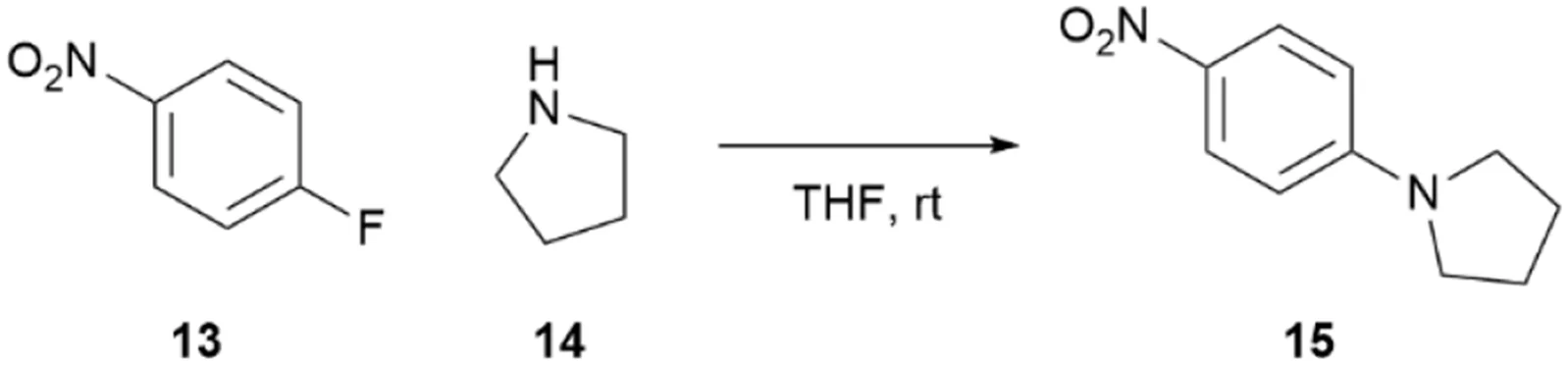
S_N_Ar reaction between 1-fluoro-4-nitrobenzene 13 and pyrrolidine 14, affording 1-(4-nitrophenyl)pyrrolidine 15.

The dispersion profile of a 5.0 mL reaction plug was measured over 2 hours of reciprocating flow to determine the timing of valve switching and to check whether the reaction steady state is unaffected (or affected negligibly) by the dispersion averaging effect. Afterwards the S_N_Ar reaction was employed in the same setup and the dependence of the yield of 15 on residence time was extracted (Fig. 21, black dots).

**Fig. 21:**
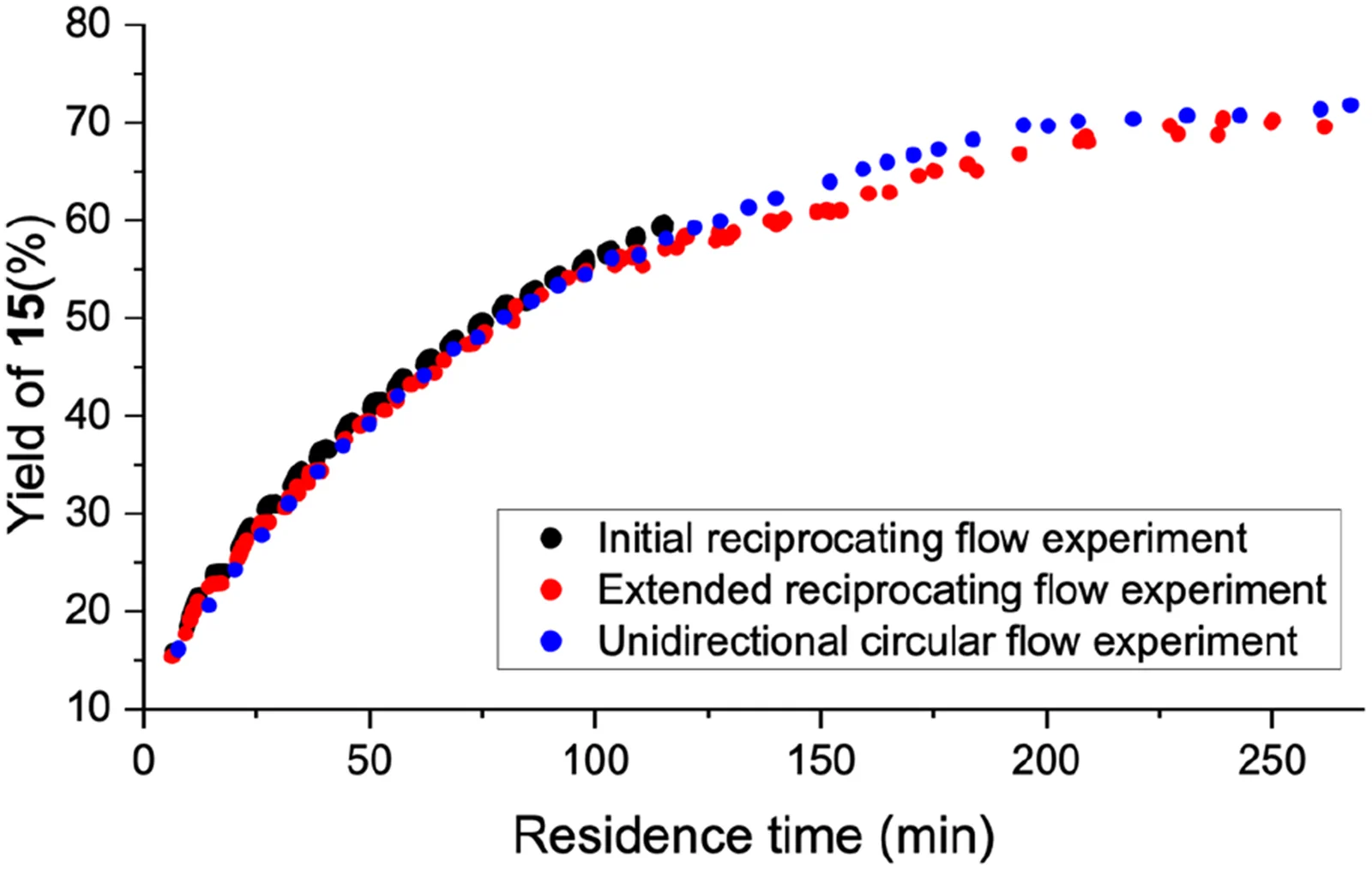
Yield of 15 obtained from calibrated in-line UV-vis monitoring of cyclic flow experiments.

It is important to notice when using reciprocating flow method that when the reaction plug passes for the first, or any odd numbered, time through the detector, the entire plug has spent the same amount of time in the reactor. However, upon reversal of the flow direction, the front, according to the flow direction, of the reaction plug will have had only one reactor worth of reaction time, whereas the end of the reaction plug will have experienced two reactor residence times (Fig. 22).

**Fig. 22:**
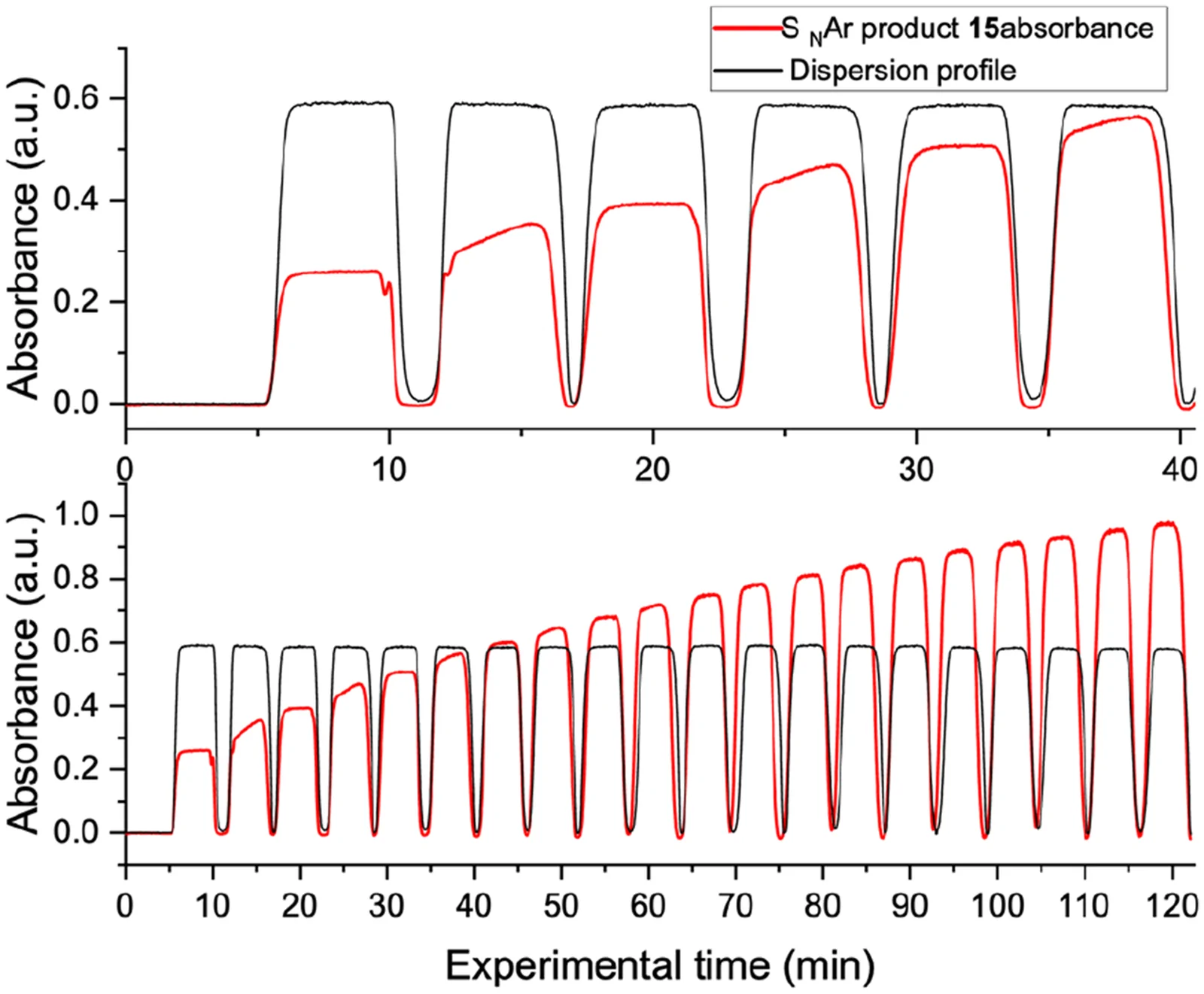
In-line UV-vis absorbance of 15 (red) obtained from an initial reciprocating flow S_N_Ar experiment and flow system dispersion profile (black).

The experiment was then repeated with valves switching over 270 min of experimental time, allowing observation of reaction outcome over longer residence times, and showed excellent agreement with the shorter experiment (Fig. 21, red dots).

#### Unidirectional (non-reciprocating) cyclic flow

To avoid the residence time variation that originates from reversing the flow direction a closed-loop unidirectional flow was pursued. To accomplish this, the flow setup was equipped with 6-port and 10-port valves (both 2-position) (Fig. 23).

**Fig. 23:**
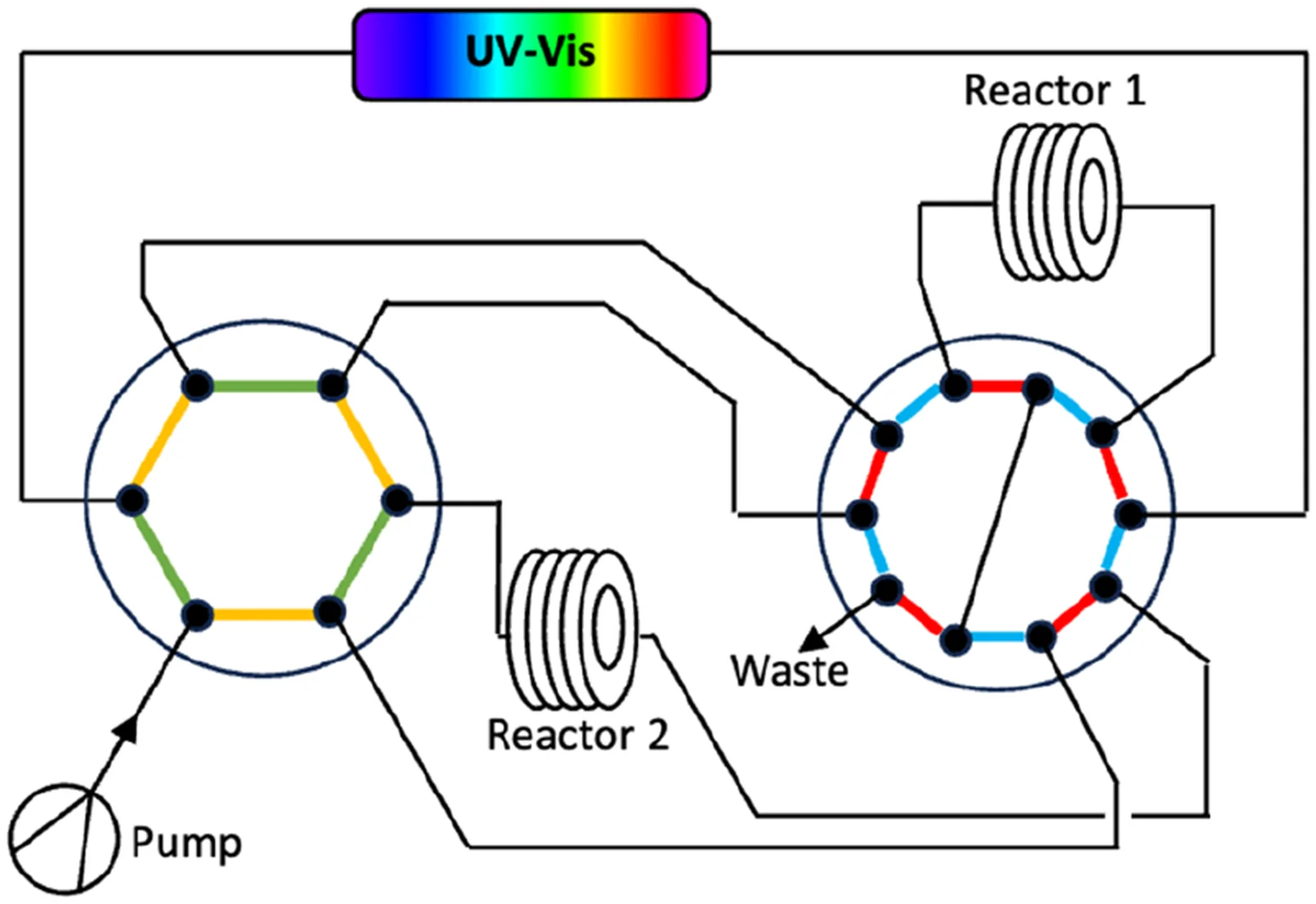
Flow setup used in unidirectional flow system.

In this setup the reaction plug enters the 6-port valve and is directed to the detector and then the first flow reactor, effectively generating the *t*_R_ = 0 time point, after which both valves are switched, and the plug is again directed to the detector and the second flow reactor. The 10-port valve is then switched again, and the reaction plug is directed through the detector and into the first flow reactor. Switching of the 10-port valve at selected times allows for the reaction mixture to be circulated between the reactor and the detector until the operator ends the experiment or until the dispersion has spread the reaction plug to an unusable level.

The same model S_N_Ar reaction between 13 and 14 was then employed in the unidirectional flow setup. The UV-vis monitoring (Fig. 24) shows consistent residence times throughout the reaction plug all having flat steady states. Analysis of the spectral data shows excellent agreement with the previous experiments, giving approx. 70% yield of 15 at 250 min residence time (Fig. 21, blue points).

**Fig. 24:**
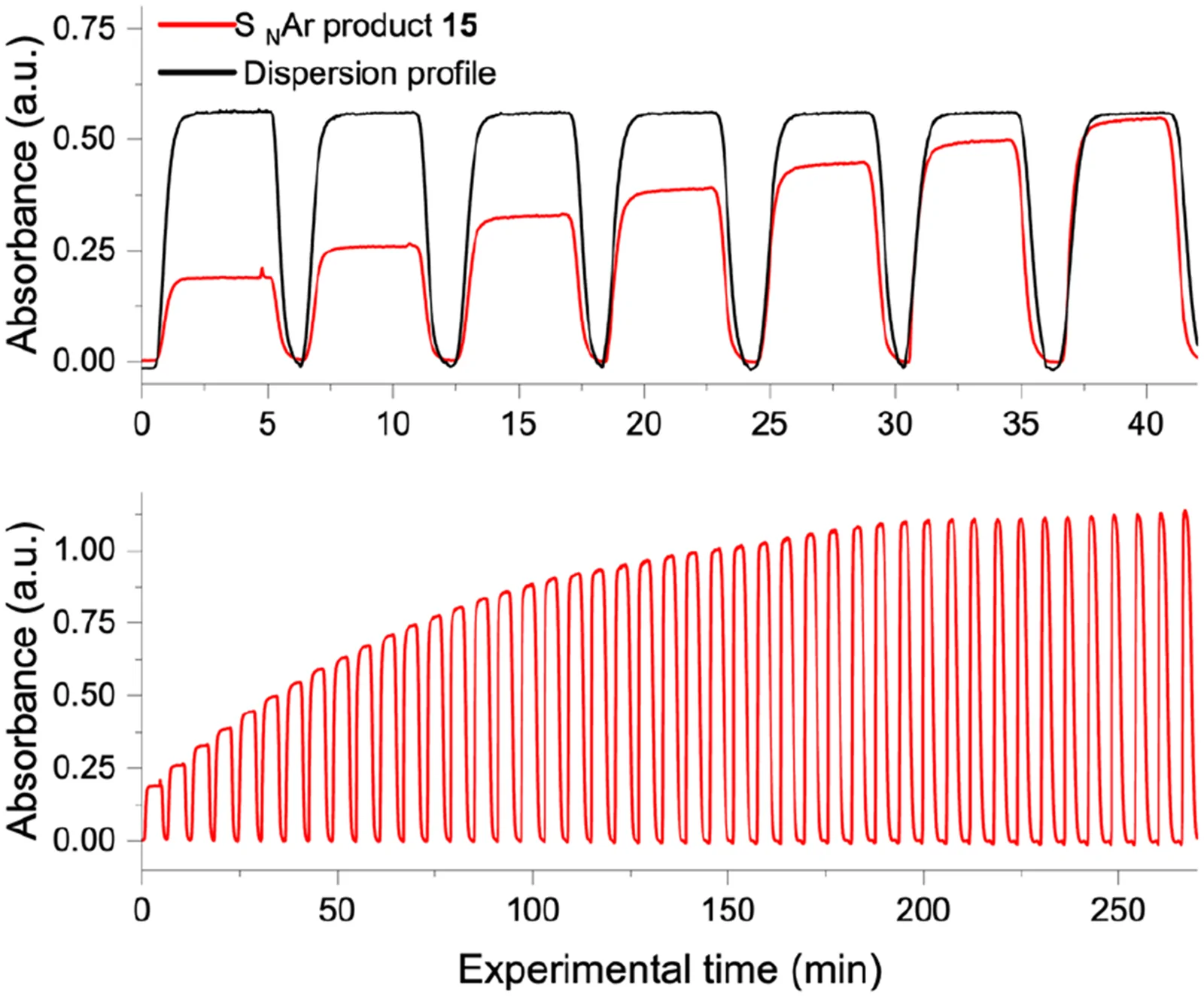
In-line UV-vis absorbance of 15 (red) in a unidirectional cyclic flow and flow system dispersion profile (black).

#### Unidirectional cyclic flow with a single concentration gradient

To reduce the size of the plug that must be circulated, clipping was used to extract only the concentration gradient from one end of the initial dispersed plug before combining it with the remaining reaction components. The flow setup was modified accordingly (Fig. 25) with an additional 6-port 2-position valve allowing injection of only the front end of the concentration gradient. Moreover, in an effort to reduce dispersion, the flow reactors were filled with glass beads (Fig. 26), enhancing cross-tube mixing and thereby reducing dispersion, allowing longer operation times (see the SI for details).

**Fig. 25:**
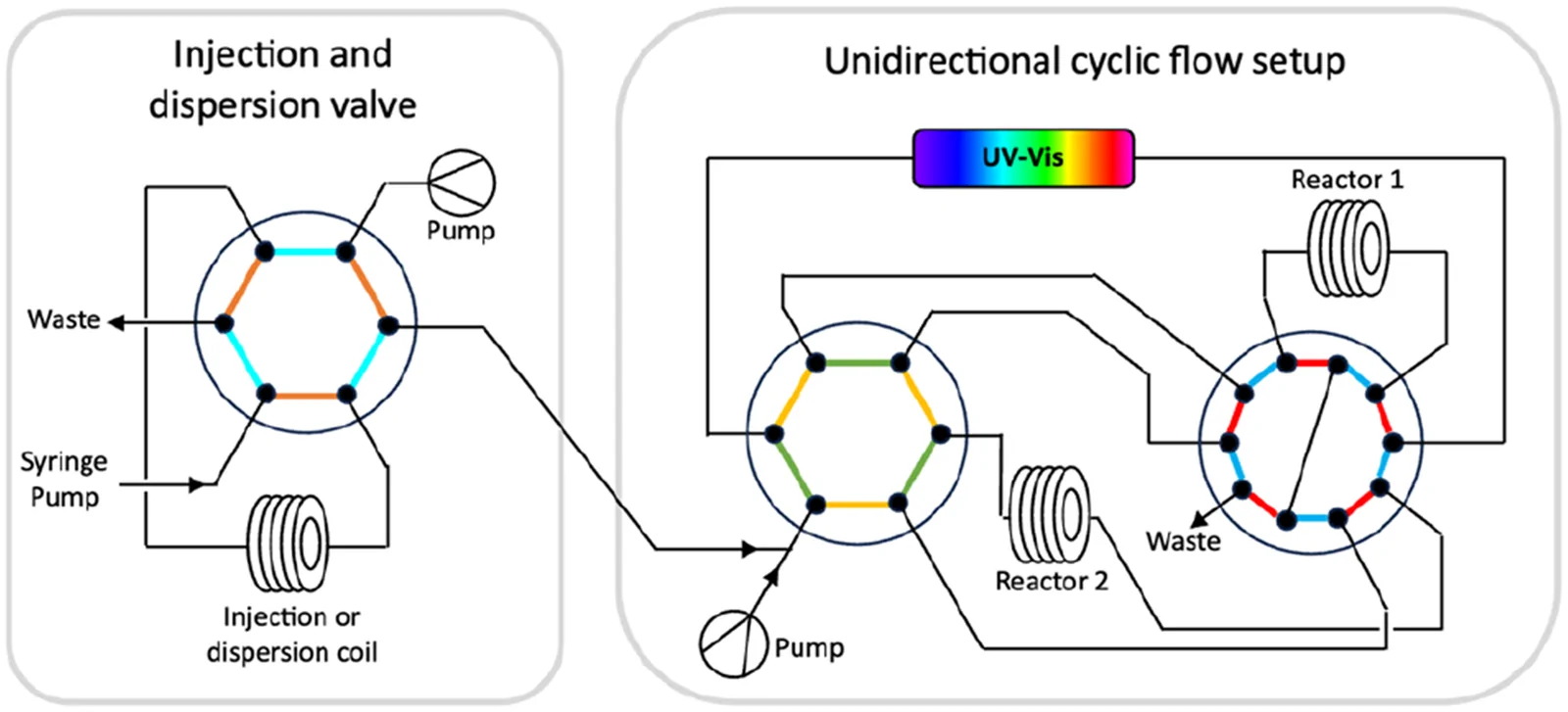
Flow setup for gradient clipping and unidirectional cyclic flow system.

**Fig. 26:**
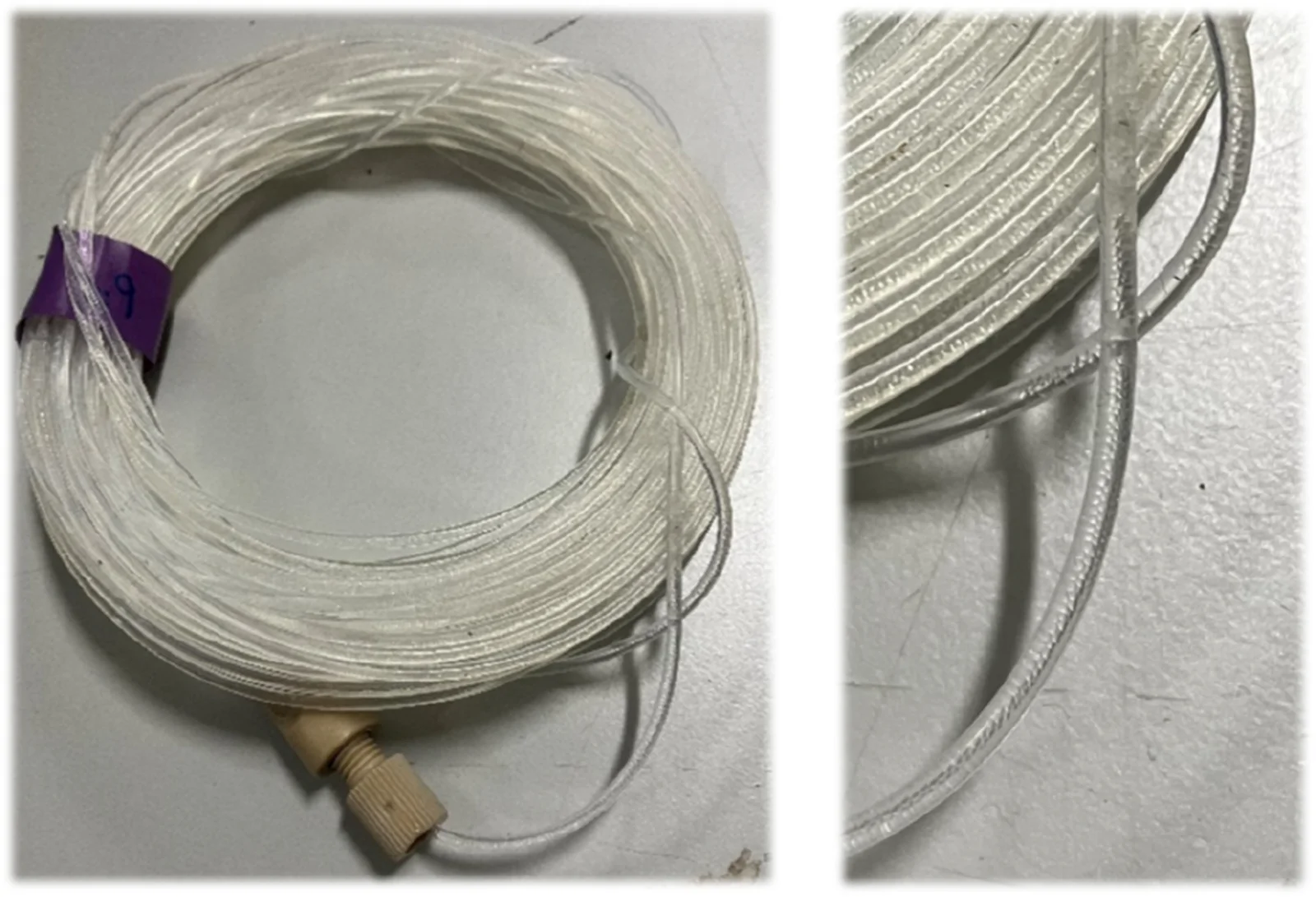
Left: Tubular flow reactor (1.0 mm ID, 1.6 mm OD, PFA) packed with glass beads (0.5 mm diameter). Right: Zoom into tubing filled with glass beads.

The same model S_N_Ar reaction between 13 and 14 was then used with a clipped concentration gradient of pyrrolidine 14 up to a maximum of 15.0 equivalents. A Vapourtec flow system and switching valves control were used as well as post-reaction data analysis was performed with custom Python script (see the SI for more details). Although the results are unsurprising, showing that yield of 15 increases with both time and concentration of 14 (Fig. 27 and 28), the large amount of data generated demonstrates the potential of the method.

**Fig. 27:**
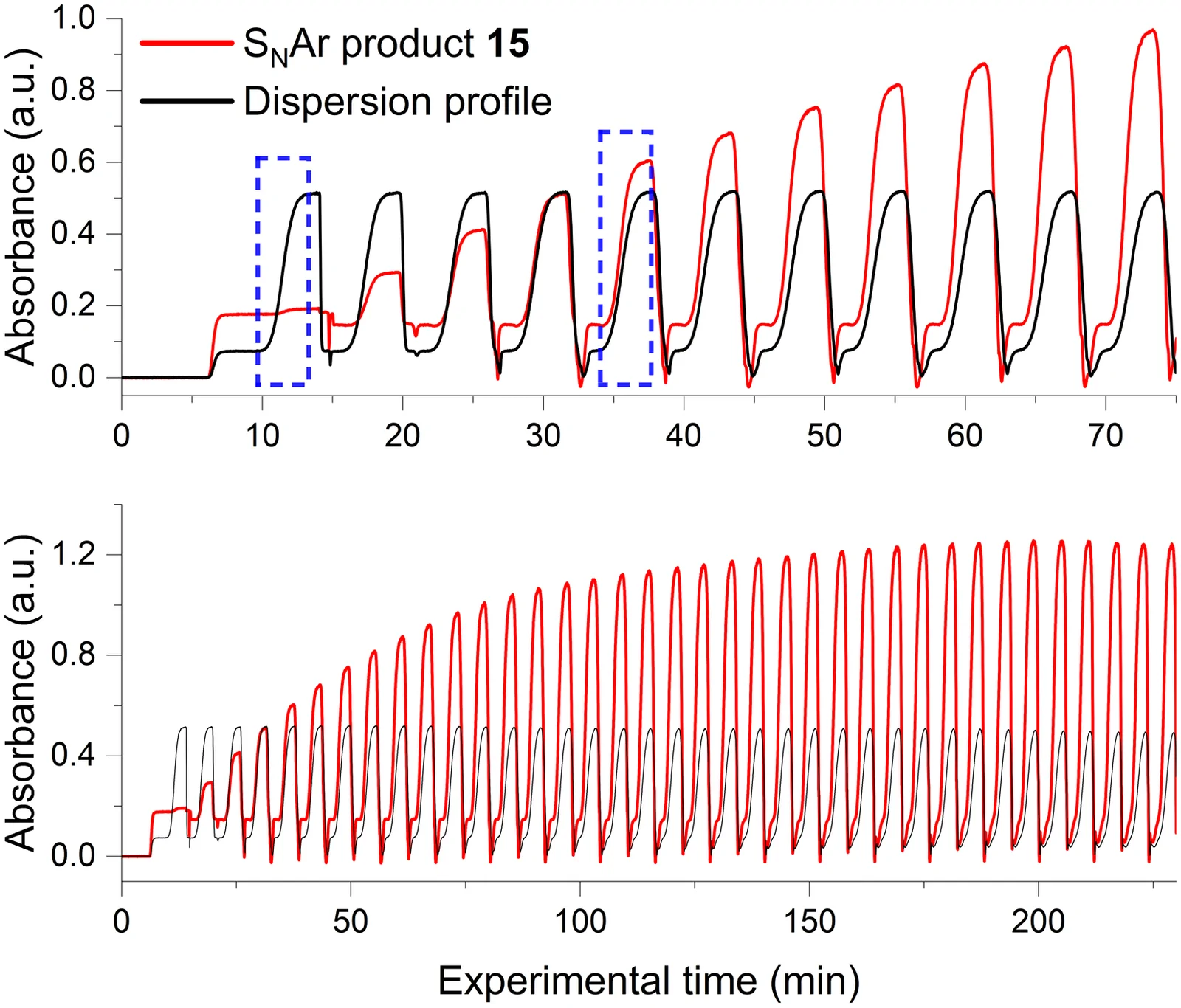
In-line UV-vis absorbance of 15 with gradient of 14 injected into unidirectional flow system. Selected concentration gradient regions are shown in blue dashed rectangles.

**Fig. 28:**
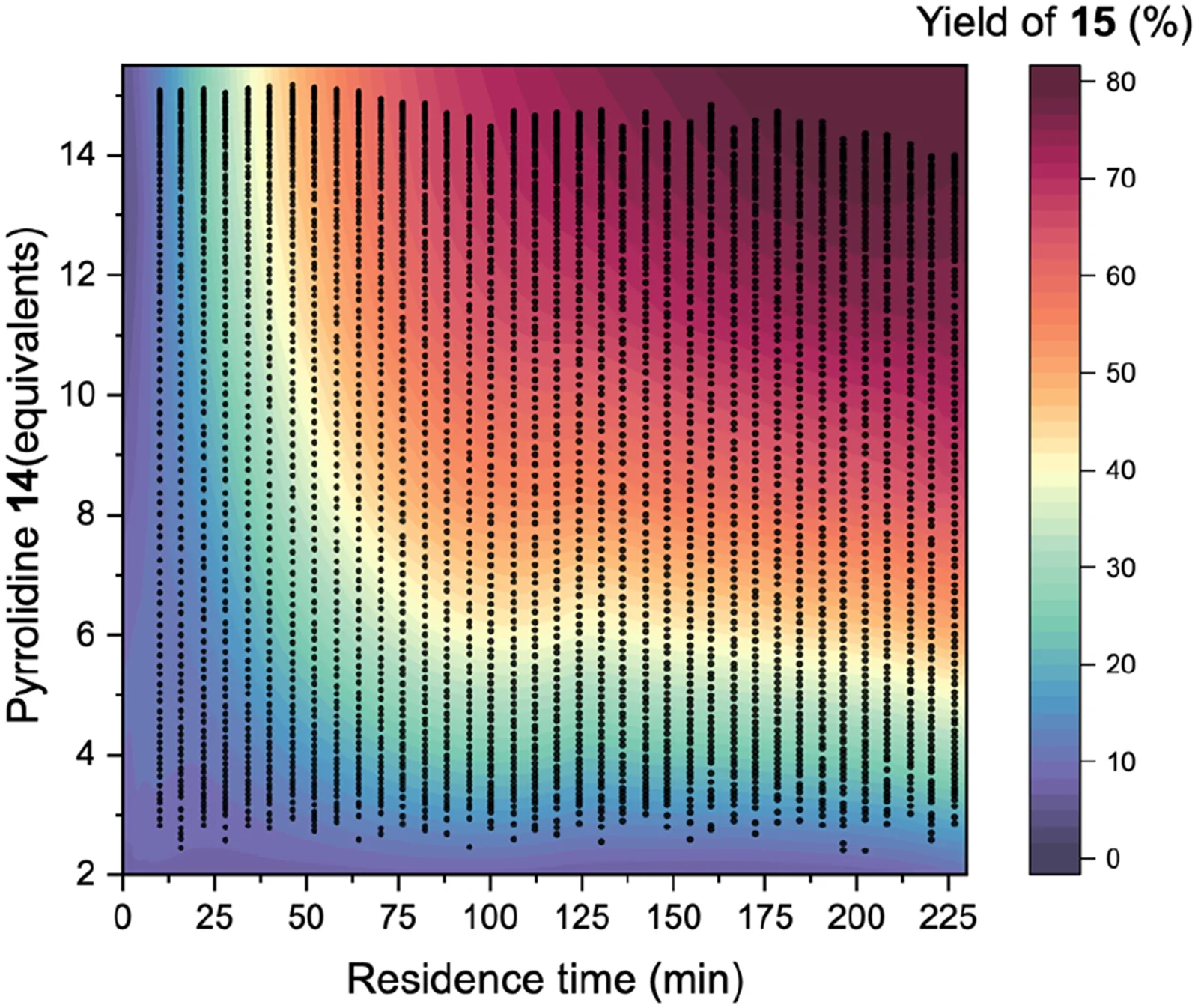
Contour plot of yield of 15*vs.* residence time and pyrrolidine 14 concentration obtained from calibrated in-line UV-vis monitoring. Black dots indicate data points obtained from gradient areas of the unidirectional flow experiment.

#### Unidirectional cyclic flow with multiple concentration gradients

To increase the utility of the methodology and extract even more process information, we looked to accommodate several concentration gradients in a single cyclic flow experiment. To enable this the volume of the clipped gradient was reduced by shortening the dispersion coil and decreasing the volume of the injected dispersed reagent plug.

The flow setup was modified by incorporating a low-volume manual injection sample loop and a low-volume dispersion coil connected to the clipping valve (Fig. 29). The sample loop is initially loaded with the first reactant and then switched to introduce it into the dispersion coil. The dispersion of the first reactant occurs while the sample loop is simultaneously refilled with the second reactant. The sample loop valve is subsequently switched to introduce the second reactant into the dispersion coil attached to the clipping valve. The clipping valve is timed such that the plug containing the first reactant exits the valve only after the plug containing the second reactant has completely entered the dispersion coil. This sequence can be repeated as required. The setup provides straightforward control over the extent of dispersion by varying either the flow rate or the size of the dispersion coil. In addition, the separation volume between the first and the second reactant plugs can be controlled by adjusting the flow rate of the pump connected to the clipping valve.

**Fig. 29:**
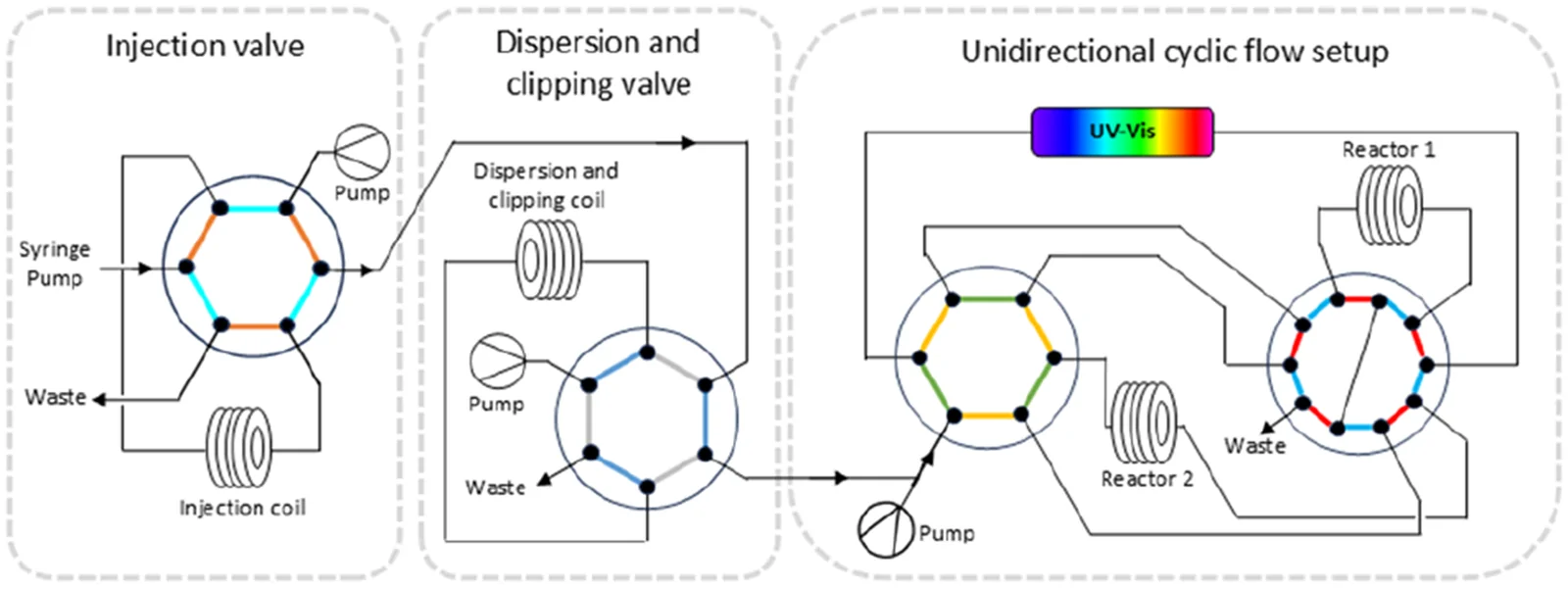
Flow setup used for injection of multiple reactants undergoing further dispersion, gradient clipping, and injection into unidirectional flow system.

The effect of amine concentration on the S_N_Ar reaction of 13 was studied with three different amines (pyrrolidine 14, piperidine 16, and azepane 17) (Scheme 6) in one experiment using the above-mentioned flow setup, allowing us to inject three subsequent clipped concentration gradients of 14, 15, and 16 into continuous stream of 13. Dispersion profile UV-vis data (Fig. 30, black line) show good steady-state retention; however, the plugs of dispersed reagents start to overlap after approx. 40 min of experimental time. Analysis of the UV-vis data (Fig. 31–33) shows the expected increase in yields with increasing residence time and amine concentration, and decrease in reactivity as the amine ring size increases, which matches the trend in decreasing nucleophilic parameters of the amines 14, 16, and 17, respectively.

**Scheme 6:**
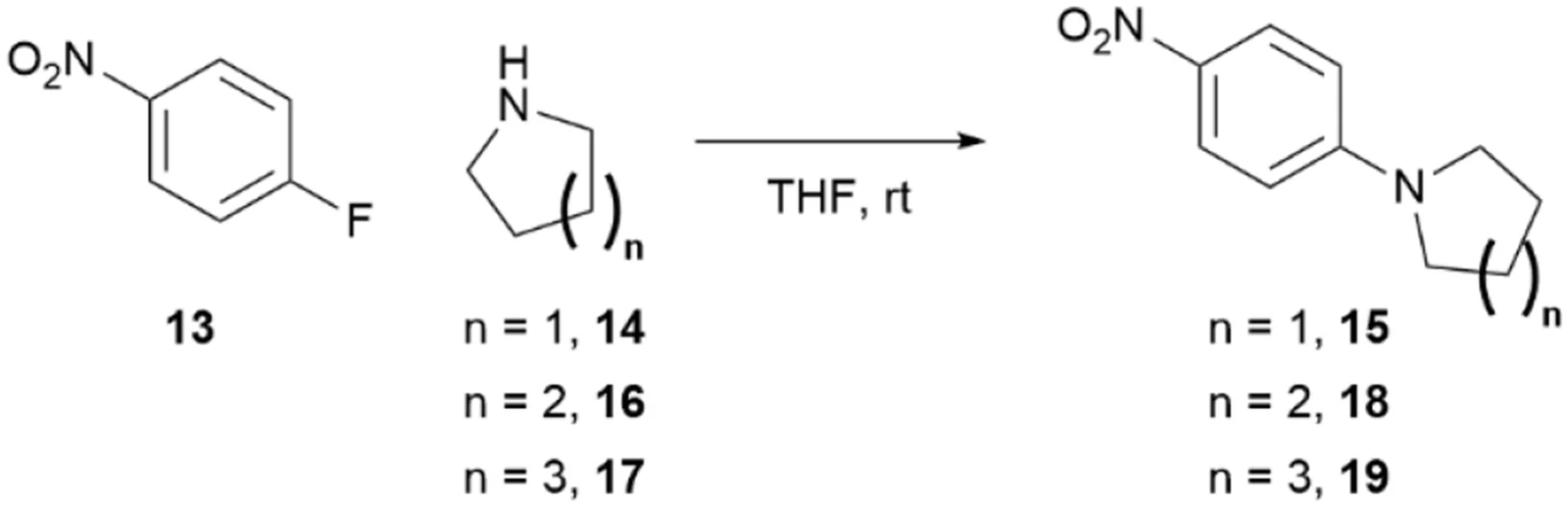
S_N_Ar reaction of 1-fluoro-4-nitrobenzene 13 with various cyclic amines used in the multiple gradient multiple timepoint unidirectional flow system.

**Fig. 30:**
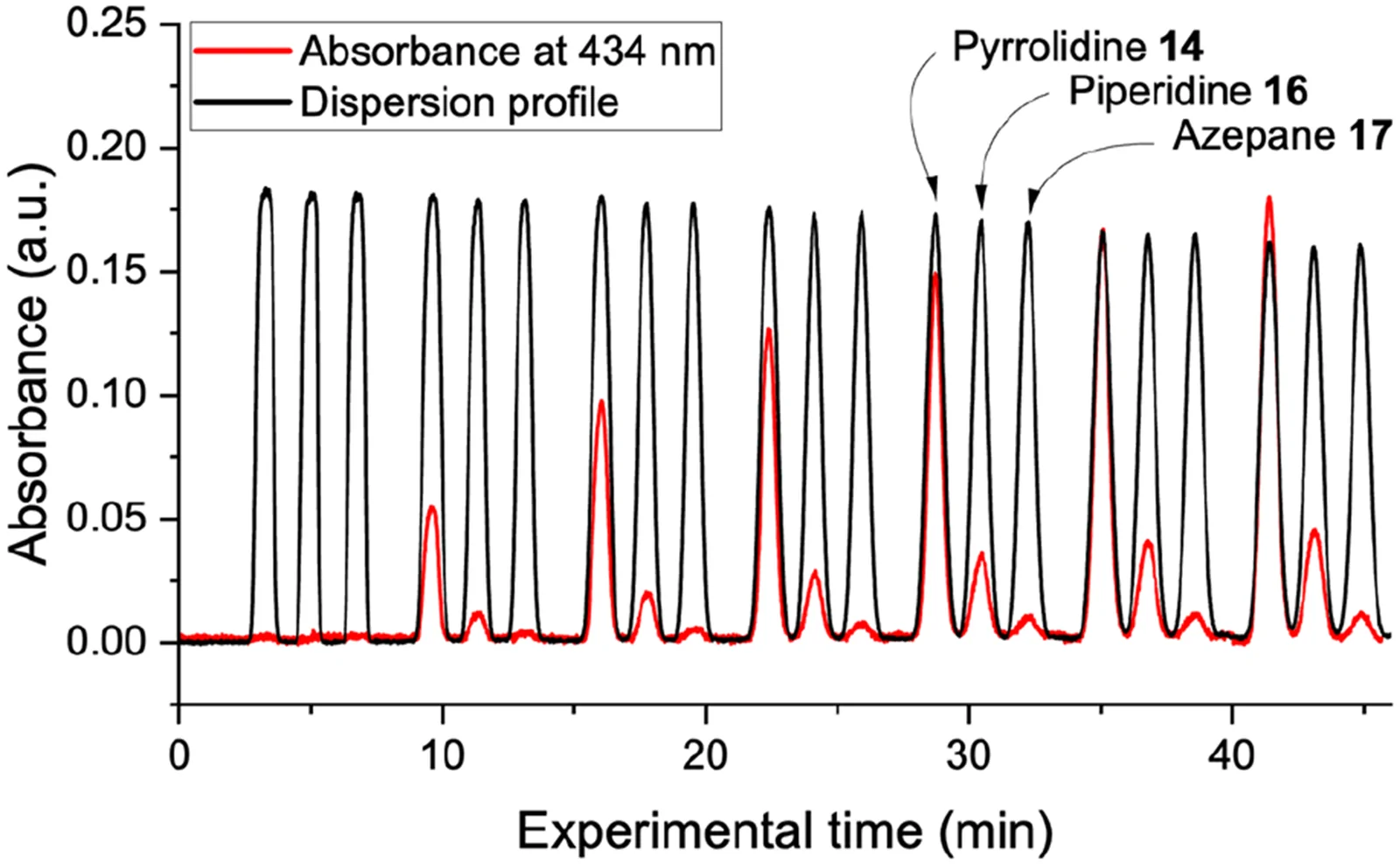
In-line UV-vis absorbance of S_N_Ar reaction products (15, 18, and 19) (red) and dispersion profile of the flow system (black).

**Fig. 31:**
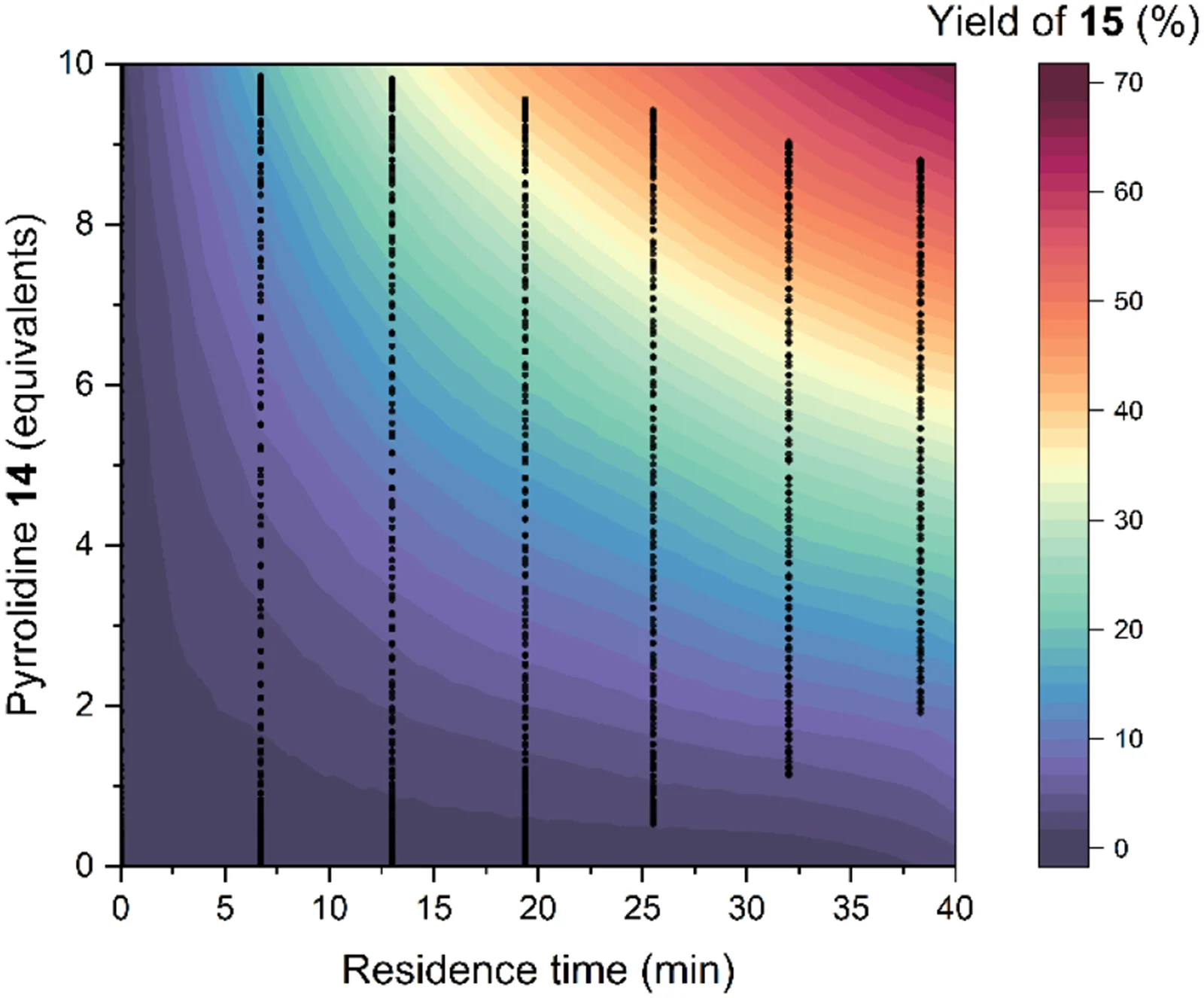
Yield of 15*vs.* [14] and residence time obtained from calibrated in-line UV-vis monitoring.

**Fig. 32:**
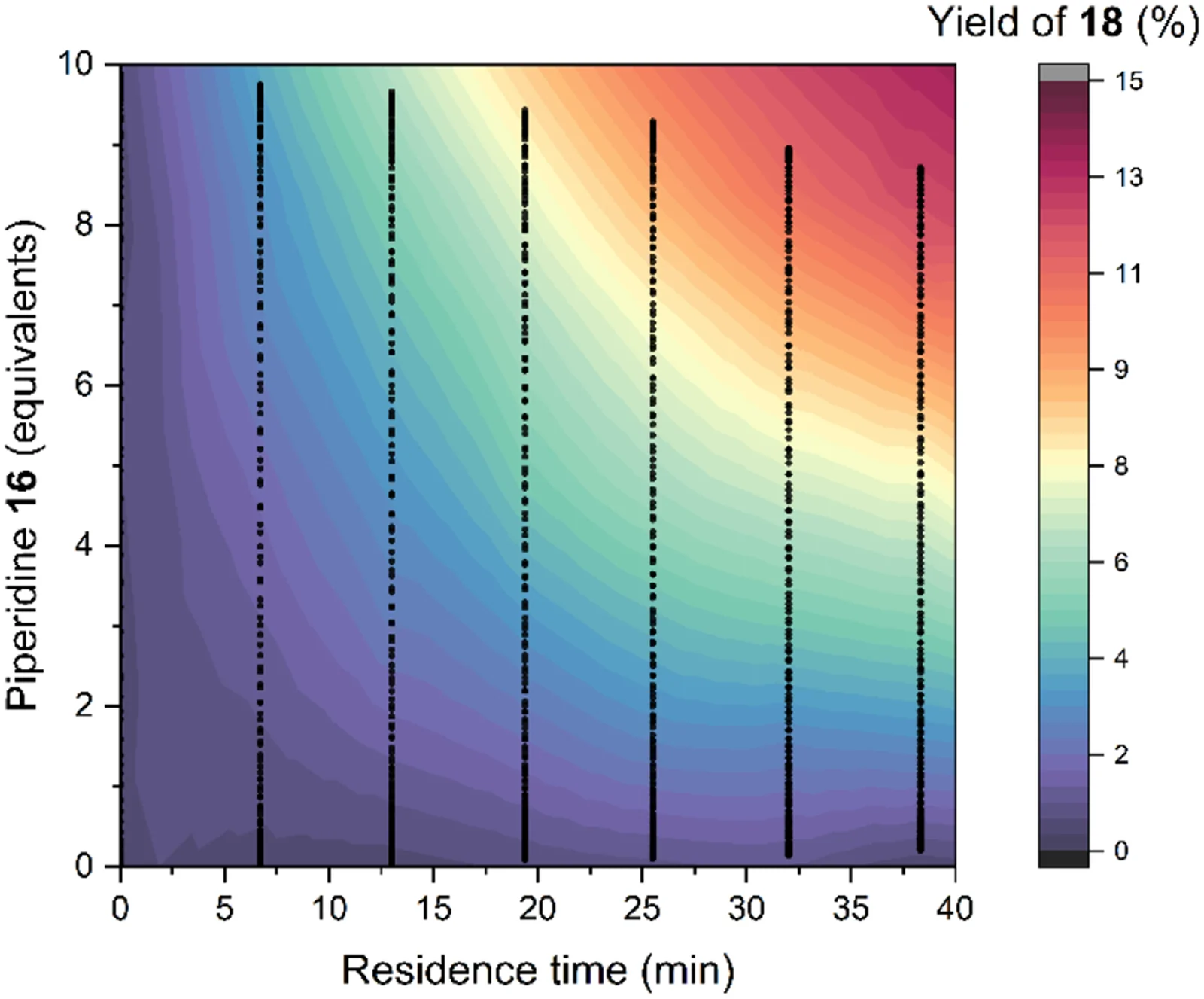
Yield of 18*vs.* [16] and residence time obtained from calibrated in-line UV-vis monitoring.

**Fig. 33:**
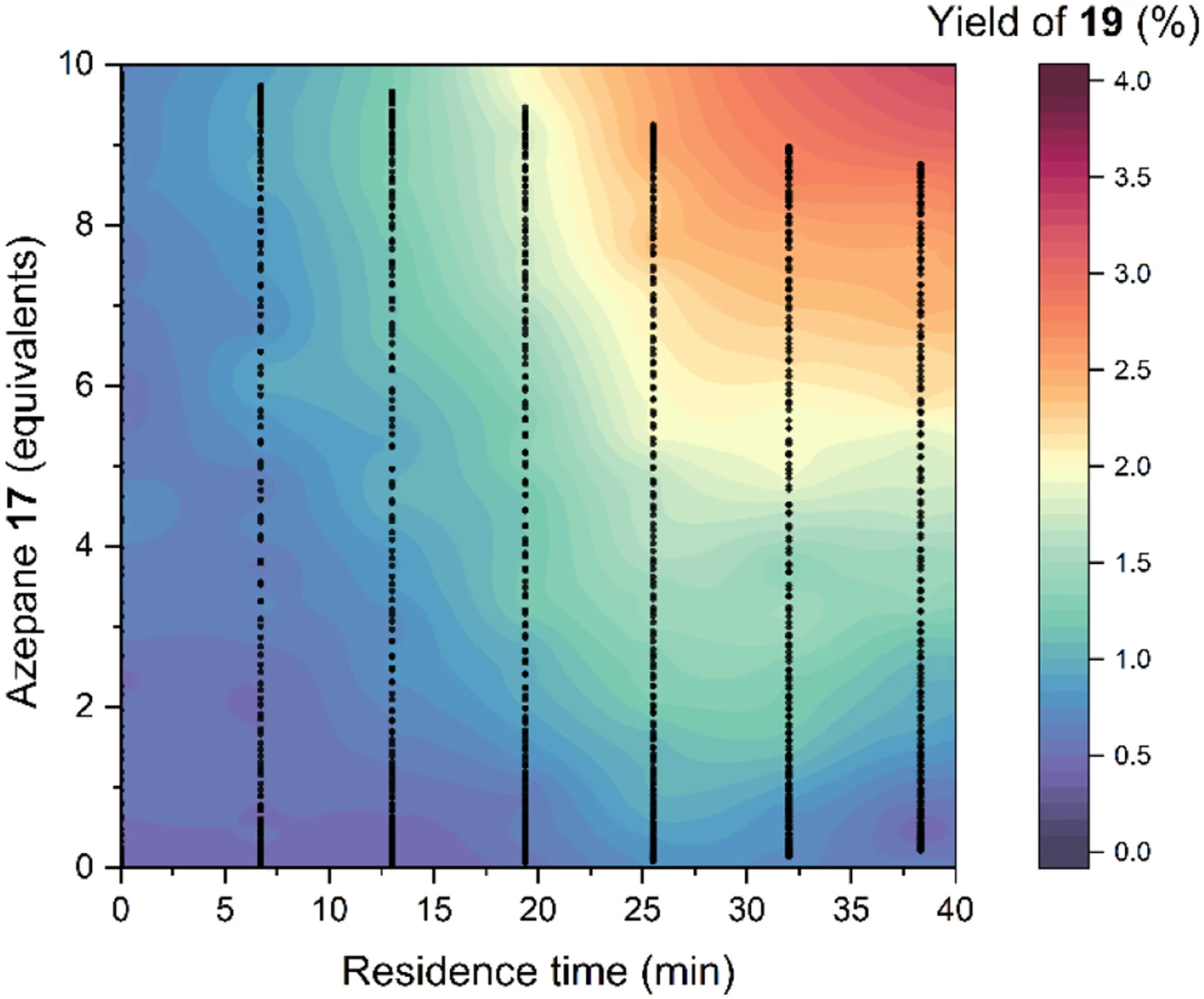
Yield of 19*vs.* [17] and residence time obtained from calibrated in-line UV-vis monitoring.

Validation of the obtained results was then performed for S_N_Ar reaction of 13 with 2.0, 5.0, and 8.0 equivalents of 14 using conventional steady-state flow experiments (Fig. 34) and showed excellent agreement (with maximum error less than 5%) with the data obtained from the unidirectional cyclic flow with the multi-concentration-gradient method.

**Fig. 34:**
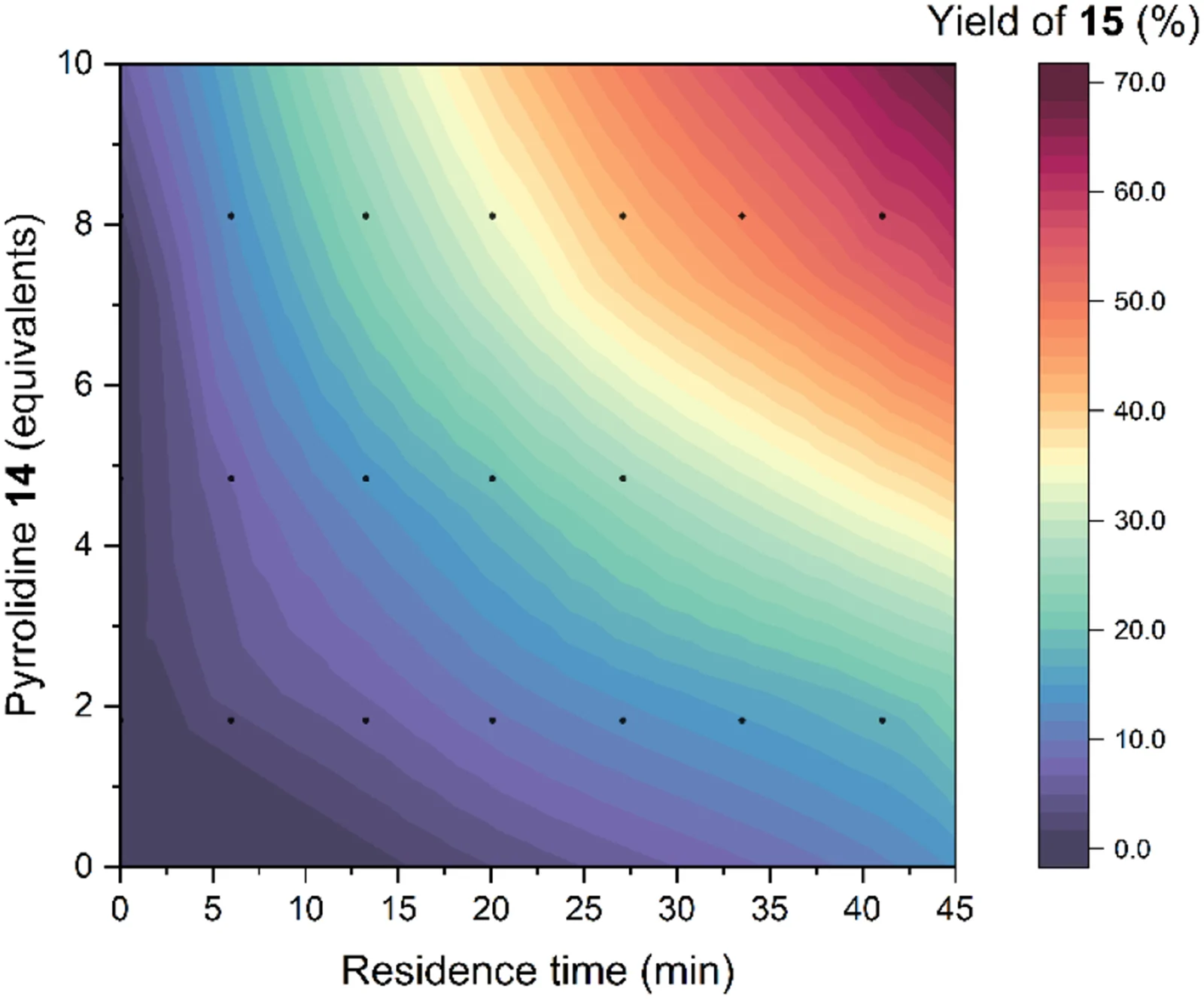
Yield of 15*vs.* concentration of pyrrolidine 14 and residence time obtained from calibrated in-line UV-vis monitoring of flow steady state experiments.

## Conclusions

A novel methodology using dispersion-generated concentration gradients that allows the effect of a range of reaction component concentrations on a process outcome to be investigated in a single flow experiment without needing dynamic control of flow rates has been developed. The methodology exploits natural and usually unwanted Taylor dispersion phenomena by passing a plug of the reactant under study through a tube to give concentration gradients at the leading and trailing ends of the plug. Subsequent combination with a steady-state stream of other reactants and passage through a flow reactor with in-line or off-line data acquisition on the products allows the effects of all concentrations of the dispersed reagent up to a maximum to be measured in a single short experiment.

Application of the newly developed dispersion-generated concentration gradient methodology was demonstrated and validated by application to the kinetic analysis of model reactions. For the thermal fragmentation of 1,3-dioxin-4-one a linear 1st-order plot was obtained and the rate constant closely matched values obtained *via* other methods. The Diels–Alder cycloaddition of isoprene and methyl acrylate gave linear second-order plot. These results highlight the efficacy of the dispersion-generated concentration gradient technique for rapid kinetic analysis in flow chemistry. For more complex kinetic analysis the method should be complementary to other batch methods using concentration *vs.* reaction time data. Particular strengths will be the determination of initial rates of reactions as the starting concentration of a component is varied and probing the roles of components which are not consumed in the studied reaction (for example catalysts, ligands, other additives) for which time domain data are less useful.

The methodology was then applied to the investigation of Heck and Sonogashira coupling reactions using flow chemistry and dispersion-generated concentration gradient techniques. For the Heck coupling, palladium diacetate was identified as the optimal catalyst source with ligand-free conditions providing full conversion. The effects of various phosphine and nitrogen-based ligands were explored, revealing that most phosphines and N-containing ligands inhibited the reaction to varying degrees. Additives such as sulphur-containing compounds and heterocycles were also examined, with nucleophilic thiols causing rapid inhibition while less reactive compounds like DMSO had minimal impact. The presence of competing alkenes and alkynes generally led to reaction inhibition, with the extent depending on the specific compound. Surprisingly, in the case of the Sonogashira coupling, triphenylphosphine was found to be optimal at much higher concentrations (around 47 eq.) than traditionally used. Various phosphines, heterocycle-containing, and bidentate phosphines were screened, with P(*p*-tolyl)_3_ showing faster conversion than PPh_3_ at lower equivalencies, and diphenyl-2-pyridyl phosphine showing the fastest conversion at 72.5 equivalents. The study demonstrates the power of flow chemistry and dispersion-generated concentration gradients in rapidly optimizing reaction conditions and understanding the impact of various additives and ligands on organic transformations.

Moreover, an advanced methodology for creating multi-time point concentration gradients in flow chemistry, overcoming the limitation of single time point analysis in traditional flow systems, has been described. Two approaches to cyclic flow were developed, namely reciprocating and unidirectional flows, which allowed for the generation of time series data from a single flow experiment, significantly enhancing the efficiency of kinetic studies. Further methodology refinement involved gradient clipping, allowing for selective injection of only a concentration gradient portion of the dispersed reactant plug as well as sequential injection of multiple dispersed reactants.

Such an advanced system was used to study the S_N_Ar reaction with three different amines, demonstrating its capability to efficiently generate large amounts of kinetic data in a single flow experiment. Results obtained from unidirectional flow experiments employing multiple dispersion-generated concentration gradients demonstrate the significant advantages over conventional dynamic flow-rate control. The methodology enables the generation of well-defined concentration gradients within exceptionally small volumes and over short residence times, facilitating the rapid acquisition of high-quality, information-rich datasets. This high experimental efficiency is particularly advantageous when combined with clipping strategies and fast in-line analytical methods, where rapid sampling and dense data acquisition are essential. Collectively, these attributes establish the approach as a powerful platform for accelerating process characterization while improving the resolution and statistical robustness of experimental datasets.

## Author contributions

Dawid Drelinkiewicz: formal analysis, visualisation, writing – original draft, writing – review & editing. Stephen T. Alston: data curation, investigation, formal analysis, validation. Thomas Durand: data curation, investigation, formal analysis. Richard J. Whitby: conceptualisation, funding acquisition, methodology, resources, supervision, writing – review and editing.

## Conflicts of interest

There are no conflicts to declare.

## Supplementary Material

RE-OLF-D6RE00378H-s001

## Data Availability

The data supporting this article have been included as part of the supplementary information (SI). Supplementary information is available. See DOI: https://doi.org/10.1039/d6re00378h.
