# Supplementary material for: Dispersion-generated concentration gradients and cyclic flow for reaction optimisation and kinetic analysis
Source: React Chem Eng. 2026 Oct 14. Online ahead of print. doi: 10.1039/d6re00378h (PMC13737367; doi:10.1039/d6re00378h)
Supplement: Supplementary file 1 [file RE-OLF-D6RE00378H-s001.pdf]

Supplementary Information (SI) for Reaction Chemistry & Engineering.
This journal is © The Royal Society of Chemistry

Dispersion-Generated Concentration Gradients and Cyclic Flow for Reaction Optimisation and Kinetic Analysis

Dawid Drelinkiewicz, Stephen T. Alston, Thomas Durand, and Richard J. Whitby

Supporting Information

Table of contents

1. Materials and methods 3
1. 1. Materials 3
1. 2. Methods 3
2. Experimental procedures and results 4
2. 1. Example flow setup for quantification of concentration gradient by dispersion utilising tracer and in-line monitoring 4
2. 2. Retro-Diels-Alder reaction of 2,2,6-trimethyl-*4H*-1,3-dioxin-4-one 6
2. 2. 1. Steady state kinetic measurements[1] 6
2. 2. 2. Experiments utilising dispersion-generated concentration gradient method 9
2. 3. Diels-Alder cycloaddition of isoprene and methyl acrylatesrf 10
2. 3. 1. GC Calibration of methyl acrylate 10
2. 3. 2. Experiments utilising dispersion-generated concentration gradient method 11
2. 4. Heck coupling between iodobenzene and butyl acrylate 12
2. 4. 1. GC calibration of *n*-butyl cinnamate 12
2. 4. 2. Experimental protocol for flow studies utilising dispersion-generated concentration gradient method in Heck coupling reaction, example procedure of palladium diacetate dispersion 13
2. 4. 3. Results of dispersion-generated concentration gradient method in Heck coupling reaction, GC and IR traces of process components, dispersion profiles, and yield as a function of dispersed component concentration 14
2. 4. 4. Validation of results using steady-state, variable flow rate (VFR) generated concentration gradient, and dispersion-generated concentration gradient flow methods 23
2. 5. Sonogashira coupling reaction between bromobenzene and 1-octyne 25
2. 5. 1. Sample GC trace of Sonogashira reaction 25

2. 5. 2. Experimental protocol for flow studies utilising dispersion-generated concentration gradient method in Sonogashira coupling reaction, example procedure of XantPhos dispersion 25

2. 5. 3. Results of dispersion-generated concentration gradient method in Sonogashira coupling reaction, GC and IR traces of process components, dispersion profiles, and yield as a function of dispersed component concentration 26

2. 6. Calibration of analytical instruments for S_NAr reaction between 1-fluoro-4-nitrobenzene and pyrrolidine, piperidine, or azepane 31

2. 7. S_NAr reaction between 1-fluoro-4-nitrobenzene and pyrrolidine within reciprocating cyclic flow system with single concentration gradient 32

2. 8. S_NAr reaction between 1-fluoro-4-nitrobenzene and pyrrolidine within unidirectional cyclic flow system 35

2. 9. S_NAr reaction between 1-fluoro-4-nitrobenzene and pyrrolidine within unidirectional cyclic flow system utilising single dispersion-generated concentration gradient........ 38

2. 10. S_NAr reaction between 1-fluoro-4-nitrobenzene and pyrrolidine, piperidine or azepane within unidirectional cyclic flow system utilising multiple dispersion-generated concentration gradients with gradient clipping........ 43

2. 11. Procedure for validation of S_NAr between 1-fluoro-4-nitrobenzene and pyrrolidine reaction using steady-state conditions........ 45

3. Dispersion in glass bead packed tubular reactors 46

4. Syntheses and characterization data 48

4. 1. Methyl 4-methylcyclohex-3-ene-1-carboxylate (6a) and methyl 3-methylcyclohex-3-ene-1-carboxylate (6b): 48

4. 2. 1-(4-Nitrophenyl)pyrrolidine (15): 50

4. 3. 1-(4-Nitrophenyl)piperidine (18): 51

4. 4. 1-(4-Nitrophenyl)azepane (19): 51

5. References........ 52

1. Materials and methods

1. 1. Materials

All solvents and commercially available chemicals, 2,2,6-trimethyl-4*H*-1,3-dioxin-4-one **1**, ethyl acetoacetate **3**, isoprene **4**, methyl acrylate **5**, iodobenzene **7**, *n*-butyl acrylate **8**, *n*-butyl cinnamate **9**, bromobenzene **10**, 1-octyne **11**, oct-1-yn-1-ylbenzene **12**, 1-fluoro-4-nitrobenzene **13** were used as received, unless otherwise stated. Pyrrolidine **14**, piperidine **16**, and azepane **17** were distilled over CaH_2 prior to use.

1. 2. Methods

NMR spectra were obtained on Bruker DPX400 or Bruker AvanceCore AVII 400 spectrometers. ^{1}H chemical shifts are reported as values in ppm referenced to the deuterated solvent main peak. The following abbreviations are used to assign multiplicity: s = singlet, d = doublet, t = triplet, q = quartet, qu = quintet, sx = sextet, hpt = heptet, oct = octet, br = broad. Coupling constants, J, are measured in Hertz (Hz) and if indicated are reported as $^{X}J_{Y\text{-}Z}$, where X indicates the number of bonds between coupled nuclei and Y-Z indicates the nuclei. ^{13}C chemical shifts are reported as values in ppm referenced to the main peak of deuterated solvent and are proton decoupled. If indicated, in the ^{13}C NMR spectra the nature of carbons (CH, CH_2 or CH_3) was determined by DEPT-135 experiment.

Samples for off-line analysis were collected using Gilson Prep FC fraction collector. GC measurements were performed on a Hewlett Packard HP 6890 series GC system, using a HP-5 (cross-linked 5% Ph Me siloxane) 30 m column, with a film thickness of 0.25 µm and 0.32 mm internal diameter. The carrier gas was helium and the flow rate was 2.7 $mL{\cdot}min^{-1}$. The injector was maintained at 300 °C with 1.0 µL injection. The run started at 80 °C with a gradient of 25 °C/min until 275 °C which was held for 4 min.

Infrared spectra were run as neat films on a Thermo Nicolet 380 FT-IR spectrometer with a Smart Orbit Goldengate ATR attachment. In-line IR measurements were performed using Bruker Alpha transmission FT-IR spectrometer with a Harric DCL2™ demountable liquid flow cell with sodium chloride plates.

UV-Vis measurements were recorded with the Ocean Optics DH-2000-BAL light source and USB 2000+ fibre optic spectrometer at room temperature using SpectraSuite Software. All flow *in situ* UV-Vis measurements were performed with a Type 583-F Starna® fluorimeter flow cell. The wavelengths are given in nanometres and the corresponding extinction coefficients in $M^{-1}{\cdot}cm^{-1}$.

In-line UV-Vis or IR data were interpreted using Bristol Online Reaction Investigation Software (BORIS). Spectra were either interpreted via peak picking or orthogonal projection approach. Data extracted using BORIS was exported to an Excel spreadsheet. Areas of peaks were normalised to an internal standard and if necessary, a calibration curve was created to extract yields from the data. Multi-time point data was manipulated using custom Python scripts.

Thin-layer chromatography was carried out on Merck silica gel plates, which were visualised under UV irradiation of 254 nm and/or by staining with aqueous $KMnO_4$, methanolic H_2SO_4, PMA or iodine. Column chromatography was performed with Merck silica gel 60 using solvent ratios as volumes before mixing described in the method.

The Vapourtec® R series Integrated Flow Chemistry System (R2+) was the platform used for the flow experiments. Vapourtec R2 unit piston pumps were used for liquid delivery. Flow reactor temperature

control was provided by submersing the reactor in an oil bath. The reactors used were made of PFA or stainless steel tube, depending on the required reaction conditions.

2. Experimental procedures and results

2. 1. Example flow setup for quantification of concentration gradient by dispersion utilising tracer and in-line monitoring

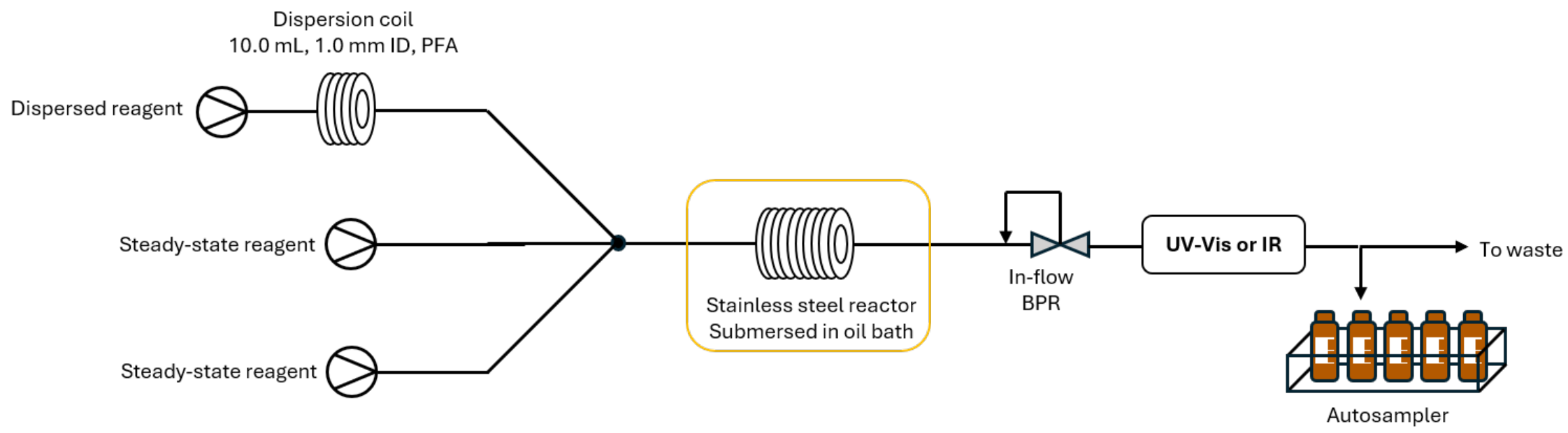

Figure S1. Example flow setup used for quantification of dispersion in fluidic system.

A dispersion profile was measured by dissolving diphenylfulvene (10.0 mg, 0.042 mmol) in DMF (20.0 mL) and connecting to the flow line equipped with dispersion coil. Reactor was set to 130 °C, after temperature stabilisation all pumps were set to 0.1 mL/min and started. UV-Vis spectrometer was continuously monitoring absorbance.

Graph below shows example dispersion of diphenylfulvene (Figure S2).

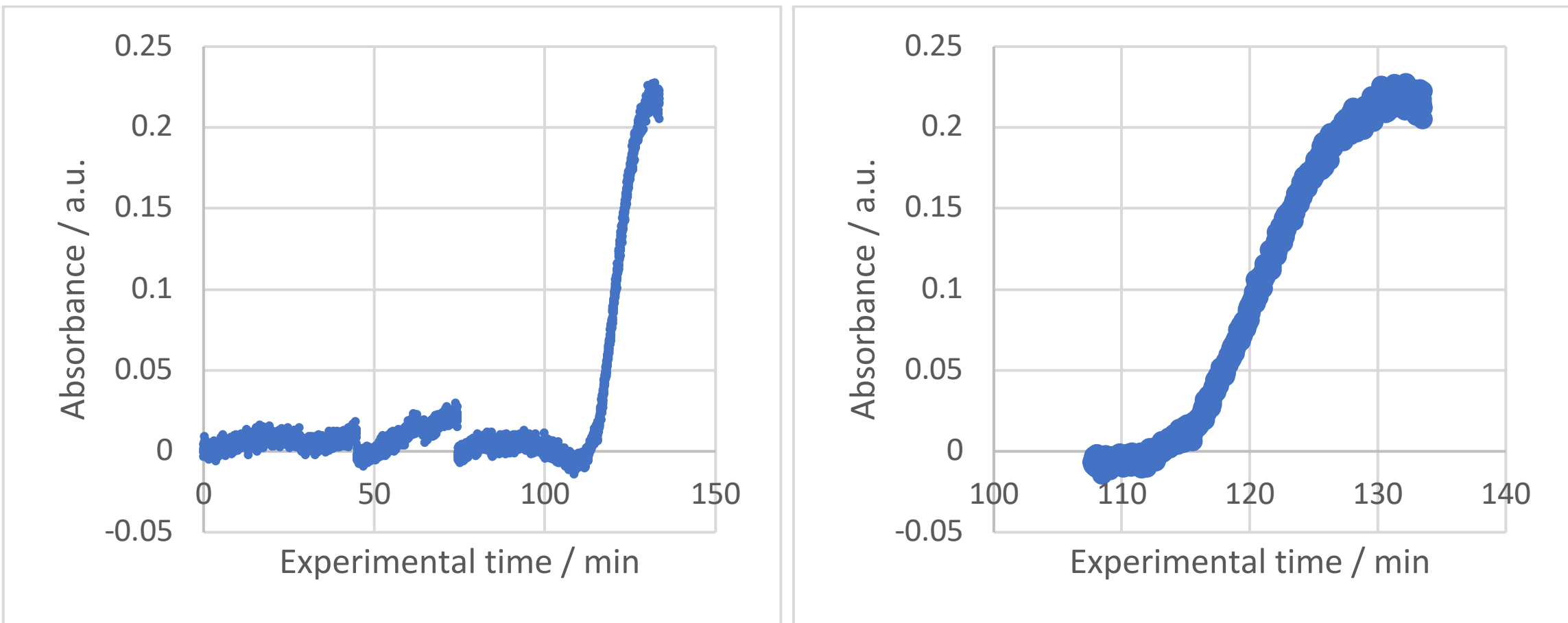

Figure S2. Absorbance of diphenylfulvene in flow system used for studies of Heck reaction via dispersion generated concentration gradient methodology. Left: Full experiment UV-Vis trace; note each step reduction in absorbance (i.e. at 48 and 75 min) is the result of background correction. Right: Absorbance in the region where front plug of dispersed diphenylfulvene was observed.

The obtained dispersion profile in the region of interest was then copied into the experimental results obtained from GC data and normalised in the range from 0 to maximum concentration of the studied dispersed reagent (Figure S3).

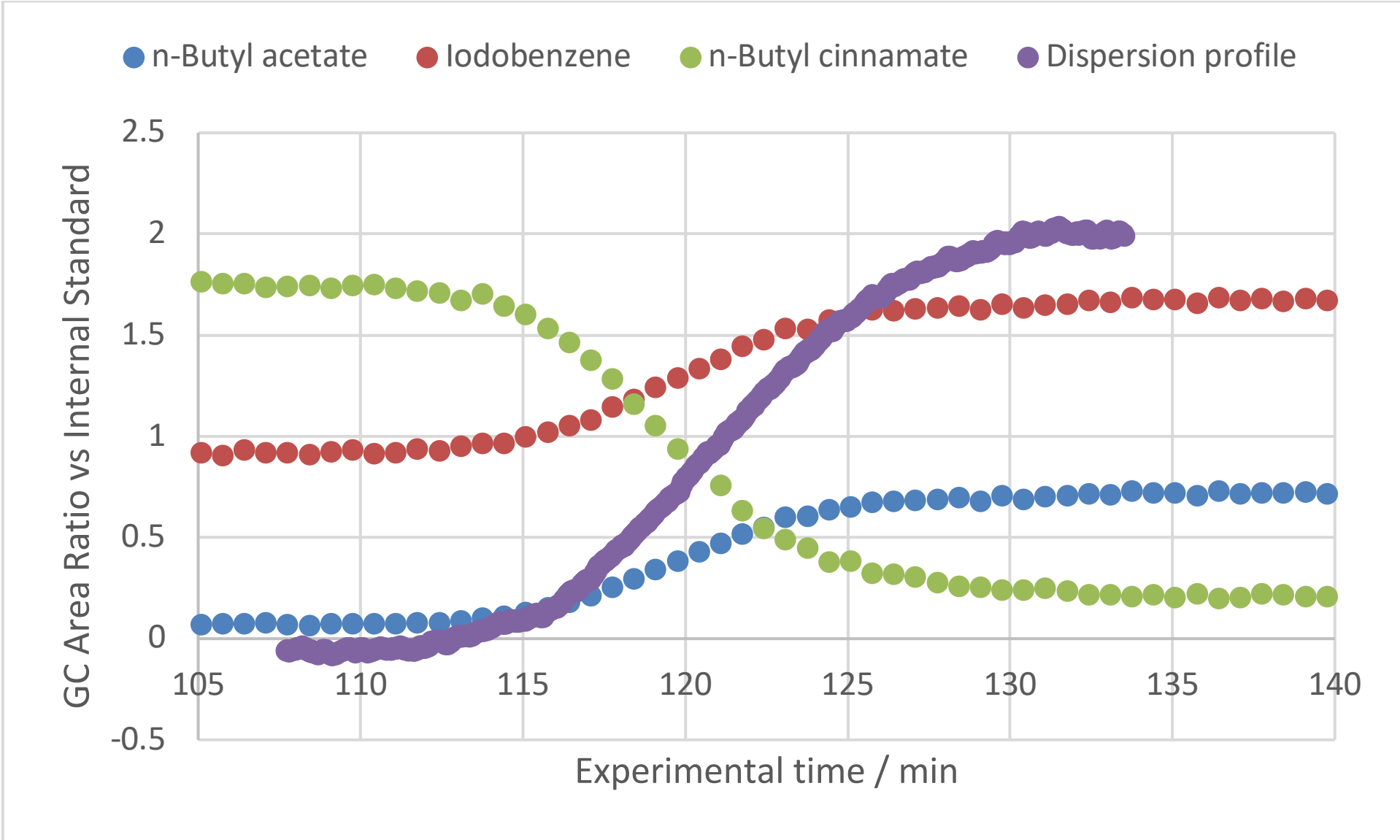

Figure S3. GC traces of reaction components and dispersion profile from the Heck reaction investigation of pyridine concentration gradient effect.

Further calculation of reaction conversion or yield from GC traces of *n*-butyl acrylate or *n*-butyl cinnamate and plotting against the concentration of dispersed reagent, in this case pyridine, obtained from dispersion profile allows for visualisation of the effect of various pyridine concentrations on reaction outcome (Figure S4).

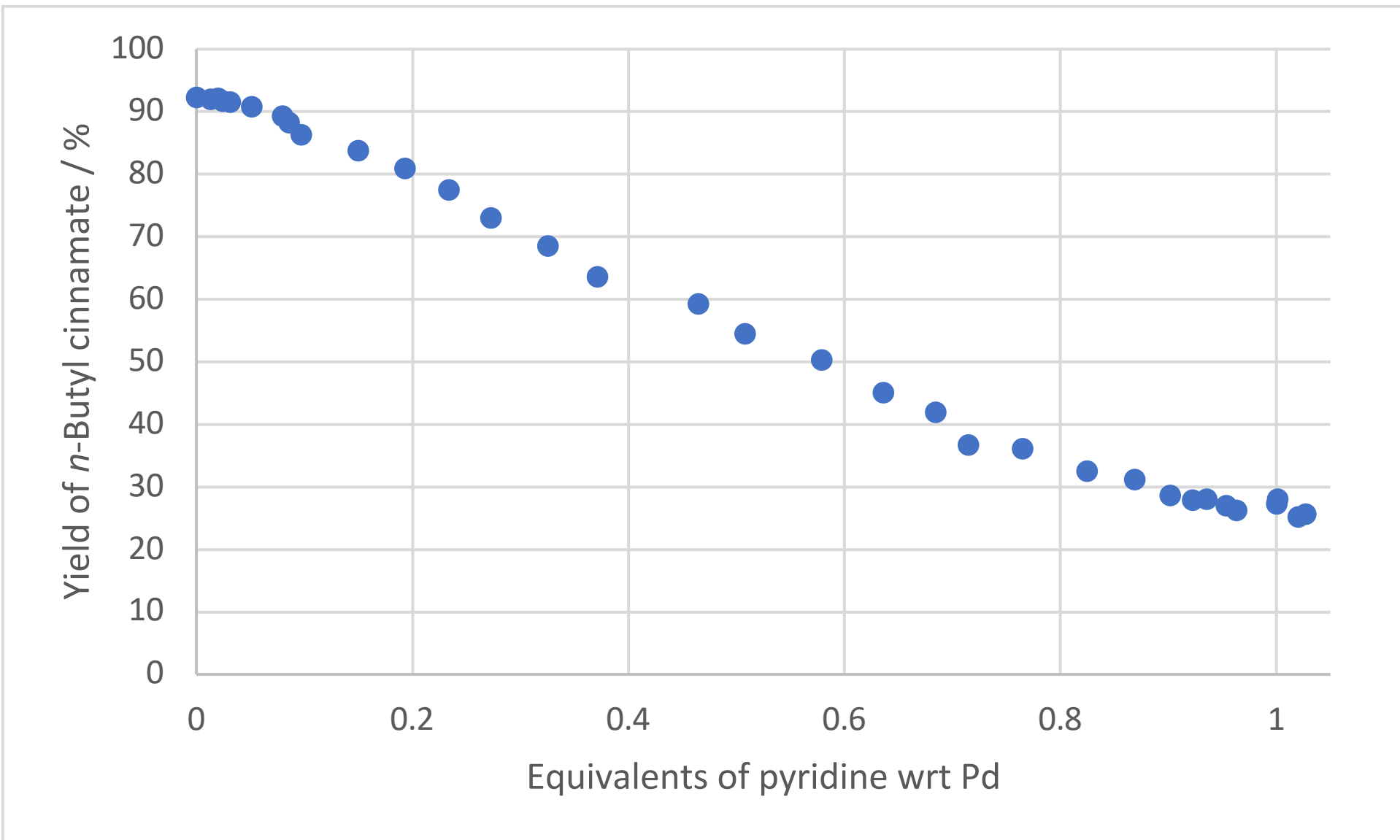

Figure S4. Yield of n-butyl cinnamate at various pyridine concentrations. Pd at 0.225 mol%.

2. 2. Retro-Diels-Alder reaction of 2,2,6-trimethyl-*4H*-1,3-dioxin-4-one

2. 2. 1. Steady state kinetic measurements[1]

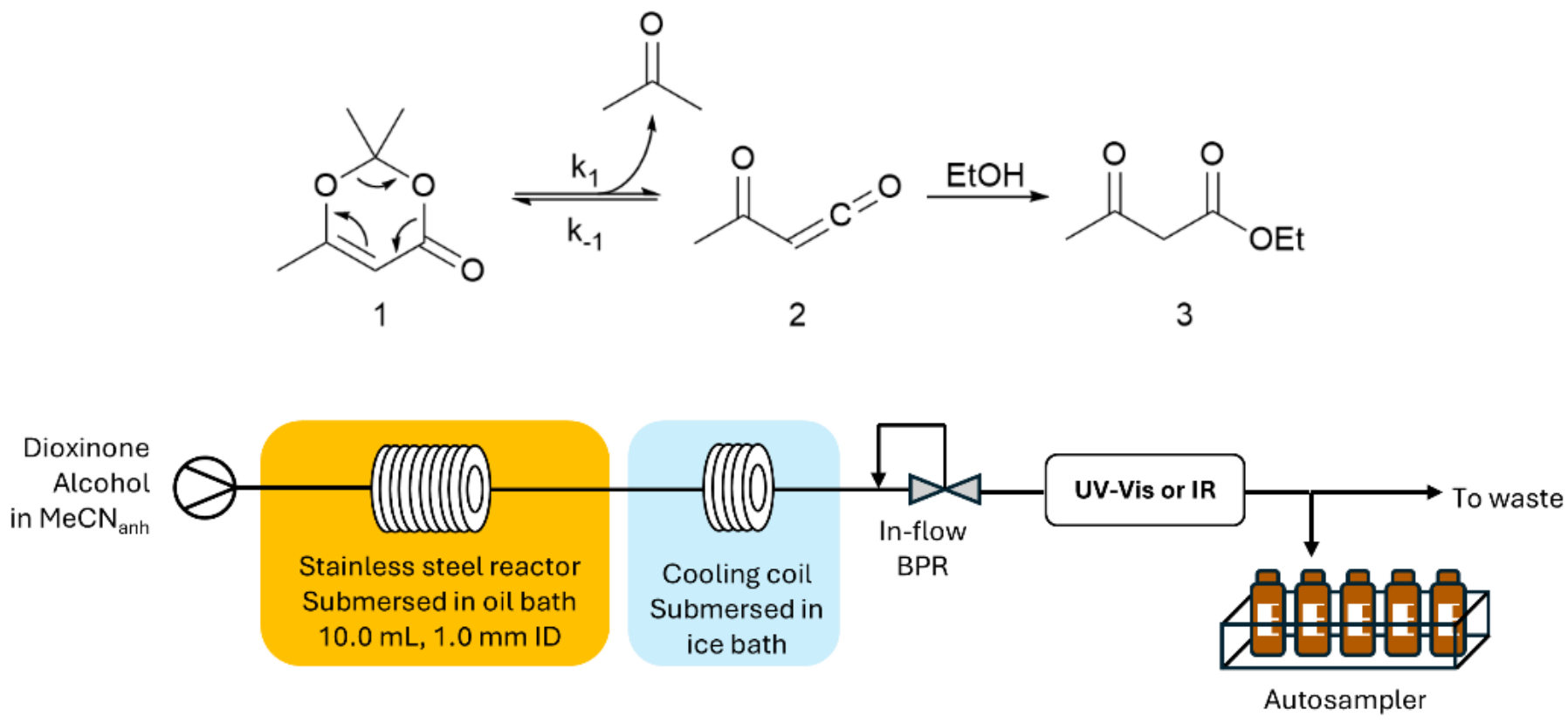

Figure S5. Top: Dioxinone 1 thermolysis scheme. Bottom: Flow used in steady-state experiments of dioxinone 1 thermolysis.

In a RBF flushed with nitrogen, 2,2,6-trimethyl-4*H*-1,3-dioxin-4-one **1** (1 equiv., [dioxinone] = 1 mM for UV analysis or 0.015 - 0.3 M for IR analysis) and EtOH (1.0, 2.0, or 4.0 eq.) were mixed with $MeCN_{anh}$. The reaction mixture was degassed by sonication whilst being saturated with nitrogen and was then connected to pump reagent stream. Vessel containing solvent ($MeCN_{anh}$) was connected to pump solvent stream. Flow system was composed of a piston pump (Vapourtec R2 unit) delivering solvent or reaction mixture to the dispersion coil (coiled PFA, 10.0 mL, 1.0 mm ID), tubular reactor (stainless steel, 10.0 mL, 1.0 mm ID), cooling coil (in ice bath), BPR (250 psi), in-line UV-Vis or IR flow cell and autosampler. Solvent (MeCN) was pumped at the desired flow rate, and the reactor was set to a desired temperature. Once the temperature was stable, the feeding valve was switched to deliver the reagent solution, and the reaction mixture was injected. At the end of the sample plug injection, the feeding valve was returned to the solvent feed. This procedure was repeated at several reaction temperatures, ensuring equilibration of temperature prior to injection of sample plug.

The completed cycle of multiple experiments (typically five within a cycle, across a range of flow rates from 2 $mL{\cdot}min^{-1}$ to 9 $mL{\cdot}min^{-1}$, Figure S6) was repeated at several temperatures. The reaction time was calculated from each flow rate displayed on the flow system with a correction for thermal expansion of acetonitrile according to $Q_{effective} = Q_{pump}\ (1 + \alpha\Delta T)$, where α = the expansion coefficient of the acetonitrile (see Table S1 for calculated $Q_{effective}$). IR and UV spectra were processed with BORIS software by calibration-less methods (OPA processing or height of non-overlapping peaks processing) and by using the spectra of 0% and 100% conversion experiments respectively conducted at 30 °C and 160 °C as references to scale the spectroscopic data between 0 and 1.

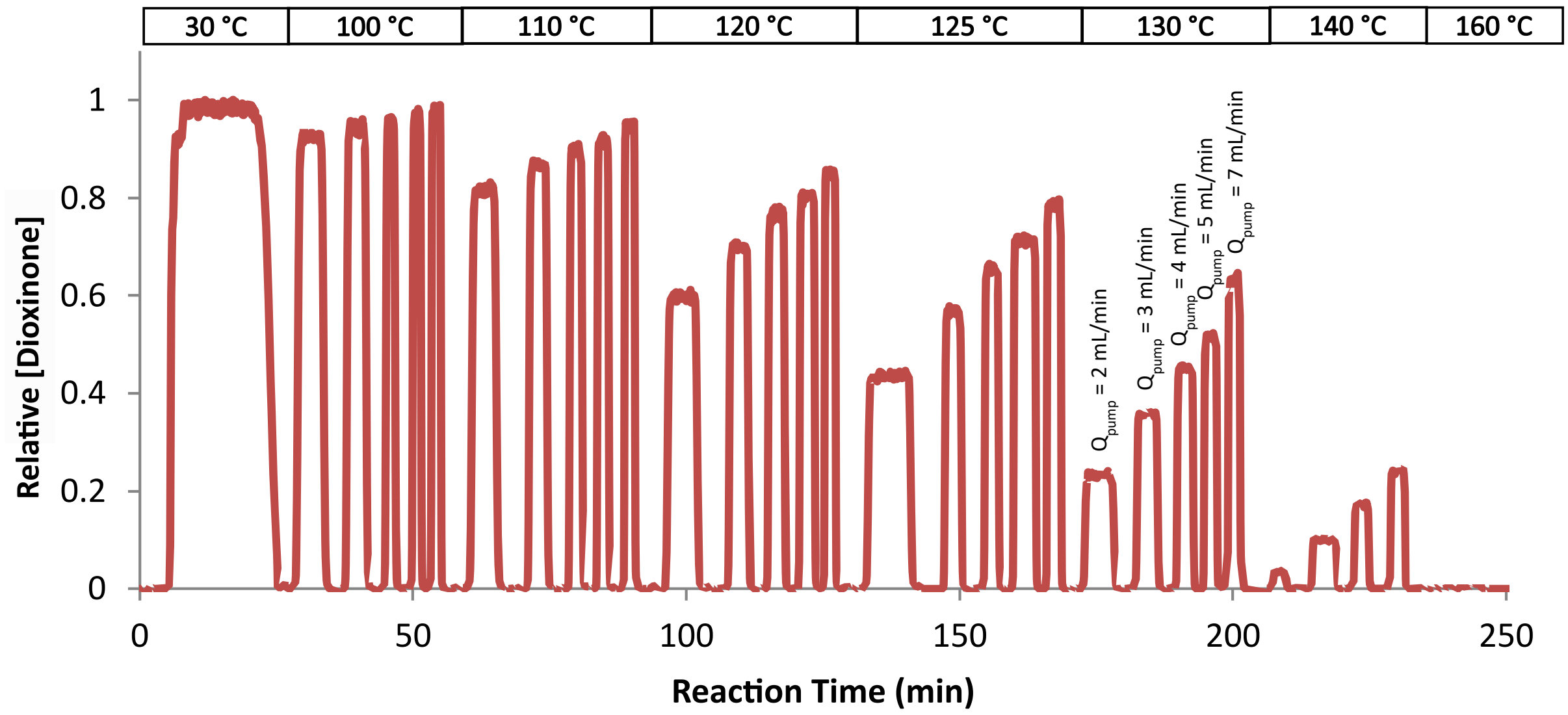

Figure S6. Concentration profile of 2,2,6-trimethyl-4H-1,3-dioxin-4-one after thermolysis in the presence of EtOH (4 equiv.) under steady-state conditions.

Table S1. Effective flow rates corrected for expansions of acetonitrile at elevated temperatures.

**Q pump** (mL·min^{-1})	**Q effective** (mL·min^{-1})					
	100 °C	110 °C	120 °C	125 °C	130 °C	140 °C
2	2.19728	2.22468	2.25208	2.26578	2.27948	2.30688
3	3.29592	3.33702	3.37812	3.39867	3.41922	3.46032
4	4.39456	4.44936	4.50416	4.53156	4.55896	4.61376
5	5.4932	5.56170	5.63020	5.66445	5.69870	5.76720
7	7.69048	7.78638	7.88228	7.93023	7.97818	-

Data from experiments conducted at 120 °C and with 1.0 eq. (Table S2 and Figure S7), 2.0 (Table S3 and Figure S8), or 4.0 eq. (Table S4 and Figure S9) of EtOH was extracted allowing for calculation of reaction rate:

$$k_1^{120\,°C,\ \ 1.0\,eq.EtOH} = 1.832 \cdot 10^{-3}[s^{-1}]$$

$$k_1^{120\,°C,\ \ 2.0\,eq.EtOH} = 1.871 \cdot 10^{-3}[s^{-1}]$$

$$k_1^{120\,°C,\ \ 4.0\,eq.EtOH} = 1.843 \cdot 10^{-3}[s^{-1}]$$

$$\bar{k}_1^{120\,°C} = 1.849 \cdot 10^{-3}[s^{-1}]$$

Table S2. Data obtained from steady state retro-Diels-Alder reaction with 1.0 eq. of ethanol at 120 °C.

**Q pump mL·min^{-1}**	**Q effective mL·min^{-1}**	**Residence Time / s**	**ln([1] / [1]$_0$)**
**9**	10.13	57.8	-0.095510180
**7**	7.88	74.3	-0.122217633
**5**	5.63	104.0	-0.178146185
**4**	4.50	130.0	-0.227135573
**3**	3.38	173.4	-0.307404700
**2**	2.25	260.0	-0.463734016

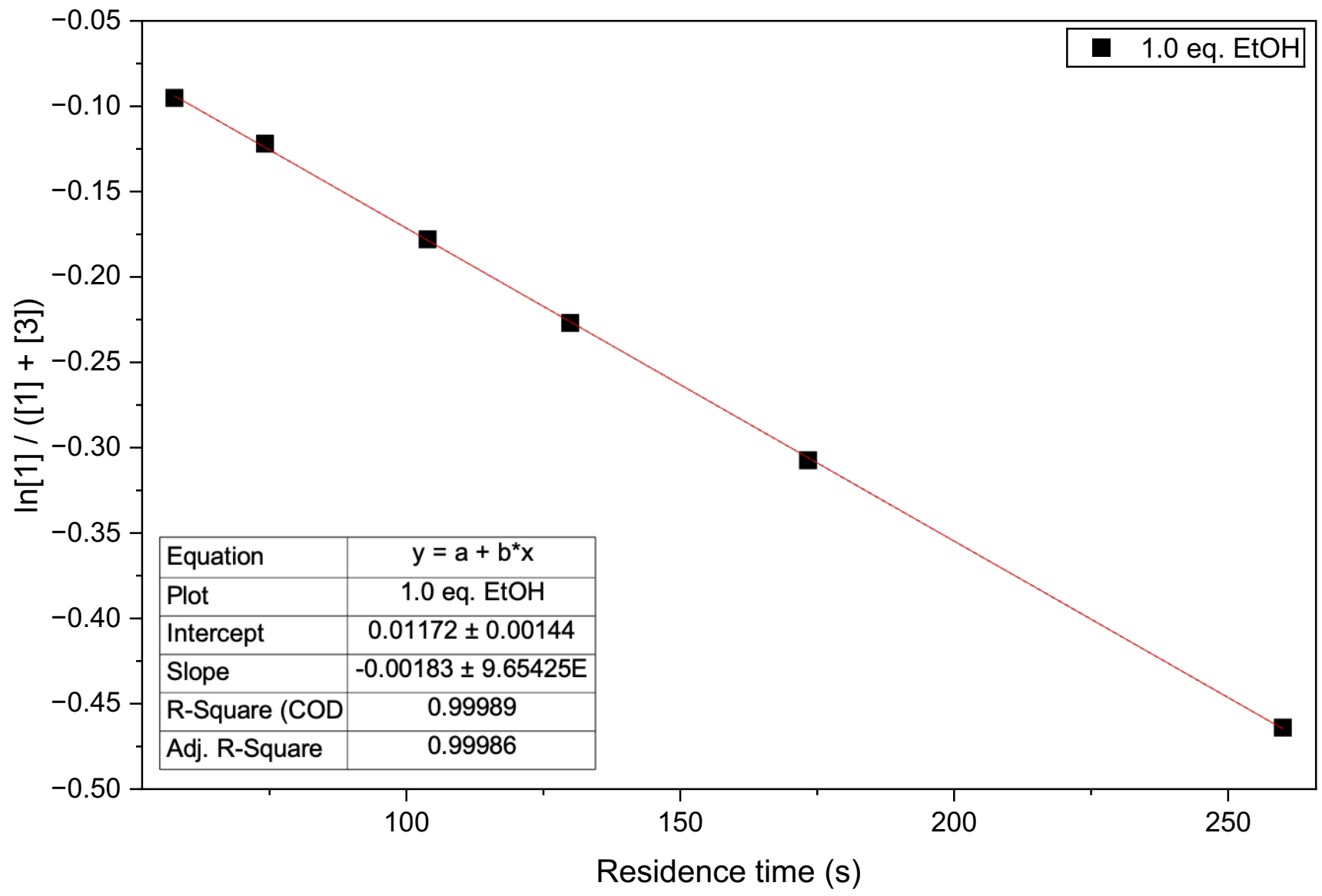

*Figure S7. Kinetic plot of retro-Diels-Alder reaction of **1** with 1.0 eq. of EtOH at 120 °C.*

Table S3. Data obtained from steady state retro-Diels-Alder reaction with 2.0 eq. of ethanol at 120 °C.

Q pump mL·min^{-1}	Q effective mL·min^{-1}	Residence Time / s	ln([1] / [1]$_0$)
**9**	10.13	57.8	-0.095510180
**7**	7.88	74.3	-0.122217633
**5**	5.63	104.0	-0.186479567
**4**	4.50	130.0	-0.235072122
**3**	3.38	173.4	-0.311154429
**2**	2.25	260.0	-0.471323757

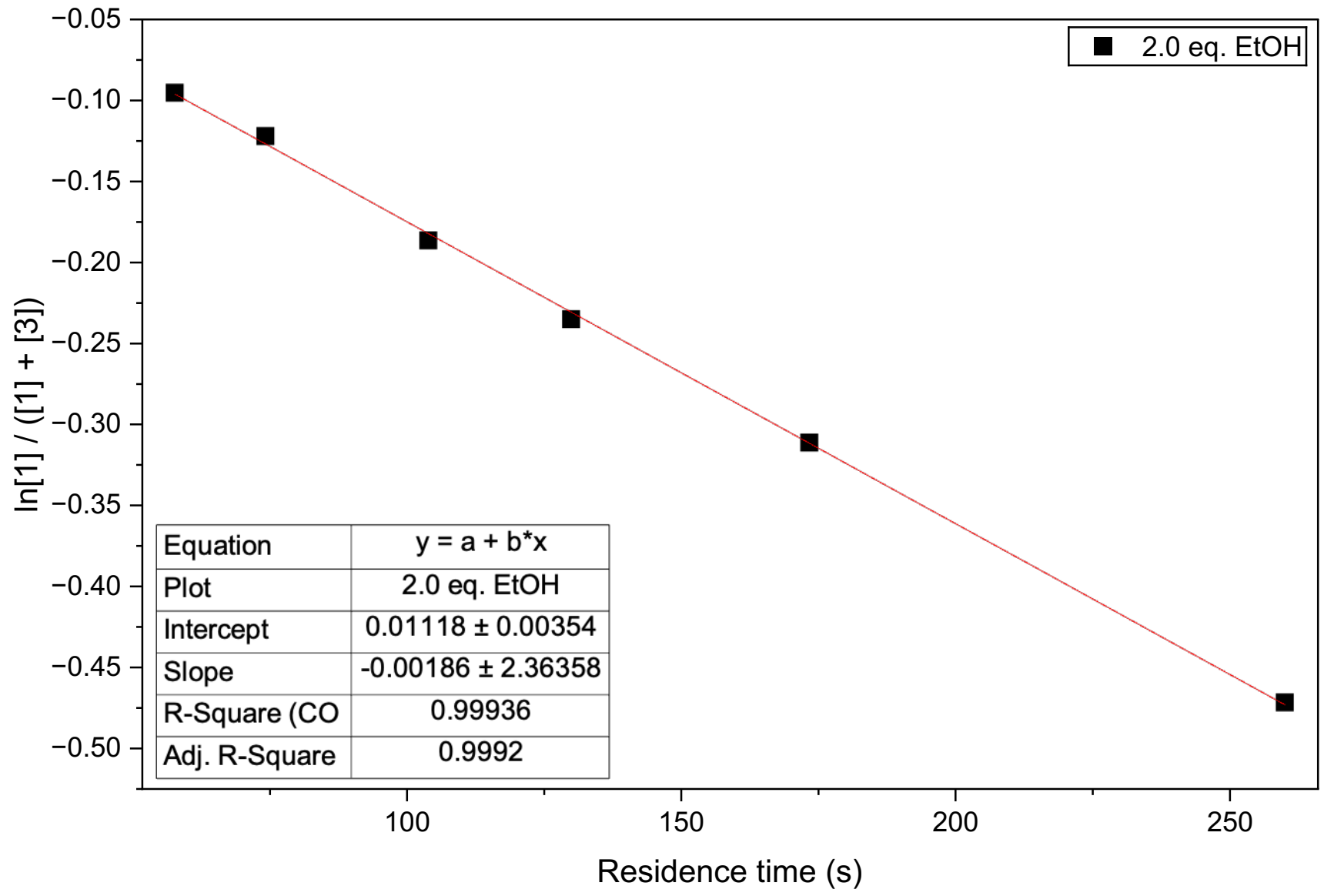

*Figure S8. Kinetic plot of retro-Diels-Alder reaction of **1** with 2.0 eq. of EtOH at 120 °C.*

Table S4. Data obtained from steady state retro-Diels-Alder reaction with 4.0 eq. of ethanol at 120 °C.

Q pump mL·min^{-1}	Q effective mL·min^{-1}	Residence Time / s	ln([1] / [1]$_0$)
**7**	7.88	74.3	-0.131008262
**5**	5.63	104.0	-0.190610360
**4**	4.50	130.0	-0.233721122
**3**	3.38	173.4	-0.314810740

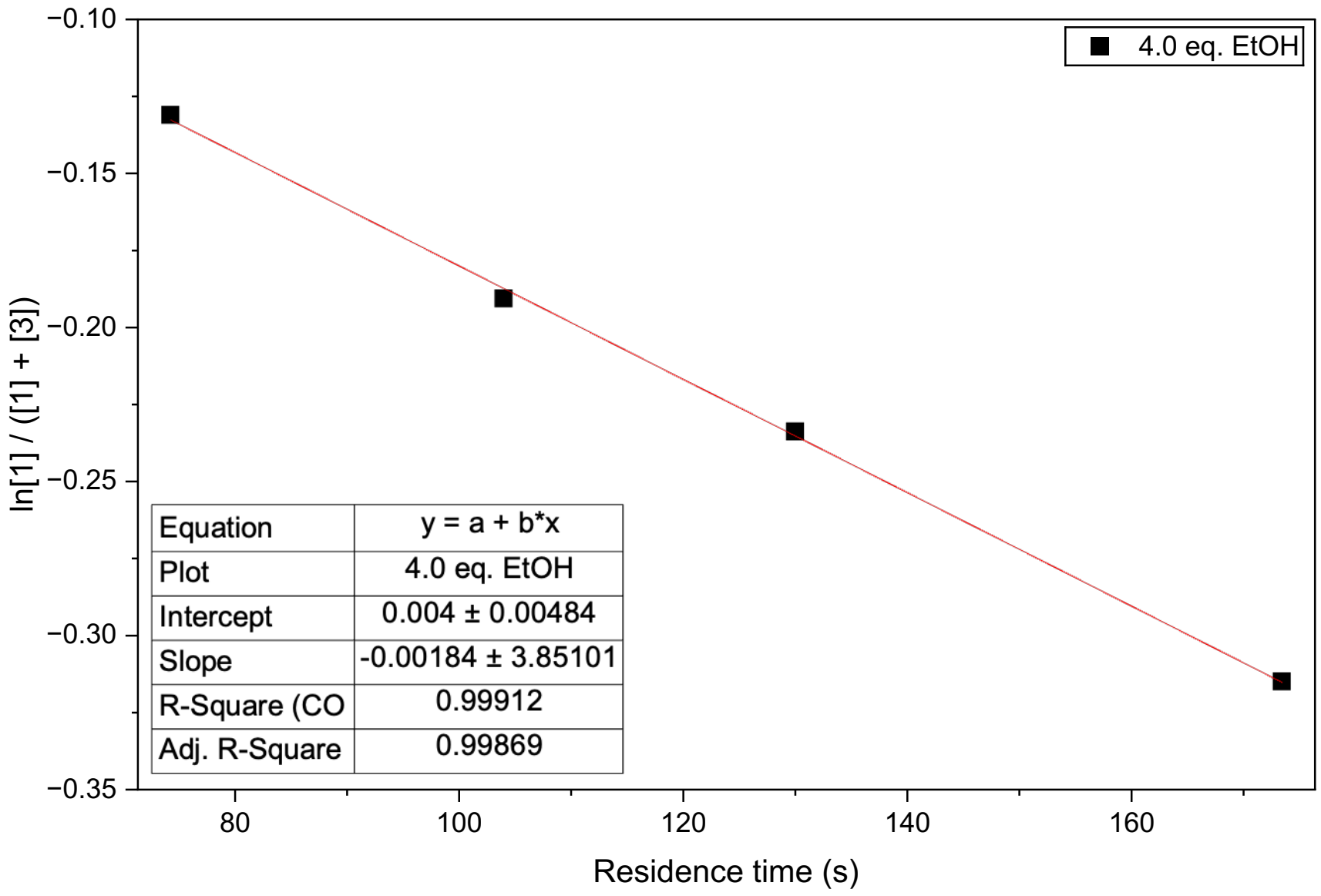

*Figure S9. Kinetic plot of retro-Diels-Alder reaction of **1** with 4.0 eq. of EtOH at 120 °C.*

2. 2. 2. Experiments utilising dispersion-generated concentration gradient method

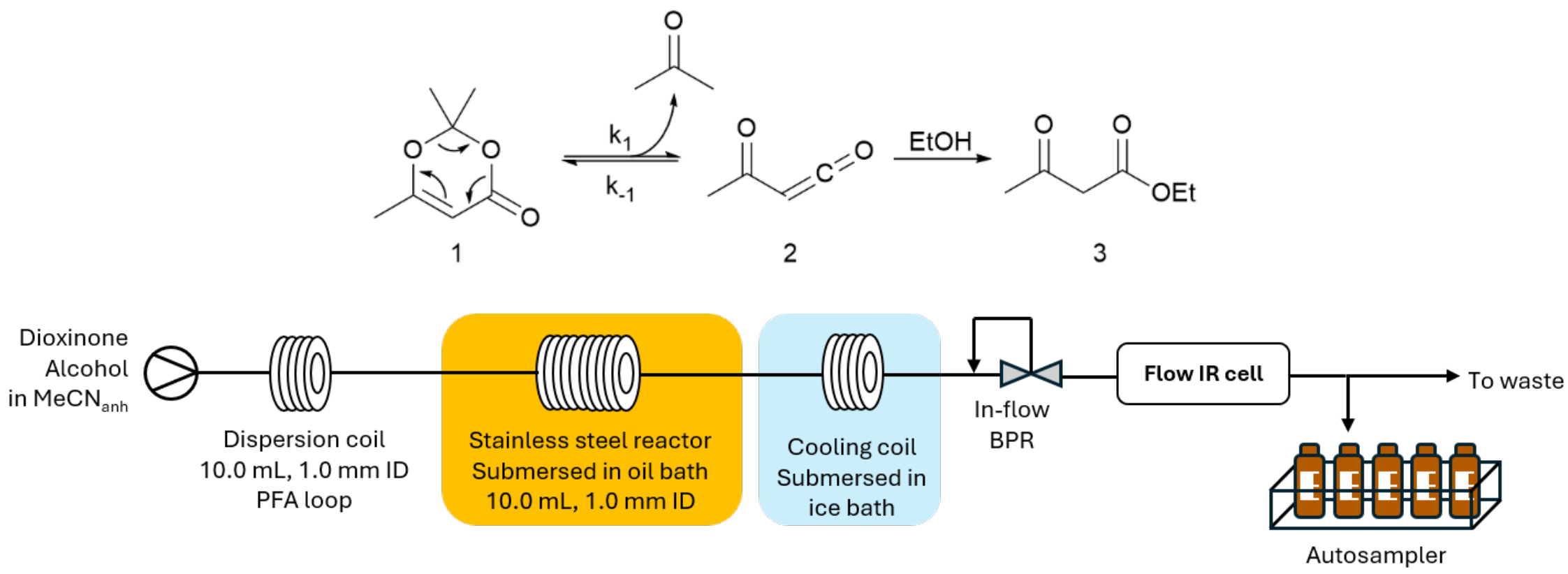

*Figure S10. Top: Thermolysis of dioxinone **1**. Bottom: Flow setup used in dispersion-generated concentration gradient experiments of dioxinone 1 thermolysis.*

In a RBF flushed with nitrogen, 2,2,6-trimethyl-4*H*-1,3-dioxin-4-one (1.50 mL, 11.3 mmol, 1.0 eq., 0.11 M solution) and EtOH (2.64 mL, 45.2 mmol, 4.0 eq., 0.44 M solution) were mixed with $MeCN_{anh}$ (100.0 mL). The reaction mixture was degassed by sonication whilst being saturated with nitrogen and was then connected to pump reagent stream. Vessel containing solvent ($MeCN_{anh}$) was connected to pump solvent stream. Flow system was composed of a piston pump (Vapourtec R2 unit) delivering solvent or reaction mixture to the dispersion coil (PFA, 10.0 mL, 1.0 mm ID), tubular reactor (stainless steel, 10.0 mL, 1.0 mm ID), cooling coil (in ice bath), BPR (250 psi), in-line IR flow cell and autosampler.

Solvent (MeCN) was pumped at the 0.3 $^{mL}/_{min}$ flow rate, and the reactor was set to a desired temperature. Once the temperature was stable, the feeding valve was switched to deliver the reagent solution and 5 mL of the reaction mixture was injected. At the end of the sample plug injection, the feeding valve was returned to the solvent feed. This procedure was repeated at several reaction temperatures, ensuring equilibration of temperature prior to injection of sample plug. The sample plug dispersed first in the 10 mL PFA loop and then the reaction occurred in the heated stainless-steel reactor. In-line IR detection was used, and the data sampling rate was determined by the acquisition parameters. The spectra were processed using BORIS software by calibration-less methods (OPA or height of non-overlapping peaks processing). When indicated, the reaction mixture was collected in vials for GC analysis.

2. 3. Diels-Alder cycloaddition of isoprene and methyl acrylatesrf

2. 3. 1. GC Calibration of methyl acrylate

Samples for GC were prepared by mixing the following amounts of methyl acrylate **5** in 1.5 mL of solvent (MeCN) (Table S5). A sample GC trace from Diels-Alder reaction studies and the GC calibration curve are presented below (Figure S11 and Figure S12, respectively).

Table S5. Table of samples used for calibration of GC for Diels-Alder starting-material quantification.

Volume of 5 (mL)	Concentration (mol/L)	GC Area of 1.265 min signal
**0.2**	1.31	9294
**0.15**	1.01	7116
**0.1**	0.69	4996
**0.05**	0.36	2511
**0.02**	0.15	996
**0.01**	0.07	494
**0.01**	0.07	468
**0.005**	0.04	238
**0.004**	0.03	195
**0.003**	0.02	140
**0.002**	0.01	91
**0.001**	0.01	38

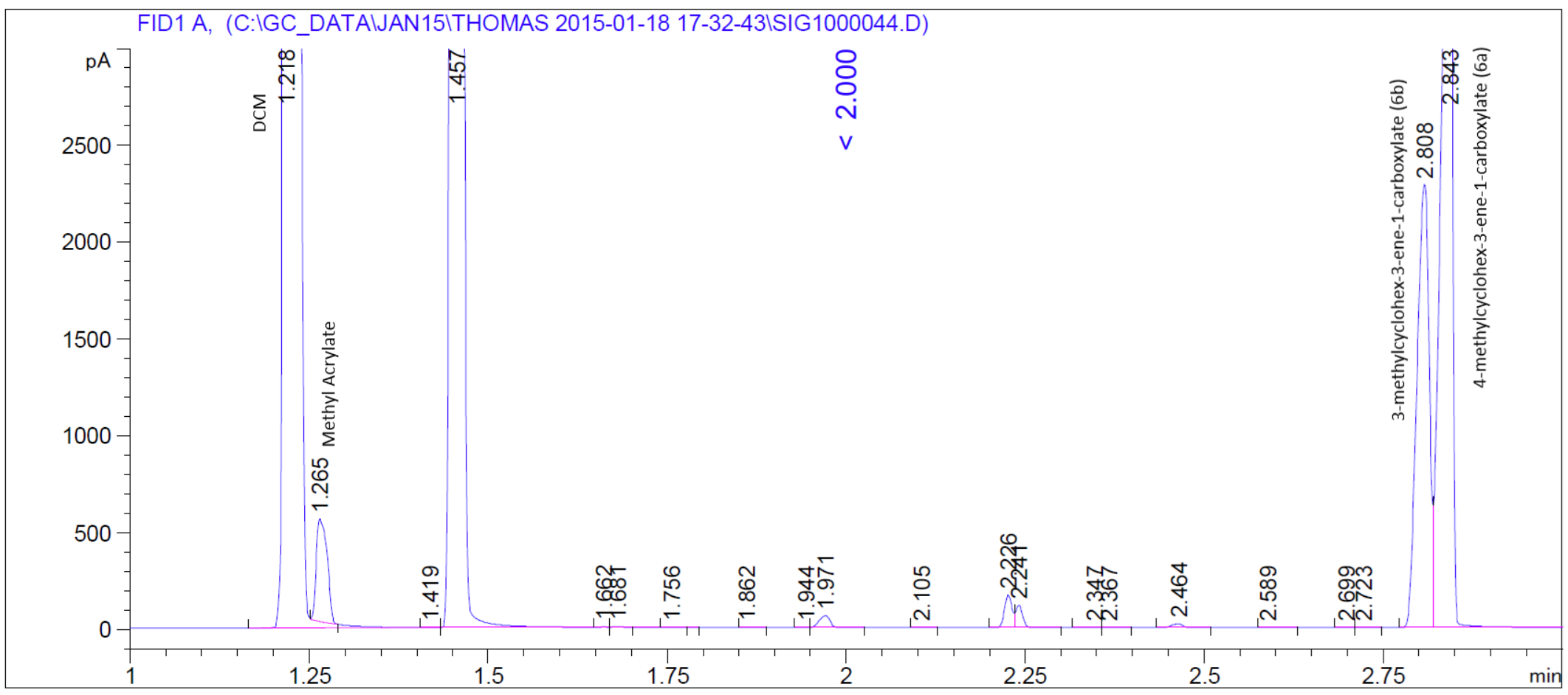

Figure S11. Example GC trace from the Diels-Alder reaction.

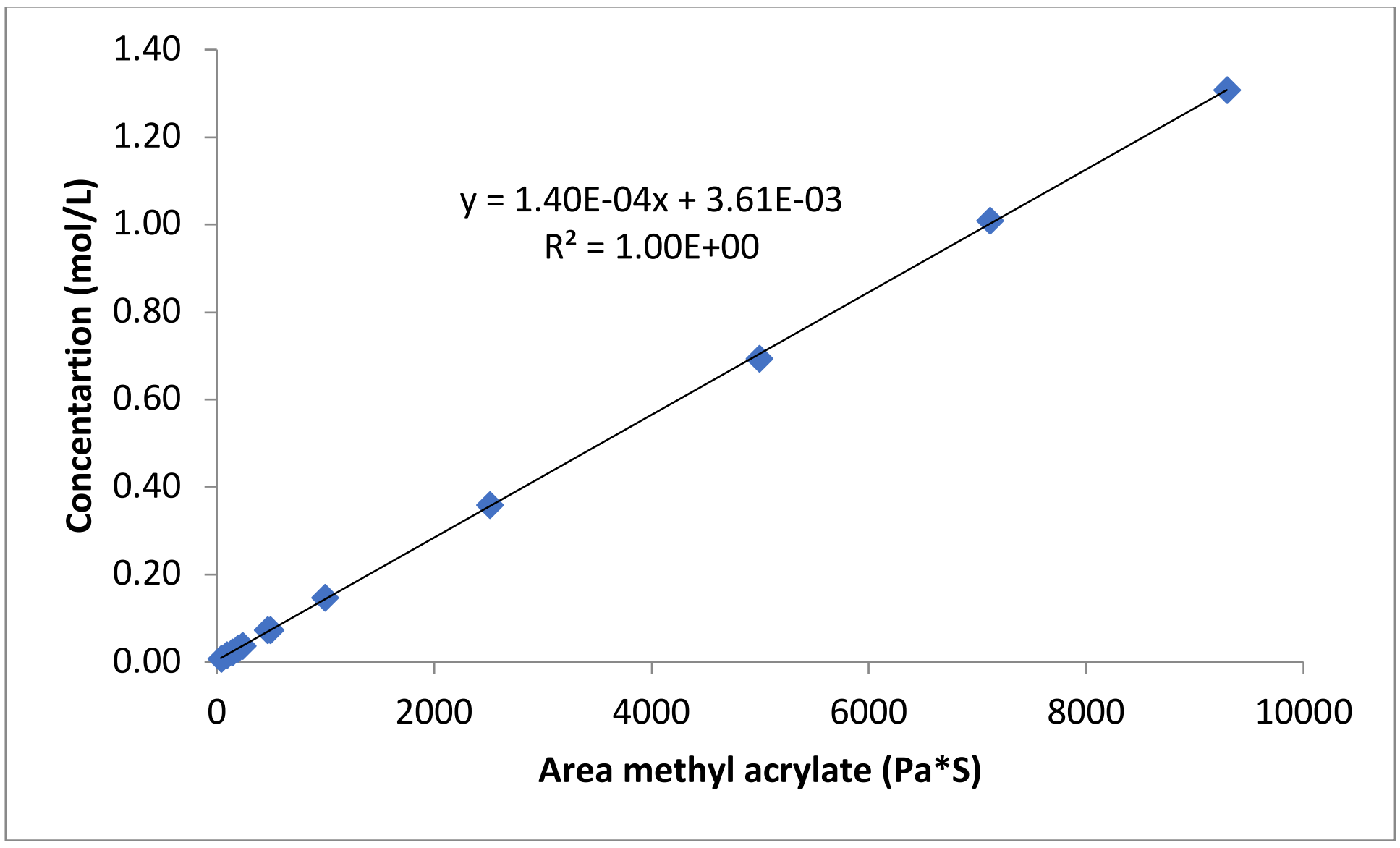

*Figure S12. GC calibration for the Diels-Alder reaction starting material (methyl acrylate **5**).*

2. 3. 2. Experiments utilising dispersion-generated concentration gradient method

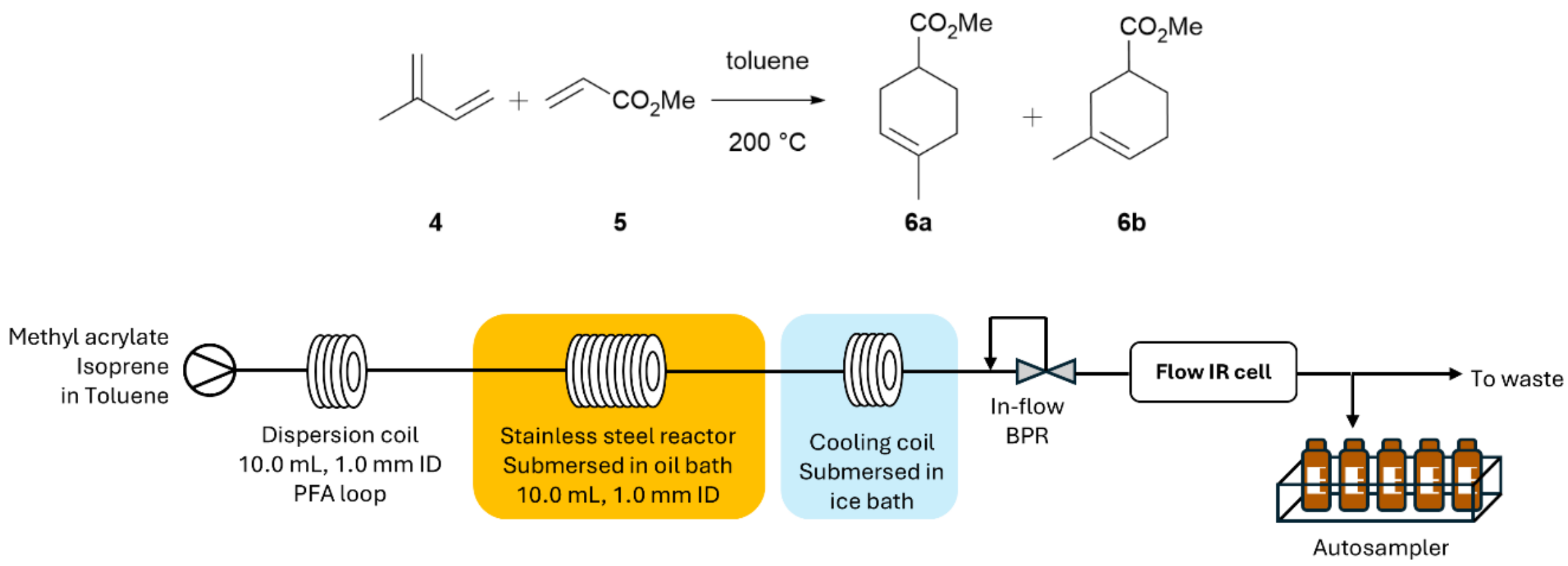

*Figure S13. Top: Scheme of Diels-Alder reaction of isoprene **4** and methyl acrylate **5**. Bottom: Flow setup used in dispersion-generated concentration gradient experiments of Diels-Alder reaction.*

In a RBF flushed with nitrogen, methyl acrylate (7.20 mL, 80.0 mmol, 1.0 eq., 3.17 M solution) and isoprene (8.00 mL, 80.0 mmol, 1.0 eq., 3.17 M solution) were mixed in toluene (10.0 mL). The reaction mixture was degassed by sonication whilst being saturated with nitrogen and was then connected to pump reagent stream. Vessel containing solvent (toluene) was connected to pump solvent stream. Flow system was composed of a piston pump (Vapourtec R2 unit) delivering solvent or reaction mixture to the dispersion coil (PFA, 10.0 mL, 1.0 mm ID), tubular reactor (stainless steel, 10.0 mL, 1.0 mm ID), cooling coil (in ice bath), BPR (250 psi), in-line IR flow cell and autosampler. Solvent (toluene) was pumped at the 0.3 $^{mL}/_{min}$ flow rate, and the reactor was set to 200 °C. Once the temperature was stable, the feeding valve was switched to deliver the reagent solution and 5 mL of the reaction mixture was injected. At the end of the sample plug injection, the feeding valve was returned to the solvent feed. The sample plug dispersed first in the 10 mL PFA loop and then the reaction occurred in the heated stainless-steel reactor. In-line IR detection was used, and the data sampling rate was determined by the acquisition parameters. The spectra were processed using BORIS software by calibration-less methods (OPA or height of non-overlapping peaks processing). When indicated, the

reaction mixture was collected in vials for GC analysis. GC samples were obtained by collection of 0.2 mL of reaction mixture into 1.0 mL of DCM.

2. 4. Heck coupling between iodobenzene and butyl acrylate

2. 4. 1. GC calibration of *n*-butyl cinnamate

Samples for GC were prepared containing following amounts of veratrole (internal standard) and *n*-butyl cinnamate (product) in 1.5 mL of solvent (Table S6). Sample GC trace from Heck reaction studies and GC calibration curve are presented below (Figure S14 and Figure S15, respectively).

Table S6. Table of samples used for calibration of GC for Heck reaction product quantification.

Sample	Mass of product [g]	Mass of internal standard [g]	Moles of product [mol]	Moles of internal standard [mol]	GC Area of 5.41 min peak (product)	GC Area of 3.04 min peak (product)	Area ratio of product/IS	Mole ratio of product/IS
1	0.01305	0.01039	$6.39\cdot10^{-5}$	$7.52\cdot10^{-5}$	1995.4	1414.9	1.410276	0.849522
2	0.00353	0.01065	$1.73\cdot10^{-5}$	$7.71\cdot10^{-5}$	489.7	1259.6	0.388774	0.224184
3	0.01433	0.00189	$7.02\cdot10^{-5}$	$1.37\cdot10^{-5}$	1864.6	230.3	8.096396	5.128192
4	0.00378	0.00998	$1.85\cdot10^{-5}$	$7.22\cdot10^{-5}$	495.4	1135.6	0.436245	0.256178
5	0.01277	0.00632	$6.25\cdot10^{-5}$	$4.57\cdot10^{-5}$	1719.5	746.1	2.304651	1.366639

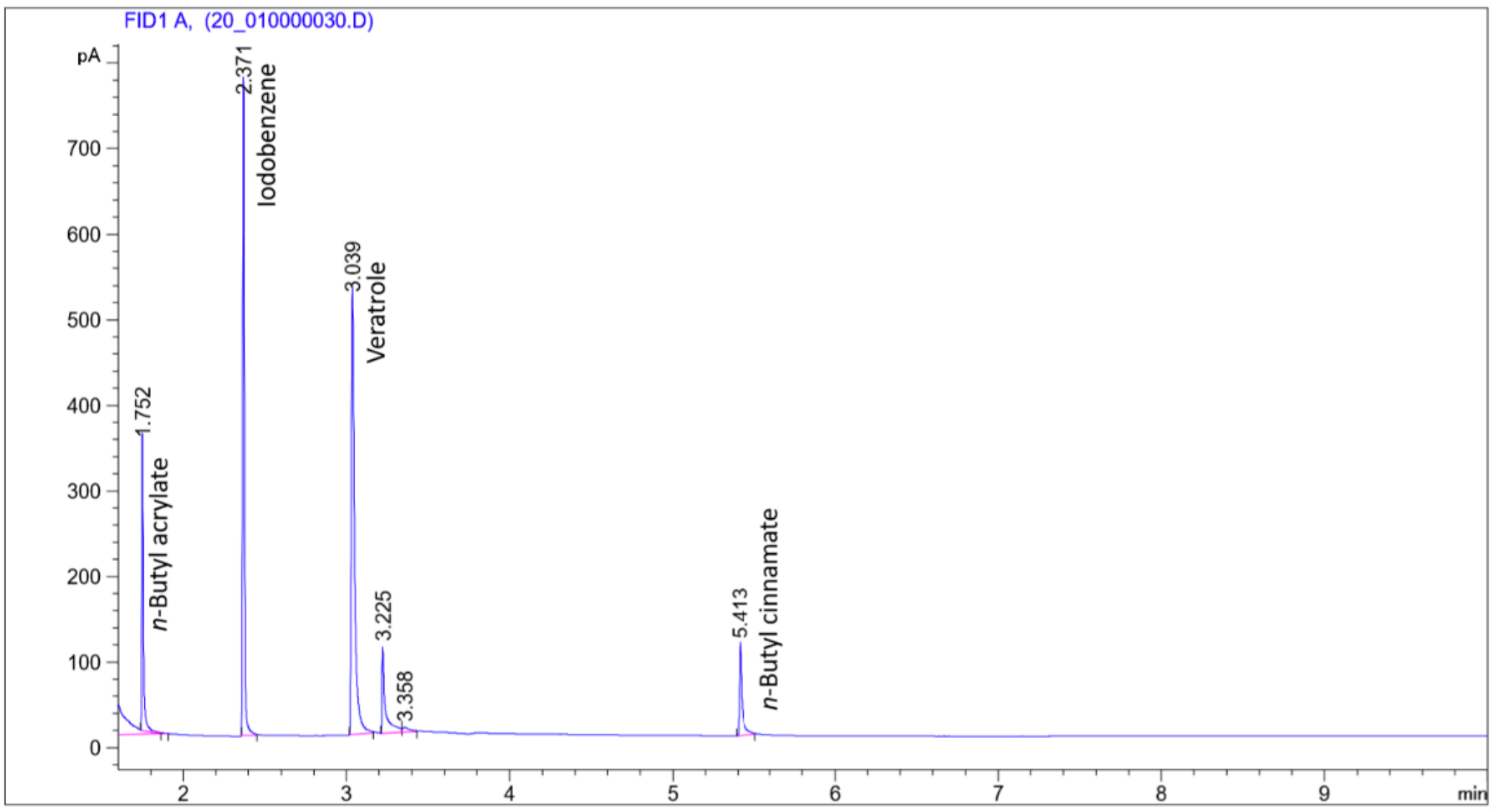

Figure S14. Example GC trace from Heck coupling reaction.

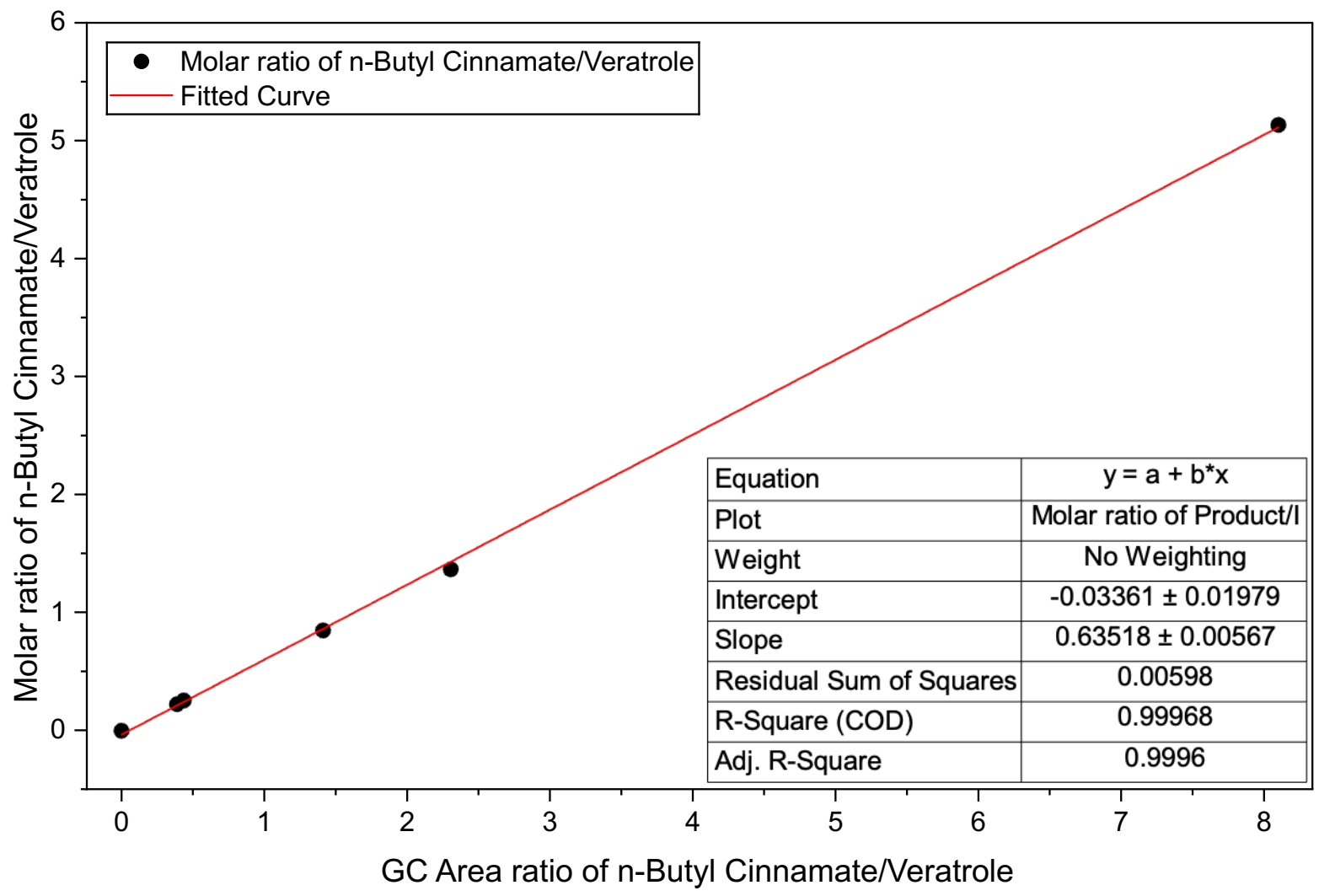

Figure S15. GC calibration curve for Heck reaction product concentration calculation.

2. 4. 2. Experimental protocol for flow studies utilising dispersion-generated concentration gradient method in Heck coupling reaction, example procedure of palladium diacetate dispersion

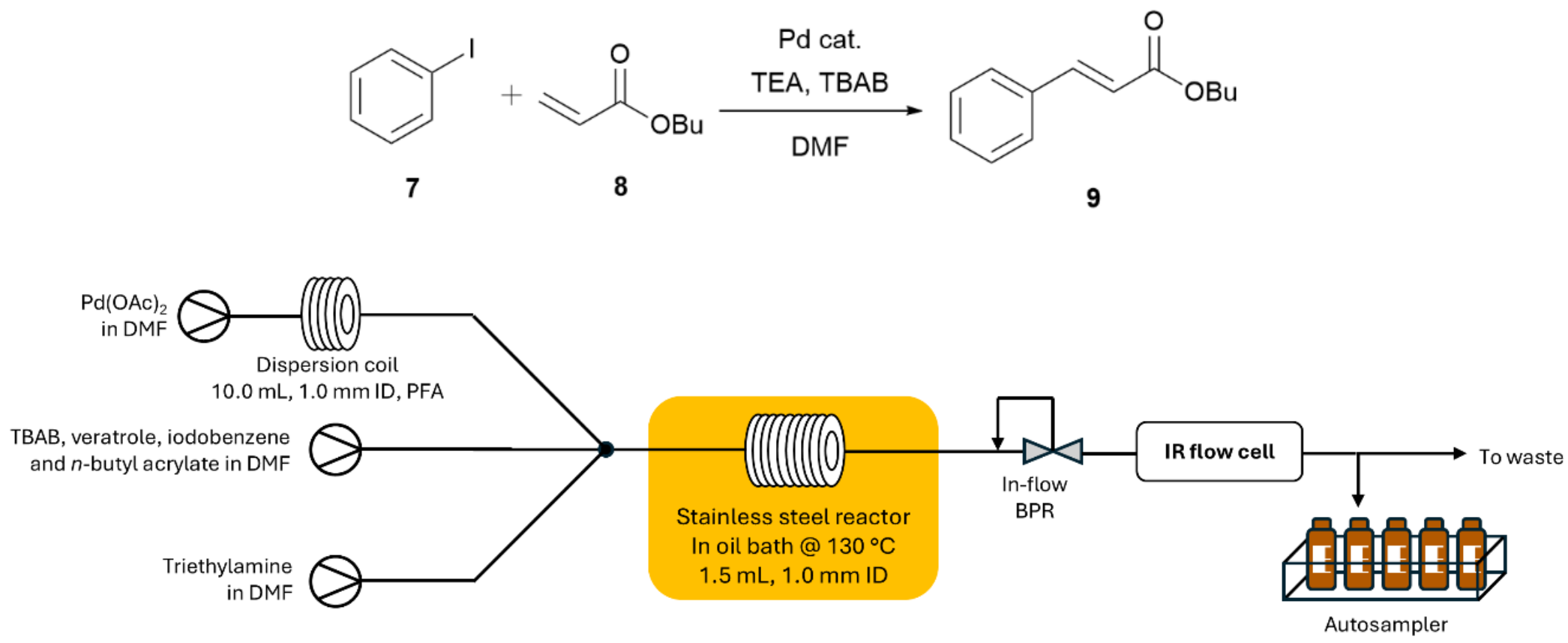

Figure S16. Top: Scheme of Heck coupling reaction of iodobenzene 7 and n-butyl acrylate 8. Bottom: Flow setup used in dispersion-generated concentration gradient experiments of Heck coupling, on example of $Pd(OAc)_2$ dispersion experiment.

$Pd(OAc)_2$ (7.55 mg, 0.034 mmol, 0.45 mol%) was dissolved in DMF (15.0 mL) and obtained mixture was connected to the 1st dispersed reagent flow stream. Veratrole (internal standard, 0.96 mL, 1.04 g, 7.53 mmol, 1.0 eq.), TBAB (2.41 g, 7.48 mmol, 1.0 eq.), iodobenzene (1.08 mL, 1.94 g, 9.68 mmol, 1.3 eq.), and *n*-butyl acrylate (1.07 mL, 0.97 g, 7.53 mmol, 1.0 eq.) were dissolved in DMF (15.0 mL) and obtained mixture was connected to the 2nd reagent stream. Triethylamine (1.8 mL, 1.31 g, 12.91 mmol, 1.72 eq.) was dissolved in DMF (15.0 mL) and obtained solution was connected to 3rd reagent stream. DMF was connected to all solvent lines. Flow system was composed of a piston pump (Vapourtec R2 units) delivering dispersed reagent to the dispersion coil (PFA, 10.0 mL, 1.0 mm ID), then joined with other reagent streams and directed to tubular reactor (stainless steel, 1.5 mL, 1.0 mm ID, in oil bath at 130 °C), BPR (250 psi), in-line IR flow cell and autosampler. Solvent was initially pumped at all lines at the 0.1 $^{mL}/_{min}$ flow rate until reactor temperature equilibrated. Feeding valves were then switched to reagent lines and 15.0 mL plugs of each solution were pumped through the system. Dispersed reagent plug was infused into the flow system earlier in time wrt to steady-state reagents to allow for the plug to be dispersed and then joined with other reaction components – in this case 2nd and 3rd

feeding valves had a delay of 47 min (4.7 mL each) before switching to reagents lines. At the end of the reagents plugs injection, the feeding valves were returned to the solvent feed.

Reaction progress was monitored by in-line IR and off-line GC measurements. GC samples were prepared by acquisition of 0.2 mL of reaction mixture and further dilution in 1.0 mL of acetone. The earlier acquired dispersion profile was then overlaid with the acquired IR and/or GC data allowing for calculation of the $Pd(OAc)_2$ concentration during the experiment.

2. 4. 3. Results of dispersion-generated concentration gradient method in Heck coupling reaction, GC and IR traces of process components, dispersion profiles, and yield as a function of dispersed component concentration

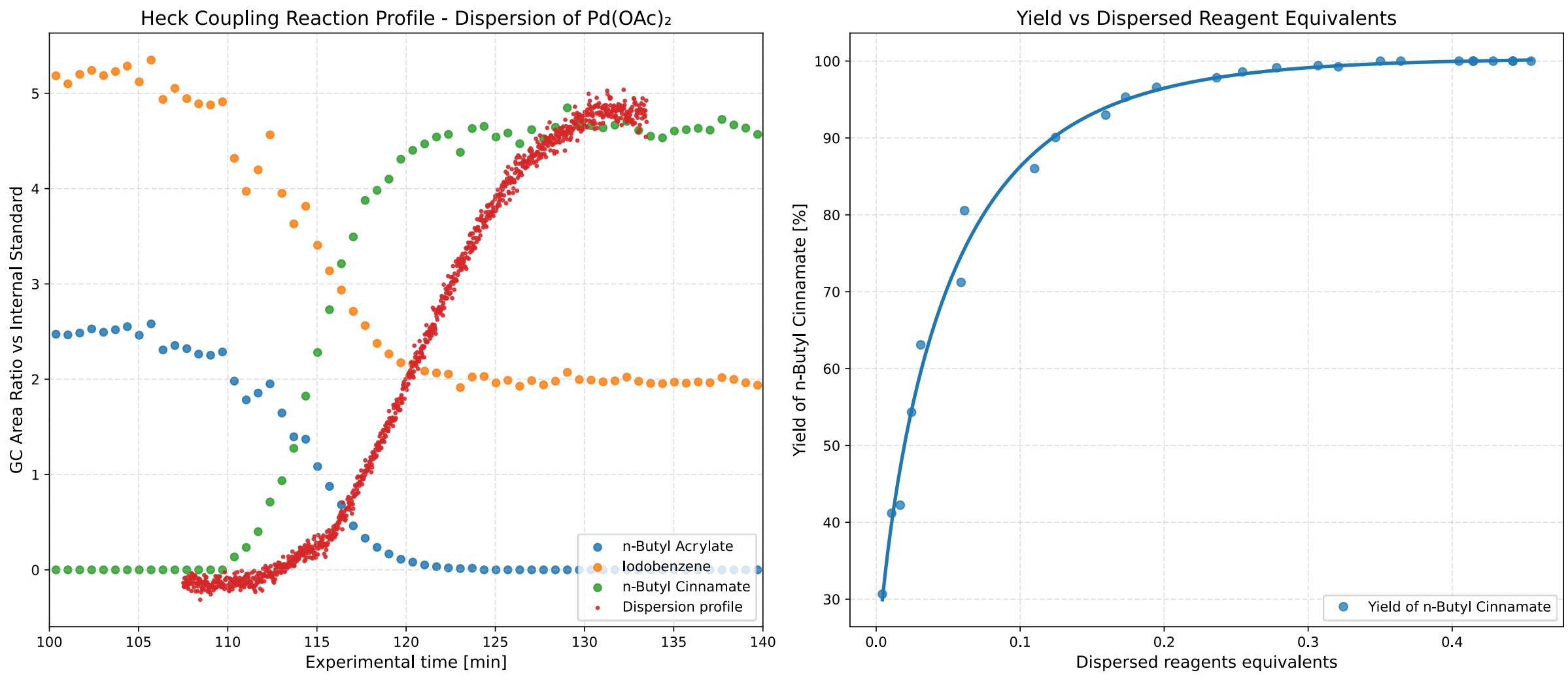

Figure S17. Dispersion generated concentration gradient of $Pd(OAc)_2$. Left: GC traces of reaction components and dispersion profile of dispersed reagent. Right: Effect of $Pd(OAc)_2$ concentration on n-butyl cinnamate yield. Fitted line added for visual representation of the trend only.

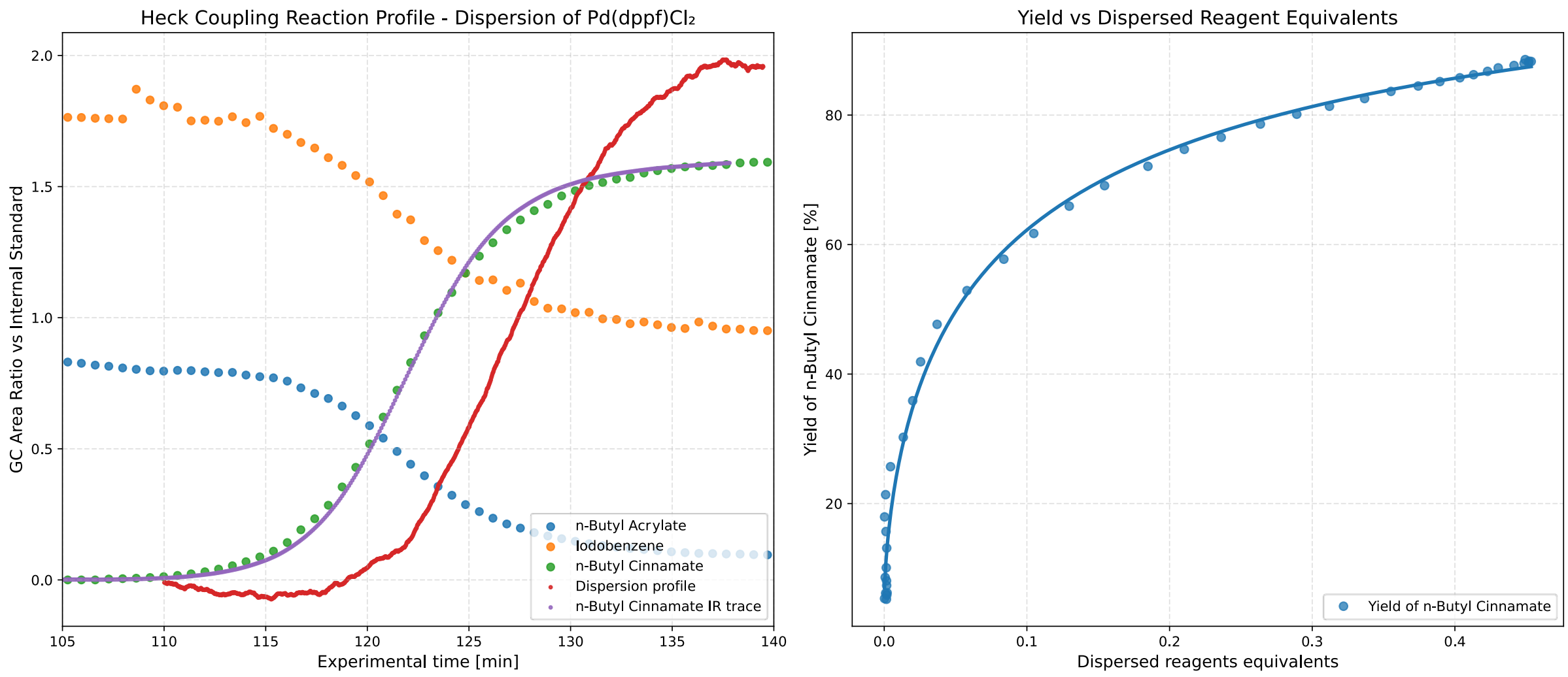

Figure S18. Dispersion generated concentration gradient of $Pd(dppf)Cl_2$. Left: GC and IR traces of reaction components and dispersion profile of dispersed reagent. Right: Effect of $Pd(dppf)Cl_2$ concentration on n-butyl cinnamate yield. Fitted line added for visual representation of the trend only.

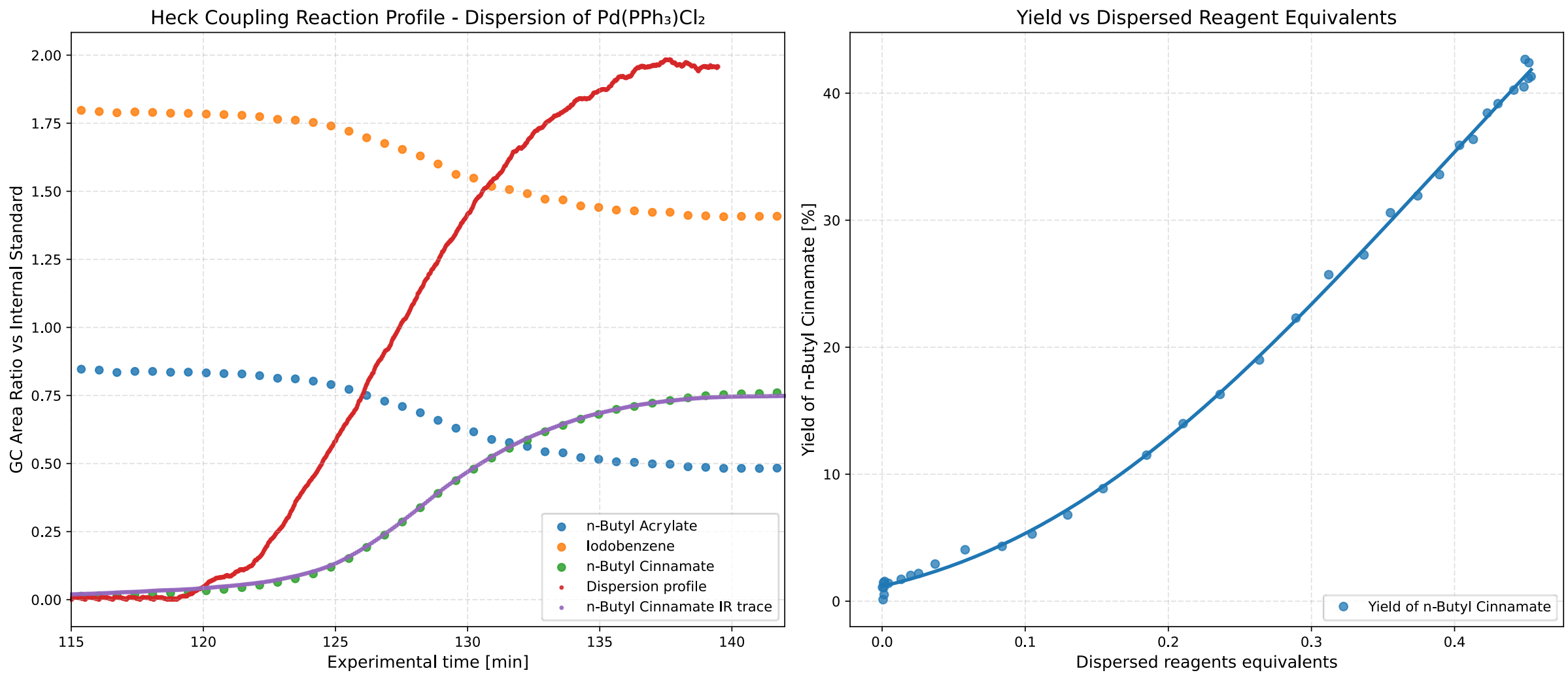

Figure S19. Dispersion generated concentration gradient of $Pd(PPh_3)Cl_2$. Left: GC and IR traces of reaction components and dispersion profile of dispersed reagent. Right: Effect of $Pd(PPh_3)Cl_2$ concentration on n-butyl cinnamate yield. Fitted line added for visual representation of the trend only.

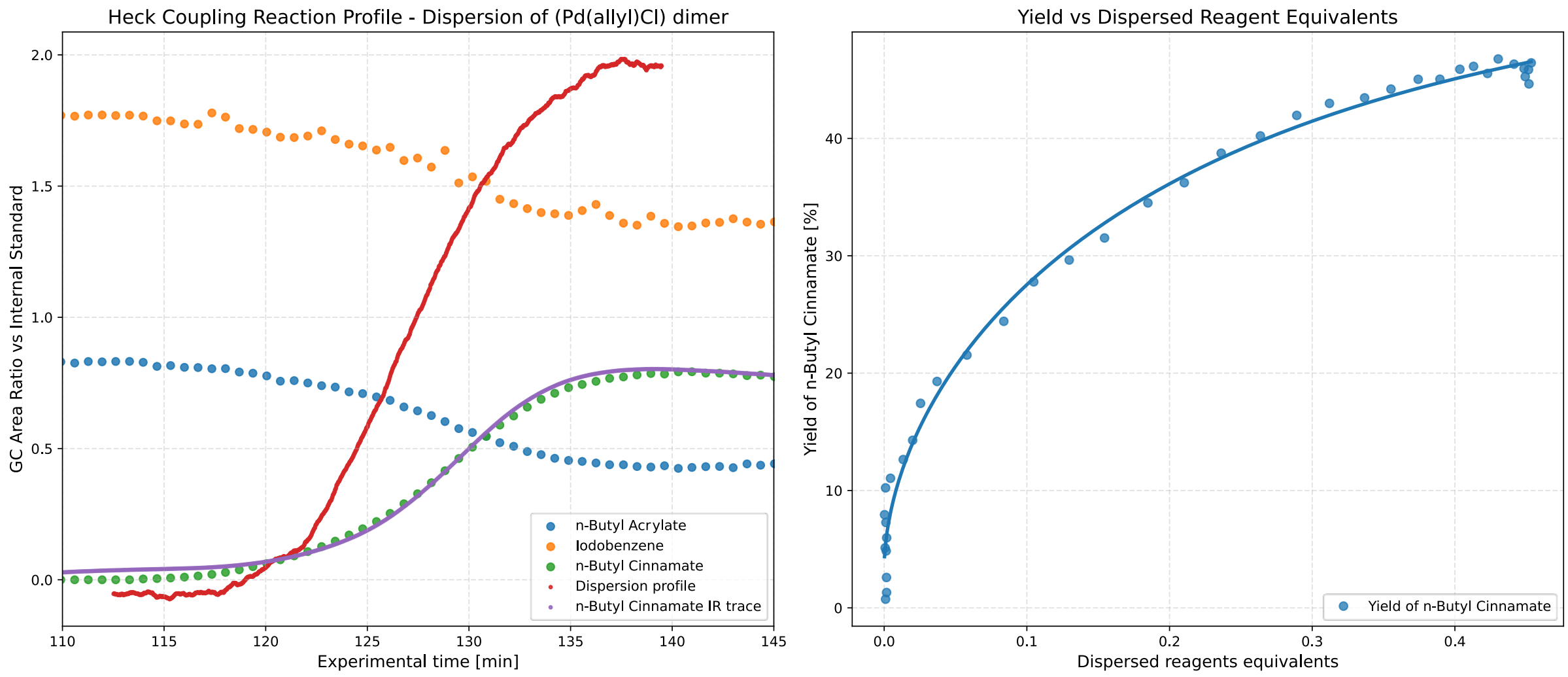

Figure S20. Dispersion generated concentration gradient of Pd(allyl)Cl dimer. Left: GC and IR traces of reaction components and dispersion profile of dispersed reagent. Right: Effect of Pd(allyl)Cl dimer concentration on n-butyl cinnamate yield. Fitted line added for visual representation of the trend only.

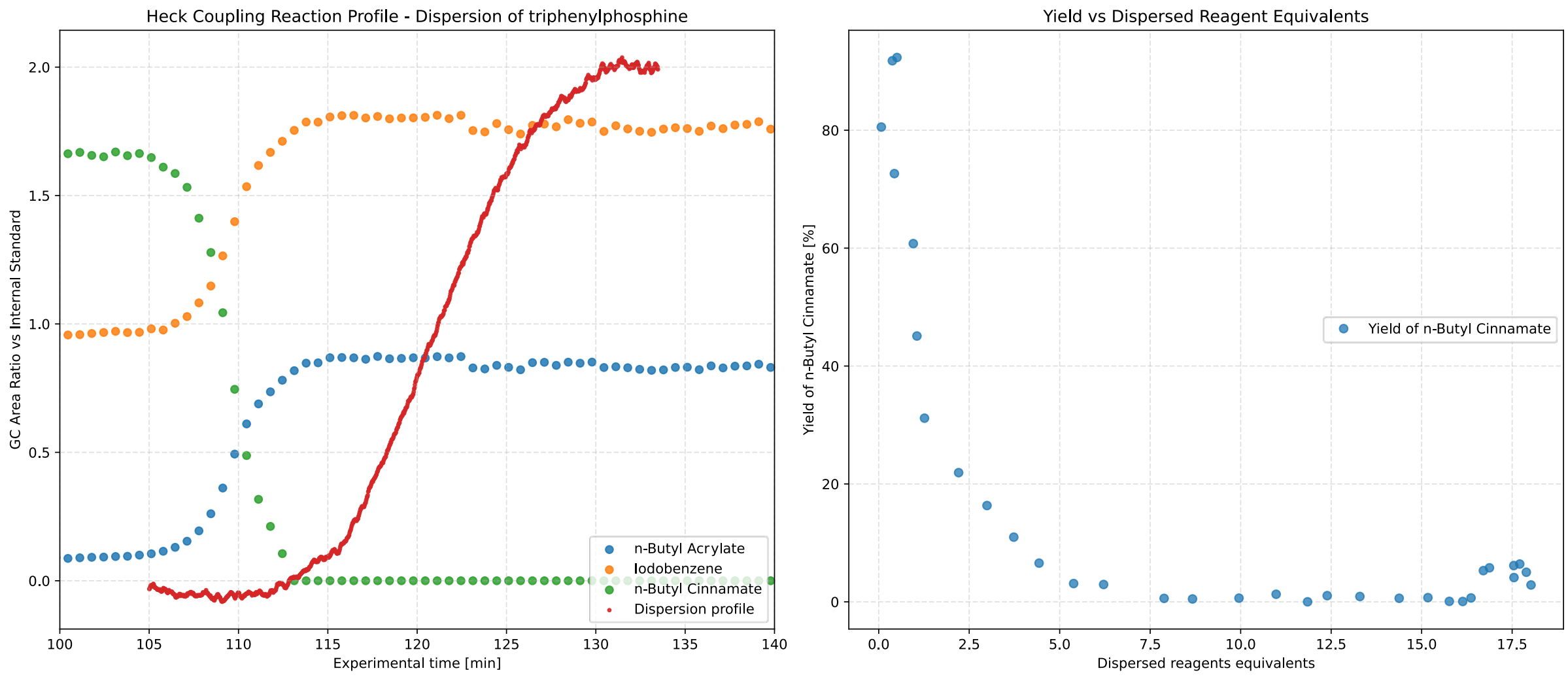

Figure S21. Dispersion generated concentration gradient of triphenylphosphine. Left: GC traces of reaction components and dispersion profile of dispersed reagent. Right: Effect of triphenylphosphine concentration on n-butyl cinnamate yield.

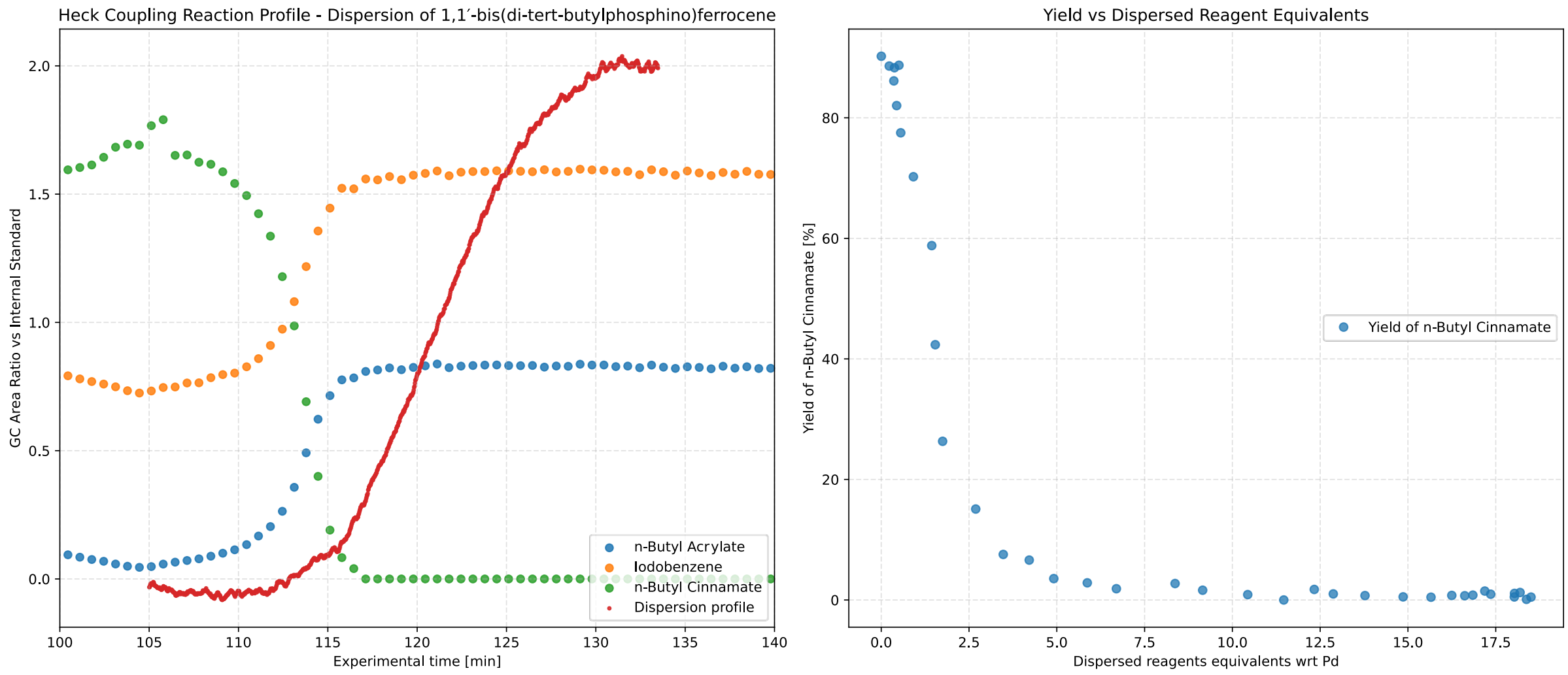

Figure S22. Dispersion generated concentration gradient of dtbpf. Left: GC traces of reaction components and dispersion profile of dispersed reagent. Right: Effect of dtbpf concentration on n-butyl cinnamate yield.

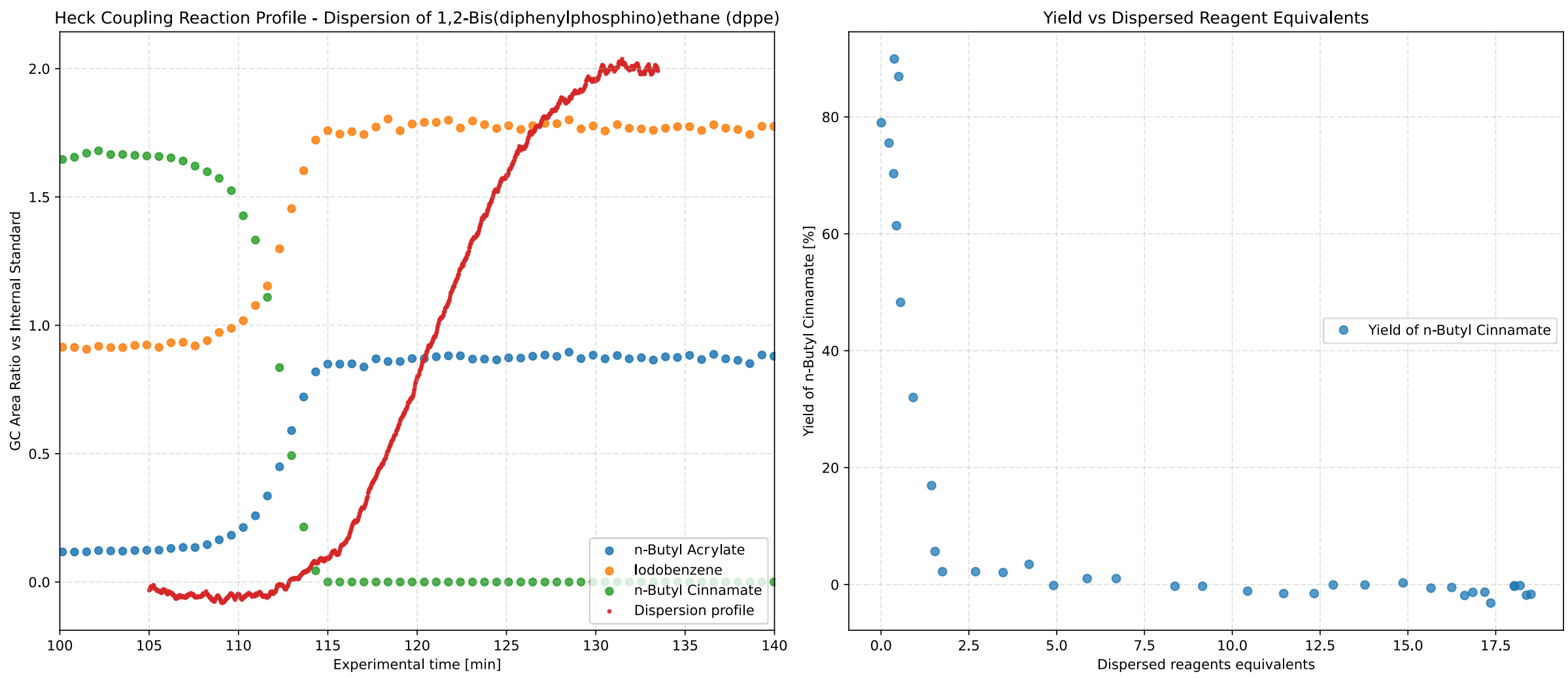

Figure S23. Dispersion generated concentration gradient of dppe. Left: GC traces of reaction components and dispersion profile of dispersed reagent. Right: Effect of dppe concentration on n-butyl cinnamate yield.

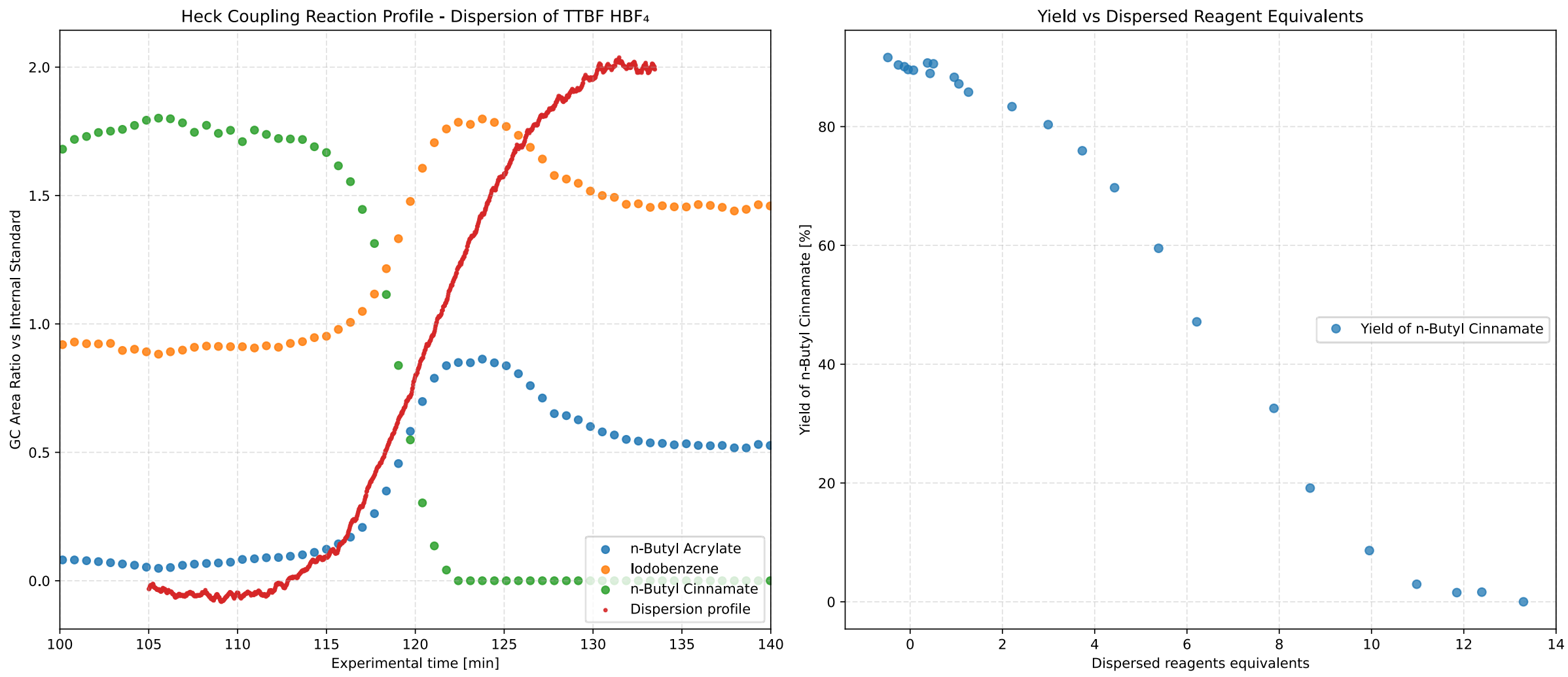

Figure S24. Dispersion generated concentration gradient of TTBF·HBF_4. Left: GC traces of reaction components and dispersion profile of dispersed reagent. Right: Effect of TTBF·HBF_4 concentration on n-butyl cinnamate yield.

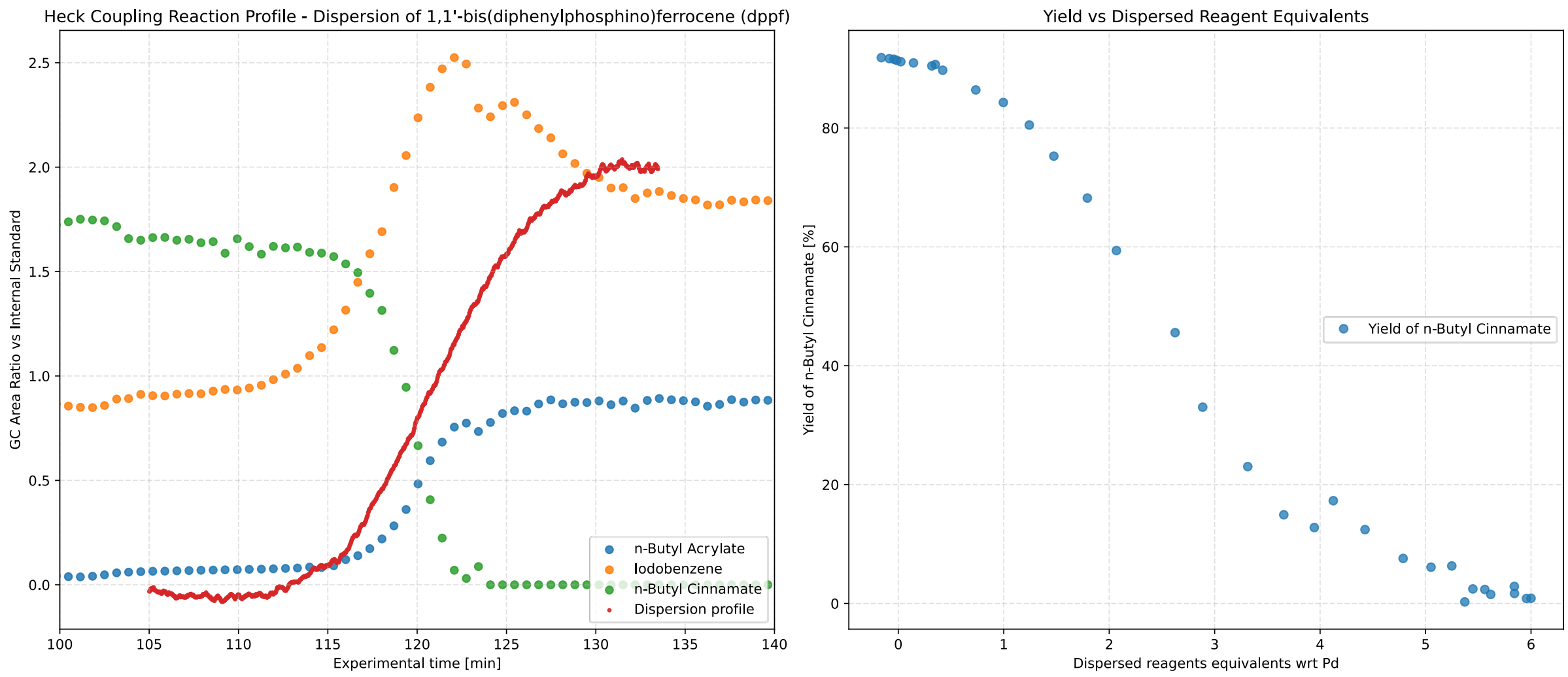

Figure S25. Dispersion generated concentration gradient of dppf. Left: GC traces of reaction components and dispersion profile of dispersed reagent. Right: Effect of dppf concentration on n-butyl cinnamate yield.

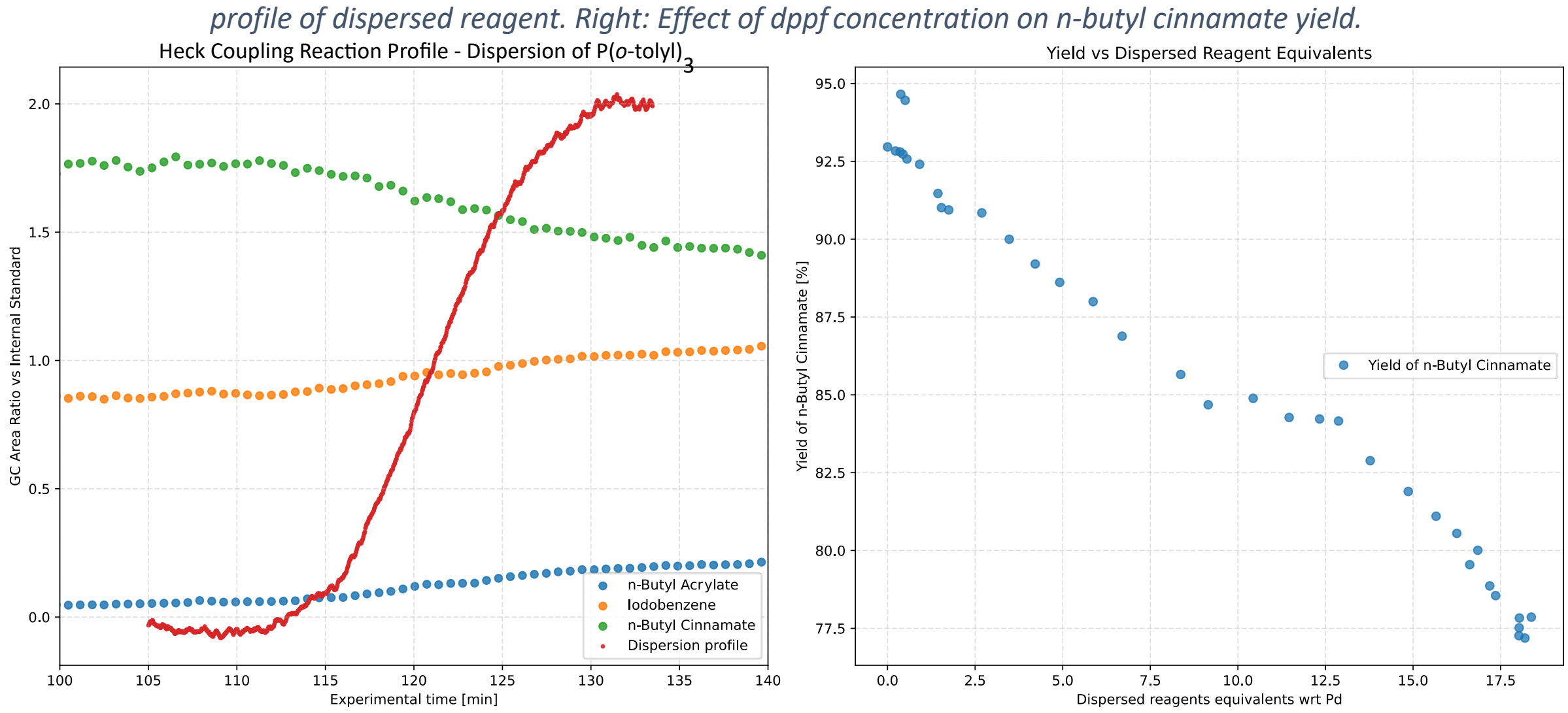

Figure S26. Dispersion generated concentration gradient of P(o-tolyl)$_3$. Left: GC traces of reaction components and dispersion profile of dispersed reagent. Right: Effect of P(o-tolyl)$_3$ concentration on n-butyl cinnamate yield.

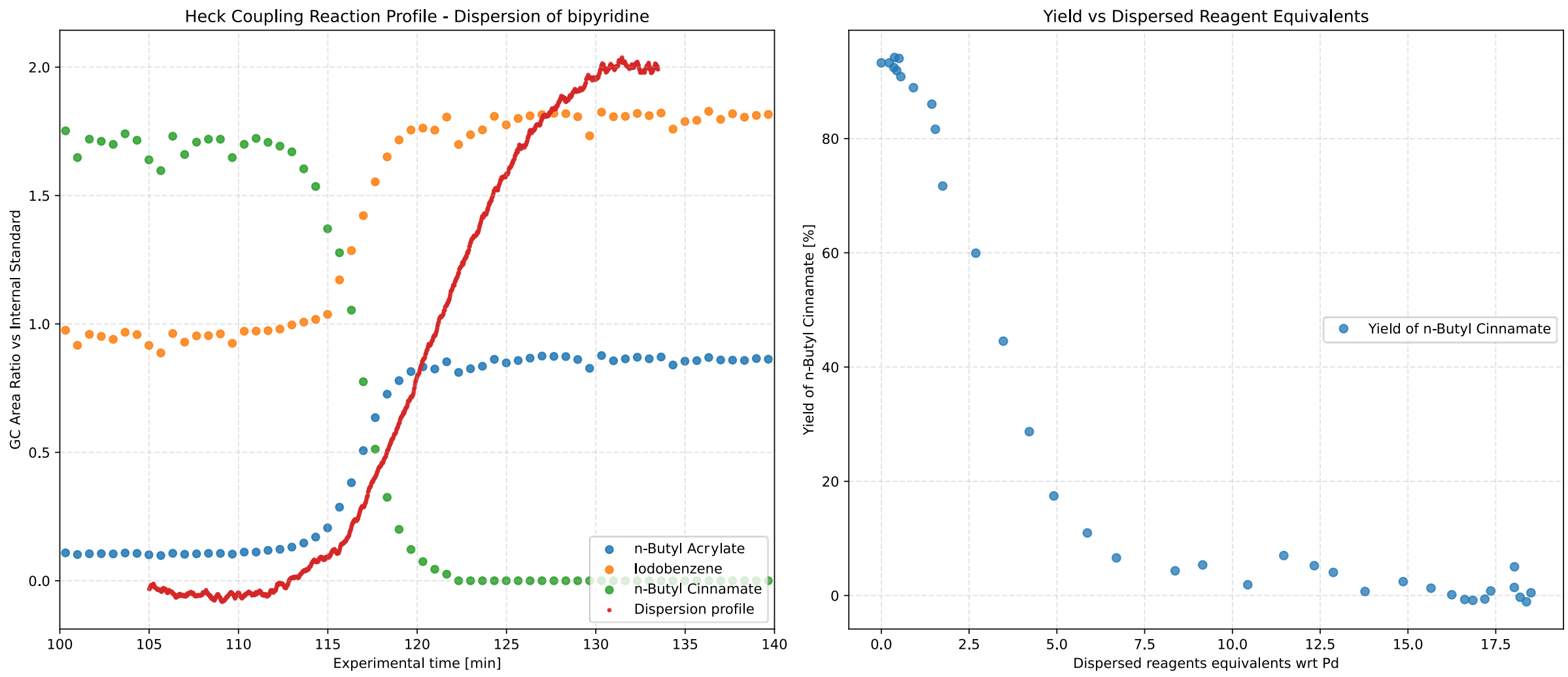

Figure S27. Dispersion generated concentration gradient of bipyridine. Left: GC traces of reaction components and dispersion profile of dispersed reagent. Right: Effect of bipyridine concentration on n-butyl cinnamate yield.

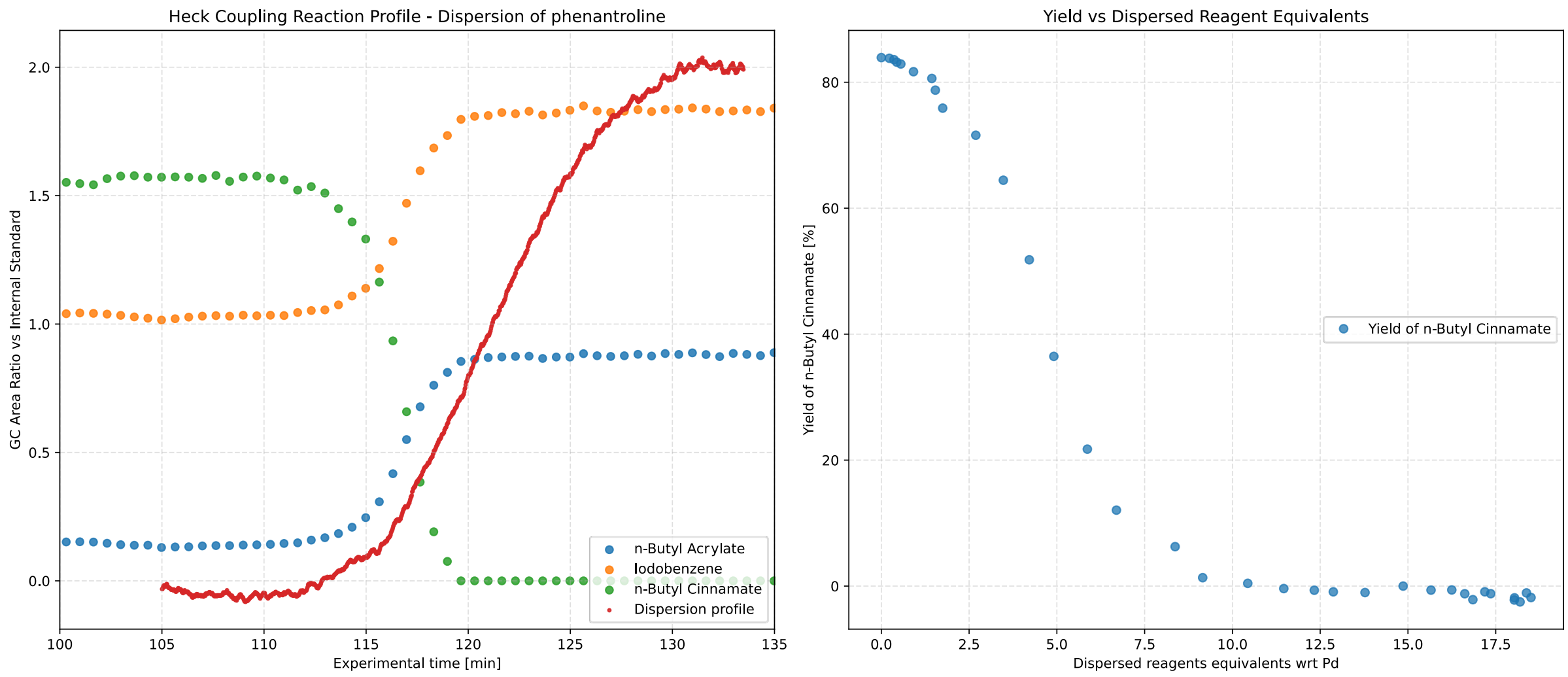

Figure S28. Dispersion generated concentration gradient of phenanthroline. Left: GC traces of reaction components and dispersion profile of dispersed reagent. Right: Effect of phenanthroline concentration on n-butyl cinnamate yield.

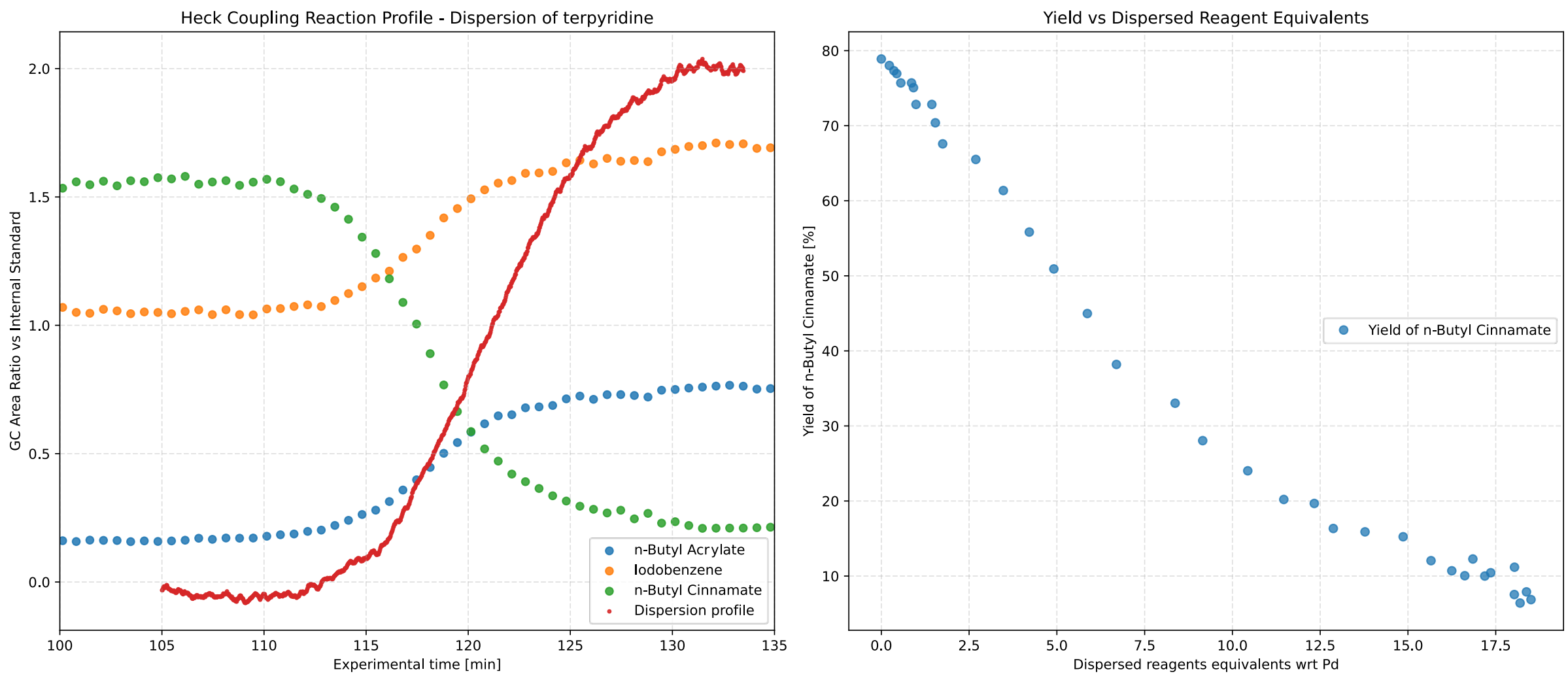

Figure S29. Dispersion generated concentration gradient of terpyridine. Left: GC traces of reaction components and dispersion profile of dispersed reagent. Right: Effect of terpyridine concentration on n-butyl cinnamate yield.

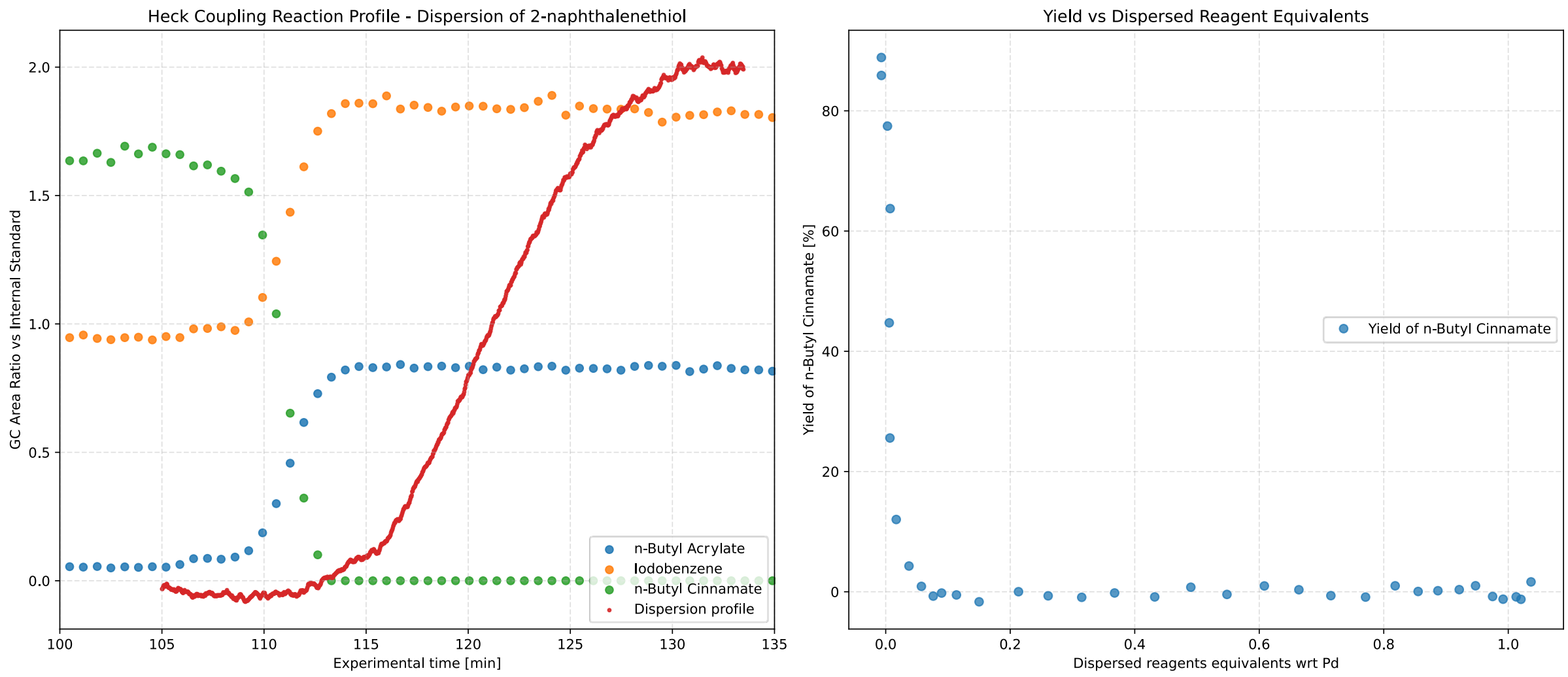

Figure S30. Dispersion generated concentration gradient of 2-naphthalenethiol. Left: GC traces of reaction components and dispersion profile of dispersed reagent. Right: Effect of 2-naphthalenethiol concentration on n-butyl cinnamate yield.

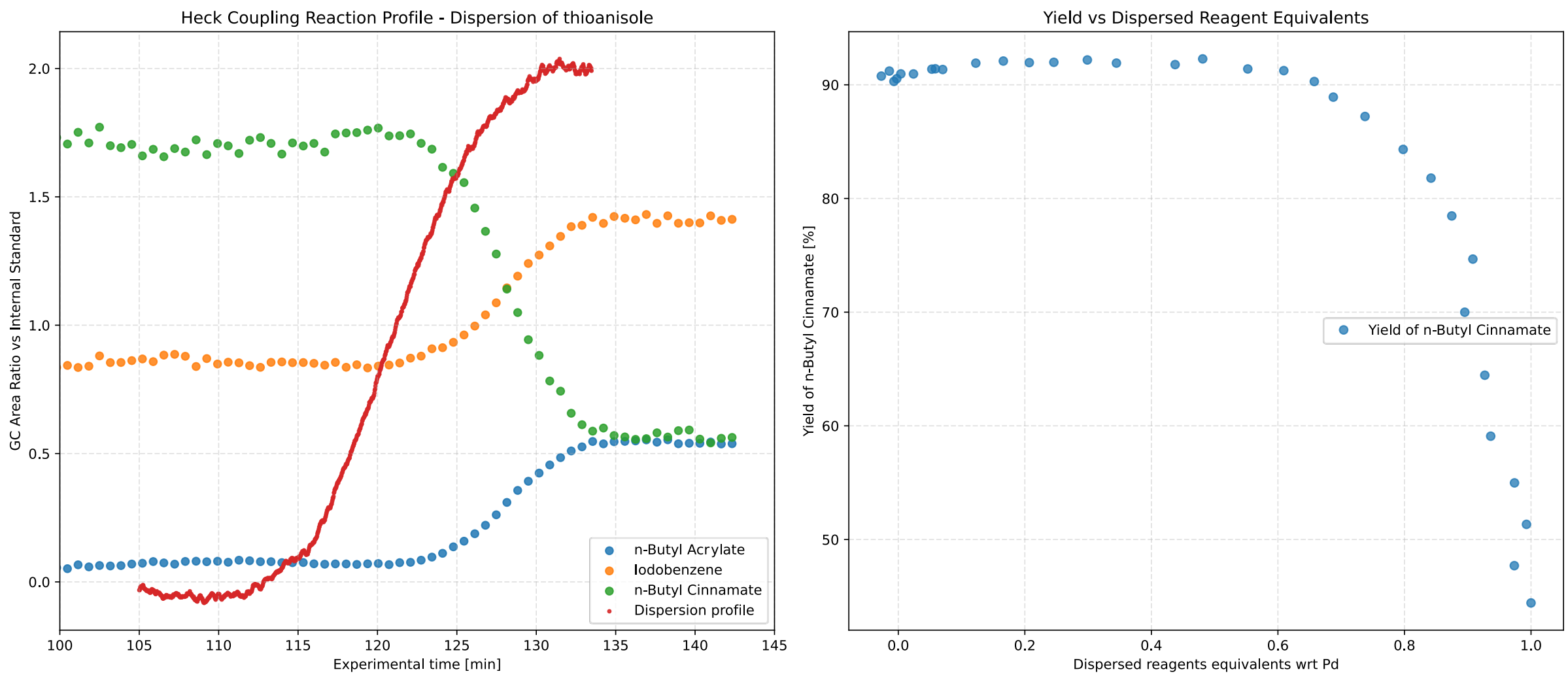

Figure S31. Dispersion generated concentration gradient of thioanisole. Left: GC traces of reaction components and dispersion profile of dispersed reagent. Right: Effect of thioanisole concentration on n-butyl cinnamate yield.

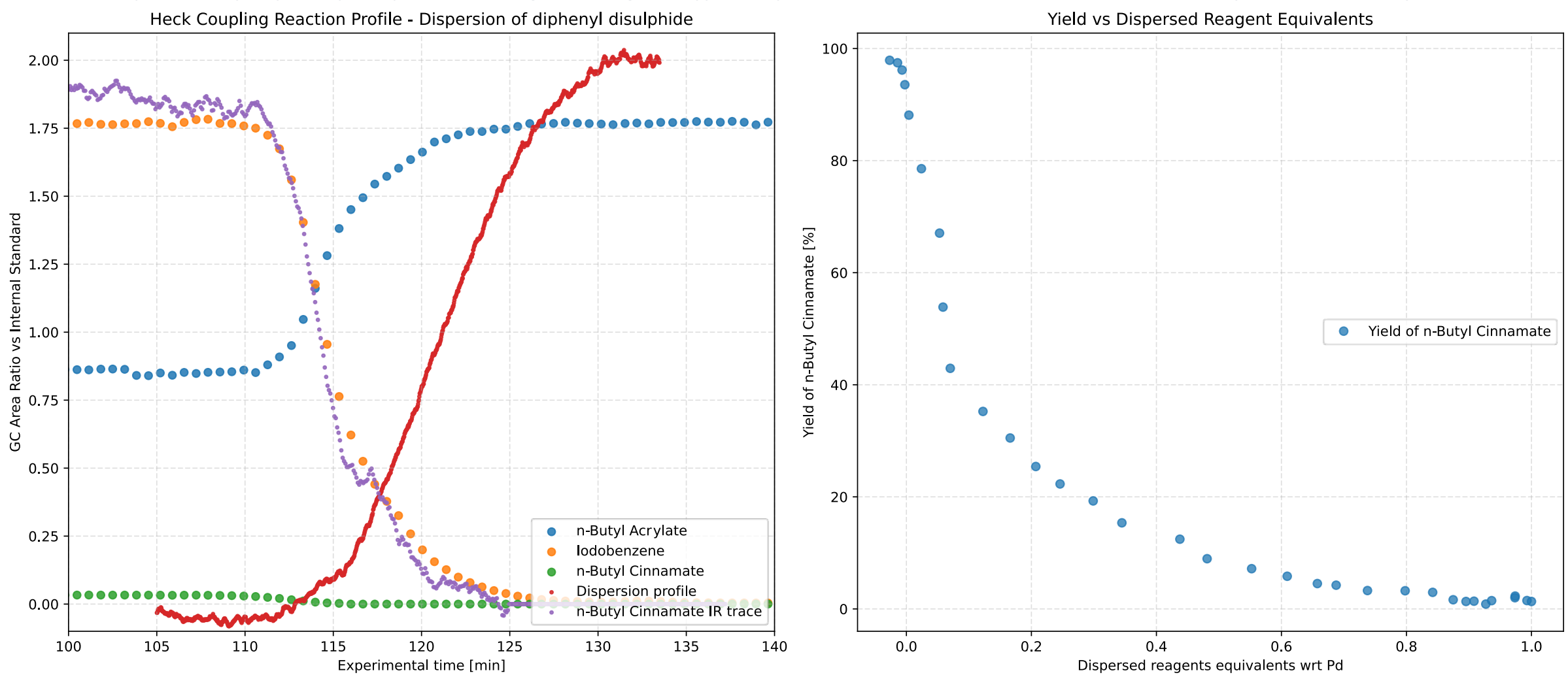

Figure S32. Dispersion generated concentration gradient of diphenyl disulphide. Left: GC and IR traces of reaction components and dispersion profile of dispersed reagent. Right: Effect of diphenyl disulphide concentration on n-butyl cinnamate yield.

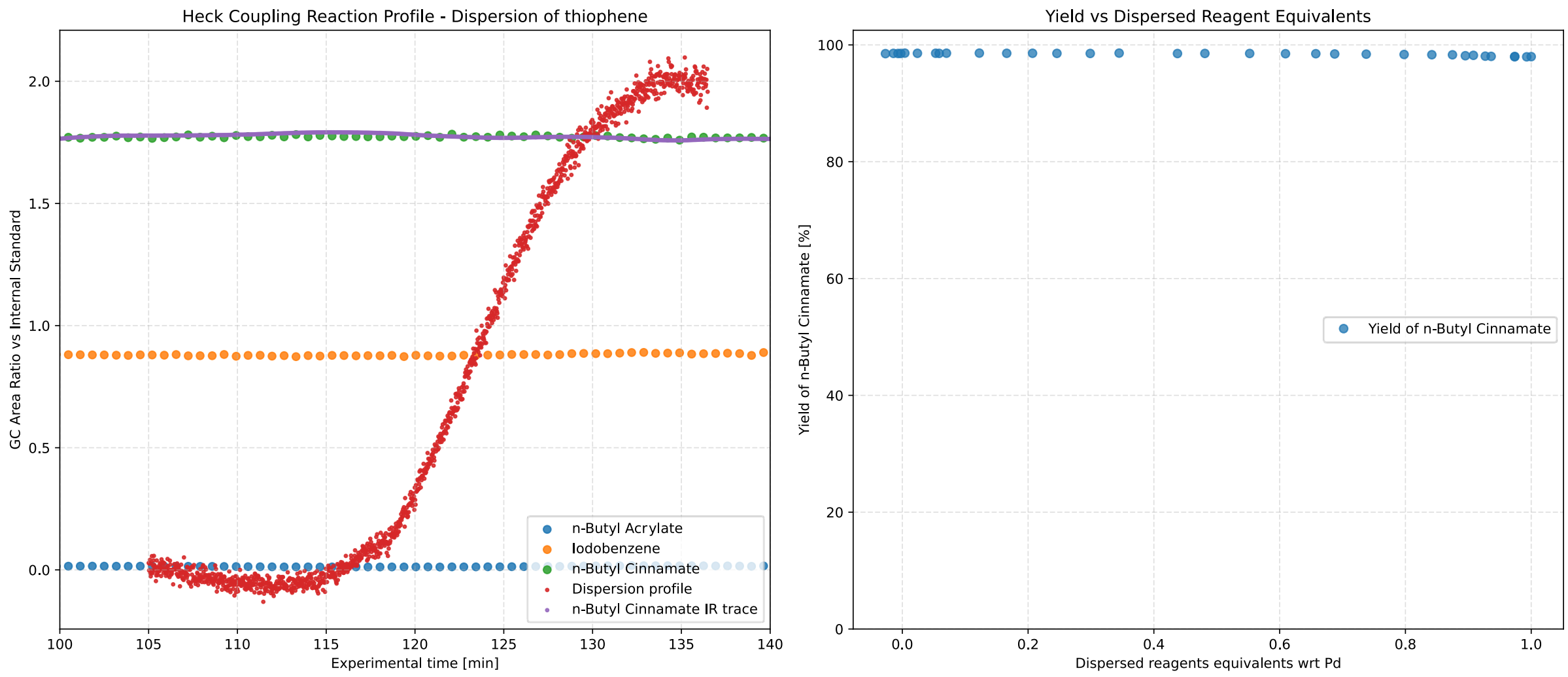

Figure S33. Dispersion generated concentration gradient of thiophene. Left: GC and IR traces of reaction components and dispersion profile of dispersed reagent. Right: Effect of thiophene concentration on n-butyl cinnamate yield.

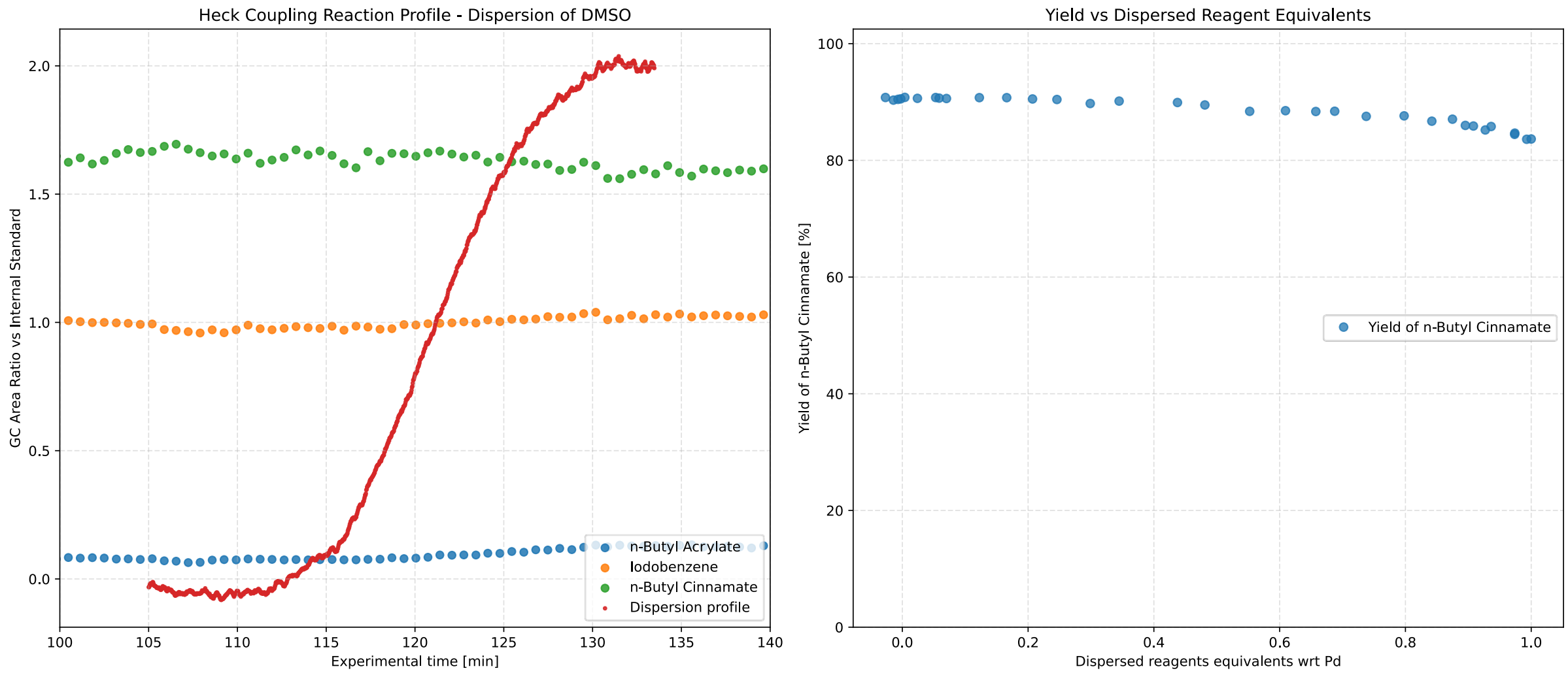

Figure S34. Dispersion generated concentration gradient of DMSO. Left: GC traces of reaction components and dispersion profile of dispersed reagent. Right: Effect of DMSO concentration on n-butyl cinnamate yield.

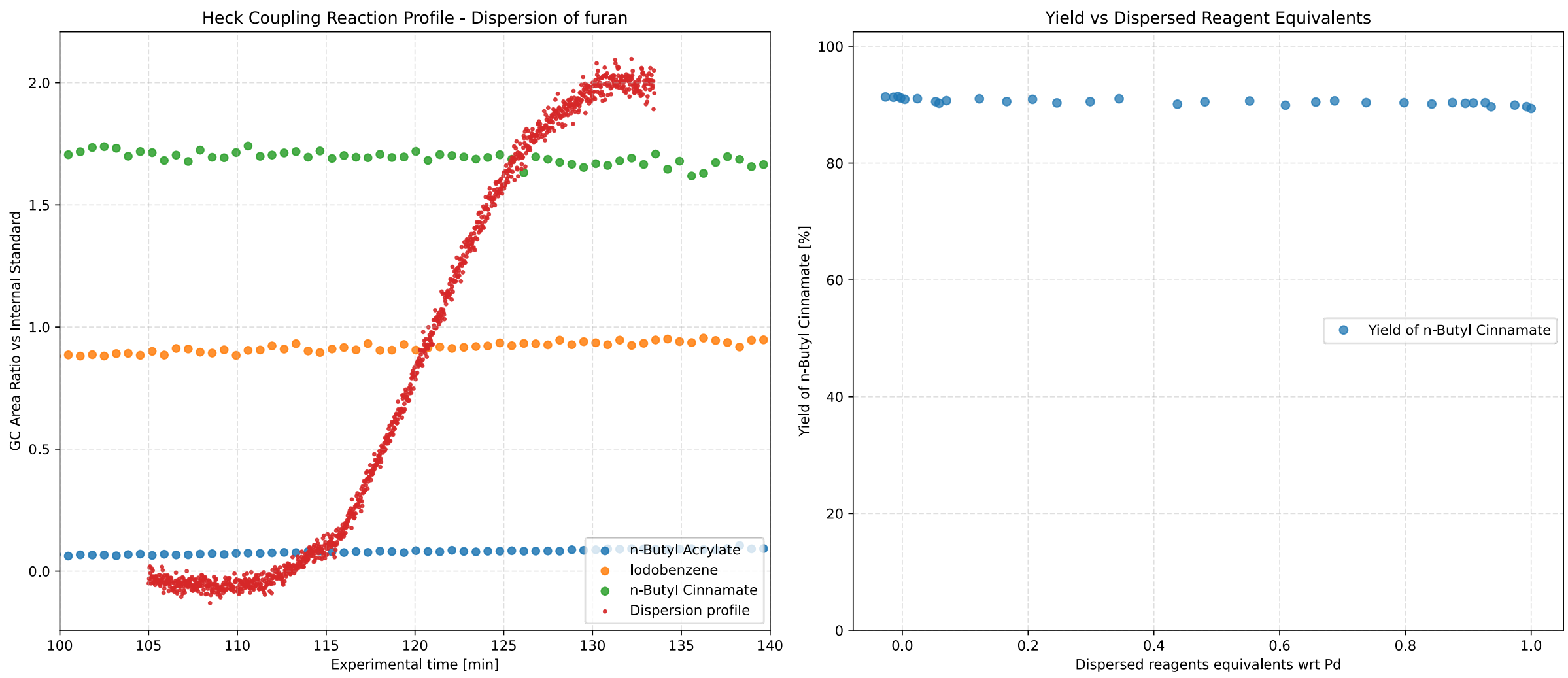

Figure S35. Dispersion generated concentration gradient of furan. Left: GC traces of reaction components and dispersion profile of dispersed reagent. Right: Effect of furan concentration on n-butyl cinnamate yield.

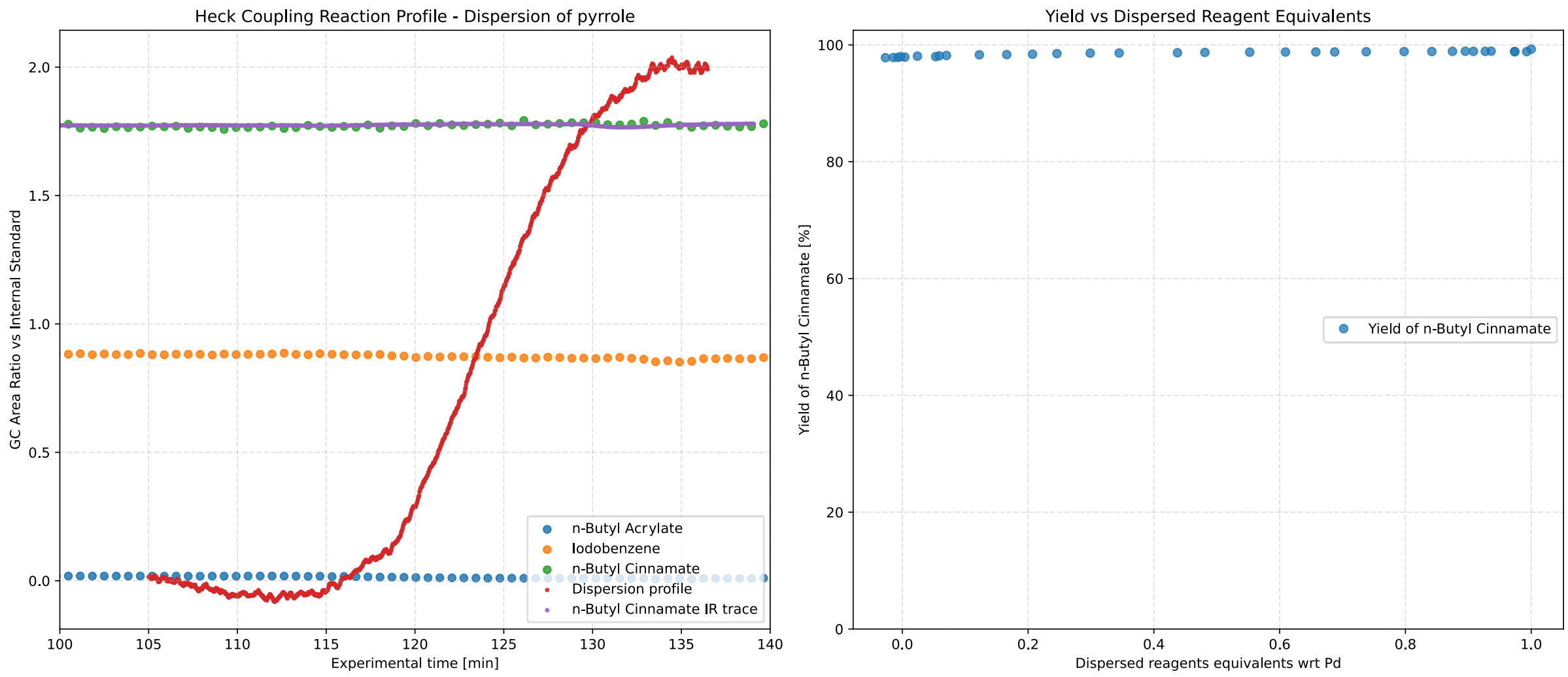

Figure S36. Dispersion generated concentration gradient of pyrrole. Left: GC traces of reaction components and dispersion profile of dispersed reagent. Right: Effect of pyrrole concentration on n-butyl cinnamate yield.

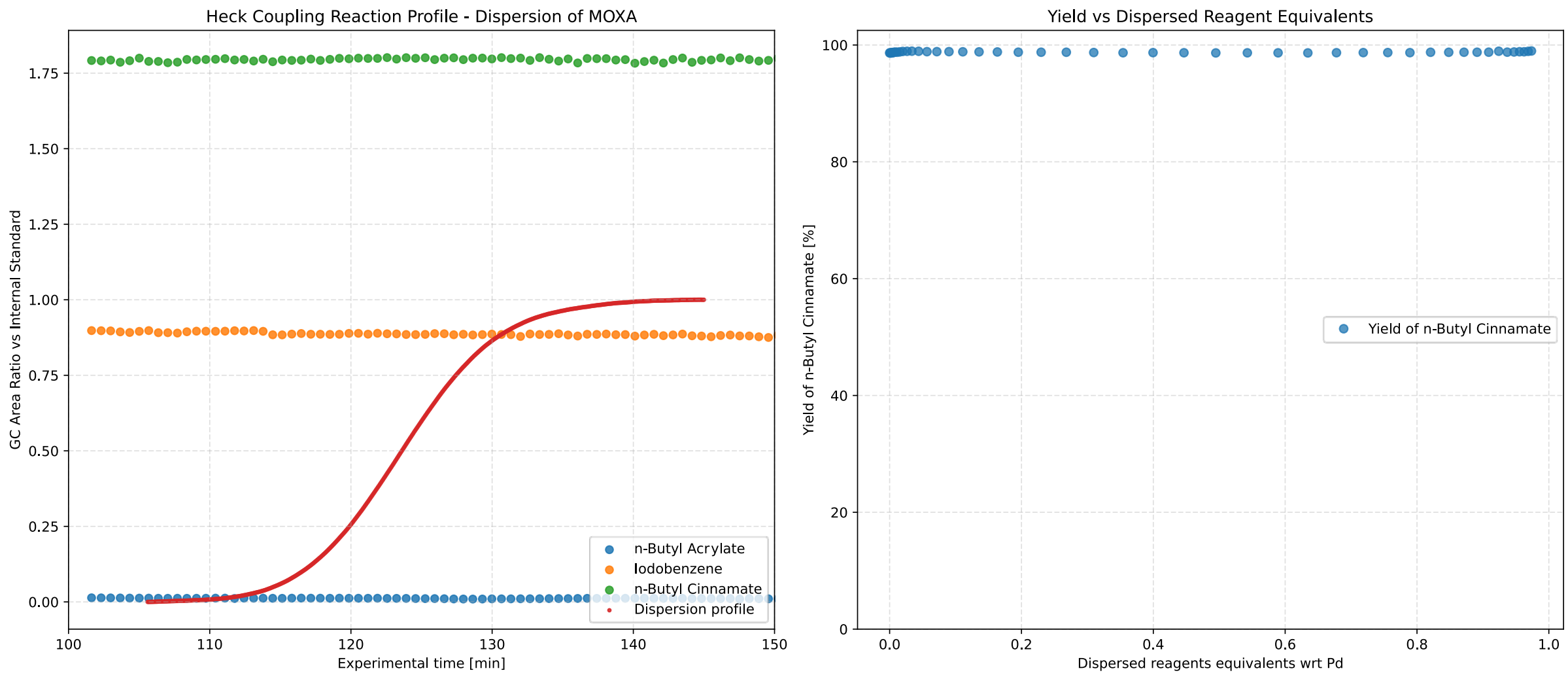

Figure S37. Dispersion generated concentration gradient of MOXA. Left: GC traces of reaction components and dispersion profile of dispersed reagent. Right: Effect of MOXA concentration on n-butyl cinnamate yield.

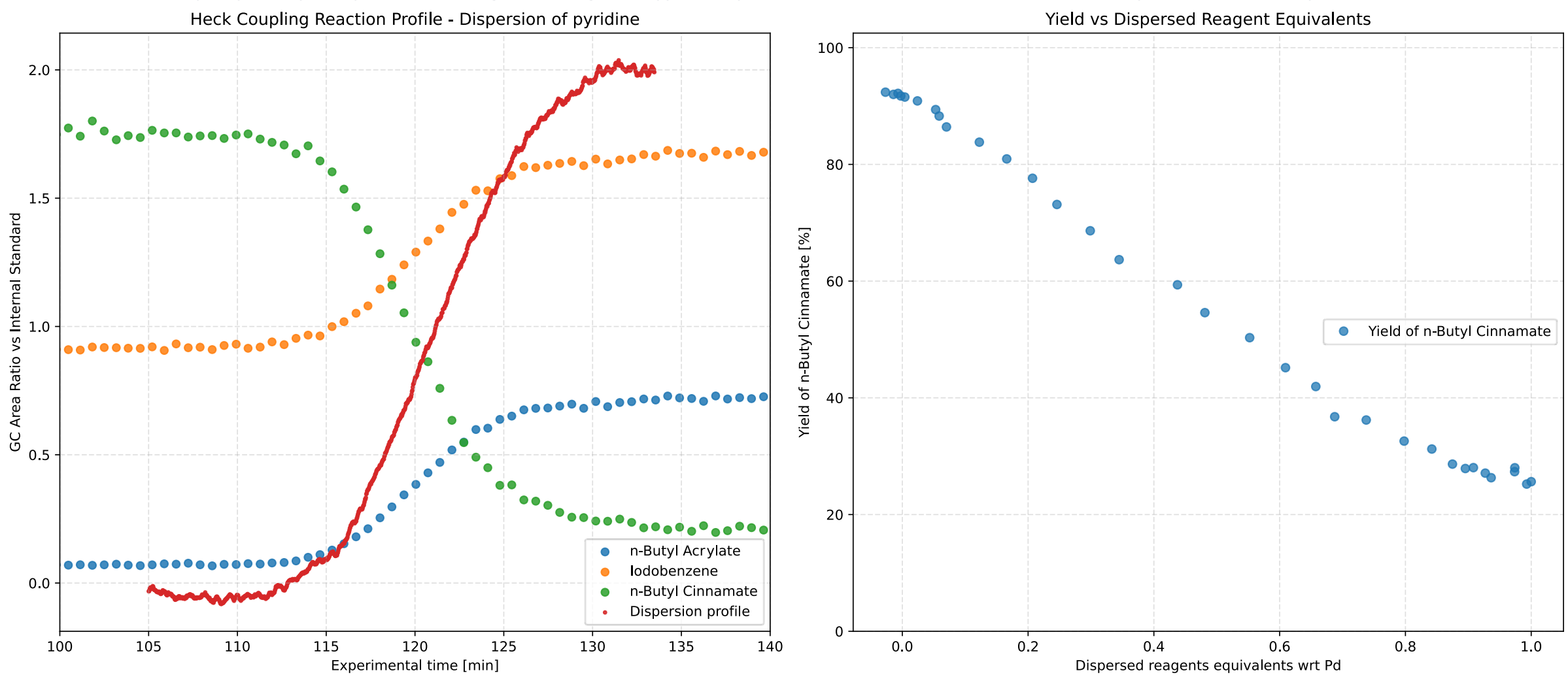

Figure S38. Dispersion generated concentration gradient of pyridine. Left: GC traces of reaction components and dispersion profile of dispersed reagent. Right: Effect of pyridine concentration on n-butyl cinnamate yield.

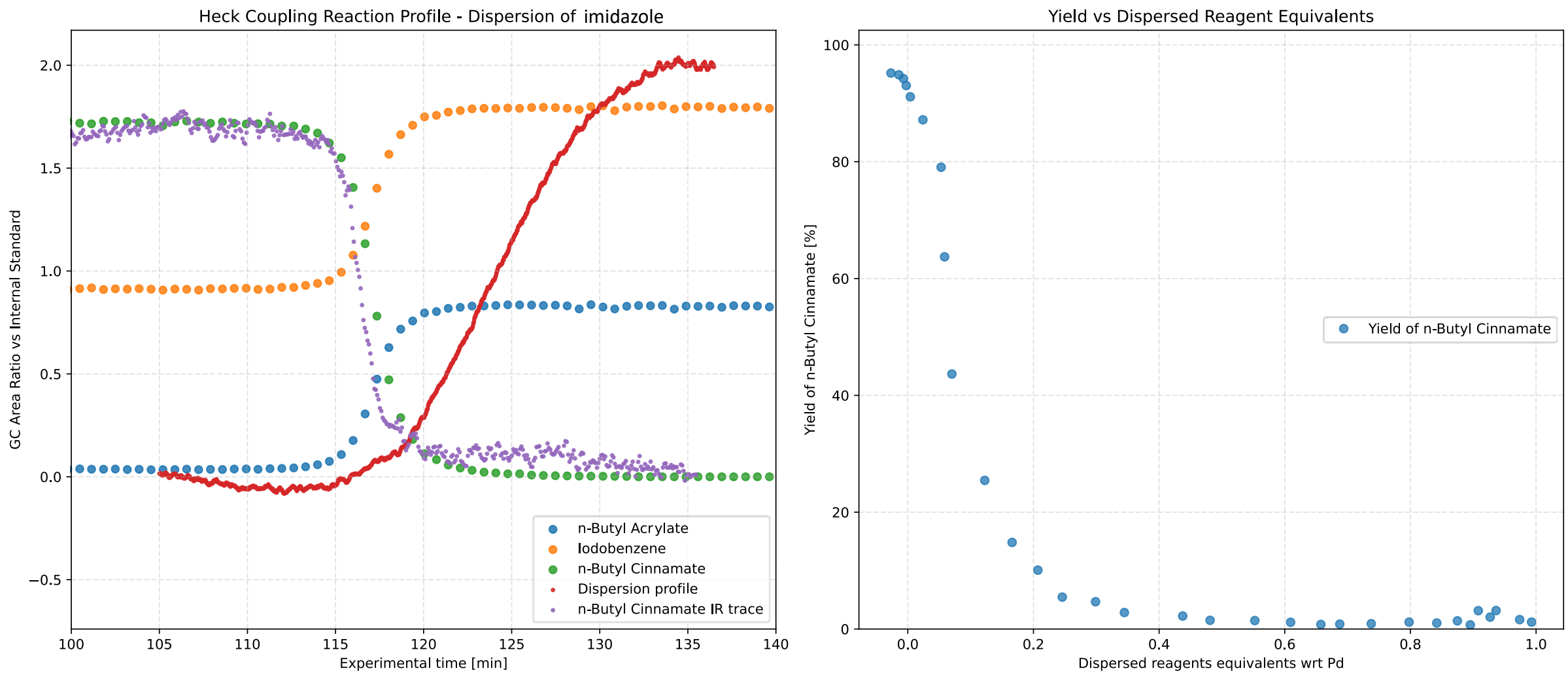

Figure S39. Dispersion generated concentration gradient of imidazole. Left: GC and IR traces of reaction components and dispersion profile of dispersed reagent. Right: Effect of imidazole concentration on n-butyl cinnamate yield.

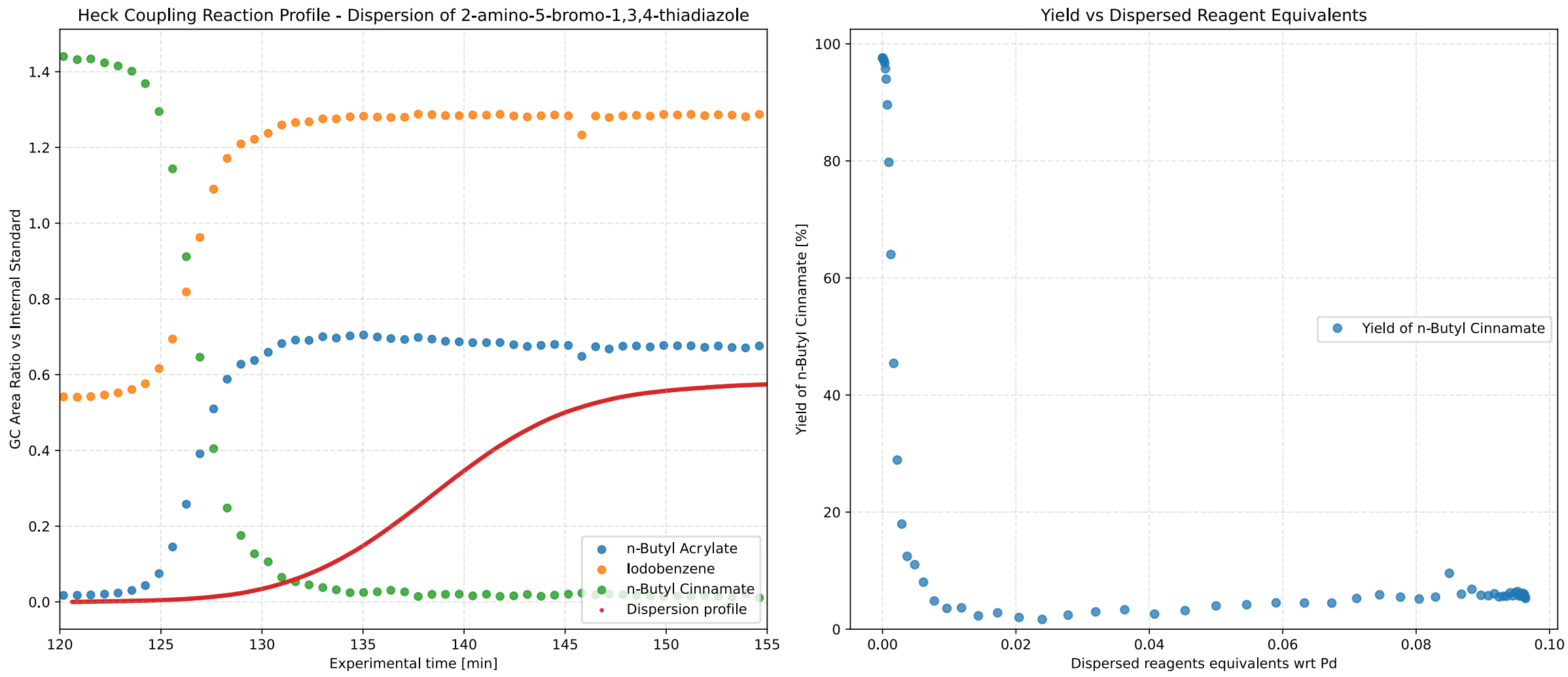

Figure S40. Dispersion generated concentration gradient of 2-amino-5-bromo-1,3,4-thiadiazole. Left: GC traces of reaction components and dispersion profile of dispersed reagent. Right: Effect of 2-amino-5-bromo-1,3,4-thiadiazole concentration on n-butyl cinnamate yield.

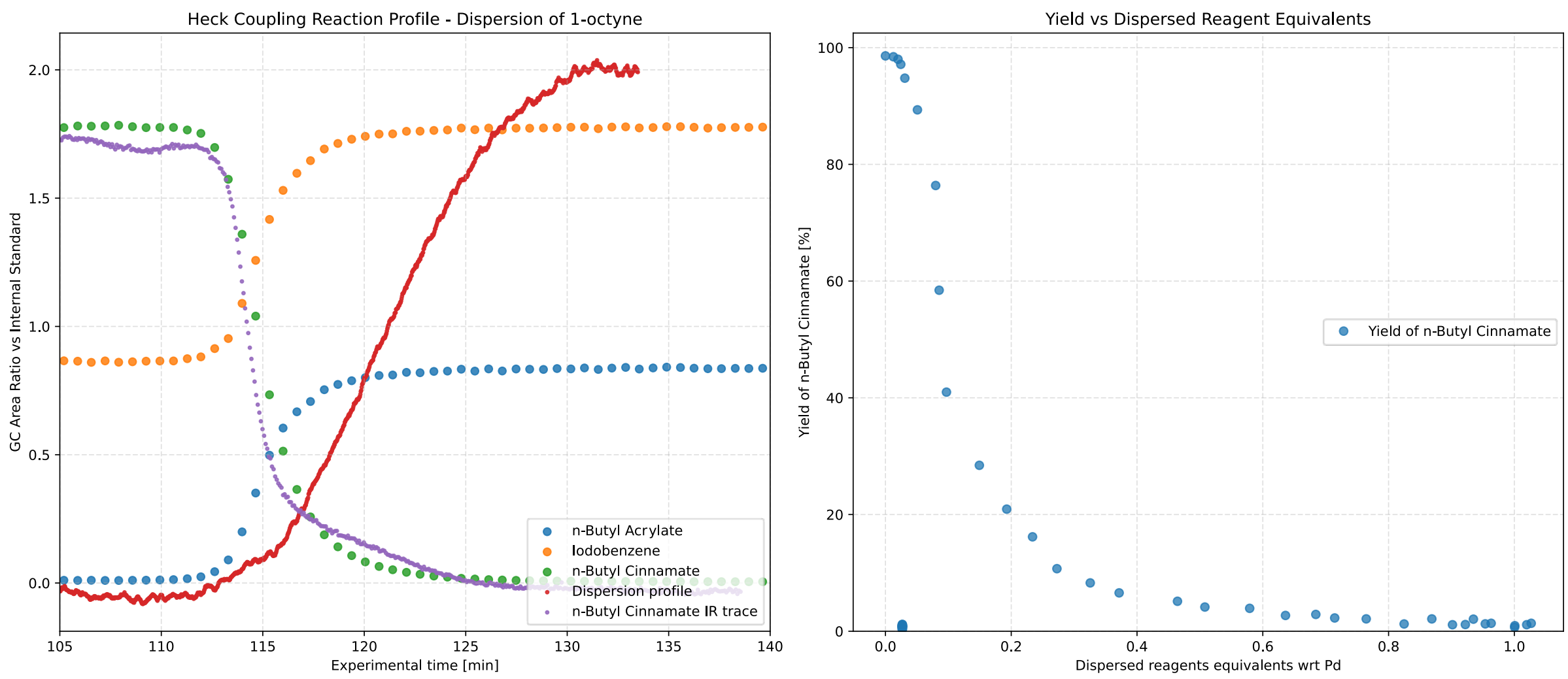

Figure S41. Dispersion generated concentration gradient of 1-octyne. Left: GC and IR traces of reaction components and dispersion profile of dispersed reagent. Right: Effect of 1-octyne concentration on n-butyl cinnamate yield.

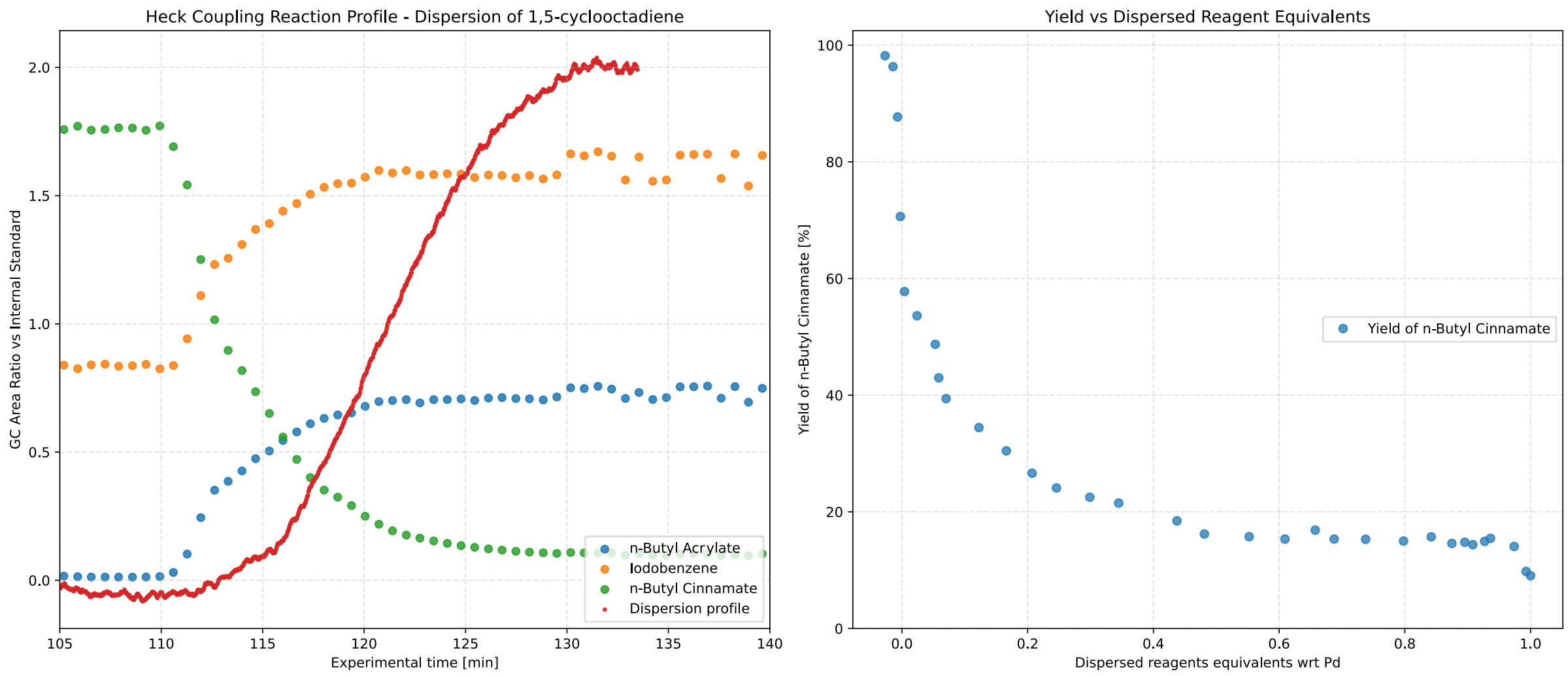

Figure S42. Dispersion generated concentration gradient of 1,5-cyclooctadiene. Left: GC and IR traces of reaction components and dispersion profile of dispersed reagent. Right: Effect of 1,5-cyclooctadiene concentration on n-butyl cinnamate yield.

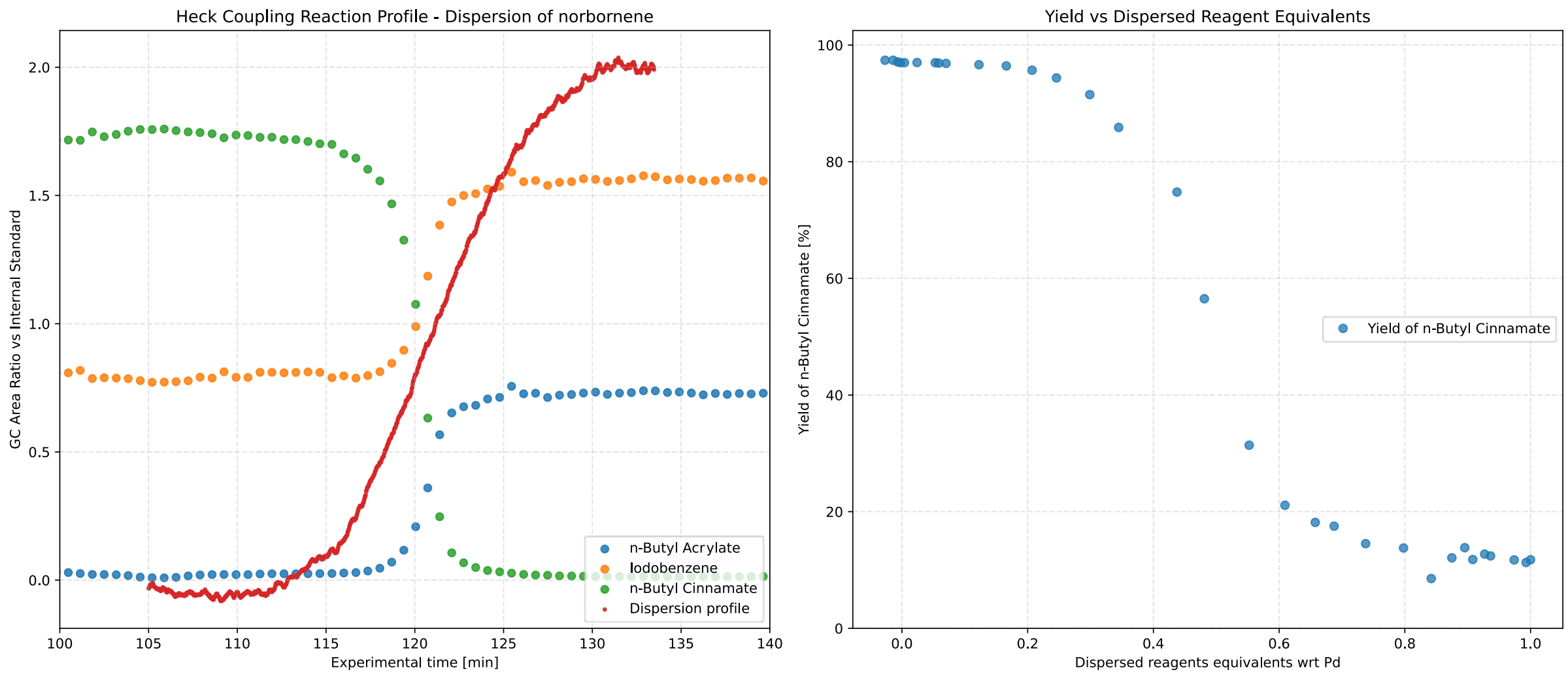

Figure S43. Dispersion generated concentration gradient of norbornene. Left: GC and IR traces of reaction components and dispersion profile of dispersed reagent. Right: Effect of norbornene concentration on n-butyl cinnamate yield.

2. 4. 4. Validation of results using steady-state, variable flow rate (VFR) generated concentration gradient, and dispersion-generated concentration gradient flow methods

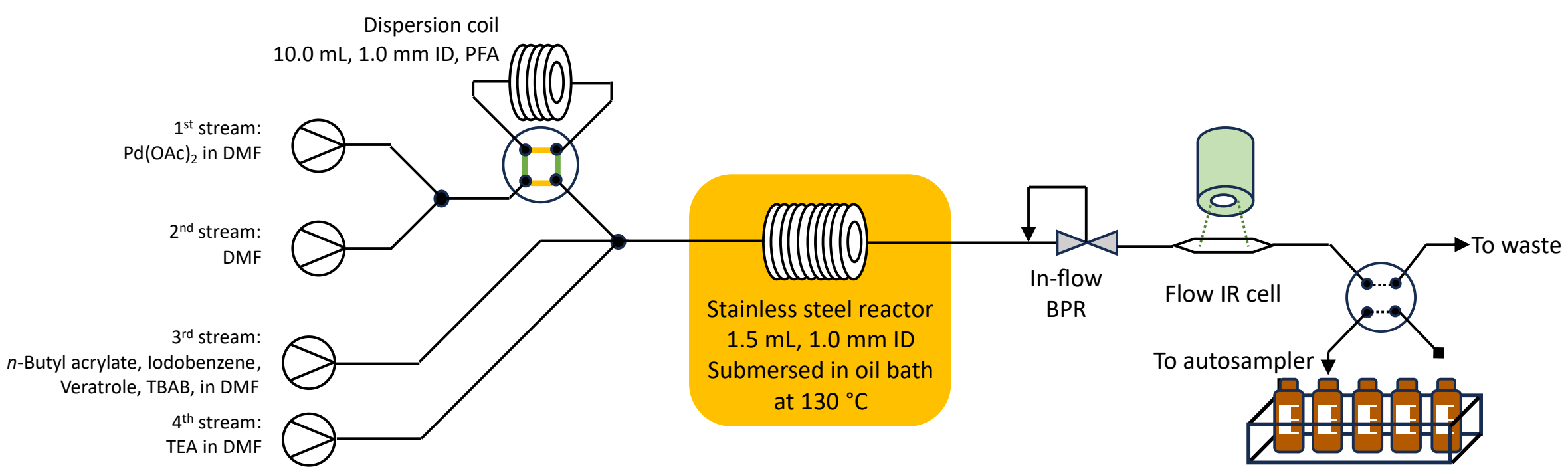

Figure S44. Flow setup used in validation of dispersion generated concentration gradient utilising VFR method.

Procedure for experiment utilising VFR methodology:

$Pd(OAc)_2$ (7.60 mg, 0.034 mmol, 0.45 mol%) was dissolved in DMF (15.0 mL) and obtained mixture was connected to the 1st dispersed reagent flow stream. Veratrole (internal standard, 0.96 mL, 1.04 g, 7.53 mmol, 1.0 eq.), TBAB (2.43 g, 7.53 mmol, 1.0 eq.), iodobenzene (1.08 mL, 1.97 g, 9.68 mmol, 1.3 eq.), and *n*-butyl acrylate (1.07 mL, 0.97 g, 7.53 mmol, 1.0 eq.) were dissolved in DMF (15.0 mL) and obtained mixture was connected to the 3rd reagent stream. Triethylamine (2.49 mL, 1.8 g, 17.84 mmol, 2.4 eq.) was dissolved in DMF (15.0 mL) and obtained solution was connected to 4th reagent stream. DMF was connected to all solvent lines and 2nd reagent stream. Flow system was composed of two piston pumps (Vapourtec R2 units) delivering dispersed reagent and DMF but bypassing the dispersion coil. Such a stream was then joined with other reagent streams and directed to tubular reactor (stainless steel, 1.5 mL, 1.0 mm ID, in oil bath at 130 °C), BPR (250 psi), in-line IR flow cell and autosampler. Solvent was initially pumped using 3x lines (2nd, 3rd, and 4th streams) at the 0.1 $^{mL}/_{min}$ flow rate until reactor temperature equilibrated. Flow rates of 1st and 2nd lines were then controlled by custom python script, while feeding valves of 3rd and 4th were switched to reagent lines and 15.0 mL plugs of each solution were pumped through the system. At the end of the reagents plugs injection, the feeding valves were returned to the solvent feed.

Reaction progress was monitored by off-line GC measurements. GC samples were prepared by acquisition of 0.2 mL of reaction mixture and further dilution in 1.0 mL of acetone.

Procedure for experiment utilising dispersion-generated concentration gradient methodology:

$Pd(OAc)_2$ (7.60 mg, 0.034 mmol, 0.45 mol%) was dissolved in DMF (15.0 mL) and obtained mixture was connected to the 1st dispersed reagent flow stream. Veratrole (internal standard, 0.96 mL, 1.04 g, 7.53 mmol, 1.0 eq.), TBAB (2.43 g, 7.53 mmol, 1.0 eq.), iodobenzene (1.08 mL, 1.97 g, 9.68 mmol, 1.3 eq.), and *n*-butyl acrylate (1.07 mL, 0.97 g, 7.53 mmol, 1.0 eq.) were dissolved in DMF (15.0 mL) and obtained mixture was connected to the 3rd reagent stream. Triethylamine (2.49 mL, 1.8 g, 17.84 mmol, 2.37 eq.) was dissolved in DMF (15.0 mL) and obtained solution was connected to 4th reagent stream. DMF was connected to all solvent lines. 2nd stream was not in use. Flow system was composed of a piston pumps (Vapourtec R2 units) delivering dispersed reagent to the dispersion coil (PFA, 10.0 mL, 1.0 mm ID), then joined with other reagent streams and directed to tubular reactor (stainless steel, 1.5 mL, 1.0 mm ID, in oil bath at 130 °C), BPR (250 psi), in-line IR flow cell and autosampler. Solvent was initially pumped at all lines at the 0.1 $^{mL}/_{min}$ flow rate until reactor temperature equilibrated. Feeding valve of the dispersion coil was then switched to deliver plug of the $Pd(OAc)_2$ to the dispersion coil. Dispersed reagent plug was infused into the flow system earlier in time wrt to steady-state reagents to allow for the plug to be dispersed and then joined with other reaction components – in this case 2nd and 3rd feeding valves had a delay of 40 min (4.0 mL each) before switching to reagents lines. Feeding valves of 3rd and 4th stream were switched to reagent lines and 15.0 mL plugs of each solution were pumped through the system. At the end of the reagents plugs injection, the feeding valves were returned to the solvent feed.

Reaction progress was monitored by off-line GC measurements. GC samples were prepared by acquisition of 0.2 mL of reaction mixture and further dilution in 1.0 mL of acetone. The earlier acquired dispersion profile was then overlaid with the acquired GC data allowing for calculation of the $Pd(OAc)_2$ concentration during the experiment.

2. 5. Sonogashira coupling reaction between bromobenzene and 1-octyne

2. 5. 1. Sample GC trace of Sonogashira reaction

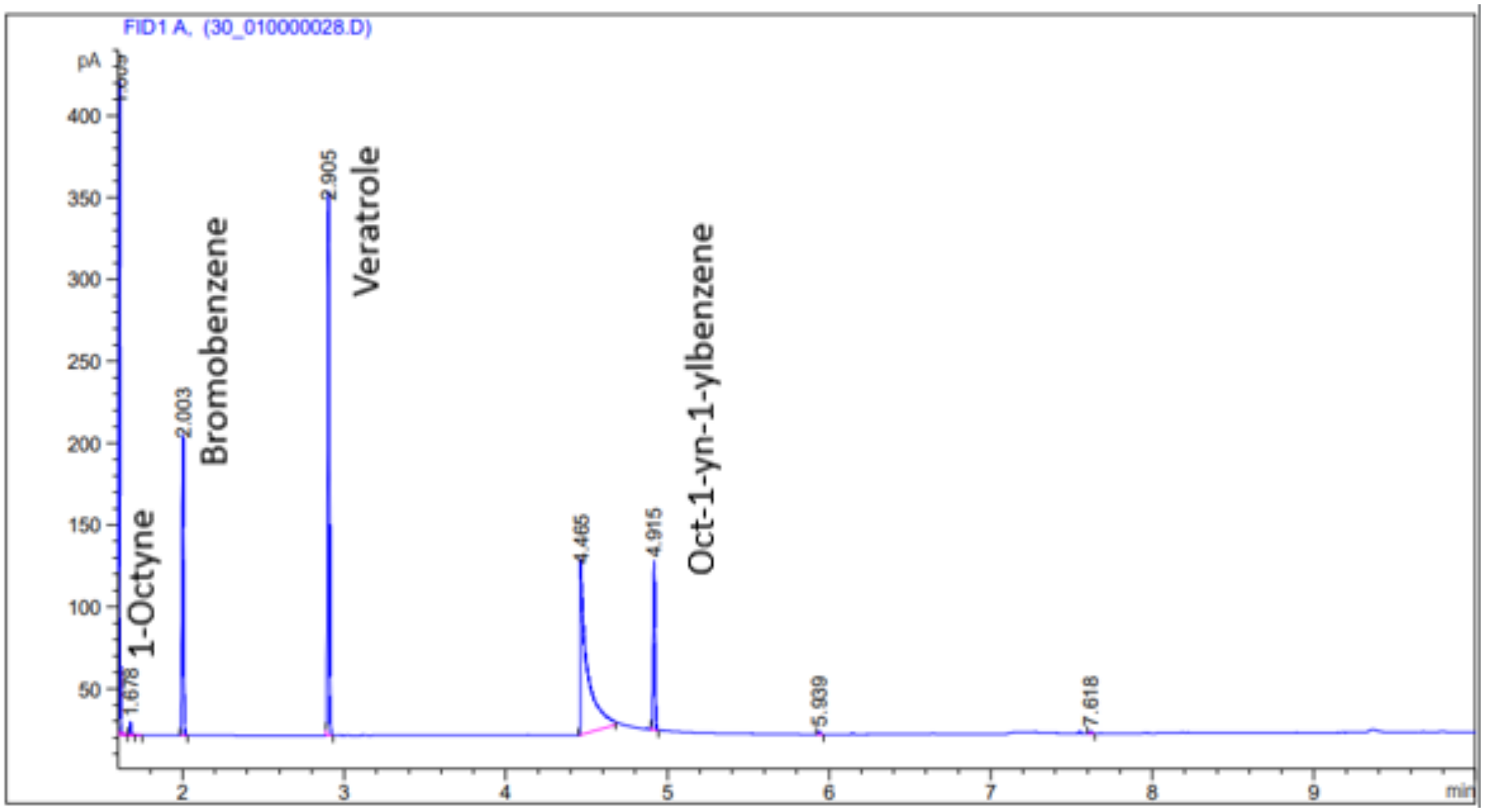

Figure S45. Example GC trace from Sonogashira coupling reaction.

2. 5. 2. Experimental protocol for flow studies utilising dispersion-generated concentration gradient method in Sonogashira coupling reaction, example procedure of XantPhos dispersion

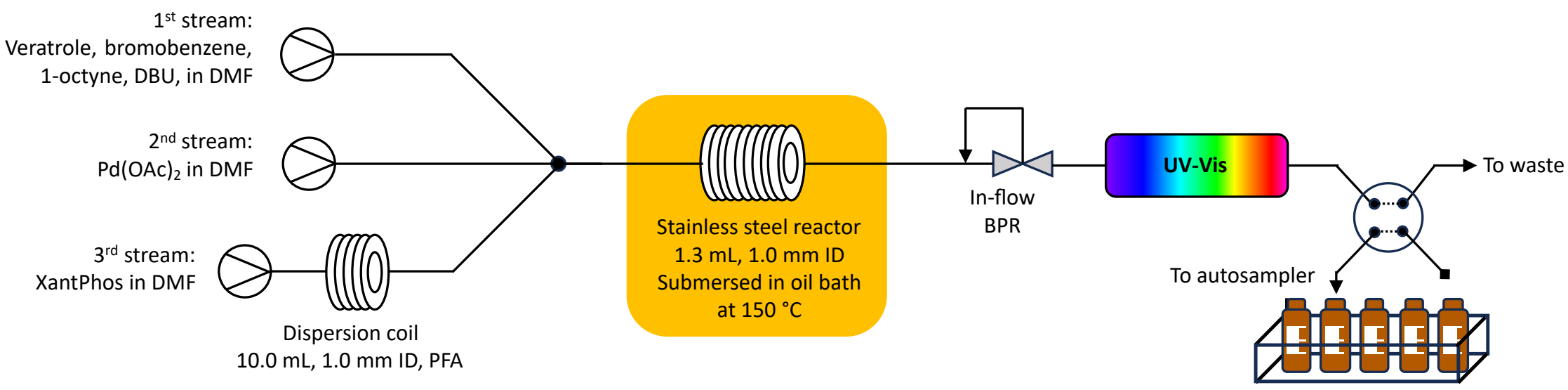

Figure S46. Flow setup used for Sonogashira coupling experiments.

Veratrole (internal standard, 0.34 mL, 0.370 g, 2.70 mmol, 1.1 eq.), bromobenzene (0.385 g, 2.45 mmol, 1.0 eq.), 1-octyne (0.72 mL, 0.540 g, 4.90 mmol, 2.0 eq.), and DBU (0.747 g, 4.90 mmol, 2.0 eq.) were dissolved in DMF (15.0 mL) and the obtained mixture was connected to the 1st reagent stream. $Pd(OAc)_2$ (2.75 mg, 0.012 mmol, 0.5 mol%) was dissolved in DMF (15.0 mL) and the obtained mixture was connected to the 2nd reagent flow stream. XantPhos (0.292 g, 0.51 mmol, 0.206 eq.) was dissolved in DMF (15.0 mL) and the obtained solution was connected to the 3rd reagent stream. DMF was connected to all solvent lines. The flow system was composed of piston pumps (Vapourtec R2 units) delivering dispersed reagent to the dispersion coil (PFA, 10.0 mL, 1.0 mm ID), then joined with other reagent streams and directed to a tubular reactor (stainless steel, 1.3 mL, 1.0 mm ID, in oil bath at 150 °C), BPR (250 psi), in-line UV-Vis flow cell and autosampler. Solvent was initially pumped at all lines at the 0.1 $^{mL}/_{min}$ flow rate until reactor temperature equilibrated. The feeding valve of the dispersion coil was then switched to deliver a plug of XantPhos to the dispersion coil. The dispersed reagent plug was infused into the flow system earlier in time wrt to steady-state reagents to allow for the plug to be dispersed and then joined with other reaction components. Feeding valves of 1st and 2nd stream were switched to reagent lines and 15.0 mL plugs of each solution were pumped through

the system. At the end of the reagent-plug injection, the feeding valves were returned to the solvent feed.

Reaction progress was monitored by off-line GC measurements. GC samples were prepared by acquisition of 0.2 mL of reaction mixture and further dilution in 1.0 mL of acetone. The earlier acquired dispersion profile was then overlaid with the acquired GC data allowing for calculation of the $Pd(OAc)_2$ concentration during the experiment.

2. 5. 3. Results of dispersion-generated concentration gradient method in Sonogashira coupling reaction, GC and IR traces of process components, dispersion profiles, and yield as a function of dispersed component concentration

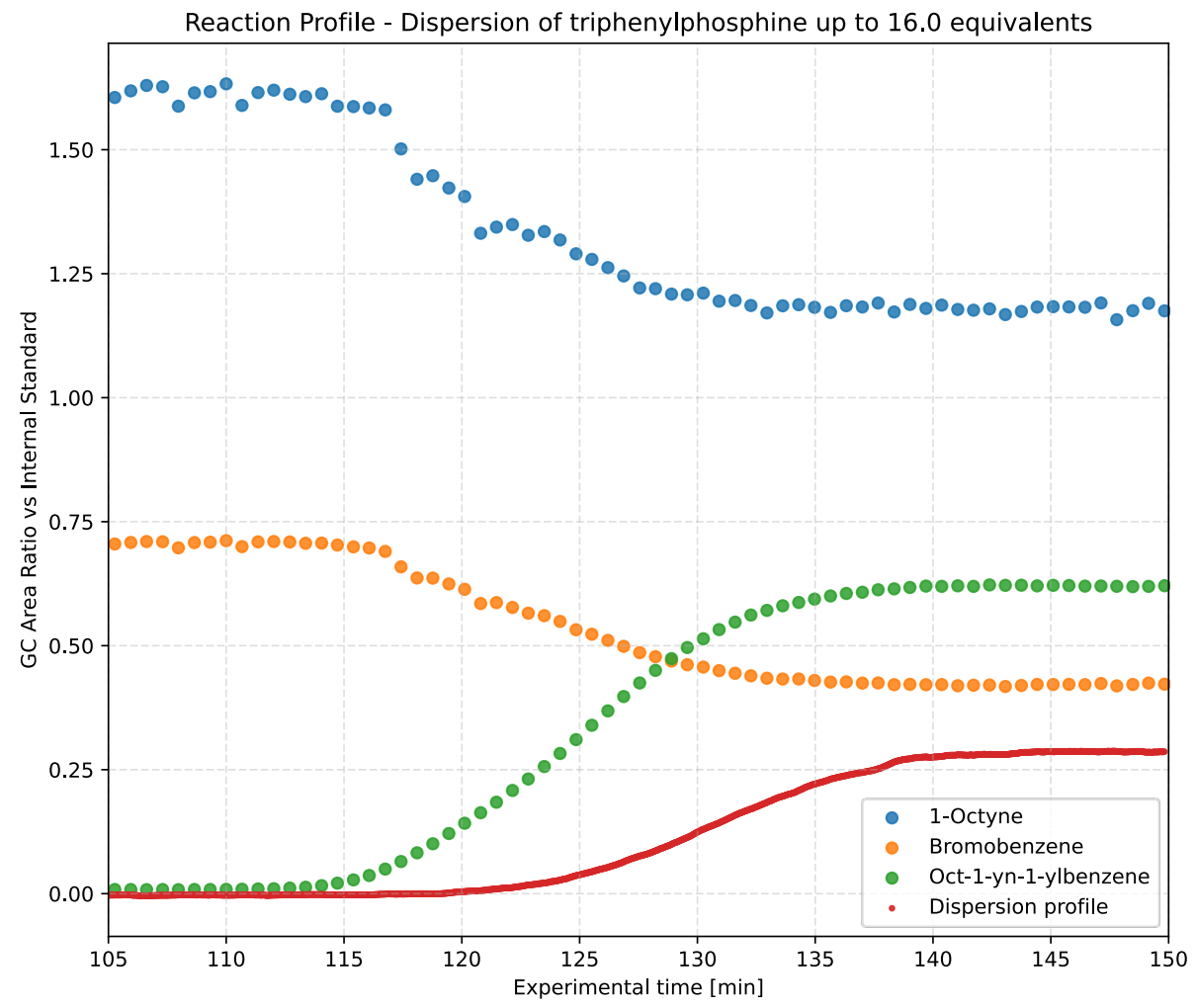

Figure S47. Dispersion generated concentration gradient of triphenylphosphine, up to 16.0 eq. Left: GC traces of reaction components and dispersion profile of dispersed reagent. Right: Effect of triphenylphosphine concentration on oct-1-yn-1-ylbenzene yield.

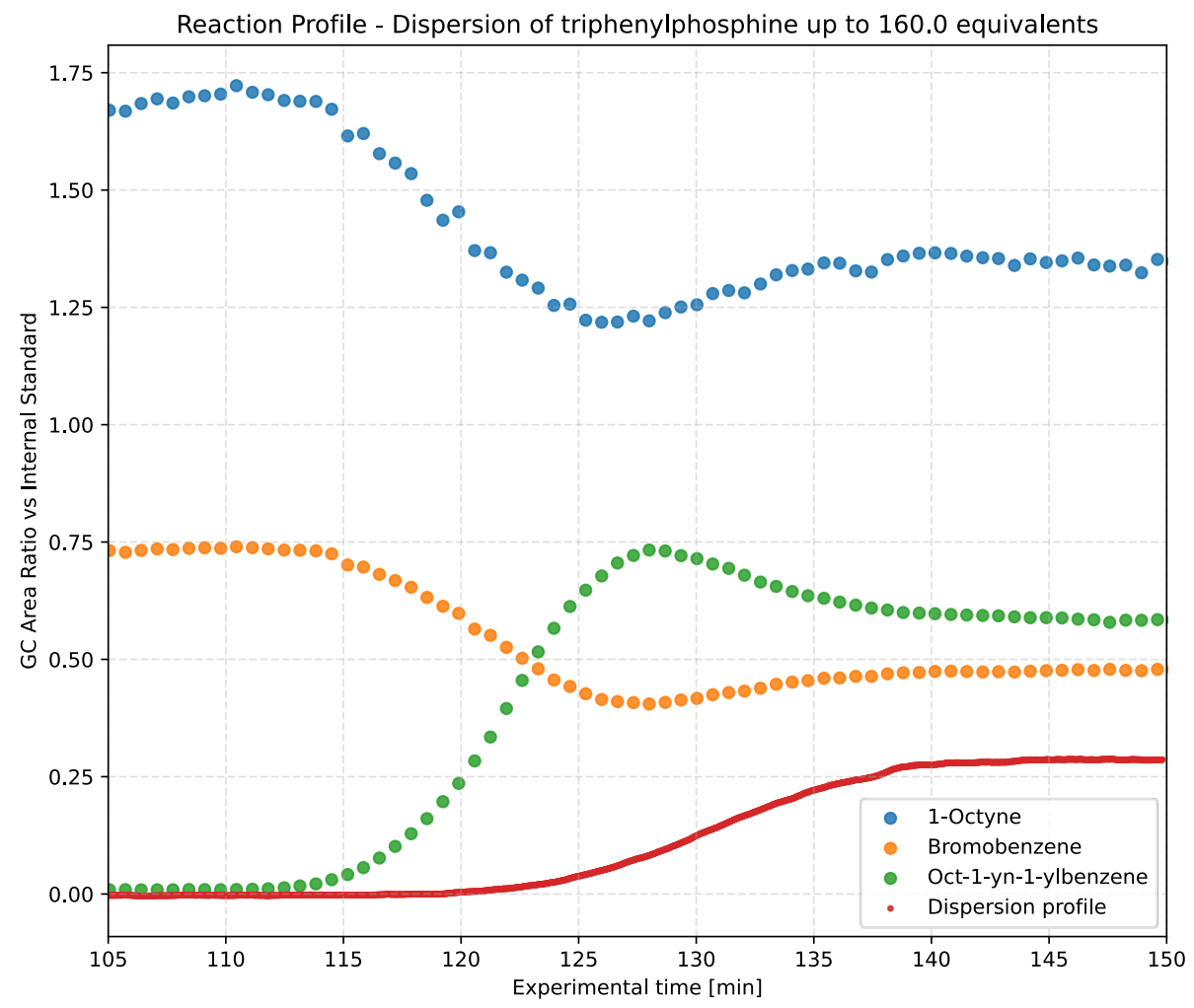

Figure S48. Dispersion generated concentration gradient of triphenylphosphine, up to 160.0 eq. Left: GC traces of reaction components and dispersion profile of dispersed reagent. Right: Effect of triphenylphosphine concentration on oct-1-yn-1-ylbenzene yield.

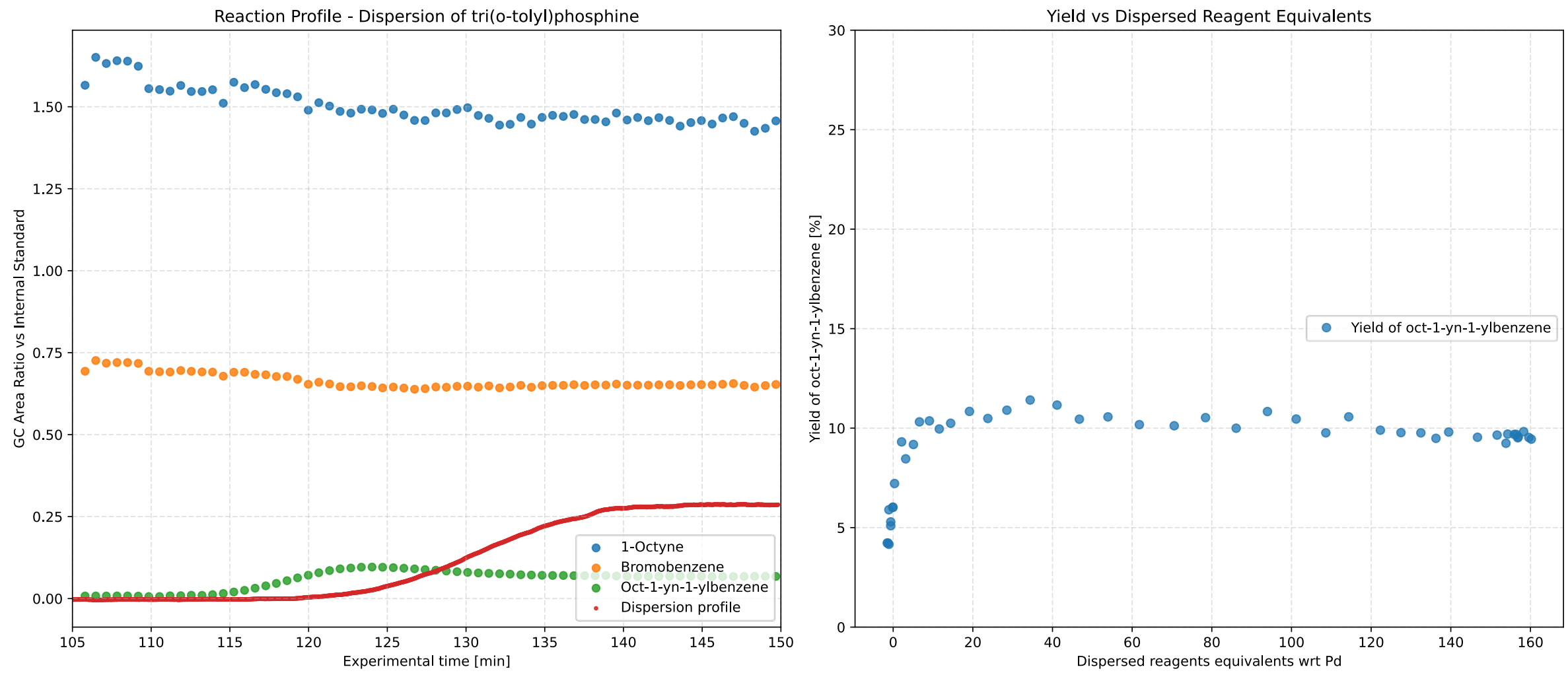

Figure S49. Dispersion generated concentration gradient of tri(o-tolyl)phosphine. Left: GC traces of reaction components and dispersion profile of dispersed reagent. Right: Effect of tri(o-tolyl)phosphine concentration on oct-1-yn-1-ylbenzene yield.

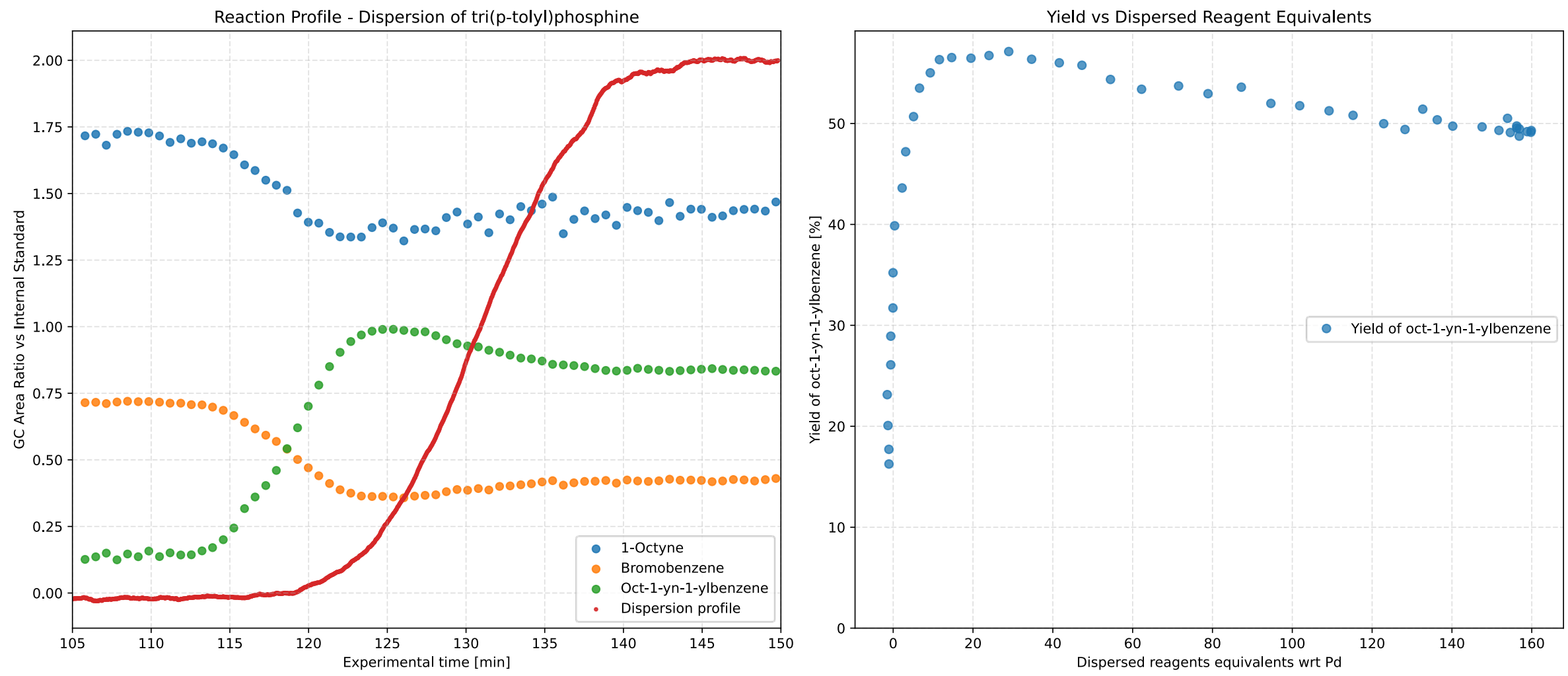

Figure S50. Dispersion generated concentration gradient of tri(p-tolyl)phosphine. Left: GC traces of reaction components and dispersion profile of dispersed reagent. Right: Effect of tri(p-tolyl)phosphine concentration on oct-1-yn-1-ylbenzene yield.

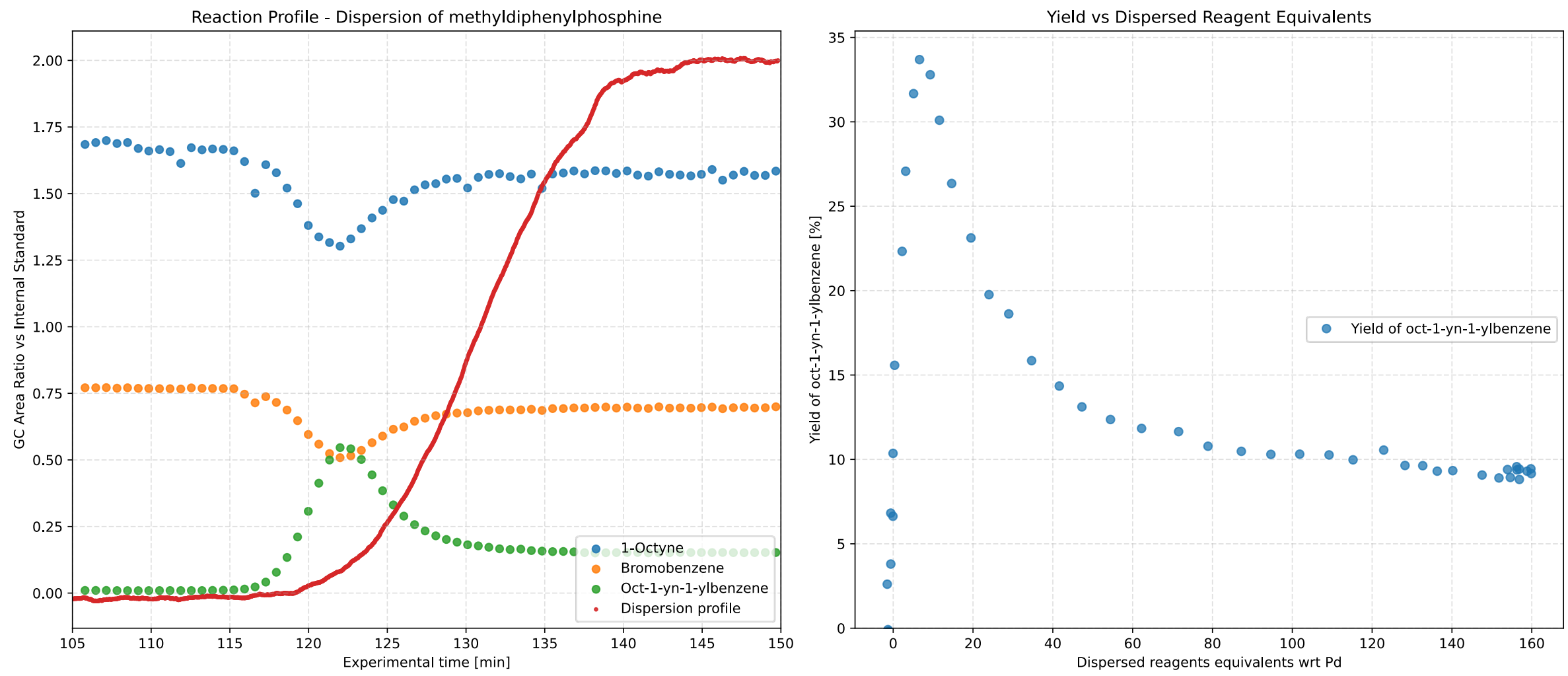

Figure S51. Dispersion generated concentration gradient of methyldiphenylphosphine. Left: GC traces of reaction components and dispersion profile of dispersed reagent. Right: Effect of methyldiphenylphosphine concentration on oct-1-yn-1-ylbenzene yield.

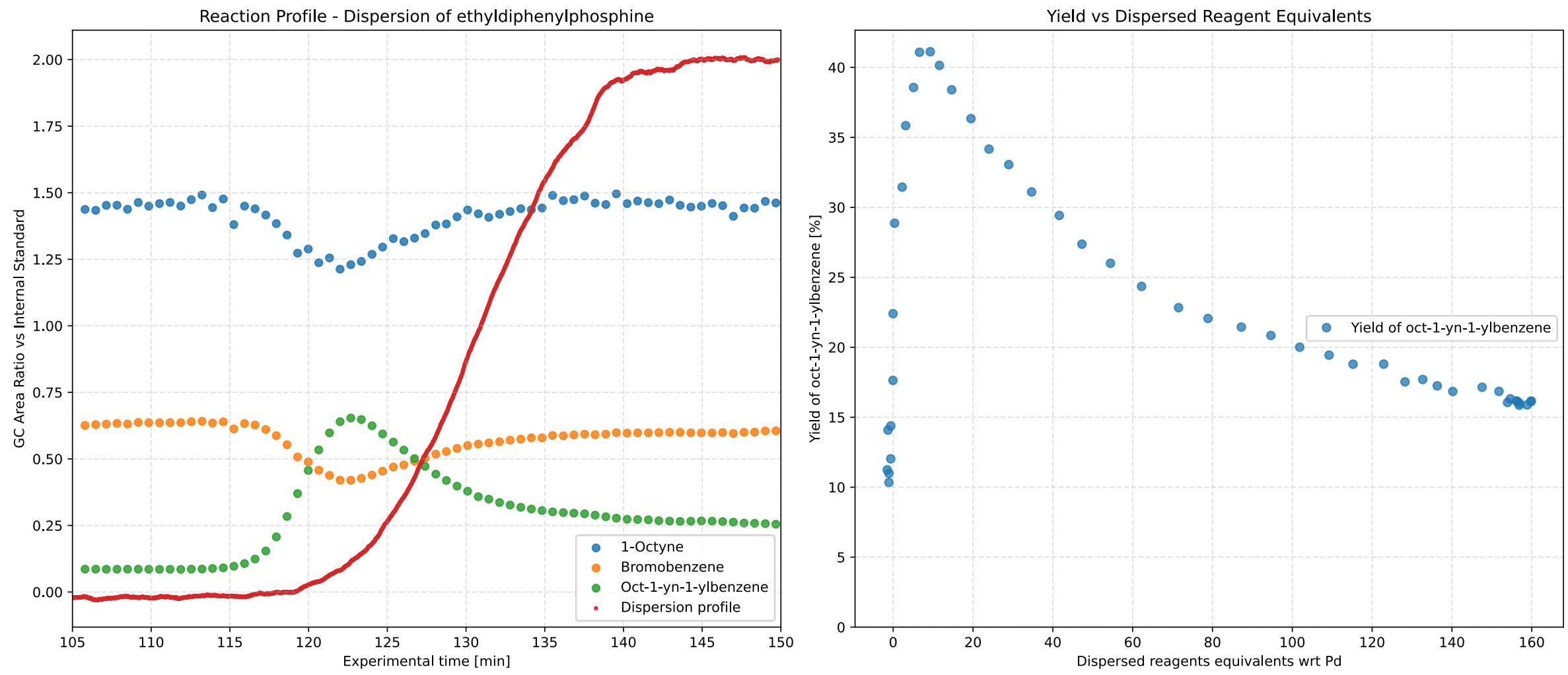

Figure S52. Dispersion generated concentration gradient of ethyldiphenylphosphine. Left: GC traces of reaction components and dispersion profile of dispersed reagent. Right: Effect of ethyldiphenylphosphine concentration on oct-1-yn-1-ylbenzene yield.

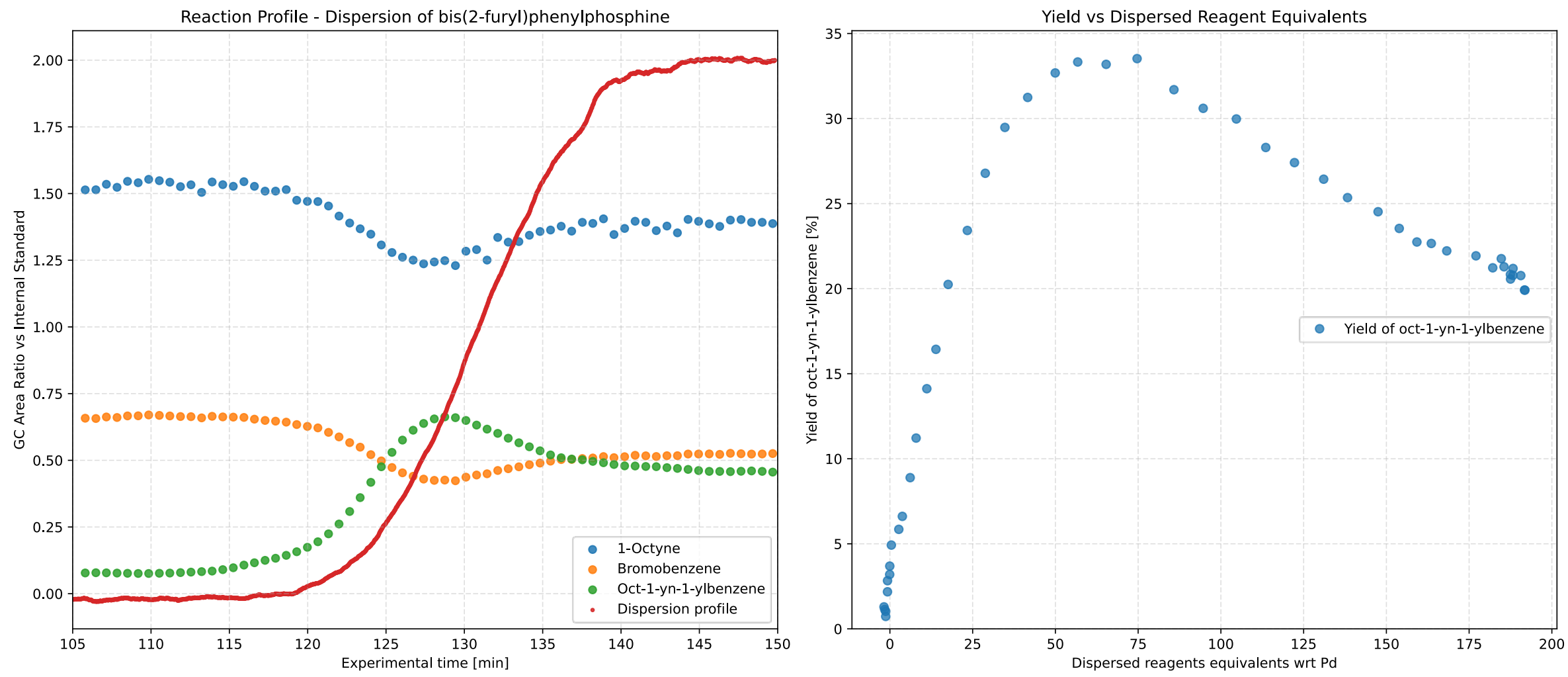

Figure S53. Dispersion generated concentration gradient of bis(2-furyl)phenylphosphine. Left: GC traces of reaction components and dispersion profile of dispersed reagent. Right: Effect of bis(2-furyl)phenylphosphine concentration on oct-1-yn-1-ylbenzene yield.

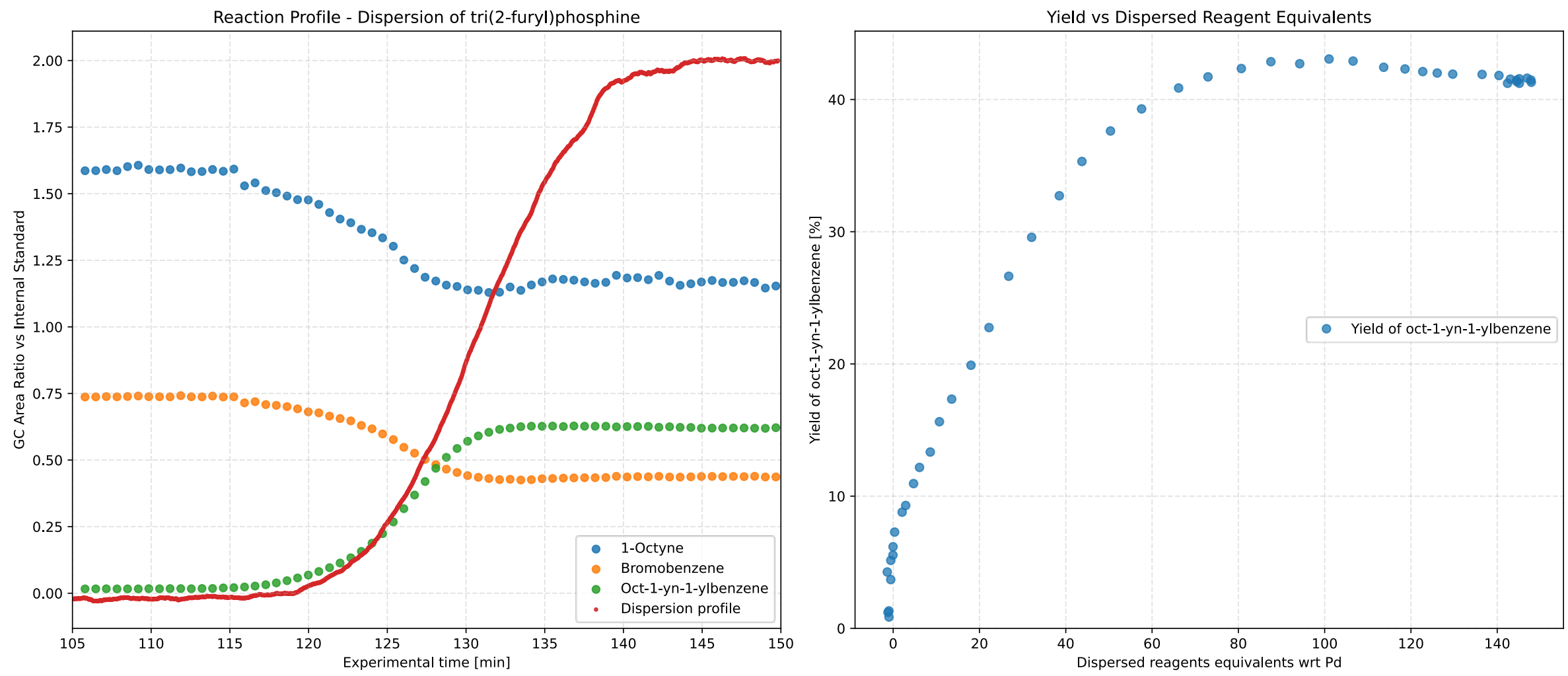

Figure S54. Dispersion generated concentration gradient of tri(2-furyl)phosphine. Left: GC traces of reaction components and dispersion profile of dispersed reagent. Right: Effect of tri(2-furyl)phosphine concentration on oct-1-yn-1-ylbenzene yield.

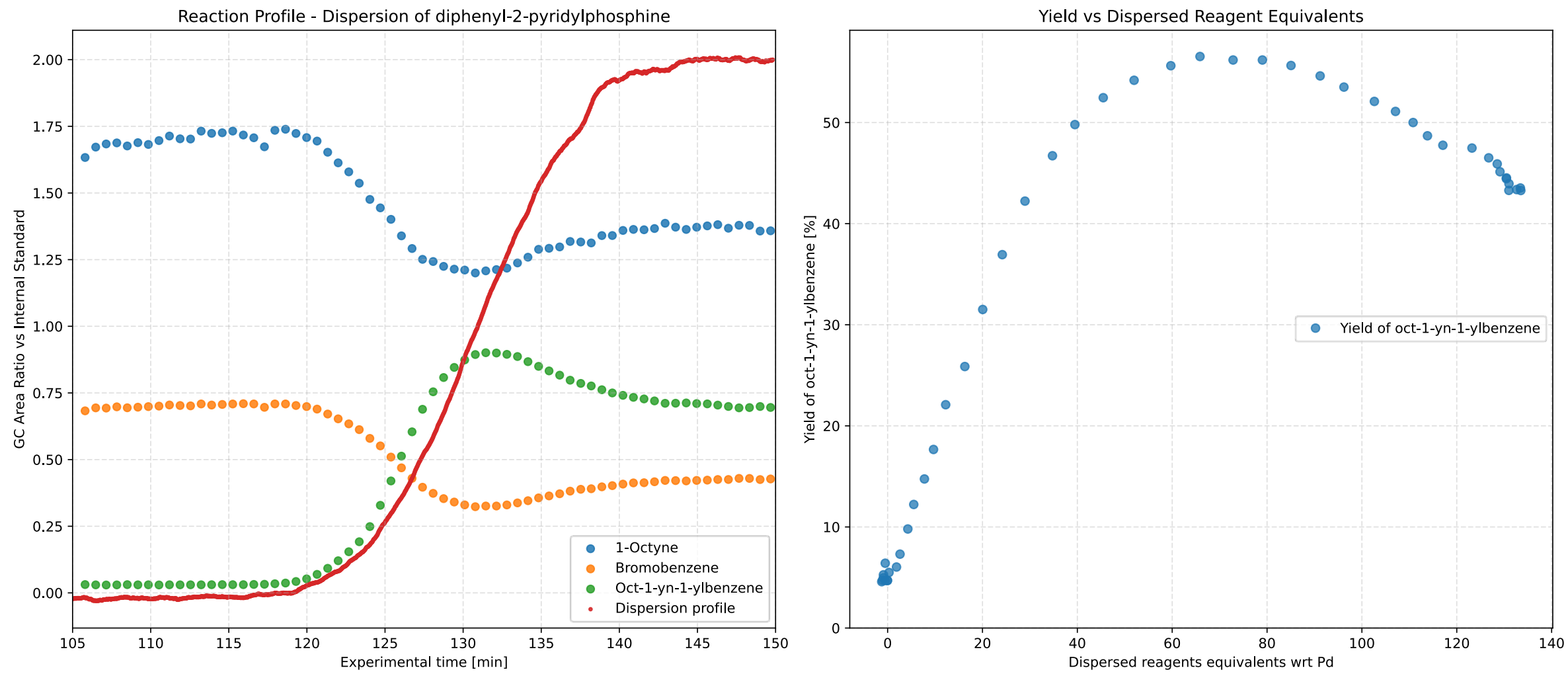

Figure S55. Dispersion generated concentration gradient of diphenyl-2-pyridylphosphine. Left: GC traces of reaction components and dispersion profile of dispersed reagent. Right: Effect of diphenyl-2-pyridylphosphine concentration on oct-1-yn-1-ylbenzene yield.

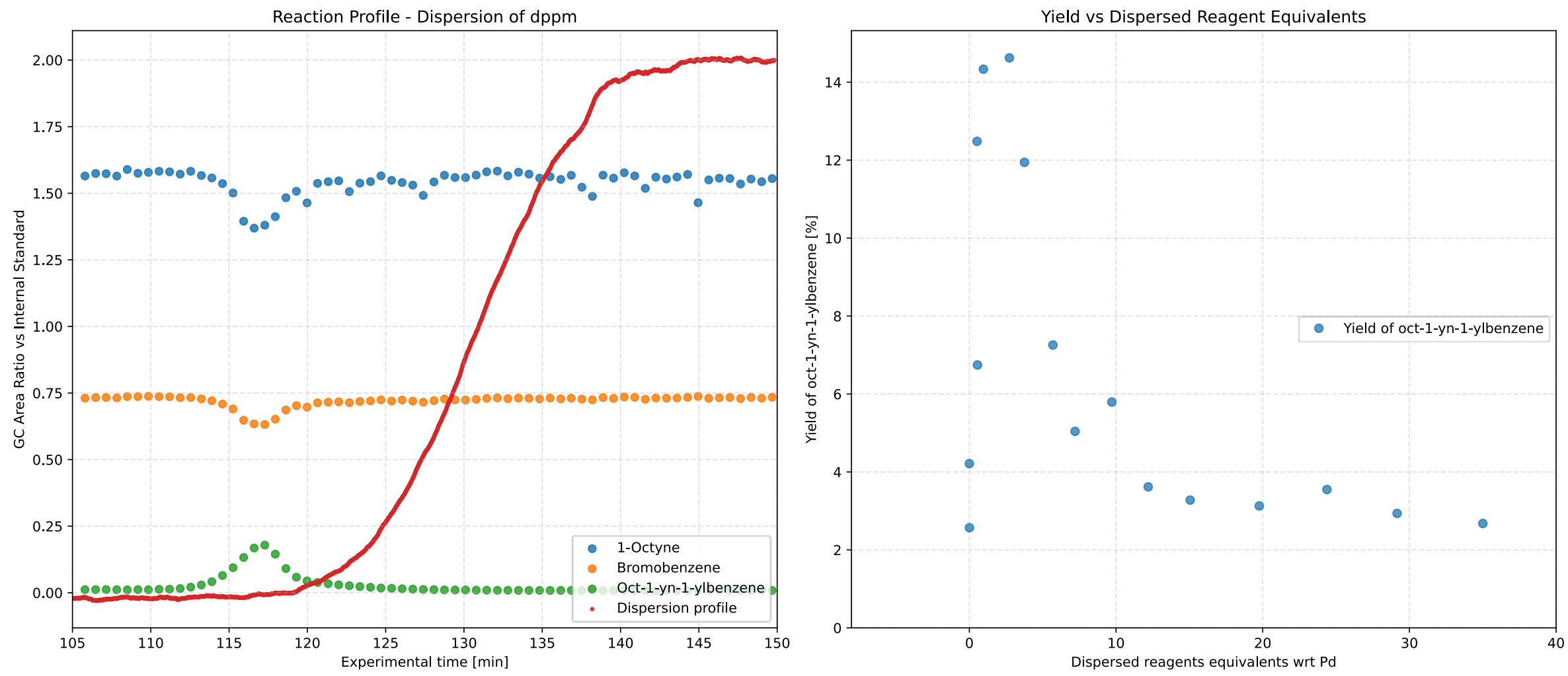

Figure S56. Dispersion generated concentration gradient of dppm. Left: GC traces of reaction components and dispersion profile of dispersed reagent. Right: Effect of dppm concentration on oct-1-yn-1-ylbenzene yield.

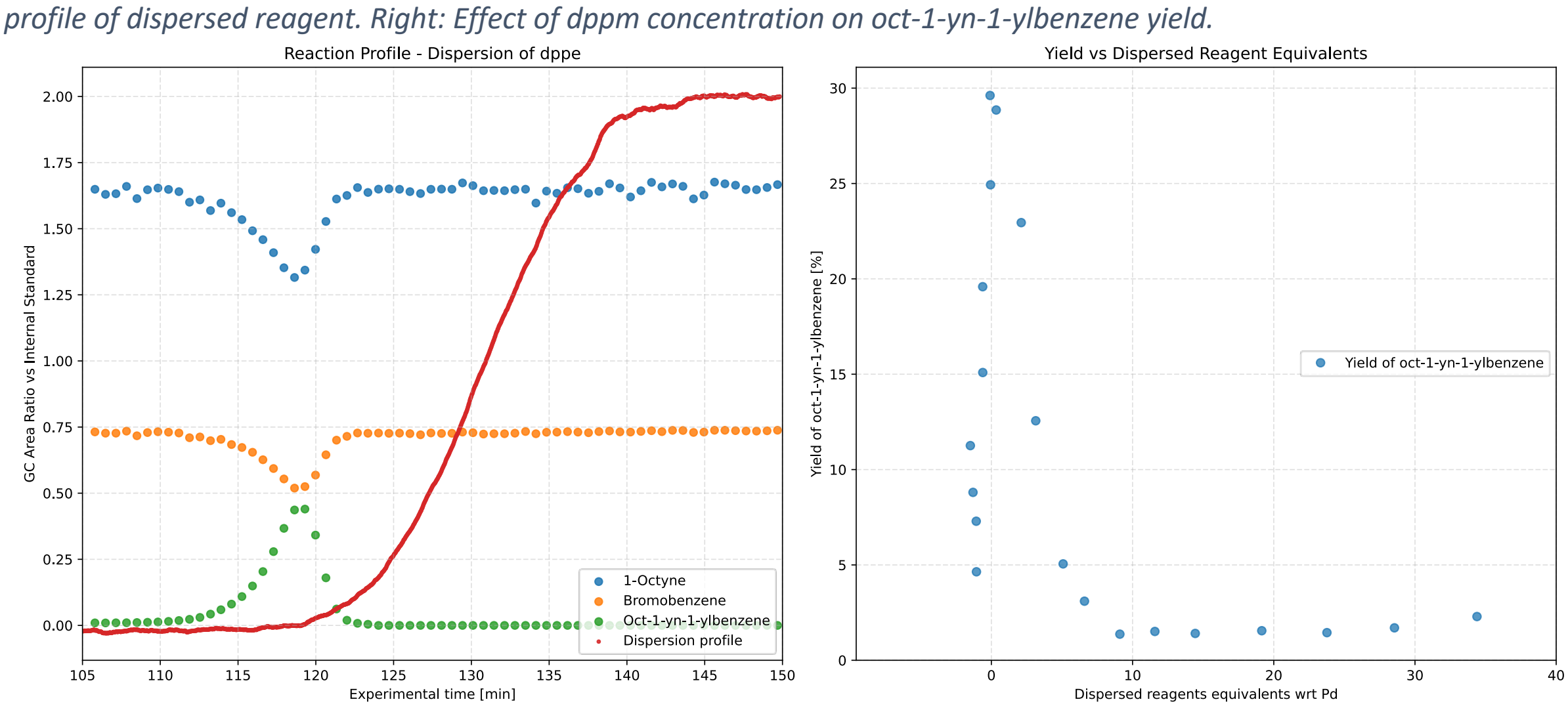

Figure S57. Dispersion generated concentration gradient of dppe. Left: GC traces of reaction components and dispersion profile of dispersed reagent. Right: Effect of dppe concentration on oct-1-yn-1-ylbenzene yield.

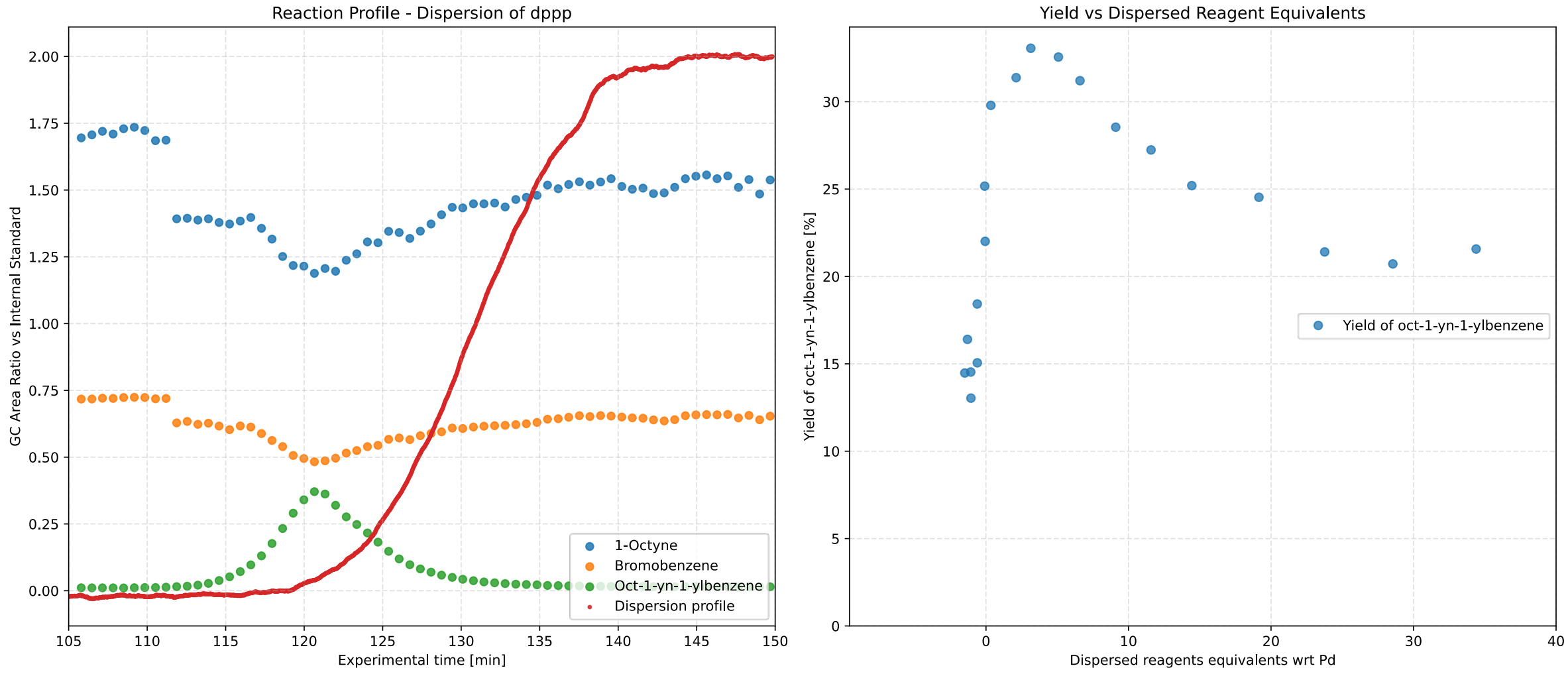

Figure S58. Dispersion generated concentration gradient of dppp. Left: GC traces of reaction components and dispersion profile of dispersed reagent. Right: Effect of dppp concentration on oct-1-yn-1-ylbenzene yield.

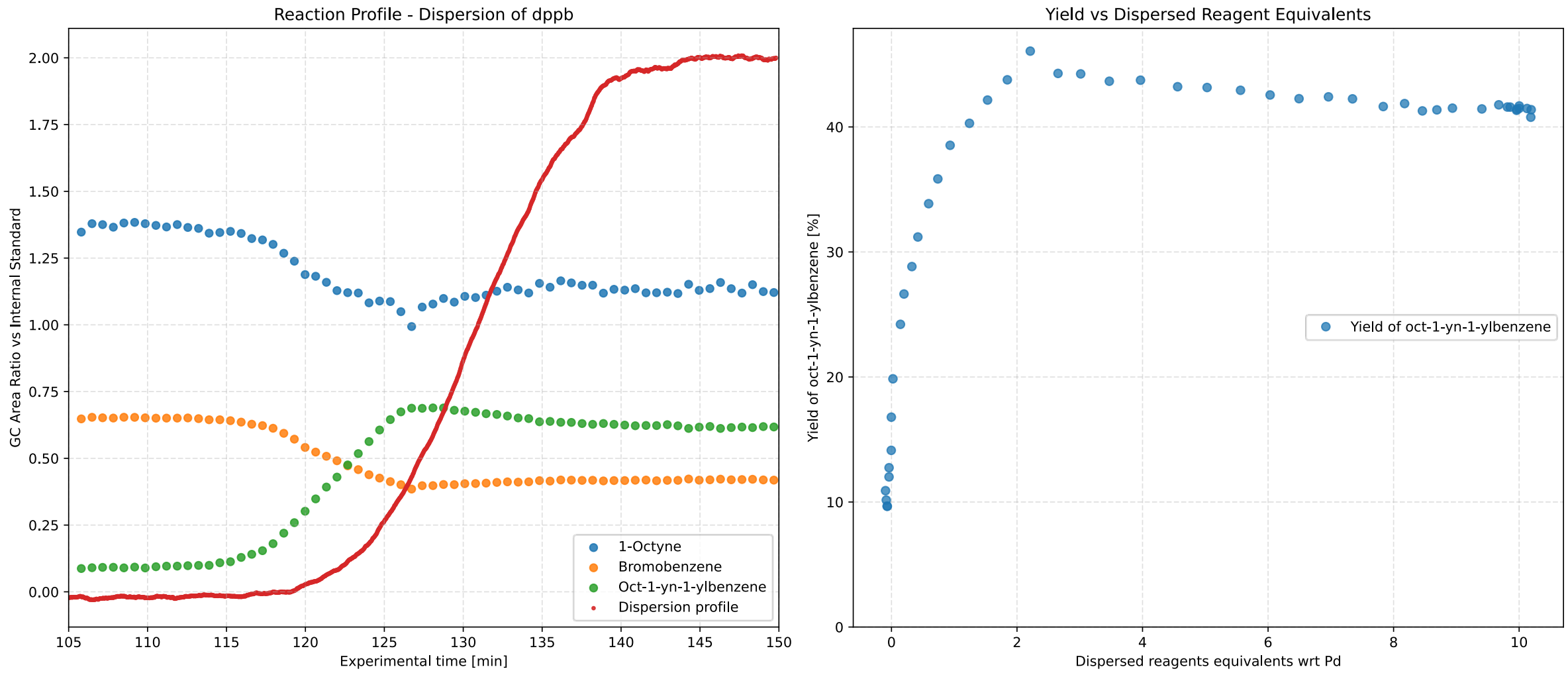

Figure S59. Dispersion generated concentration gradient of dppb. Left: GC traces of reaction components and dispersion profile of dispersed reagent. Right: Effect of dppb concentration on oct-1-yn-1-ylbenzene yield.

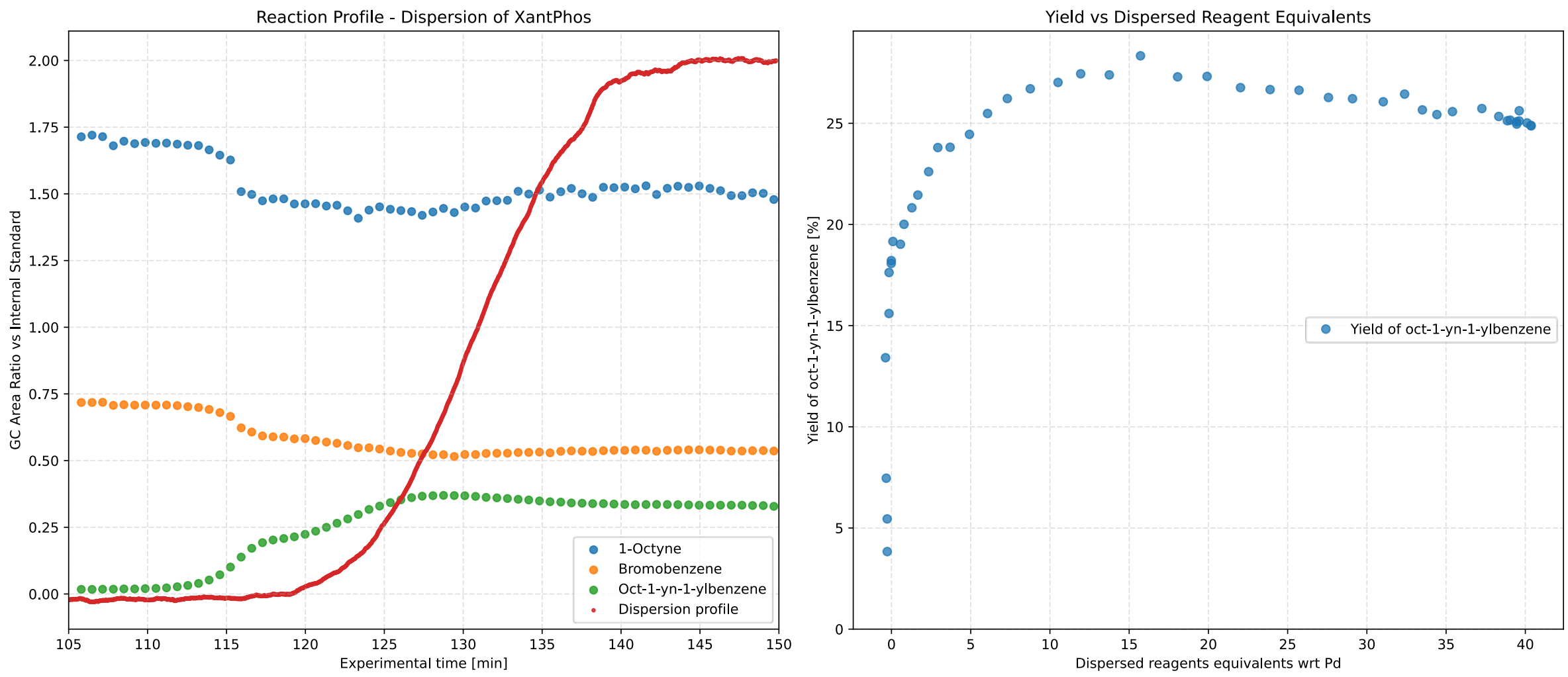

Figure S60. Dispersion generated concentration gradient of XantPhos. Left: GC traces of reaction components and dispersion profile of dispersed reagent. Right: Effect of XantPhos concentration on oct-1-yn-1-ylbenzene yield.

2. 6. Calibration of analytical instruments for S_NAr reaction between 1-fluoro-4-nitrobenzene and pyrrolidine, piperidine, or azepane

UV-Vis spectroscopy was calibrated utilising simple UV-Vis measurements at 434 nm of 1-(4-nitrophenyl)pyrrolidine **15**, 1-(4-nitrophenyl)piperidine **18**, and 1-(4-nitrophenyl)azepane **19** solutions under steady-state conditions. To do so, 5.0 mL of 65.0 mM stock solutions of each amine in THF were prepared, infused into the flow system (Figure S65, two pumps with 0.5 mL/min flow rate each, thus resulting in in-flow two-fold dilution of the sample) and UV-Vis cell while absorbance was measured.

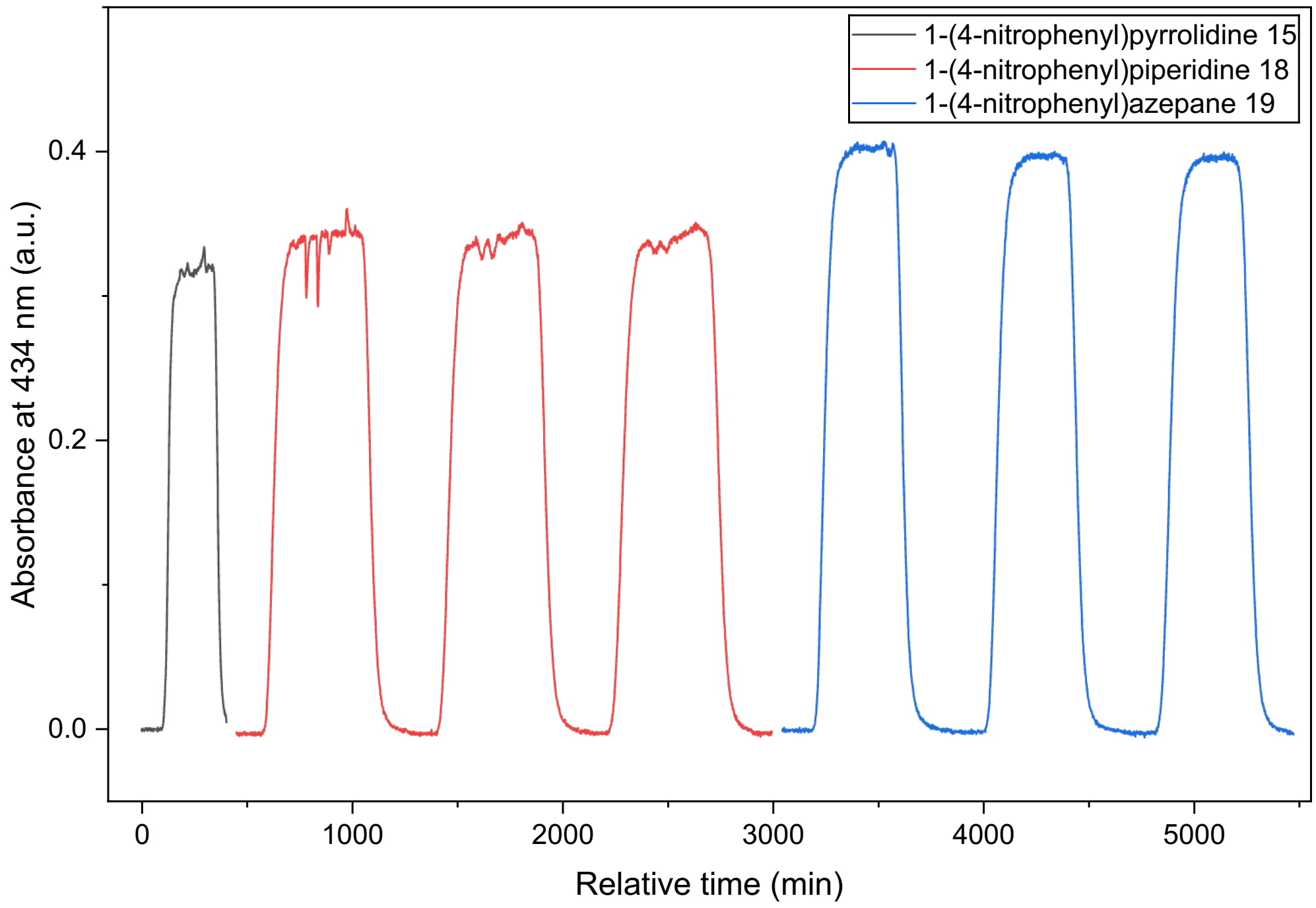

*Figure S61. Absorbance at 434 nm of 32.5 mM (65.0 mM diluted 2x) solutions of **15** (grey), **18** (red), and **19** (blue) in THF.*

Average absorbances of solutions at steady states were then extracted from obtained UV-Vis data (Figure S61) and Beer-Lambert law was utilised to calculate molar extinction coefficients (ε).

$$\varepsilon_{1-(4-nitrophenyl)pyrrolidine}^{434\,nm,\ THF} = \frac{0.315}{0.0325 \cdot 0.01} = 969\ \left[{}^{a.u.}/_{M \cdot cm}\right]$$

$$\varepsilon_{1-(4-nitrophenyl)piperidine}^{434\,nm,\ THF} = \frac{0.347}{0.0322 \cdot 0.01} = 1077\ \left[{}^{a.u.}/_{M \cdot cm}\right]$$

$$\varepsilon_{1-(4-nitrophenyl)azepane}^{434\,nm,\ THF} = \frac{0.395}{0.0320 \cdot 0.01} = 1235\ \left[{}^{a.u.}/_{M \cdot cm}\right]$$

2. 7. S_NAr reaction between 1-fluoro-4-nitrobenzene and pyrrolidine within reciprocating cyclic flow system with single concentration gradient

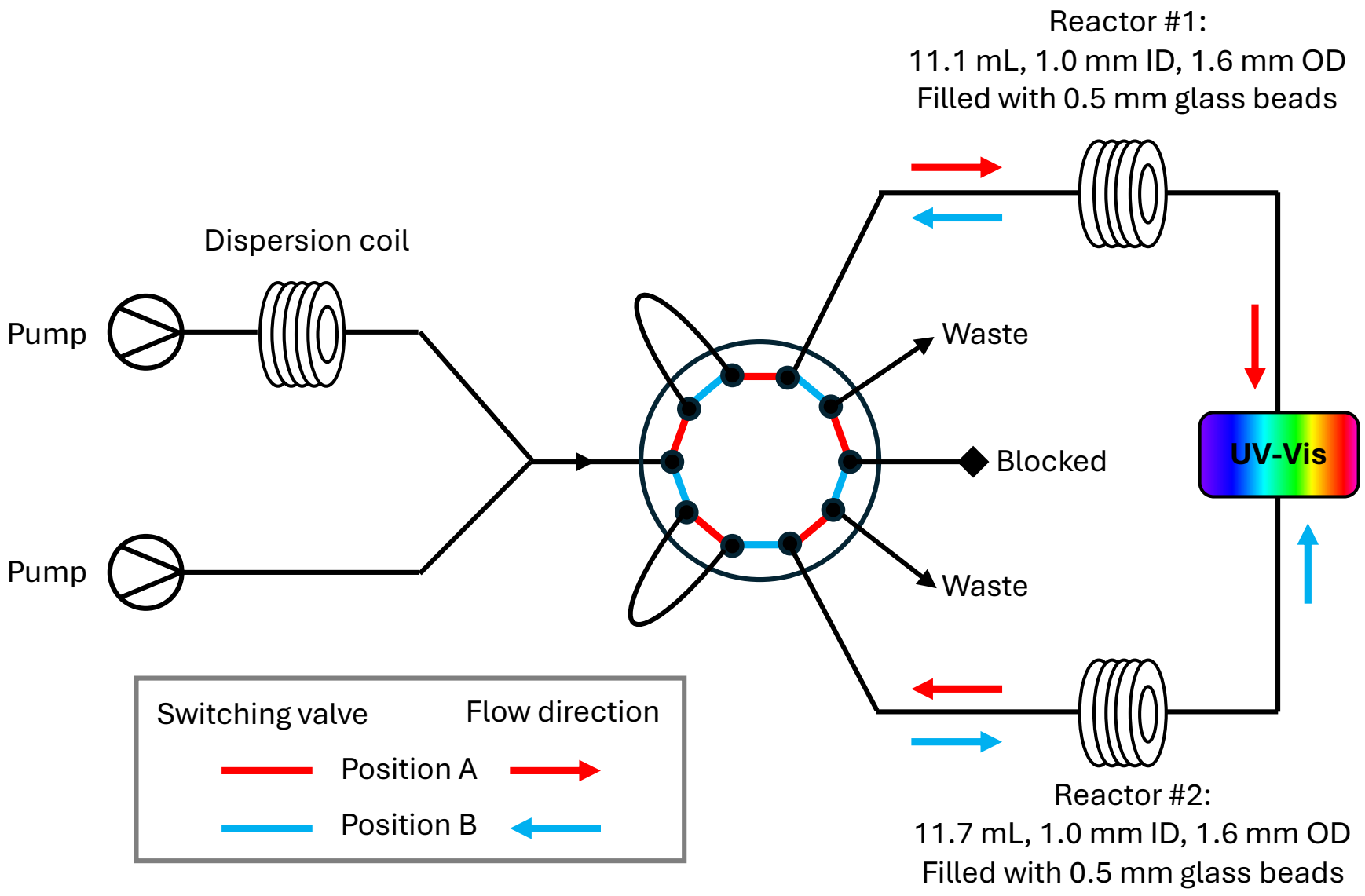

Figure S62. Reciprocating flow setup for experiments with dispersion generated concentration gradient.

1-Fluoro-4-nitrobenzene (92.0 mg, 0.65 mmol) was dissolved in THF (5.0 mL) and was attached to first reagent stream. Pyrrolidine (459.0 mg, 6.45 mmol) was dissolved in THF (5.0 mL) and was attached to second reagent stream. THF was attached to both solvent streams. Flow setup was primed with THF. Feeding valves were then switched to deliver reagents, which were directed to the reactors (1.6 mm OD, 1.0 mm ID, PFA, filled with 0.5 mm diameter glass beads, first reactor 11.1 mL and second with 11.7 mL internal void volume) heated to 75 °C with 0.5 mL·min^{-1} flow rates, for each pump. Reaction progress was monitored using UV-Vis spectrometer.

The VICI valves controlling the direction of the flow were set up using python script below, with following parameters:

- First wait = 734
- Interval time = 330
- Total time = 54000

```
import serial
import time

while True:
    try:
        VICIPort = int(input("Port number of VICI valve: "))
        if VICIPort >= 0:
            print("Port: COM" + str(VICIPort))
            break
        else:
            print("Port must be an integer")
            continue
    except ValueError:
        print("Port must be an integer")
        continue

ser = serial.Serial(
```

```
    port="COM" + str(VICIPort),
    baudrate=9600,
    parity=serial.PARITY_NONE,
    stopbits=serial.STOPBITS_ONE,
    bytesize=serial.EIGHTBITS,
)

SwitchB = [int(0x63)] + [int(0x63)] + [int(0x0D)]
SwitchA = [int(0x63)] + [int(0x77)] + [int(0x0D)]

while True:
    try:
        FirstWait = int(input("Time until first switch (seconds): "))
        if FirstWait >= 0:
            print("Port: COM" + str(VICIPort))
            print("Initial wait: " + str(FirstWait) + " seconds")
            break
        else:
            print("Time must be a positive integer")
            continue
    except ValueError:
        print("Time must be a positive integer")
        continue

while True:
    try:
        IntervalTime = int(input("Interval between valve switching
(seconds): "))
        if IntervalTime >= 0:
            print("Port: COM" + str(VICIPort))
            print("Initial wait: " + str(FirstWait) + " seconds")
            print("Interval: " + str(IntervalTime) + " seconds")
            break
        else:
            print("Port: COM" + str(VICIPort))
            print("Initial wait: " + str(FirstWait) + " seconds")
            print("Time must be a positive integer")
            continue
    except ValueError:
        print("Port: COM" + str(VICIPort))
        print("Initial wait: " + str(FirstWait) + " seconds")
        print("Time must be a positive integer")
        continue

while True:
    try:
        ReactionTime = int(input("Total reaction time (seconds): "))
        if ReactionTime >= 0:
            print("Port: COM" + str(VICIPort))
            print("Initial wait: " + str(FirstWait) + " seconds")
            print("Interval: " + str(IntervalTime) + " seconds")
            print("Total reaction time: " + str(ReactionTime) + " seconds")
            break
        else:
            print("Port: COM" + str(VICIPort))
            print("Initial wait: " + str(FirstWait) + " seconds")
            print("Interval: " + str(IntervalTime) + " seconds")
            print("Time must be a positive integer")
            continue
```

```
    except ValueError:
        print("Port: COM" + str(VICIPort))
        print("Initial wait: " + str(FirstWait) + " seconds")
        print("Interval: " + str(IntervalTime) + " seconds")
        print("Time must be a positive integer")
        continue

MaxTime = (ReactionTime - FirstWait) / (2 * IntervalTime)

ser.isOpen()

time.sleep(FirstWait)

for x in range(0, int(round(MaxTime))):
    ser.write(SwitchA)
    time.sleep(IntervalTime)

    ser.write(SwitchB)
    time.sleep(IntervalTime)

ser.close()
```

Data pre-processing was performed using following python script:

```
import openpyxl

wb = openpyxl.load_workbook('test.xlsx')
sheet = wb.active

for rowNum in range(3, sheet.max_row):
    YieldDiff = abs(sheet.cell(row=rowNum, column=12).value)
    if YieldDiff > 0.004:
        sheet['K' + str(rowNum)] = 0
        sheet['A' + str(rowNum)] = ''

# 0.01, 0.003, 0.004
for rowNum in range(3, sheet.max_row):
    Yield = abs(sheet.cell(row=rowNum, column=11).value)
    if Yield < 15.3:
        sheet['K' + str(rowNum)] = ''
        sheet['A' + str(rowNum)] = ''

wb.save('test2.xlsx')
```

The time between valve switching was determined by sending a UV active compound through the setup under the reaction conditions and monitoring when the plug passed through the in-line detector. The time was noted, and the valve manually switched to reverse the flow direction. This process was repeated until an average time for each interval could be calculated. The method was then repeated with automated control to obtain the complete dispersion profile for the desired reaction time.

2. 8. S_NAr reaction between 1-fluoro-4-nitrobenzene and pyrrolidine within unidirectional cyclic flow system

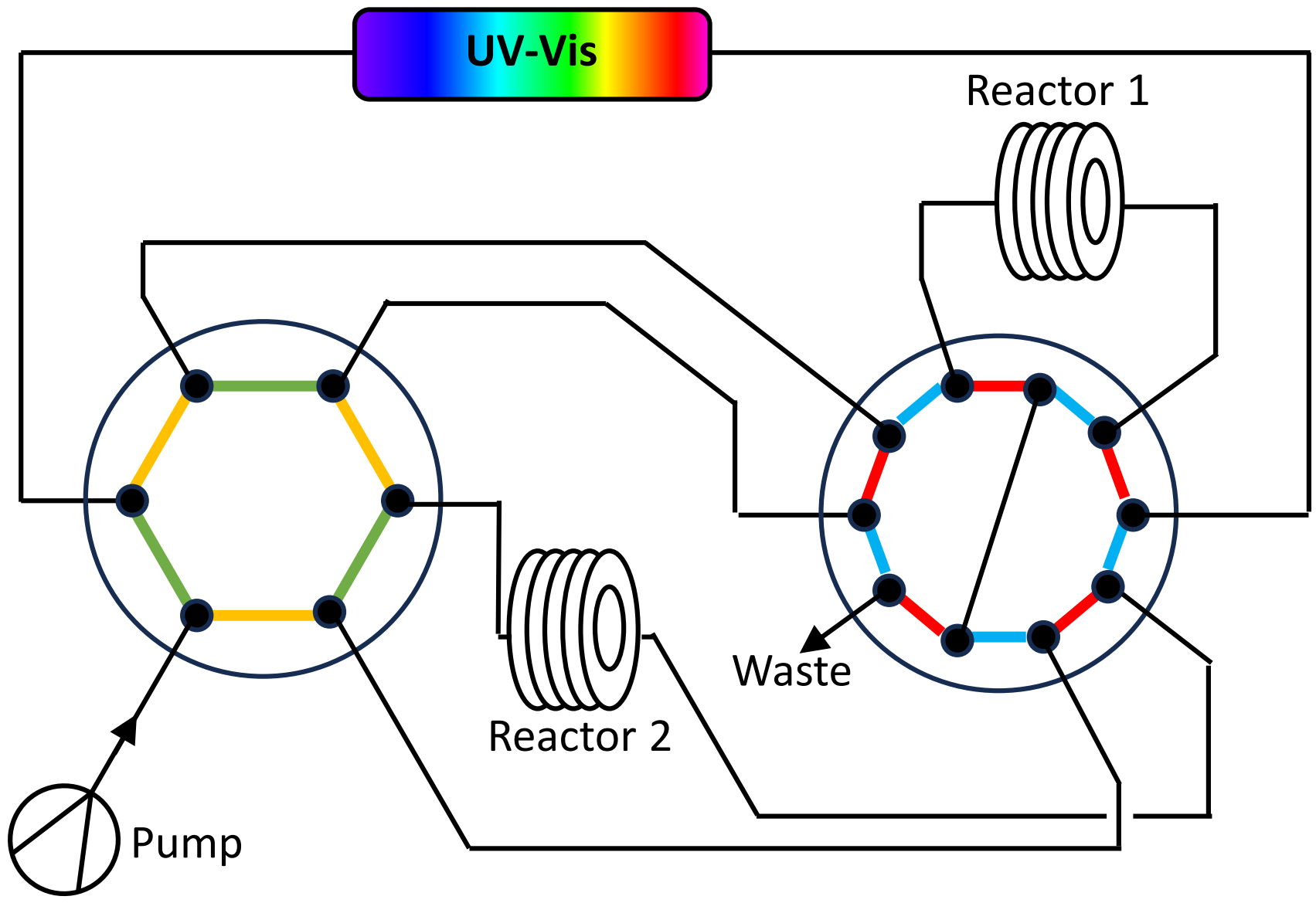

Figure S63. Flow setup for unidirectional experimentation allowing for acquisition of multiple time points in a single flow experiment. Reactor coil #1 is 11.1 mL, 1.0 mm ID, 1.6 mm OD PFA tube filled with 0.5 mm diameter glass beads. Reactor coil #2 is 11.7 mL, 1.0 mm ID, 1.6 mm OD PFA tube filled with 0.5 mm diameter glass beads.

1-Fluoro-4-nitrobenzene (93.0 mg, 0.66 mmol) was dissolved in THF (5.0 mL) and was attached to first reagent stream. Pyrrolidine (454.0 mg, 6.38 mmol) was dissolved in THF (5.0 mL) and was attached to second reagent stream. THF was attached to both solvent streams. Flow setup was primed with THF. Feeding valves were then switched to deliver reagents, which were directed to the reactors (1.6 mm OD, 1.0 mm ID, PFA, filled with 0.5 mm diameter glass beads, first reactor 11.1 mL and second with 11.7 mL internal void volume) heated to 75 °C with 1.0 mL·min^{-1} flow rates, for each pump. Reaction progress was monitored using UV-Vis spectrometer.

The VICI valves controlling the direction of the flow were set up using python script below, with following parameters:

- First wait = 373
- First interval = 353
- Second interval = 374
- Total time = 54000

```
import serial
import time

while True:
    try:
        VICIPort = int(input("Port number of VICI valve: "))
        if VICIPort >= 0:
            print("Port: COM" + str(VICIPort))
            break
        else:
            print("Port must be an integer")
            continue
    except ValueError:
```

```
        print("Port must be an integer")
        continue

ser = serial.Serial(
    port="COM" + str(VICIPort),
    baudrate=9600,
    parity=serial.PARITY_NONE,
    stopbits=serial.STOPBITS_ONE,
    bytesize=serial.EIGHTBITS,
)

SwitchBothA = [int(0x2A)] + [int(0x63)] + [int(0x77)] + [int(0x0D)]
SwitchB = [int(0x33)] + [int(0x63)] + [int(0x63)] + [int(0x0D)]
SwitchA = [int(0x33)] + [int(0x63)] + [int(0x77)] + [int(0x0D)]

while True:
    try:
        FirstWait = int(input("Time until first switch (seconds): "))
        if FirstWait >= 0:
            print("Port: COM" + str(VICIPort))
            print("Initial wait: " + str(FirstWait) + " seconds")
            break
        else:
            print("Time must be a positive integer")
            continue
    except ValueError:
        print("Time must be a positive integer")
        continue

while True:
    try:
        IntervalTime = int(input("Interval between valve switching
(seconds): "))
        if IntervalTime >= 0:
            print("Port: COM" + str(VICIPort))
            print("Initial wait: " + str(FirstWait) + " seconds")
            print("Interval: " + str(IntervalTime) + " seconds")
            break
        else:
            print("Port: COM" + str(VICIPort))
            print("Initial wait: " + str(FirstWait) + " seconds")
            print("Time must be a positive integer")
            continue
    except ValueError:
        print("Port: COM" + str(VICIPort))
        print("Initial wait: " + str(FirstWait) + " seconds")
        print("Time must be a positive integer")
        continue

while True:
    try:
        SecondIntervalTime = int(input("Interval between second valve
switch (seconds): "))
        if SecondIntervalTime >= 0:
            print("Port: COM" + str(VICIPort))
            print("Initial wait: " + str(FirstWait) + " seconds")
            print("Interval: " + str(IntervalTime) + " seconds")
            print("Second interval: " + str(SecondIntervalTime) + "
seconds")
```

```
            break
        else:
            print("Port: COM" + str(VICIPort))
            print("Initial wait: " + str(FirstWait) + " seconds")
            print("Time must be a positive integer")
            continue
    except ValueError:
        print("Port: COM" + str(VICIPort))
        print("Initial wait: " + str(FirstWait) + " seconds")
        print("Time must be a positive integer")
        continue

while True:
    try:
        ReactionTime = int(input("Total reaction time (seconds): "))
        if ReactionTime >= 0:
            print("Port: COM" + str(VICIPort))
            print("Initial wait: " + str(FirstWait) + " seconds")
            print("Interval: " + str(IntervalTime) + " seconds")
            print("Second interval: " + str(SecondIntervalTime) + "
seconds")
            print("Total reaction time: " + str(ReactionTime) + " seconds")
            break
        else:
            print("Port: COM" + str(VICIPort))
            print("Initial wait: " + str(FirstWait) + " seconds")
            print("Interval: " + str(IntervalTime) + " seconds")
            print("Second interval: " + str(SecondIntervalTime) + "
seconds")
            print("Time must be a positive integer")
            continue
    except ValueError:
        print("Port: COM" + str(VICIPort))
        print("Initial wait: " + str(FirstWait) + " seconds")
        print("Interval: " + str(IntervalTime) + " seconds")
        print("Second interval: " + str(SecondIntervalTime) + " seconds")
        print("Time must be a positive integer")
        continue

MaxTime = (ReactionTime - FirstWait) // (IntervalTime + SecondIntervalTime)

ser.isOpen()

time.sleep(FirstWait)

ser.write(SwitchBothA)

for x in range(0, int(round(MaxTime))):
    time.sleep(IntervalTime)
    ser.write(SwitchB)

    time.sleep(SecondIntervalTime)
    ser.write(SwitchA)

ser.close()
```

Pre-processing script was then applied to collected data to remove the non-steady state data utilising a dispersion profile previously obtained using the system and conditions. Experimental time was then

converted into residence time for the steady states by equating each plug pass through to the first time it is detected, minus the time taken to initially enter the multi-time point setup.

```
import openpyxl

wb = openpyxl.load_workbook('test.xlsx')
sheet = wb.active

for rowNum in range(3, sheet.max_row):
    YieldDiff = abs(sheet.cell(row=rowNum, column=12).value)
    if YieldDiff > 0.004:
        sheet['K' + str(rowNum)] = 0
        sheet['A' + str(rowNum)] = ''

# 0.01, 0.003, 0.004
for rowNum in range(3, sheet.max_row):
    Yield = abs(sheet.cell(row=rowNum, column=11).value)
    if Yield < 15.3:
        sheet['K' + str(rowNum)] = ''
        sheet['A' + str(rowNum)] = ''

wb.save('test2.xlsx')
```

The time between valve switching was determined by sending a UV active compound through the setup under the reaction conditions and monitoring when the plug passed through the in-line detector. The time was noted, and the valve manually switched to reverse the flow direction. This process was repeated until an average time for each interval could be calculated. The method was then repeated with automated control to obtain the complete dispersion profile for the desired reaction time.

2. 9. S_NAr reaction between 1-fluoro-4-nitrobenzene and pyrrolidine within unidirectional cyclic flow system utilising single dispersion-generated concentration gradient

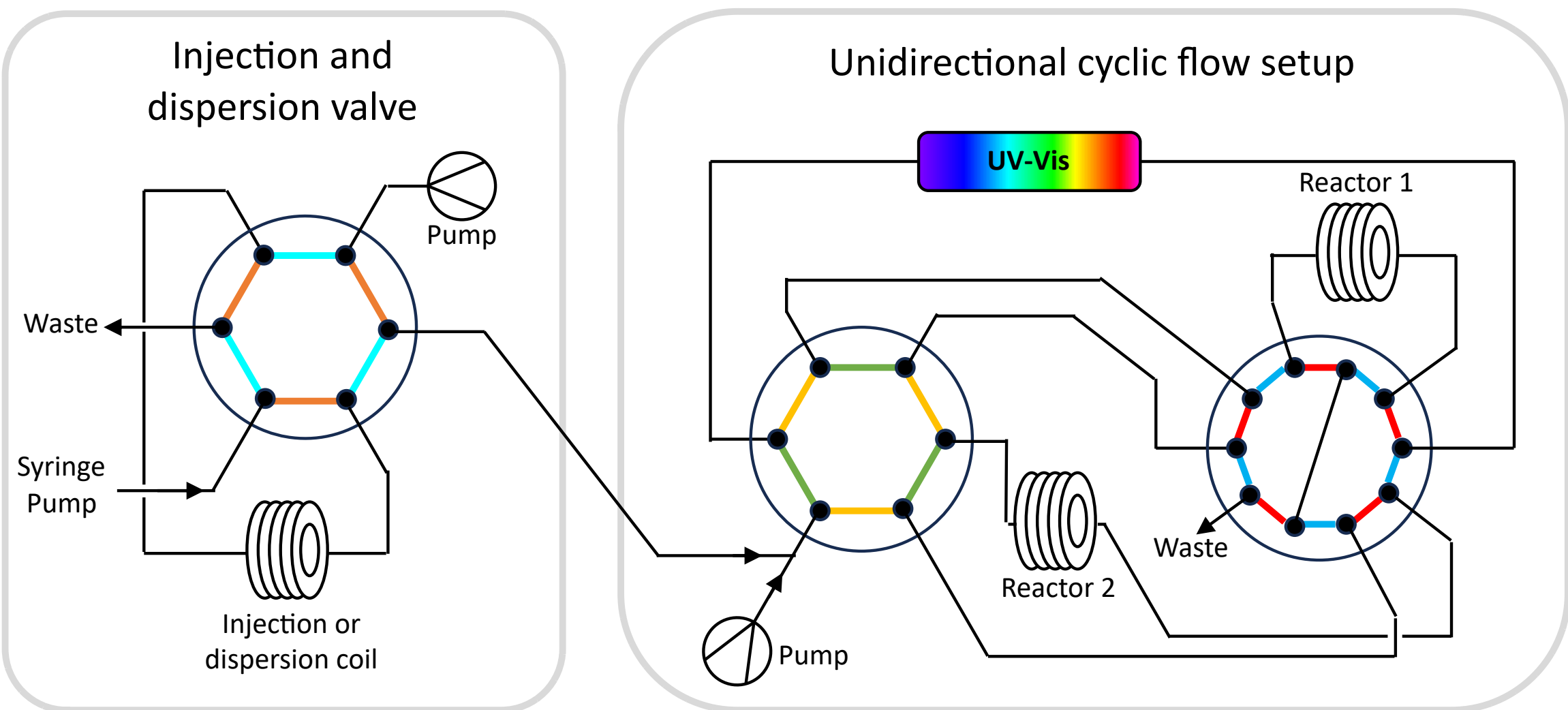

Figure S64. Flow setup used for experiments involving dispersion generated concentration gradient studies within unidirectional cyclic flow setup.

1-Fluoro-4-nitrobenzene (184.0 mg, 1.30 mmol) was dissolved in THF (10.0 mL) and was attached to the steady-state reagent line. Pyrrolidine (683.0 mg, 9.60 mmol) was dissolved in THF (5.0 mL) and

was attached to dispersed reagent stream. THF was attached to both solvent streams. Flow setup was primed with THF. Feeding valves were then switched to deliver reagents, which were directed to the reactors (1.6 mm OD, 1.0 mm ID, PFA, filled with 0.5 mm diameter glass beads, first reactor 11.1 mL and second with 11.7 mL internal void volume) heated to 75 °C with 1.0 mL·min^{-1} flow rates, for each pump. Reaction progress was monitored using UV-Vis spectrometer.

The VICI valves controlling the direction of the flow were set up using python script below, with following parameters:

- First wait = 885
- Gradient clip = 840
- First interval = 350
- Second interval = 370
- Total time = 54000

```
import serial
import time

while True:
    try:
        VICIPort = int(input("Port number of VICI valve: "))
        if VICIPort >= 0:
            print("Port: COM" + str(VICIPort))
            break
        else:
            print("Port must be an integer")
            continue
    except ValueError:
        print("Port must be an integer")
        continue

ser = serial.Serial(
    port="COM" + str(VICIPort),
    baudrate=9600,
    parity=serial.PARITY_NONE,
    stopbits=serial.STOPBITS_ONE,
    bytesize=serial.EIGHTBITS,
)

SwitchBothA = [int(0x2A)] + [int(0x63)] + [int(0x77)] + [int(0x0D)]
SwitchB = [int(0x33)] + [int(0x63)] + [int(0x63)] + [int(0x0D)]
SwitchA = [int(0x33)] + [int(0x63)] + [int(0x77)] + [int(0x0D)]
GradientClip = [int(0x32)] + [int(0x63)] + [int(0x77)] + [int(0x0D)]

while True:
    try:
        FirstWait = int(input("Time until first switch (seconds): "))
        if FirstWait >= 0:
            print("Port: COM" + str(VICIPort))
            print("Initial wait: " + str(FirstWait) + " seconds")
            break
        else:
            print("Time must be a positive integer")
            continue
    except ValueError:
        print("Time must be a positive integer")
        continue
```

```
while True:
    try:
        GradClipWait = int(input("Time until gradient clip (seconds): "))
        if GradClipWait >= 0:
            print("Port: COM" + str(VICIPort))
            print("Initial wait: " + str(FirstWait) + " seconds")
            print("Gradient Clip wait: " + str(GradClipWait) + " seconds")
            break
        else:
            print("Time must be a positive integer")
            continue
    except ValueError:
        print("Time must be a positive integer")
        continue

while True:
    try:
        IntervalTime = int(input("Interval between valve switching
(seconds): "))
        if IntervalTime >= 0:
            print("Port: COM" + str(VICIPort))
            print("Initial wait: " + str(FirstWait) + " seconds")
            print("Gradient Clip wait: " + str(GradClipWait) + " seconds")
            print("Interval: " + str(IntervalTime) + " seconds")
            break
        else:
            print("Port: COM" + str(VICIPort))
            print("Initial wait: " + str(FirstWait) + " seconds")
            print("Gradient Clip wait: " + str(GradClipWait) + " seconds")
            print("Time must be a positive integer")
            continue
    except ValueError:
        print("Port: COM" + str(VICIPort))
        print("Initial wait: " + str(FirstWait) + " seconds")
        print("Gradient Clip wait: " + str(GradClipWait) + " seconds")
        print("Time must be a positive integer")
        continue

while True:
    try:
        SecondIntervalTime = int(input("Interval between second valve
switch (seconds): "))
        if SecondIntervalTime >= 0:
            print("Port: COM" + str(VICIPort))
            print("Initial wait: " + str(FirstWait) + " seconds")
            print("Gradient Clip wait: " + str(GradClipWait) + " seconds")
            print("Interval: " + str(IntervalTime) + " seconds")
            print("Second interval: " + str(SecondIntervalTime) + "
seconds")
            break
        else:
            print("Port: COM" + str(VICIPort))
            print("Initial wait: " + str(FirstWait) + " seconds")
            print("Gradient Clip wait: " + str(GradClipWait) + " seconds")
            print("Interval: " + str(IntervalTime) + " seconds")
            print("Time must be a positive integer")
            continue
    except ValueError:
```

```
        print("Port: COM" + str(VICIPort))
        print("Initial wait: " + str(FirstWait) + " seconds")
        print("Gradient Clip wait: " + str(GradClipWait) + " seconds")
        print("Interval: " + str(IntervalTime) + " seconds")
        print("Time must be a positive integer")
        continue

while True:
    try:
        ReactionTime = int(input("Total reaction time (seconds): "))
        if ReactionTime >= 0:
            print("Port: COM" + str(VICIPort))
            print("Initial wait: " + str(FirstWait) + " seconds")
            print("Gradient Clip wait: " + str(GradClipWait) + " seconds")
            print("Interval: " + str(IntervalTime) + " seconds")
            print("Second interval: " + str(SecondIntervalTime) + "
seconds")
            print("Total reaction time: " + str(ReactionTime) + " seconds")
            break
        else:
            print("Port: COM" + str(VICIPort))
            print("Initial wait: " + str(FirstWait) + " seconds")
            print("Gradient Clip wait: " + str(GradClipWait) + " seconds")
            print("Interval: " + str(IntervalTime) + " seconds")
            print("Second interval: " + str(SecondIntervalTime) + "
seconds")
            print("Time must be a positive integer")
            continue
    except ValueError:
        print("Port: COM" + str(VICIPort))
        print("Initial wait: " + str(FirstWait) + " seconds")
        print("Gradient Clip wait: " + str(GradClipWait) + " seconds")
        print("Interval: " + str(IntervalTime) + " seconds")
        print("Second interval: " + str(SecondIntervalTime) + " seconds")
        print("Time must be a positive integer")
        continue

MaxTime = (ReactionTime - FirstWait) / (IntervalTime + SecondIntervalTime)
TimeGap = FirstWait - GradClipWait

ser.isOpen()

time.sleep(GradClipWait)

ser.write(GradientClip)

time.sleep(TimeGap)

ser.write(SwitchBothA)

for x in range(0, int(round(MaxTime))):
    time.sleep(IntervalTime)
    ser.write(SwitchB)

    time.sleep(SecondIntervalTime)
    ser.write(SwitchA)

ser.close()
```

Pre-processing script was then applied to collected data in order to remove parts of data originating from outside of the pass through, by utilising a dispersion profile previously obtained using the same system and conditions. Experimental time was then converted into residence time for the gradients by equating each plug pass through to the first time it is detected, minus the time taken to initially enter the multi-time point setup.

```
import openpyxl

wb = openpyxl.load_workbook('test.xlsx')
# test.xlsx in Users folder. Can be changed by altering the path in python
sheet = wb.active

rowNum3 = 3
# Row number currently worked on after deletion
x = 3
# Row number if none deleted

while rowNum3 < sheet.max_row:
    YieldDiff = sheet.cell(row=rowNum3, column=26).value
    # Z column on excel sheet, difference between neighbouring cells
    Absorbance = sheet.cell(row=rowNum3, column=25).value
    # Y column on excel sheet, spectrometer reading
    if YieldDiff < 0.001 and Absorbance < 3:
        print(rowNum3, x)
        sheet.delete_rows(rowNum3, 1)
        rowNum3 = rowNum3 - 1
        x = x + 1
    else:
        rowNum3 = rowNum3 + 1
        x = x + 1

# Goes through every cell in Z and Y column and deletes the row if
parameter not met.

wb.save('test2.xlsx')
```

The correct timing for the gradient clipping was determined by attaching the UV-Vis cell directly to the output of the clipping valve and pumping a UV active compound through the system using the same flow parameters. The resulting dispersion profile was used to calculate the clipping time.

2. 10. S_NAr reaction between 1-fluoro-4-nitrobenzene and pyrrolidine, piperidine or azepane within unidirectional cyclic flow system utilising multiple dispersion-generated concentration gradients with gradient clipping

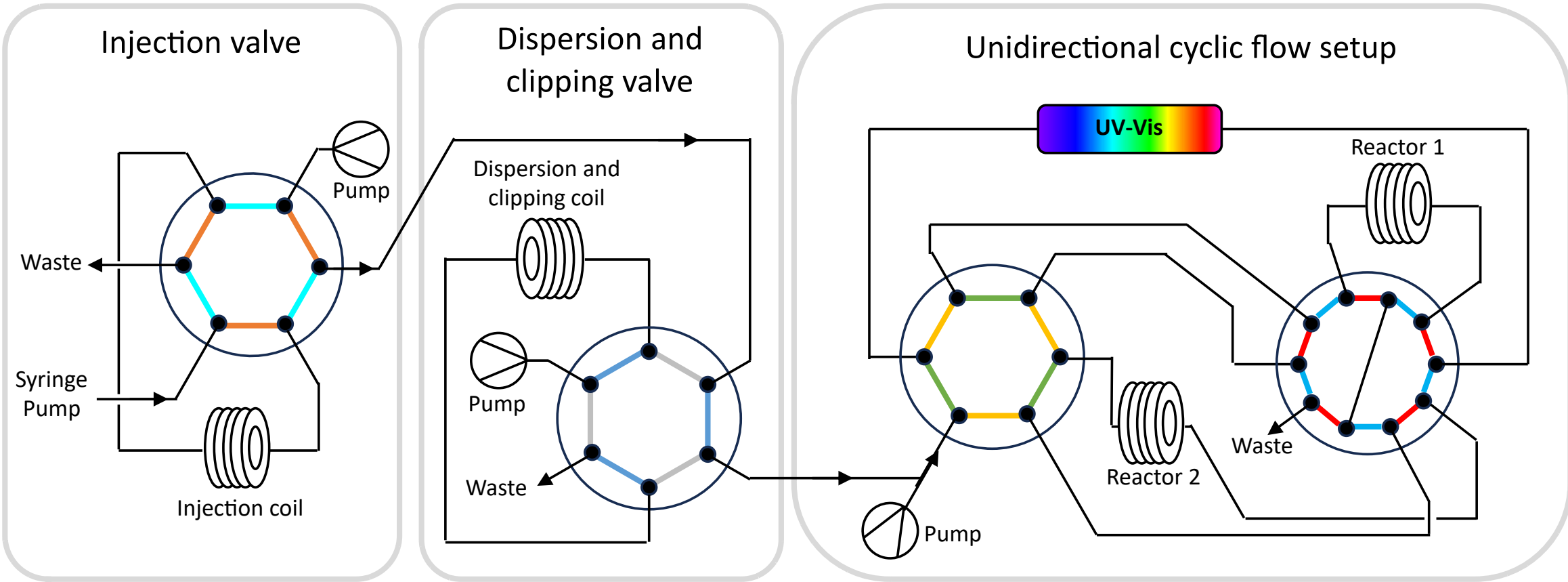

Figure S65. Flow setup used for experiments involving dispersion generated concentration gradient of multiple reactants within unidirectional cyclic flow setup.

1-Fluoro-4-nitrobenzene (184.0 mg, 1.30 mmol) was dissolved in THF (10.0 mL) and was attached to the steady-state reagent line. Pyrrolidine (0.27 mL, 3.3 mmol) was dissolved in THF (5.0 mL) and was infused via syringe into the injection coil. THF was attached to both solvent streams. Flow setup was primed with THF. Dispersed reagents were pumped to the dispersion coil (1.0 mm ID, 1.6 mm OD, 2.6 mL, PFA) and then reactors (1.6 mm OD, 1.0 mm ID, PFA, filled with 0.5 mm diameter glass beads, first reactor 11.1 mL and second with 11.7 mL internal void volume) heated to 75 °C with 1.0 mL·min^{-1} flow rate. Steady state reagent was pumped with 1.0 mL·min^{-1} flow rate. The gradient clipping pump was set to 0.5 mL·min^{-1}. Reaction progress was monitored using UV-Vis spectrometer.

The VICI valves were controlled manually (valves position colours as on Figure S65):

Experimental time / min:seconds	Action
**00:00**	Experiment start
**00:45**	Injection valve switched to brown position; sample loop filled with piperidine (0.32 mL, 3.3 mmol) in THF (5.0 mL).
**01:15**	Sample loop valve switched to teal position
**02:00**	Injection valve switched to brown position; sample loop filled with azepane (0.37 mL, 3.3 mmol) in THF (5.0 mL).
**02:30**	Injection valve switched to teal position
**03:15**	Injection valve switched to brown position
**03:20**	Gradient clipping valve switched to blue position
**04:20**	Gradient clipping valve switched to grey position
**05:05**	Gradient clipping valve switched to blue position
**06:10**	Gradient clipping valve switched to grey position
**06:55**	Gradient clipping valve switched to blue position
**07:20**	Unidirectional valve switched to green position
**13:45**	10-port directional valve switched to light blue position
**20:02**	10-port directional valve switched to red position
**26:30**	10-port directional valve switched to light blue position

**32:55**	10-port directional valve switched to red position
**39:15**	10-port directional valve switched to light blue position. Reaction mixture allowed to be flushed out to waste.

Pre-processing script was then applied to collected data in order to remove parts of data originating from outside of the pass through, by utilising a dispersion profile previously obtained using the same system and conditions. Experimental time was then converted into residence time for the gradients by equating each plug pass through to the first time it is detected, minus the time taken to initially enter the multi-time point setup. Concentration gradients corresponding to each dispersed reactant (pyrrolidine, piperidine, azepane) were separated into respective workbooks.

```
import openpyxl

wb = openpyxl.load_workbook('test.xlsx')
# test.xlsx in Users folder. Can be changed by altering the path in python
sheet = wb.active

rowNum3 = 3
# Row number currently worked on after deletion
x = 3
# Row number if none deleted

while rowNum3 < sheet.max_row:
    YieldDiff = sheet.cell(row=rowNum3, column=26).value
    # Z column on excel sheet, difference between neighbouring cells
    Absorbance = sheet.cell(row=rowNum3, column=25).value
    # Y column on excel sheet, spectrometer reading
    if YieldDiff < 0.001 and Absorbance < 3:
        print(rowNum3, x)
        sheet.delete_rows(rowNum3, 1)
        rowNum3 = rowNum3 - 1
        x = x + 1
    else:
        rowNum3 = rowNum3 + 1
        x = x + 1

# Goes through every cell in Z and Y column and deletes the row if
parameter not met.

wb.save('test2.xlsx')
```

The correct timing for the gradient clipping was determined by attaching the UV-Vis cell directly to the output of the clipping valve and pumping a UV active compound through the system using the same flow parameters. The resulting dispersion profile was used to calculate the clipping time.

2. 11. Procedure for validation of S_NAr between 1-fluoro-4-nitrobenzene and pyrrolidine reaction using steady-state conditions

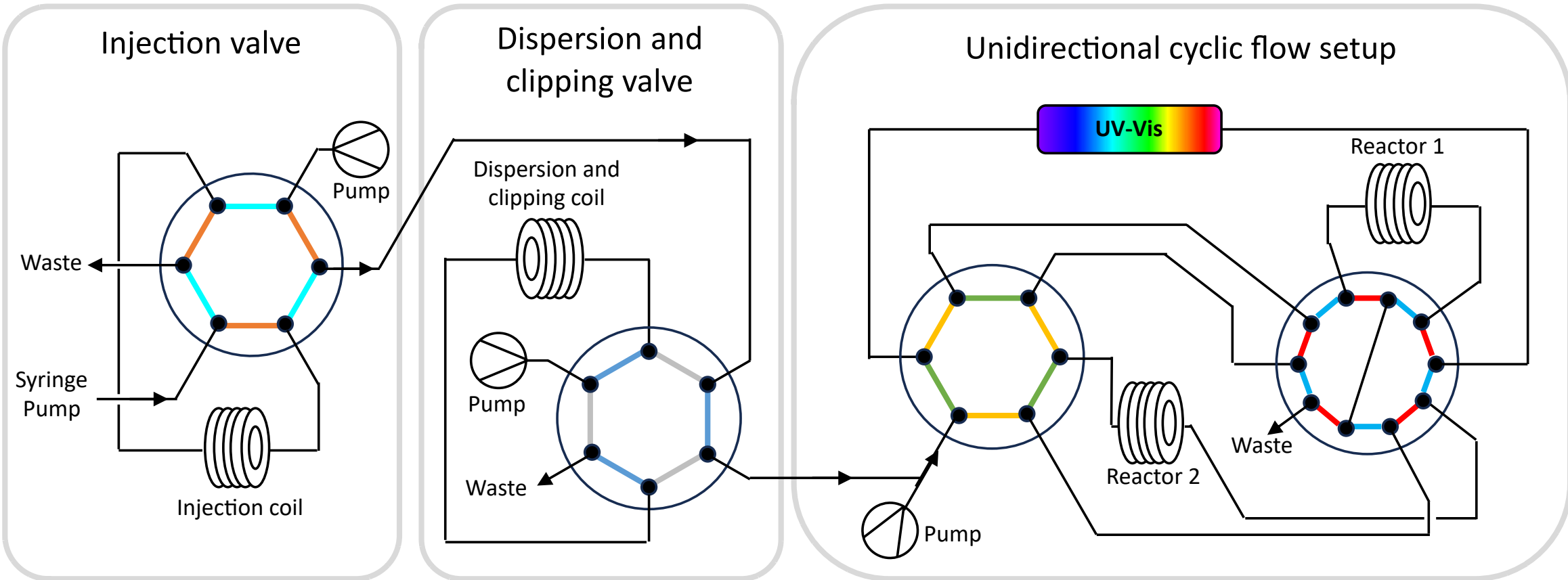

Figure S66. Flow setup used for validation of results obtained from experiments involving multiple concentration gradients within unidirectional flow system.

1-Fluoro-4-nitrobenzene (47.0 mg, 0.325 mmol) was dissolved in THF (5.0 mL) and was attached to the steady-state reagent line. Pyrrolidine (43.5 mg, 0.65 mmol) was dissolved in THF (5.0 mL) and was attached to dispersed reagent stream. THF was attached to both solvent streams. Flow setup was primed with THF. Reagents were directed to the reactors (1.6 mm OD, 1.0 mm ID, PFA, filled with 0.5 mm diameter glass beads, first reactor 11.1 mL and second with 11.7 mL internal void volume) heated to 75 °C with 1.0 mL·min^{-1} flow rates for injection and steady state reagent pumps, and 0.5 mL·min^{-1} for gradient clipping pump. Reaction progress was monitored using UV-Vis spectrometer.

The VICI valves were controlled manually (valves position colours as on Figure S65):

Experimental time / min:seconds	Action
**00:00**	Experiment start
**00:45**	Injection valve switched to brown position; sample loop filled with pyrrolidine solution.
**06:00**	Unidirectional valve switched to green and 10-position directional valve switched to blue
**13:15**	10-position directional valve switched to red
**20:03**	10-position directional valve switched to blue
**27:06**	10-position directional valve switched to red
**33:54**	10-position directional valve switched to red
**41:03**	10-position directional valve switched to blue, flow system flushed with THF

Such protocol allowed for acquisition of datapoints with 6, 13.25, 20.05, 27.1, 33.5, 41.05 min residence times for the studied pyrrolidine concentration gradient. Whole protocol was then repeated twice, with infusion of 5.0 eq. of pyrrolidine (solution made by mixing 111.8 mg, 1.625 mmol of pyrrolidine with 5.0 mL THF) and 8.0 eq. of pyrrolidine (solution made by mixing 189.2 mg, 2.6 mmol of pyrrolidine with 5.0 mL of THF) into the sample loop, respectively.

3. Dispersion in glass bead packed tubular reactors

Glass-bead filled tubular reactors were investigated as a simple method for reducing dispersion in meso-scale flow systems. In an empty tubular reactor operated under laminar flow, an injected plug spreads as a result of the parabolic velocity profile across the tube. Introducing an inert packed bed disrupts this velocity profile and promotes radial mixing, thereby narrowing the residence-time distribution and reducing plug broadening.

The reactors used in this work were prepared by packing 1.0 mm ID PFA tubing with 0.5 mm diameter glass beads (Sigma-Aldrich Z259465) (Figure S67). Glass beads were selected because they are chemically inert under the conditions examined, readily available, inexpensive, and sufficiently uniform in size to allow reproducible packing. The bead diameter was chosen to be smaller than the tube internal diameter while avoiding excessive pressure drop or frequent blockage.

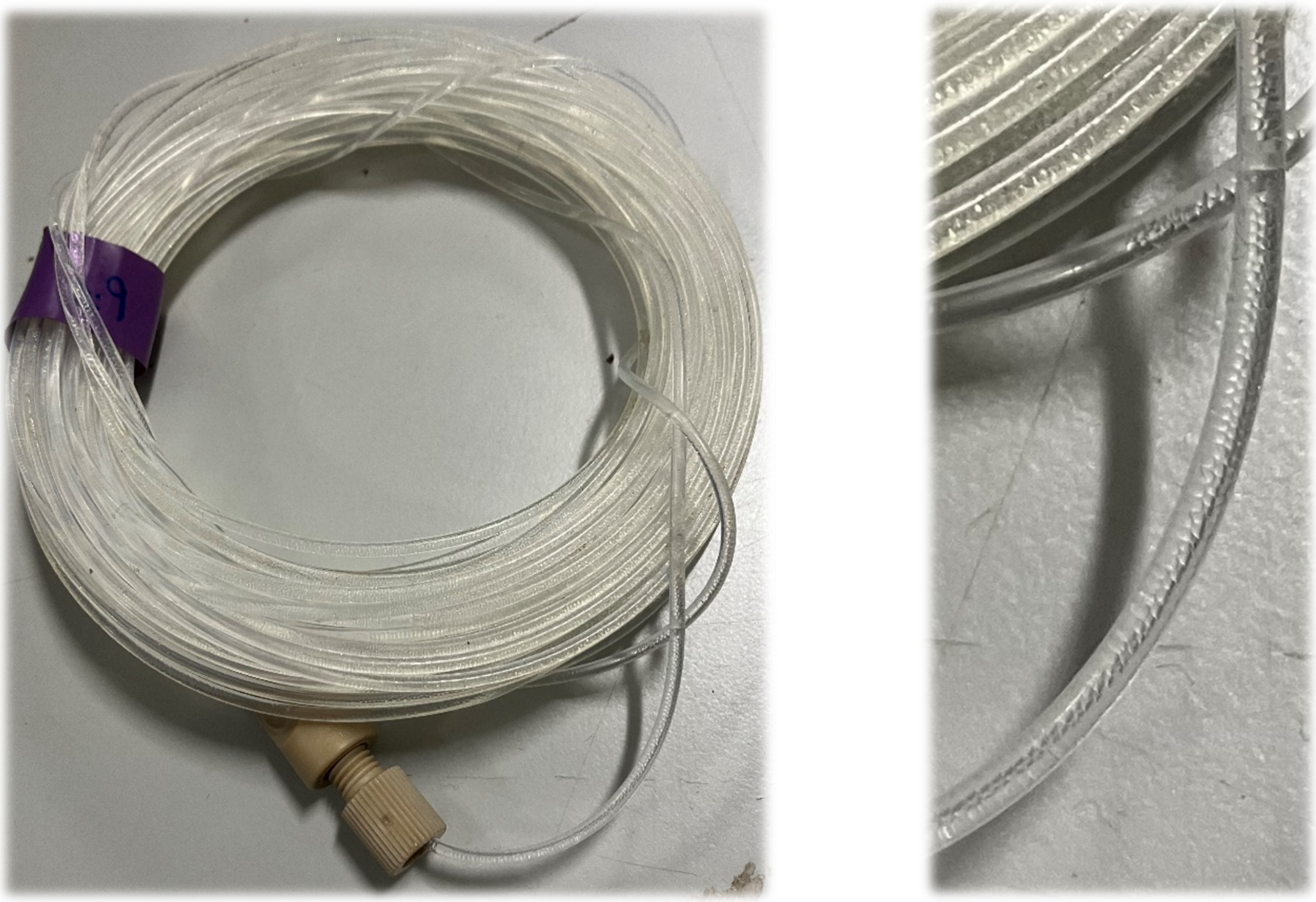

Figure S67. Glass-bead (0.5 mm diameter) filled tubular reactor (1.0 mm ID).

To make the packed tubes a syringe barrel containing glass beads was connected to the PFA tubing using a Luer adapter, the whole filled with water, and the beads encouraged to enter the tubing by agitation. The presence of water reduced bead bridging and made blockages easier to clear. Manual agitation of the syringe allowed short bead-filled reactors to be prepared, while longer reactors (up to 15 m) could be produced by suspending the tubing vertically and applying mechanical vibration to the bead reservoir. After packing, the reactors were flushed thoroughly with solvent before use.

The packed reactors were characterised by their accessible internal volume rather than the geometric volume of the empty tubing, since the glass beads occupy a significant fraction of the tube. Reactor volume was determined experimentally by measuring the volume required to fill or displace solvent from the packed reactor.

Dispersion in flow reactors filled with glass beads was compared with empty tubular reactors by injecting a 0.1 mL plug of an UV active tracer (diphenylfulvene) in DMF using an injection loop. Tracer plug was subsequently infused into the tubular reactor and the flow cell of a UV spectrometer monitoring the signal at 300 nm. The output profile from the packed tubular reactors was compared against both a no-reactor reference and an empty tubular reactor of comparable volume (both approx. 0.9 mL) for flow rates between 0.1 and 10 mL/min (Figure S68).

The glass-bead-filled reactors gave markedly narrower dispersion profiles than equivalent empty tubular reactors, particularly at flows of 1 mL/min and above. At 10 mL/min there is little difference from the peak produced when no tubular reactor is present.

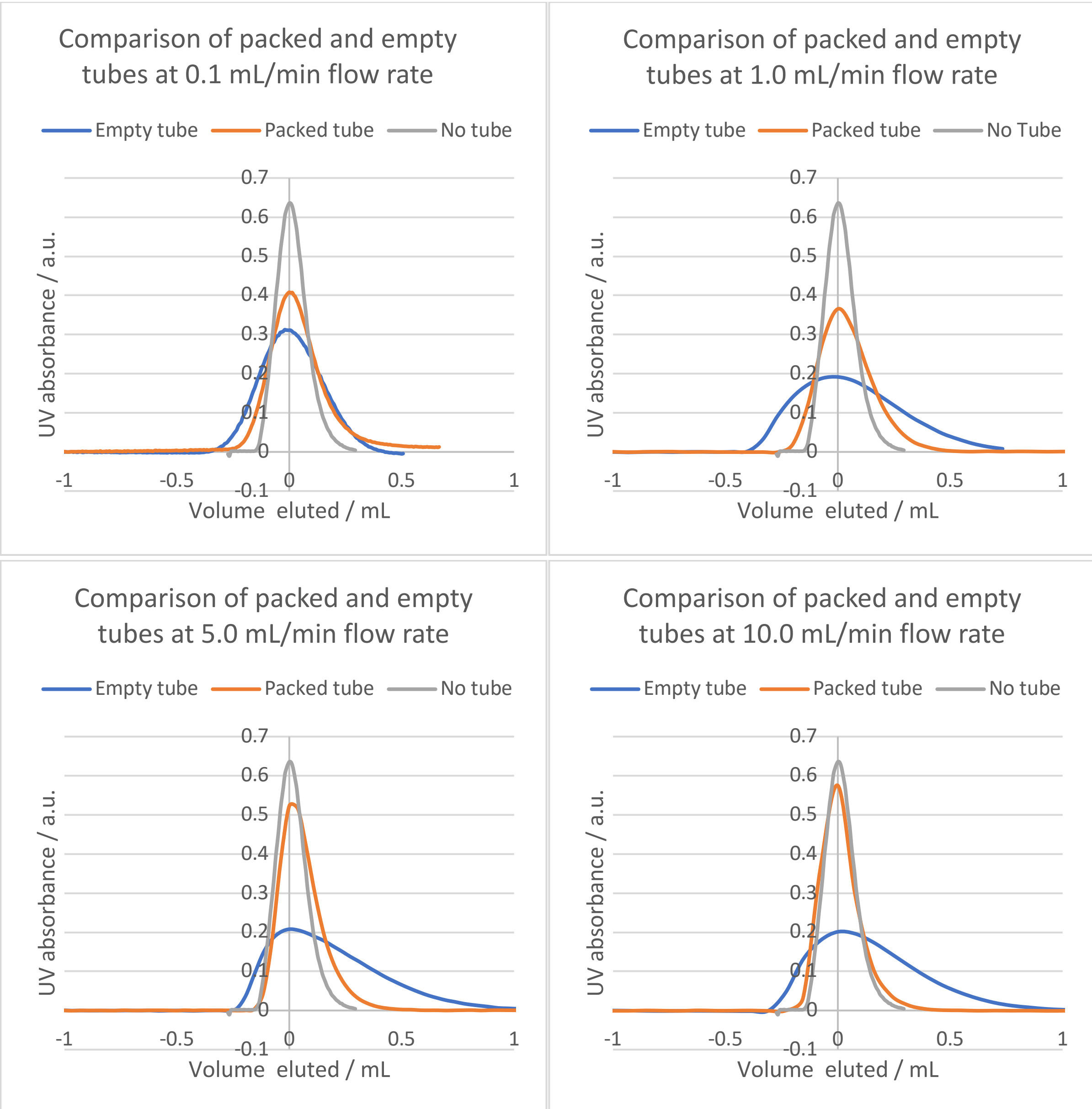

Figure S68. Dispersion in empty and packed tubular reactors. The 'no reactor' trace was from the 0.1 mL/min flow rate in all cases.

Although glass-bead-filled reactors were effective at reducing dispersion, they also introduced a higher-pressure drop than empty tubular reactors. The pressure increase was more significant at high

flow rates, with viscous solvents and for longer packed reactors. For this reason, the reactor length, bead size and flow rate must be selected carefully to remain within the safe operating pressure of the flow system and the tubing material.

The packed reactors were particularly useful in the multi-time-point and gradient-clipping experiments, where preservation of the concentration profile over repeated passes through the reactor was important.

A practical limitation was observed when bead-filled PFA reactors were used at elevated temperature and pressure. Under these conditions, slight expansion and softening of the PFA tubing may allow the beads to rearrange. In some cases, this can alter the packing density or generate local restrictions leading to pressure instability or blockage. For demanding conditions, alternative materials such as stainless-steel tubing may provide improved mechanical stability, although visual inspection of the packing is then impossible.

4. Syntheses and characterization data

4. 1. Methyl 4-methylcyclohex-3-ene-1-carboxylate (6a) and methyl 3-methylcyclohex-3-ene-1-carboxylate (6b):

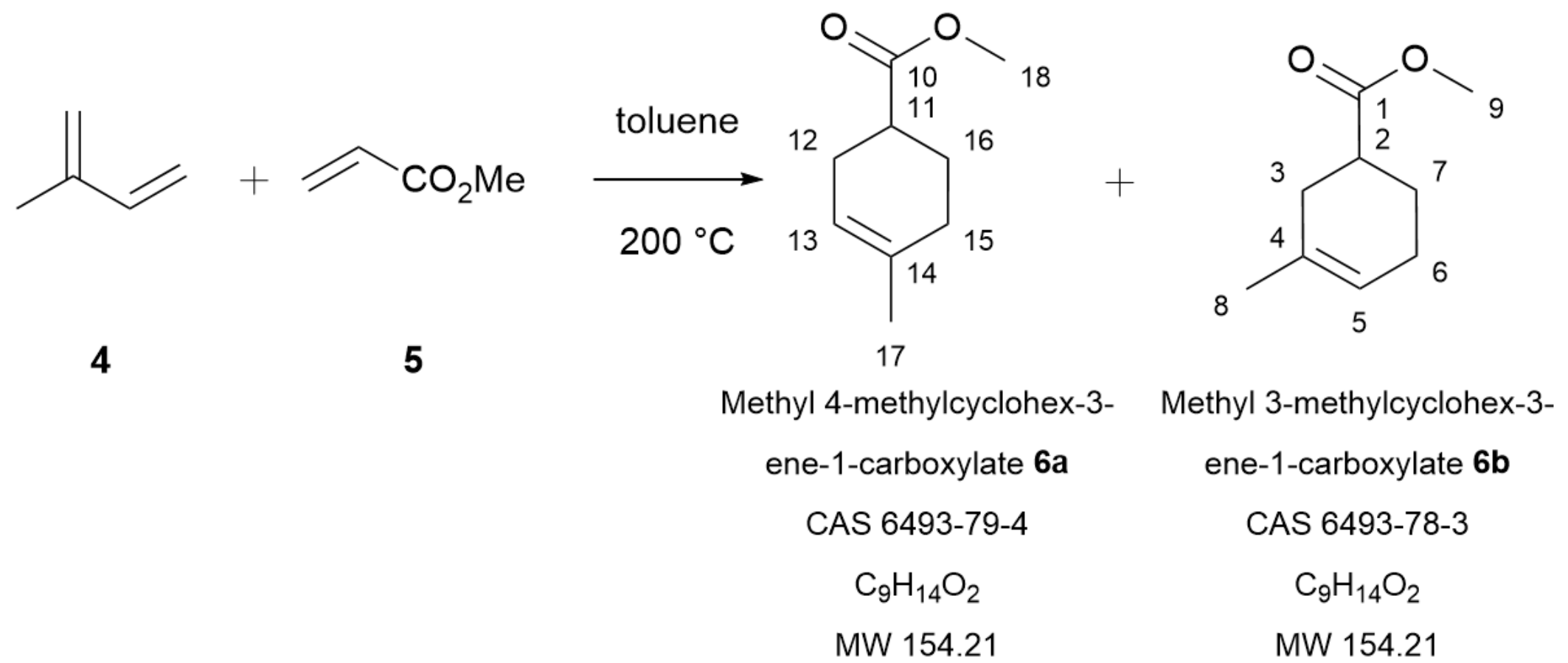

*Figure S69. Diels-Alder reaction of isoprene **4** and methyl acrylate **5** affording products **6a** and **6b**.*

In a RBF flushed with nitrogen, isoprene (0.396 mL, 3.96 mmol, 1.0 eq.) and methyl acrylate (0.357 mL, 3.96 mmol, 1.0 eq.) were mixed with dry toluene (1.24 mL). The solution was degassed by sonication whilst being saturated with nitrogen and injected into the flow system previously primed with toluene. The flow rate was set to 0.2 mL/min and the 10 mL length Stainless Steel reactor, connected to a cooling loop (100 cm, 1 mm ID) followed by a backpressure regulator (250 psi), was heated to 200 °C. After collection and evaporation of the solvent under reduced pressure, the crude product was purified using flash chromatography (SiO_2, DCM) to give a mixture of the title compounds **6a** and **6b** (295 mg, 1.91 mmol, 48%) as a yellow oil. The ^{1}H NMR identified the ratio of 3-methyl to 4-methyl isomers as 65/35. NMR data are consistent with the literature.[2–4]

Methyl 4-methylcyclohex-3-ene-1-carboxylate (**6a**):

^{1}H NMR (400 MHz, $CDCl_3$) δ ppm: 5.38 (br s, 1H), 3.68 (s, 3H), 2.53-2.45 (m, 1H), 2.25-2.19 (m, 2H), 2.03-1.96 (m, 3H), 1.77-1.69 (m, 1H), 1.65 (s, 3H).

^{13}C NMR (100 MHz, $CDCl_3$) δ ppm: 176.5 (C), 133.7 (C), 119.2 (CH), 51.6 (CH_3), 39.1 (CH), 29.3 (CH_2), 27.7 (CH_2), 25.5 (CH_2), 23.4 (CH_3).

Methyl 3-methylcyclohex-3-ene-1-carboxylate (6b):

^{1}H NMR (400 MHz, $CDCl_3$) δ ppm: 5.38 (br s, 1H), 3.69 (s, 3H), 2.62-2.54 (m, 1H), 2.18-2.10 (m, 2H), 2.10-2.04 (m, 2H), 1.96-1.91 (m, 1H), 1.67 (s, 3H), 1.63-1.56 (m, 1H).

^{13}C NMR (100 MHz, $CDCl_3$) δ ppm: 176.4 (C), 132.3 (C), 120.6 (CH), 51.6 (CH3), 39.8 (CH), 32.1 (CH_2), 24.9 (CH_2), 24.5 (CH_2), 23.5 (CH_3).

IR (MeCN) ν max / cm^{-1} = 2927 (br), 1733 (s), 1435 (m), 1305 (w), 1223 (m), 1165 (s), 1025 (w).

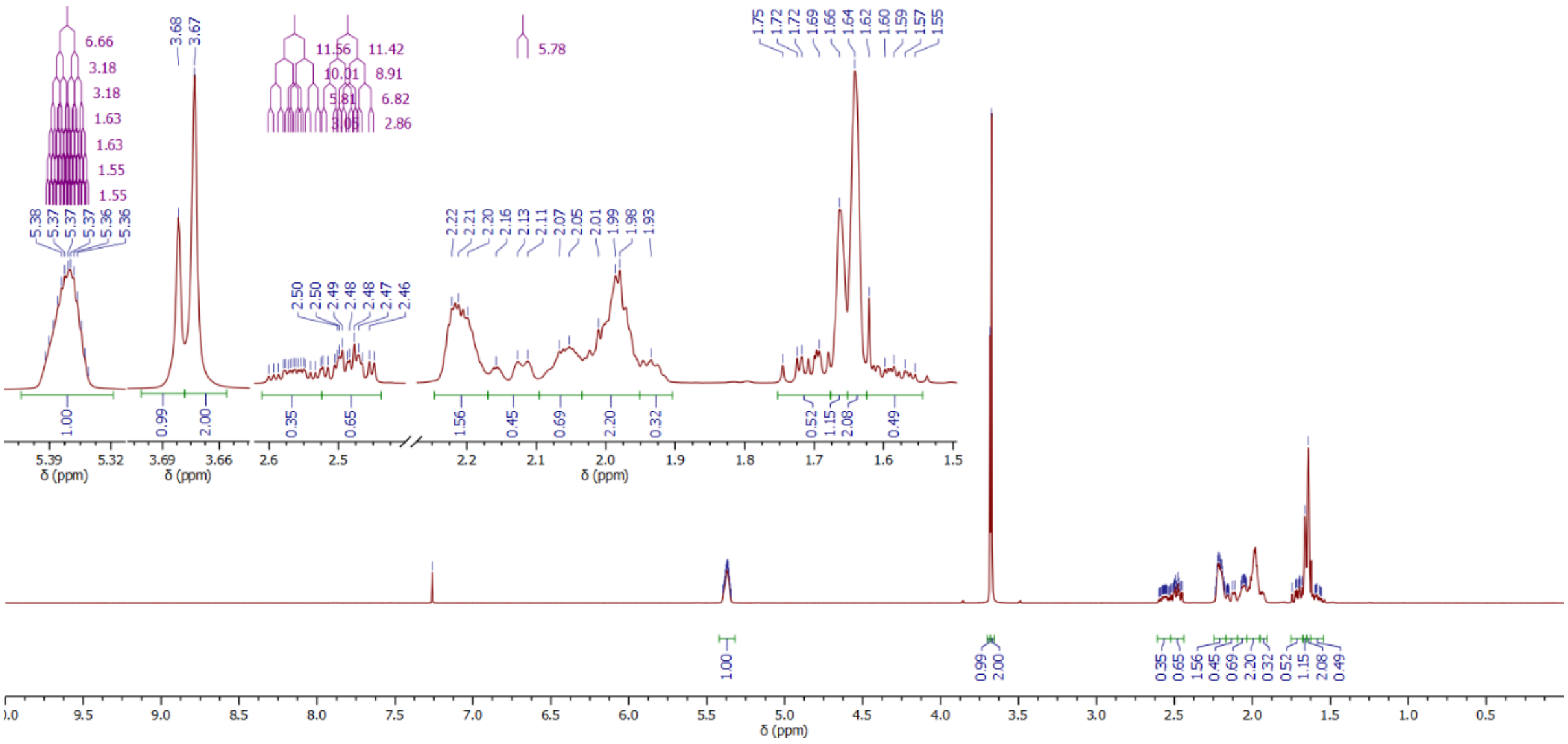

*Figure S70. ^{1}H NMR spectrum of **6a** and **6b** (2:1 ratio) mixture in $CDCl_3$.*

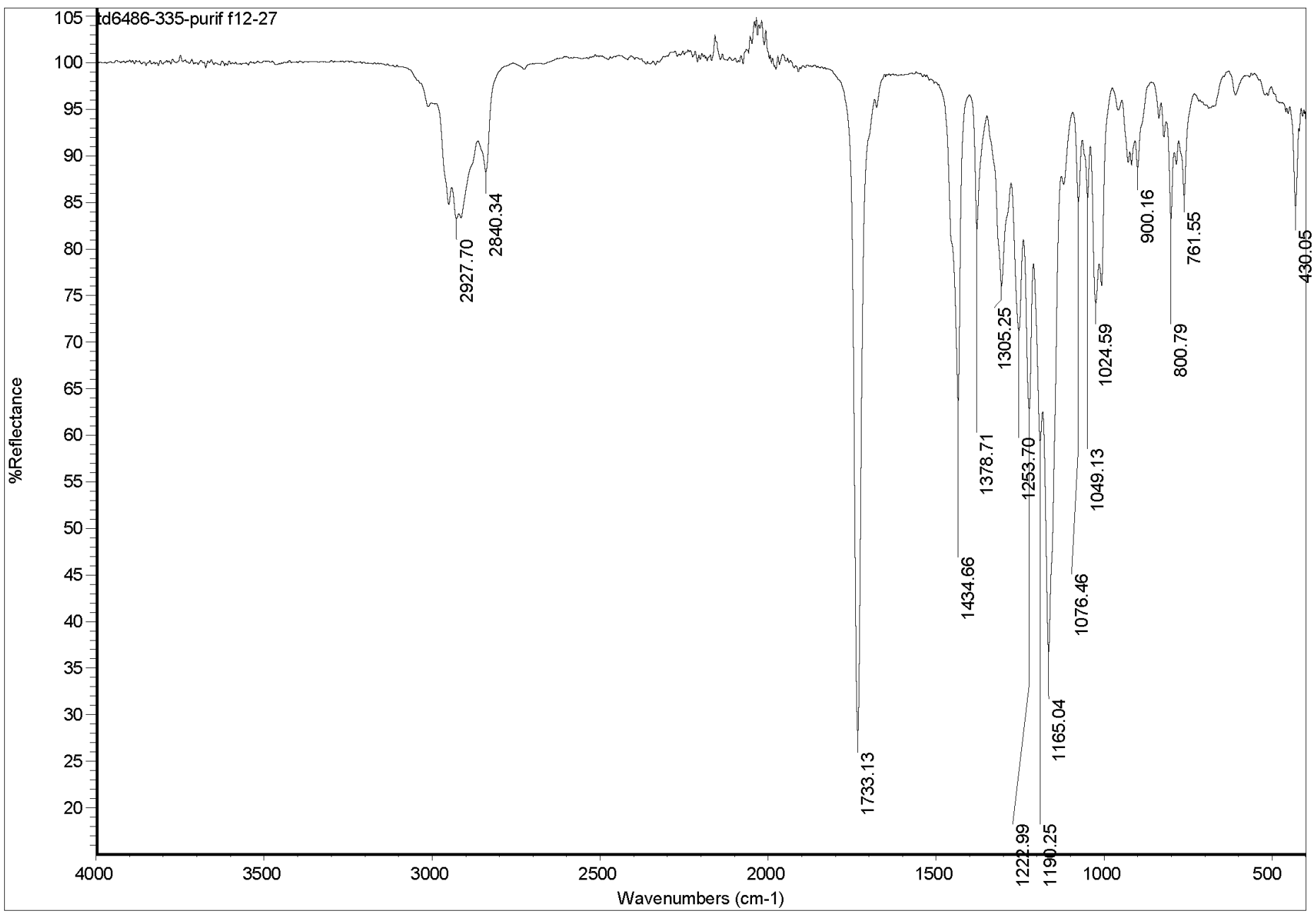

*Figure S71. ATR-IR spectrum of isolated mixture of **6a** and **6b** (2:1 ratio).*

4. 2. 1-(4-Nitrophenyl)pyrrolidine (15):

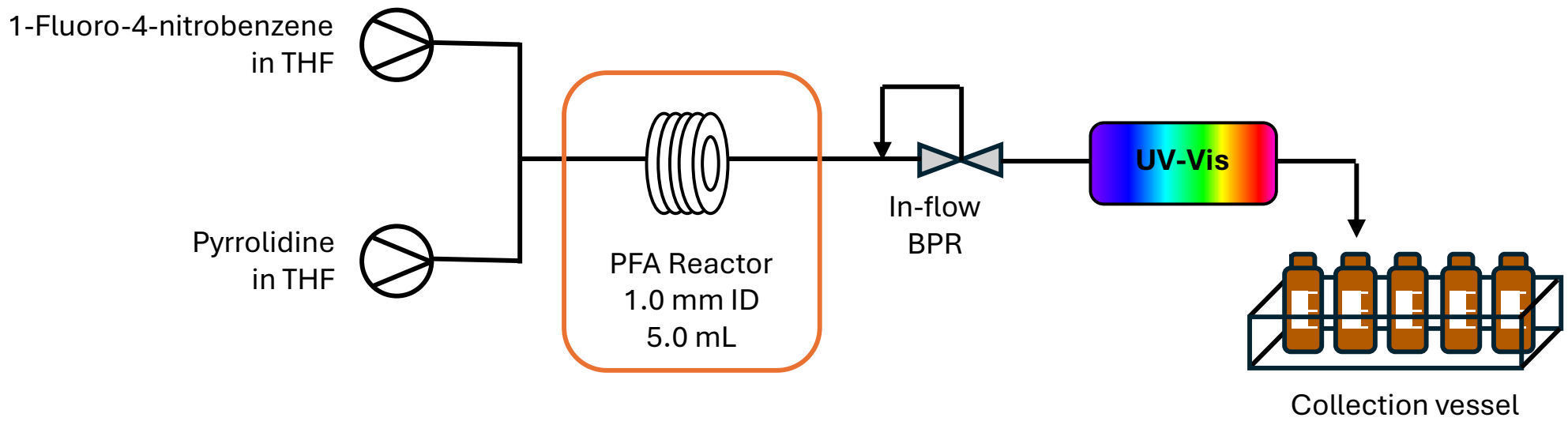

1-Fluoro-4-nitrobenzene (0.364 g, 2.6 mmol, 1.0 eq.) was dissolved in THF (10.0 mL) and connected to the first reagent stream. Pyrrolidine (1.827 g, 26.0 mmol, 10.0 eq.) was dissolved in THF (10.0 mL) and connected to the second reagent stream. THF was connected to solvent streams. Flow system was primed with THF, pumps were switched to deliver reagents into reactor (5.0 mL, PFA, 1.0 mm ID, at rt) with 0.1 mL/min flow rate, each (0.2 mL/min total, resulting in 25 min residence time). 100 psi BPR and UV-Vis flow cells were placed after the reactor. Output of flow system was collected, washed with deionised water (50.0 mL) and extracted with DCM (50.0 mL). Organic layer was collected and solvent removed under vacuum, giving 0.411 g of desired product (82% isolated yield, 2.1 mmol) as a yellow solid. NMR data are consistent with literature.[5]

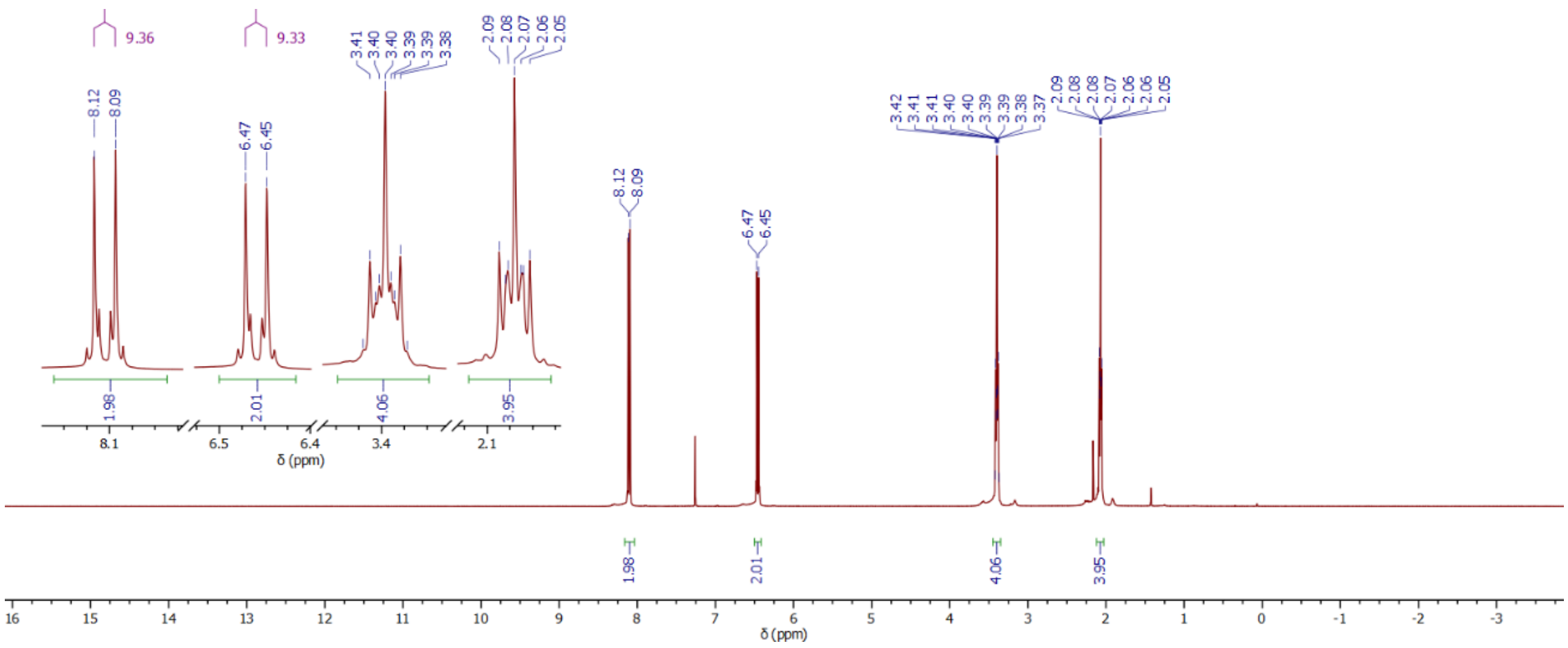

Figure S72. ^{1}H NMR spectrum of 1-(4-nitrophenyl)pyrrolidine.

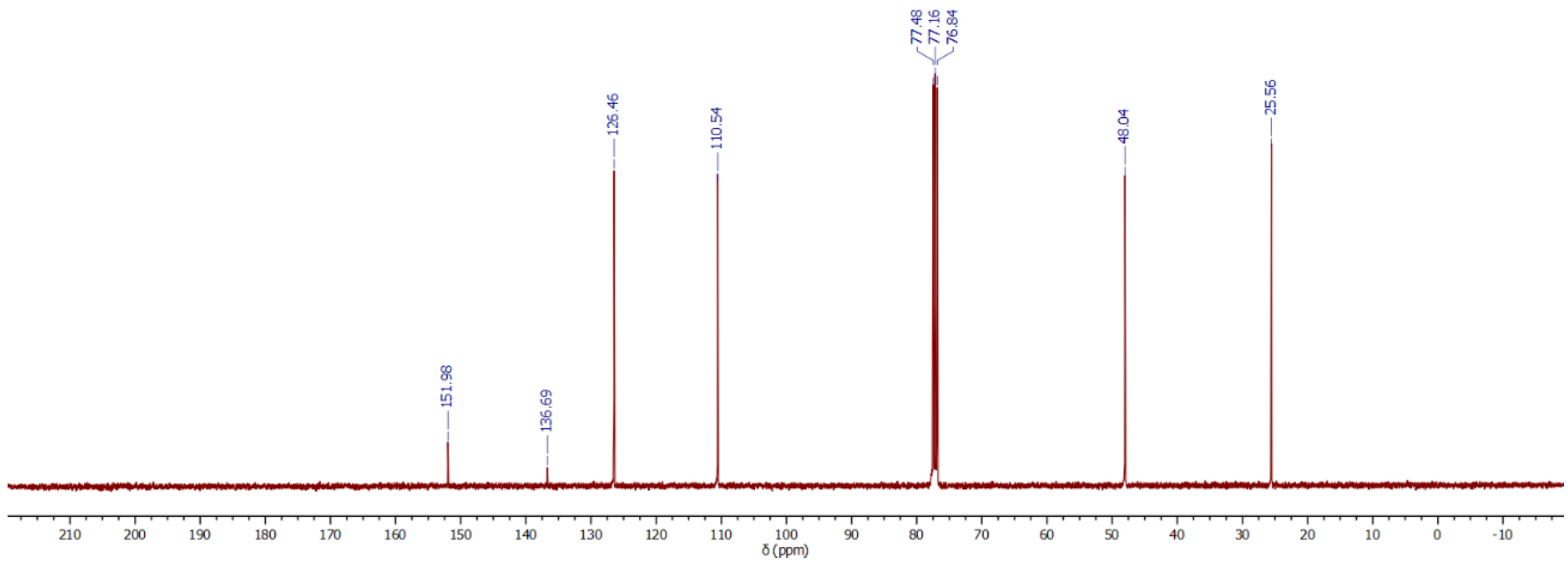

Figure S73. ^{13}C NMR spectrum of 1-(4nitrophenyl)pyrrolidine.

4. 3. 1-(4-Nitrophenyl)piperidine (18):

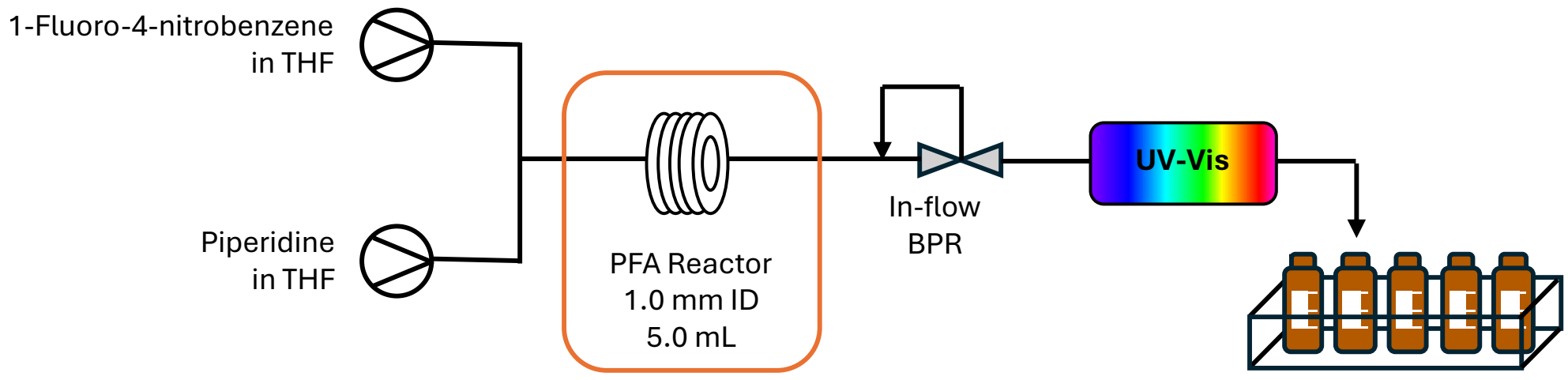

1-Fluoro-4-nitrobenzene (0.364 g, 2.6 mmol, 1.0 eq.) was dissolved in THF (10.0 mL) and connected to the first reagent stream. Piperidine (2.57 mL, 26.0 mmol, 10.0 eq.) was dissolved in THF (10.0 mL) and connected to the second reagent stream. THF was connected to solvent streams. Flow system was primed with THF, pumps were switched to deliver reagents into reactor (10.0 mL, PFA, 1.0 mm ID, at rt) heated to 100 °C with 0.1 mL/min flow rate each (0.2 mL/min total, resulting in 50 min residence time). 100 psi BPR and UV-Vis flow cells were placed after the reactor. Output of flow system was collected, washed with deionised water (50.0 mL), DCM (50.0 mL), then brine (50.0 mL). Organic layer was collected and solvent removed under vacuum, obtained residue was purified by column chromatography (SiO_2, EtOAc:*n*-hexane 3:7, R_f = 0.55). Fractions containing product were collected, solvent removed *in vacuo*, and 0.427 g of desired product (80% isolated yield, 2.1 mmol) was obtained as an orange solid. NMR data are consistent with literature.[6]

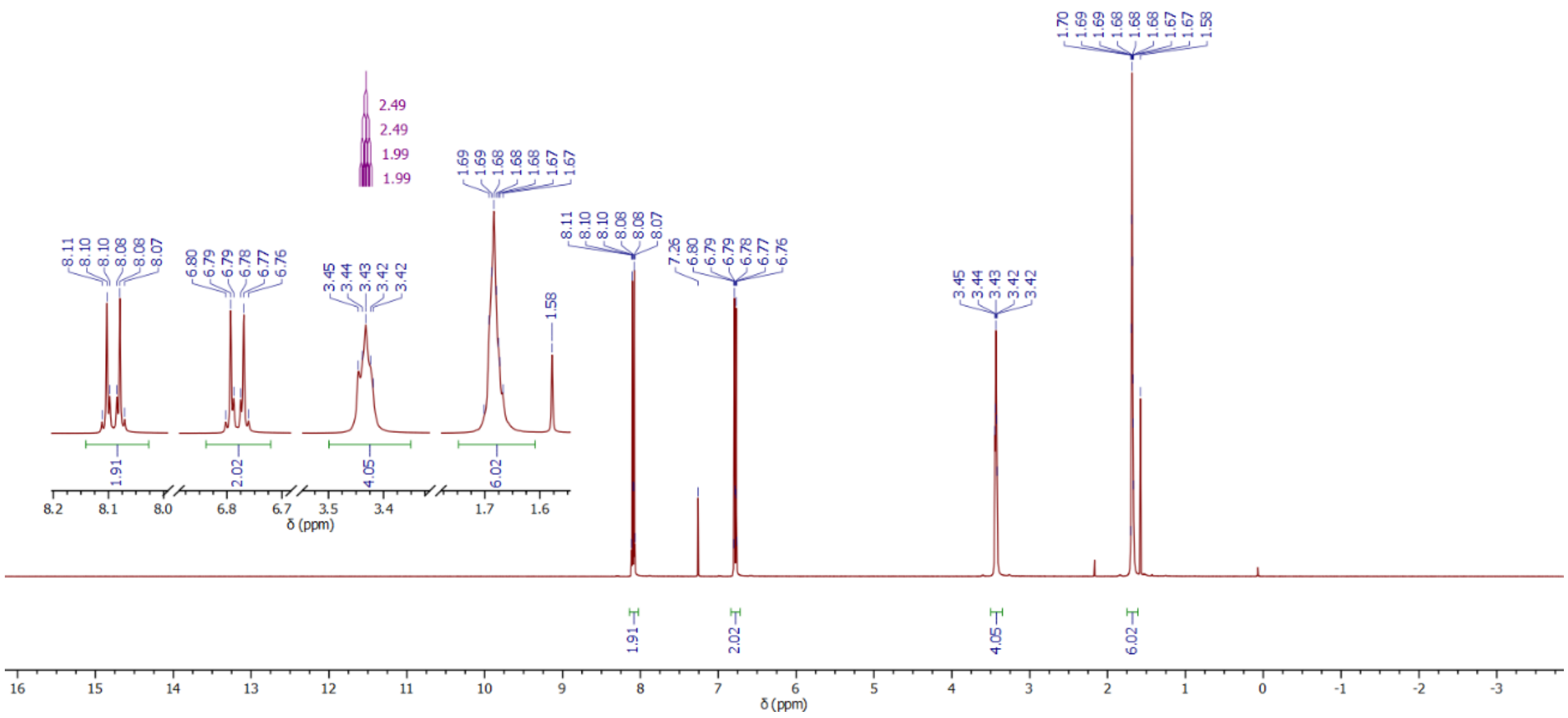

Figure S74. ¹H NMR spectrum of 1-(4-nitrophenyl)piperidine.

4. 4. 1-(4-Nitrophenyl)azepane (19):

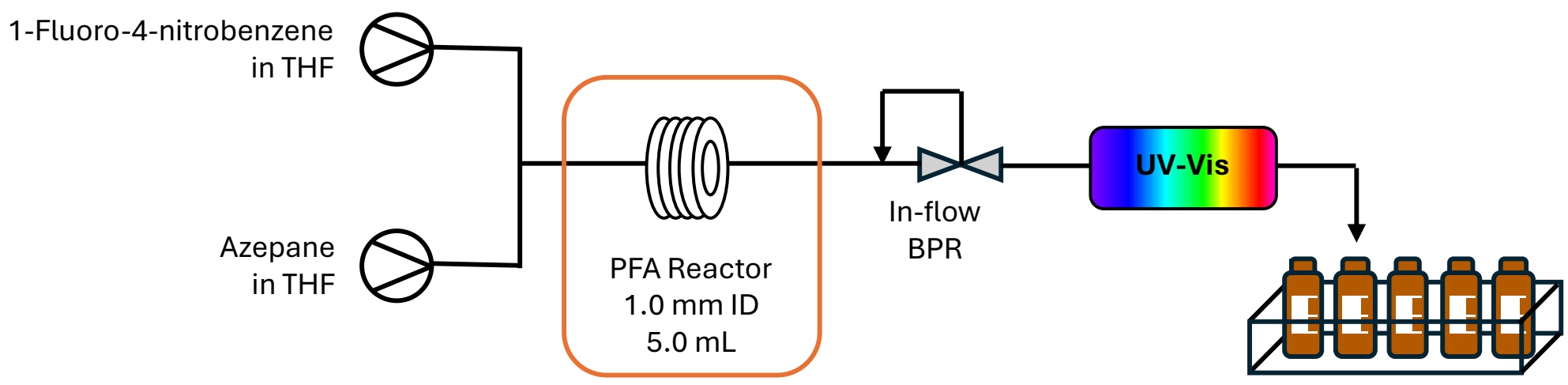

1-Fluoro-4-nitrobenzene (0.364 g, 2.6 mmol, 1.0 eq.) was dissolved in THF (10.0 mL) and connected to the first reagent stream. Azepane (2.93 mL, 26.0 mmol, 10.0 eq.) was dissolved in THF (10.0 mL) and connected to the second reagent stream. THF was connected to solvent streams. Flow system was primed with THF, pumps were switched to deliver reagents into reactor (10.0 mL, PFA, 1.0 mm ID, at rt) heated to 100 °C with 0.1 mL/min flow rate each (0.2 mL/min total, resulting in 50 min residence time). 100 psi BPR and UV-Vis flow cells were placed after the reactor. Output of flow system was collected, washed with deionised water (50.0 mL), 3x DCM (50.0 mL), then 3x deionised water (50.0 mL). Organic layer was collected and solvent removed under vacuum, obtained residue was purified by column chromatography (SiO_2, EtOAc:*n*-hexane 3:7, R_f = 0.50). Fractions containing product were collected, solvent removed *in vacuo*, and 0.466 g of desired product (81% isolated yield, 2.1 mmol) was obtained as an orange solid. NMR data are consistent with literature.[7]

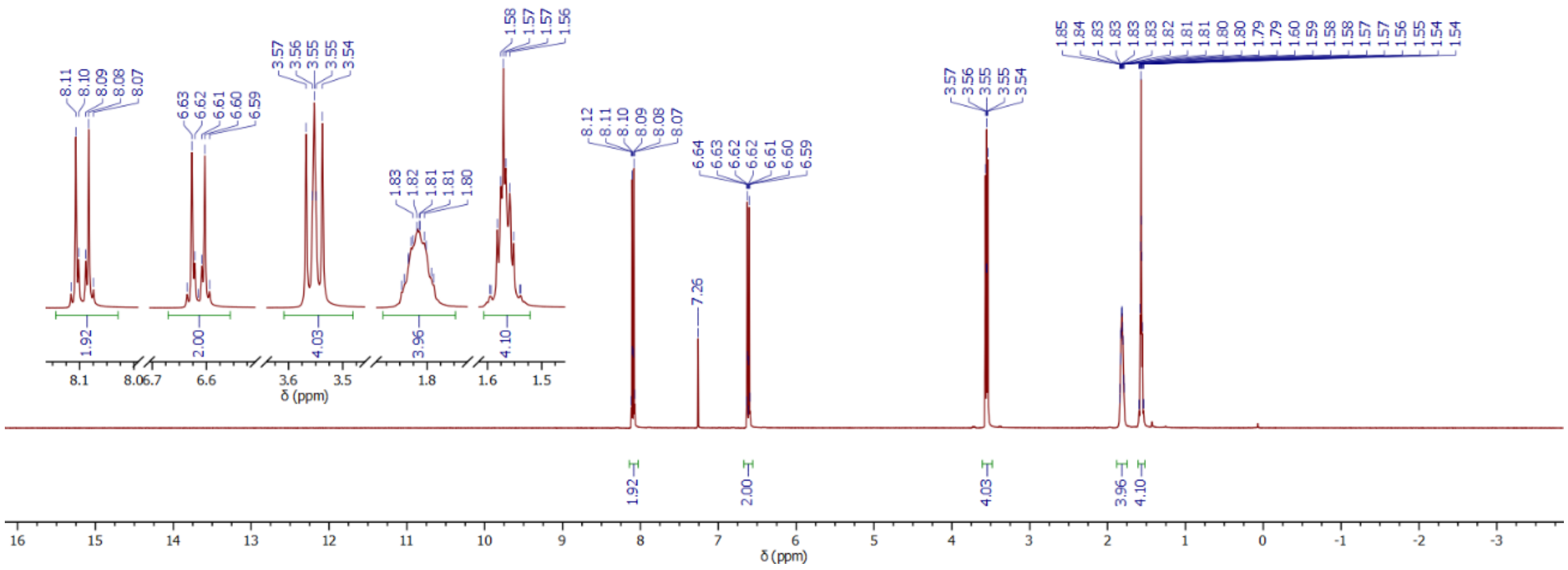

Figure S75. ^{1}H NMR of 1(4-nitrophenyl)azepane.

5. References

1 T. Durand, Use of flow techniques to investigate organic reactions, University of Southampton, PhD Thesis, 2016.

2 L. Eklund, A.-K. Axelsson, Å. Nordahl, R. Carlson, H. Møllendal, A. Sveiczer, Y. Stenstrøm, M. Trætteberg, A. Nasiri and T. Tsuda, *Acta Chem. Scand.*, 1993, **47**, 581–591.

3 K. Nakagawa, M. Sawai, Y. Ishii and M. Ogawa, *Bull. Chem. Soc. Jpn.*, 1977, **50**, 2487–2488.

4 M. R. Friedfeld, G. W. Margulieux, B. A. Schaefer and P. J. Chirik, *J. Am. Chem. Soc.*, 2014, **136**, 13178–13181.

5 G. Toma and R. Yamaguchi, *Eur. J. Org. Chem.*, 2010, **2010**, 6404–6408.

6 J. P. Wolfe and S. L. Buchwald, *J. Org. Chem.*, 2000, **65**, 1144–1157.

7 H. Koshio, F. Hirayama, T. Ishihara, Y. Taniuchi, K. Sato, Y. Sakai-Moritani, S. Kaku, T. Kawasaki, Y. Matsumoto, S. Sakamoto and S. Tsukamoto, *Bioorg. Med. Chem.*, 2004, **12**, 2179–2191.
